# A Blockchain Framework for Patient-Centered Health Records and Exchange (HealthChain): Evaluation and Proof-of-Concept Study

**DOI:** 10.2196/13592

**Published:** 2019-08-31

**Authors:** Ray Hales Hylock, Xiaoming Zeng

**Affiliations:** 1 Department of Health Services and Information Management College of Allied Health Sciences East Carolina University Greenville, NC United States

**Keywords:** blockchain, chameleon hashing, health information exchange, health information management, HL7 FHIR, patient-centered health, medical records, proxy re-encryption, redactable blockchain, smart contracts, digital health, electronic health records

## Abstract

**Background:**

Blockchain has the potential to disrupt the current modes of patient data access, accumulation, contribution, exchange, and control. Using interoperability standards, smart contracts, and cryptographic identities, patients can securely exchange data with providers and regulate access. The resulting comprehensive, longitudinal medical records can significantly improve the cost and quality of patient care for individuals and populations alike.

**Objective:**

This work presents HealthChain, a novel patient-centered blockchain framework. The intent is to bolster patient engagement, data curation, and regulated dissemination of accumulated information in a secure, interoperable environment. A mixed-block blockchain is proposed to support immutable logging and redactable patient blocks. Patient data are generated and exchanged through Health Level-7 Fast Healthcare Interoperability Resources, allowing seamless transfer with compliant systems. In addition, patients receive cryptographic identities in the form of public and private key pairs. Public keys are stored in the blockchain and are suitable for securing and verifying transactions. Furthermore, the envisaged system uses proxy re-encryption (PRE) to share information through revocable, smart contracts, ensuring the preservation of privacy and confidentiality. Finally, several PRE improvements are offered to enhance performance and security.

**Methods:**

The framework was formulated to address key barriers to blockchain adoption in health care, namely, information security, interoperability, data integrity, identity validation, and scalability. It supports 16 configurations through the manipulation of 4 modes. An open-source, proof-of-concept tool was developed to evaluate the performance of the novel patient block components and system configurations. To demonstrate the utility of the proposed framework and evaluate resource consumption, extensive testing was performed on each of the 16 configurations over a variety of scenarios involving a variable number of existing and imported records.

**Results:**

The results indicate several clear high-performing, low-bandwidth configurations, although they are not the strongest cryptographically. Of the strongest models, one’s anticipated cumulative record size is shown to influence the selection. Although the most efficient algorithm is ultimately user specific, Advanced Encryption Standard–encrypted data with static keys, incremental server storage, and no additional server-side encryption are the fastest and least bandwidth intensive, whereas proxy re-encrypted data with dynamic keys, incremental server storage, and additional server-side encryption are the best performing of the strongest configurations.

**Conclusions:**

Blockchain is a potent and viable technology for patient-centered access to and exchange of health information. By integrating a structured, interoperable design with patient-accumulated and generated data shared through smart contracts into a universally accessible blockchain, HealthChain presents patients and providers with access to consistent and comprehensive medical records. Challenges addressed include data security, interoperability, block storage, and patient-administered data access, with several configurations emerging for further consideration regarding speed and security.

## Introduction

### Overview

Health care is a data-intensive domain with vast amounts of information generated, accessed, and disseminated daily. Unfortunately, patient records are typically isolated in institution-centric *electronic health records* (EHRs), resulting in fragmentation with consequences ranging from inefficient care coordination to lack of critical information during emergencies [1-3]. Interoperability requirements were instituted as a remedy, but a system supporting comprehensive patient record integration remains elusive. Furthermore, the *Office of the National Coordinator for Health Information Technology* (ONC), in a 2015 Congressional report, detailed how technology vendors and providers limit patient access, by what has since been codified as *information blocking* [4,5]. Thus, not only do patient data remain disjointed but also barriers erected by data holders dissuade patient engagement and information exchange, culminating in the loss of agency. Blockchain technology coupled with nationally recognized interoperability standards (eg, Fast Healthcare Interoperability Resources, FHIR) has been presented as a viable solution to the said concerns [1-3,6-24].

Traditional health information exchange follows 1 of the following 3 models: *push* (sending information from 1 location to another), *pull* (extracting information from a source), or *view* (peering into a system). Although these practices technically achieve health information exchange, they are not sustainable, en masse solutions to patient-centered care. Thus, Halamka et al [23] proposed blockchains are a fourth model, with a stated goal of restoring patient agency [3,24].

Blockchains are distributed and decentralized repositories of information secured by various cryptographic primitives. Ideally, participants (eg, patients, providers, and payers) upload data to the chain in a secure, authenticated fashion. The result is a comprehensive medical record accessible by those with patient permission as enforced by smart contracts. As participants only need to communicate with the blockchain using recognized interoperability standards (eg, FHIR), once trust is established, all information is securely exchanged. That is, instead of having multiple points of connection, document formats, and exchange protocols to follow (each a security risk and potentially costly to address), a universally accessible blockchain minimizes the overall risk to the participating entity while simultaneously enriching information exchange and patient engagement.

More specifically, deploying blockchain in health care is suggested to break down information exchange barriers inherent in disparate, siloed EHR systems; empower patients through data consolidation and access controls and enabling (eg, secure and verifiable authorizations, form completion, discharge instruction review, and patient-generated data contribution); improve quality of care while reducing costs and fraud; promote data integrity, validation, and provenance; track medical devices and pharmaceuticals; facilitate clinical trial accountability and auditability; and support research through access to large-scale, longitudinal, aggregated patient records [3,6-22]. Virtually, all previous works are proof of concepts or pilots, endeavoring to address the information security, interoperability, data integrity, identity validation, and scalability concerns hindering adoption [3,6-19].

Herein, we submit technical solutions to address these concerns, culminating in a detailed framework and open-source proof-of-concept tool—*HealthChain* —for a patient-centered blockchain. In the Methods section, HealthChain’s components are defined and, when appropriate, compared with an immutable blockchain design. Contributions include a mixed-block blockchain, redactable patient blocks, amendable smart contracts, adoption of *proxy re-encryption* (PRE) for granting and revoking data sharing rights, formulation of a *2-party PRE decryption* (2PD) scheme to facilitate mediated exchange, 4 configurable modes of operation, and a comprehensive set of experiments.

### Blockchain Applications in Health Care

Heralded as a disruptive technology, blockchain research has intensified in recent years. Researchers and developers in health care have proposed, conceptualized, and implemented blockchain-based platforms to transform patient data sharing and information interoperability.

OmniPHR is a patient-centered blockchain emphasizing the distributed and interoperable principles of *personal health records* (PHRs). Patient data are recorded in encrypted, hierarchical blocks signed by the inserting entity (eg, provider, patient, or medical device). As data may be stored off-chain, OmniPHR maintains location pointers [25]. Another well-known system—MedRec—was conceived by researchers at Harvard and MIT. This Ethereum-based system links global patient identities to records held by providers. MedRec authenticates participants and stores provider pointers and record hashes (for data integrity). Patients interface with providers through MedRec to view data through smart contracts. Furthermore, patients manage third party access through the creation of smart contracts [3,23,24]. FHIRChain, developed by Zhang et al [2], is a blockchain-based architecture faceted in accordance with the secure and scalable sharing requirements of the ONC’s Shared Nationwide Interoperability Roadmap [26] and leverages the emerging FHIR standard [27]. As with MedRec, data are stored off-chain and accessible through pointers and smart contract–controlled access tokens. In addition to interoperability, Kuo et al’s [28] ModelChain performs privacy-preserving machine learning in the blockchain [28,29].

Beyond patient-centric applications, other blockchain solutions have been presented in a health care setting including supply chain management [1,12,17,30-32], clinical research and data monetization [14,16,33-36], medical and research fraud detection [34,35,37], public health surveillance [13,18,20,38], and managing internet of (healthy) things [12,39-43].

### Background and Terminology

#### Consensus and Hashing

A *consensus* algorithm is a protocol followed by the blockchain when determining the truthfulness and timeliness of blocks under consideration. On reaching consensus, blocks are accepted or rejected. There are many consensus algorithms from which to choose proof of work [44], proof of stake [45,46], proof of elapsed time [47,48], or Kafka [47,49]—a discussion of each is outside the scope of this work, and we refer the reader to the supplied references for further exploration.

**Figure 1:**
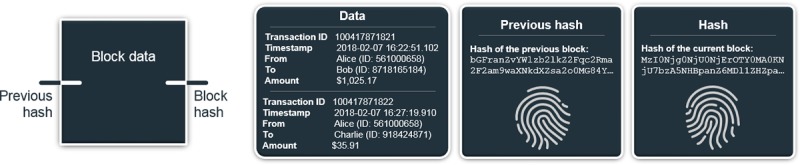
Block schematic with sample financial data and hashes.

**Figure 2:**
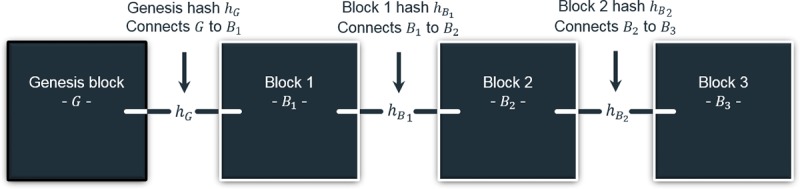
Blockchain diagram with several blocks, including the foundational genesis block, and noted hash connections.

Each block is identified by a *hash*, which is essentially a unique and verifiable fingerprint (Figure 1). A *1-way cryptographic hashing function* produces said hash, representing the content of a *message*. In blockchain, the message consists of block data and the previous block’s hash; the inclusion of the latter creates an unbreakable bond (ie, chain; Figure 2). Hashing functions satisfy 2 key principles: (1) each input has a distinct output (ie, uniqueness), and (2) a given input has the same output (ie, verifiability).

Point 1 seeks to prevent *collisions* —a phenomenon where 2 distinct messages have the same hash. Attackers can theoretically exploit collisions by *forging* blocks with desirable modifications (eg, a financial transfer), replacing authentic blocks as the forged hashes are verifiable (point 2)—this is an oversimplification but suitable for illustrative purposes. Thus, it is imperative to use hash functions without known vulnerabilities or collisions.

#### Chameleon Hashing

Before Ateniese et al’s *redactable blockchain* [50], a block was axiomatically held as immutable. The authors, however, made several keen observations as to when mutability was desirous if not mandatory to comply with the legal, regulatory, and general usability requirements. These include legal violations (eg, illicit content or intellectual property rights infringements), amending changes to information (eg, avoiding data fragmentation [see section on Patient Blocks]), patching executable code (eg, debugging smart contracts or removing an embedded virus), expunging data (eg, right to be forgotten, General Data Protection Regulation, or privacy breach), and allowing for blockchain consolidation (eg, a merger) [7,8,15,50-52].

*Chameleon hashing* was posited as a viable alternative to traditional functions. As proposed by Krawczyk and Rabin [53-55], chameleon hashing satisfies the 2 hashing principles while introducing a *trapdoor*, allowing for efficient generation of collisions by the possessor of the trapdoor (ie, private) key. However, without the said key, collisions are just as unlikely as nontrapdoor functions [50,53-55].

#### Smart Contracts

*Smart contracts* are autonomous transactions executed when stipulated terms of an agreement are met [3,22,51,52,56-59]. The creation of such contracts arose from the need to engender trust in an inherently untrustworthy environment. For instance, if some condition is met, how can one guarantee each party will comply with the agreement? Once executed, it will carry out the specified terms without fail.

Smart contracts, popularized by cryptocurrencies such as Bitcoin and Ethereum, have many practical applications in health care. Patients, for instance, can provide authorization through smart contracts to participate in studies or share information. They can codify rules leading to patient notification, for example, data accessed or communication received. They can also be used as a form of context-based access control, stipulating access rights to covered entities, business associates, and subcontractors [3,21,22].

#### Proxy Re-Encryption

**Figure 3:**
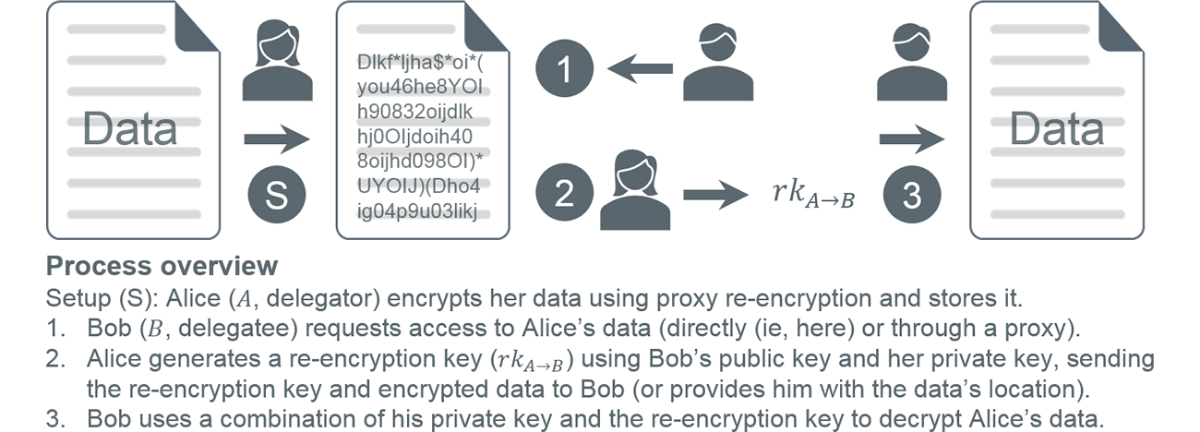
Proxy re-encryption process overview.

PRE enables the *delegation* of decryption rights by a *delegator* to a *delegatee* through an intervening *proxy* [60-63]. The notion is quite intuitive and shown in Figure 3. A user, for example, Alice, encrypts a message forming a ciphertext. Bob requests a *re-encryption key* to facilitate decryption of Alice’s ciphertext without exposing either party’s secret information. PRE has been generally deployed in the cloud [63-70] for network storage [60], distributed file systems [71], email forwarding [65,71], and information exchange [72]. In health care, it has been suggested to safeguard patient data and identities in cloud-based systems [73,74], secure mobile health monitoring and telehealth [66-68,75], and control disclosure of information in PHRs [65] and health information exchanges [72].

Most PRE schemes use *elliptic curve cryptography* (ECC) [76-80], an asymmetric cryptosystem (*Federal Information Processing Standard* [FIPS] 186-4 [81]). Advancements in quantum computing [82-85] and modern attacks [86-88], however, foreshadow its demise. One promising replacement is quantum-resistant *lattices* [71,89-95]. Kirshanova [71] and Kim and Jeong [93] have recently published frameworks for implementing PRE using lattices. Thus, as we enter a postquantum age, so too will PRE, ensuring its longevity.

## Methods

### Proposed HealthChain Framework

Herein, an overview of the HealthChain framework is presented in Textbox 1. Specific details are provided in the proceeding subsections.

#### Patient Centered

As a patient-centric framework, HealthChain presents patients with a holistic view of their medical record, restoring agency through interaction. It encourages the accumulation, modification, generation, and review of information; ensures data integrity; authenticates identities; promotes unambiguous exchange; and executes user-granted access rights through smart contracts. Thus, HealthChain does not suffer from the common ailments plaguing PHR adoption such as data security and validity concerns, interoperability challenges, trust, and technological barriers to adoption [96-98]. Of note, HealthChain is not intended to replace EHRs but to serve as an interface between patients and third parties (eg, providers or payers).

#### Permissioned Blockchain

HealthChain is defined as a *permissioned* blockchain; only trusted parties (eg, hospitals, research institutions, universities, and government agencies) have the authority to manipulate the blockchain within a *private* network. These parties form a consortium (eg, a Regional HealthChain Organization) to manage the HealthChain, ensuring compliance with, for example, relevant statutes (eg, Health Insurance Portability and Accountability Act of 1996, HIPAA) and interoperability standards. Permissioning is further extended to patients, who are validated by a consortium member before account creation; this process can imitate those for patient portals. Although permissioning is a HealthChain requisite, a specific implementation is not. Thus, adopters may incorporate any system of choice.

**The 6 components of the HealthChain framework.**

1. Patient centered
2. Permissioned blockchain: nodes and users
3. Interoperability: nationally recognized interoperability standards
4. Mixed-block blockchain: log and patient blocks
5. Smart contracts: permissioned interoperability
6. Health Insurance Portability and Accountability Act of 1996 and HealthChain: legal requirements and supporting components

#### Interoperability

Patient-managed health information systems must conform to nationally recognized interoperability standards to be successful [99,100]. Using proprietary or lesser known standards (if at all) erects interoperability barriers, diminishing utility and adoption. Although any standard(s) is acceptable in this framework, HL7 FHIR [27] is recommended.

Although not yet federally required, HL7 FHIR is, according to the 2019 Interoperability Standards Advisory by ONC, under consideration for 26 interoperability needs [100]. Furthermore, ONC’s Interoperability Proving Ground (an open community platform for sharing interoperability projects) indicates 21.3% (96/450) of projects use FHIR. Notable participants include Allscripts, the American Medical Association, Cisco, NewYork-Presbyterian Hospital, OpenHIE, the Sequoia project, and the US Department of Veterans Affairs [101]. Apple has also invested in FHIR for their PHR app [102]. Clearly, the anticipation is standard acceptance, thus our selection.

#### Mixed-Block Blockchain

**Figure 4:**
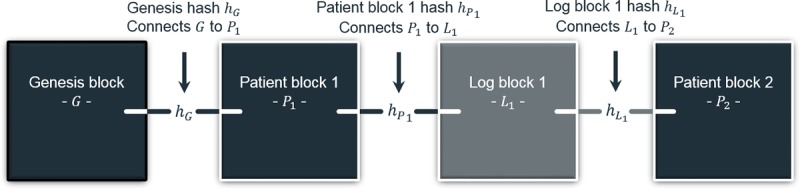
Mixed-block blockchain adaptation of Figure 2.

The proposed blockchain integrates 2 semantically distinct block types: *log* and *patient*, each detailed in subsequent sections. The distinction lies in block *redaction* or editability. Patient blocks are proposed to be redactable, whereas log blocks are not. Figure 4 augments Figure 2 to account for the 2 types. As detailed, the architecture is unaffected. A simple flag in each block distinguishes the types.

##### Log Blocks

Log blocks are an immutable, historical account of operations on the blockchain, such as added patients and blocks, patient block modification metadata, and the issuance and execution of smart contracts. Hence, traditional hashing algorithms (eg, Secure Hash Algorithm [SHA]-256) suffice. In addition, as the data are not sensitive (ie, contain no identifying information), encryption is unnecessary. Thus, log blocks are added to the blockchain following contemporary means (eg, through consensus).

##### Patient Blocks

Structurally, patient blocks consist of plaintext metadata (eg, unique identifier, type flag, patient’s anonymous identifier, timestamp, hash, and the issuing miner’s identifier and signature), encrypted patient data, and smart contracts. Principally, they adhere to Ateniese et al’s redaction scheme [50] and are established and updated as follows.

**Figure 5:**
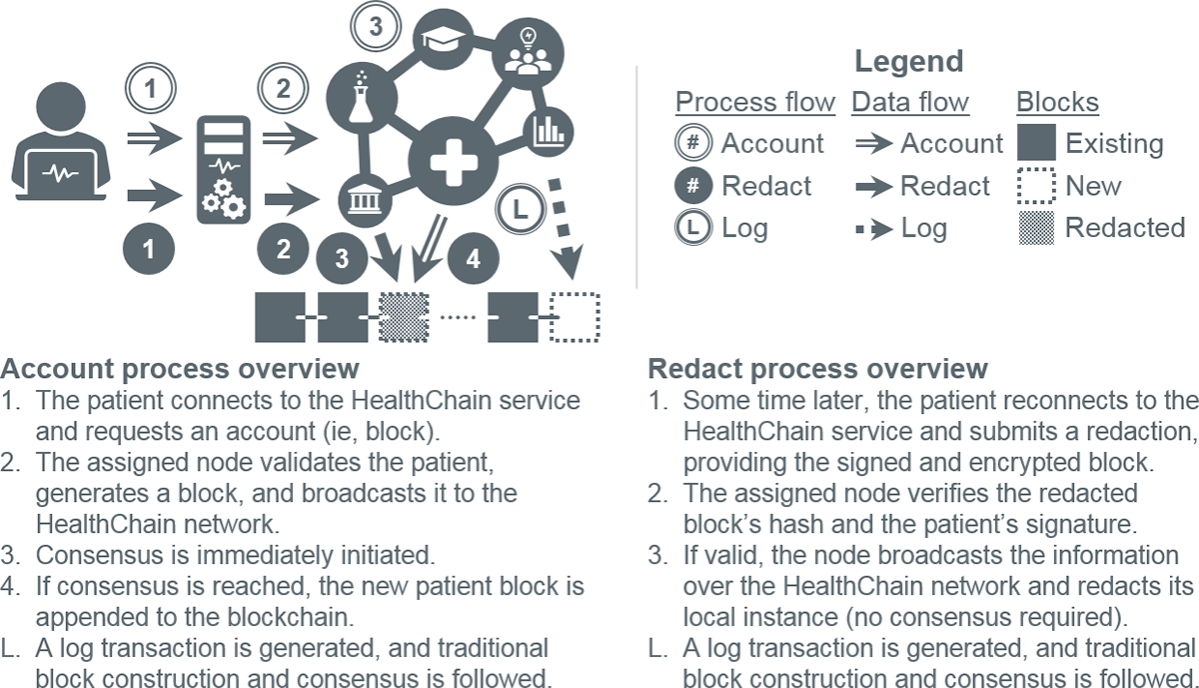
Patient account (ie, block) establishment, redaction, and logging processes in HealthChain.

When a patient requests an account, an authorized node prepares a block and submits it through a selected consensus method for inclusion. This represents the only instance of consensus in the patient block process (Figure 5, account flow). Once accepted, the patient assumes control. Block alterations are hashed, signed, and broadcasted to the network. Nodes validate the transactions and apply the addendums (Figure 5, redact flow). Multimedia data require special handling, as their large size disproportionately (likely prohibitively) consumes bandwidth and computational resources. We adopt the methodology in MedRec [3], where data remain at the source with a location reference stored in the block for ad-hoc retrieval. Throughout this process, transactions in the form of logs are collected. As stated in the previous section, log blocks are added following a traditional consensus methodology and the chain amended (Figure 5, log flow).

**Figure 6:**
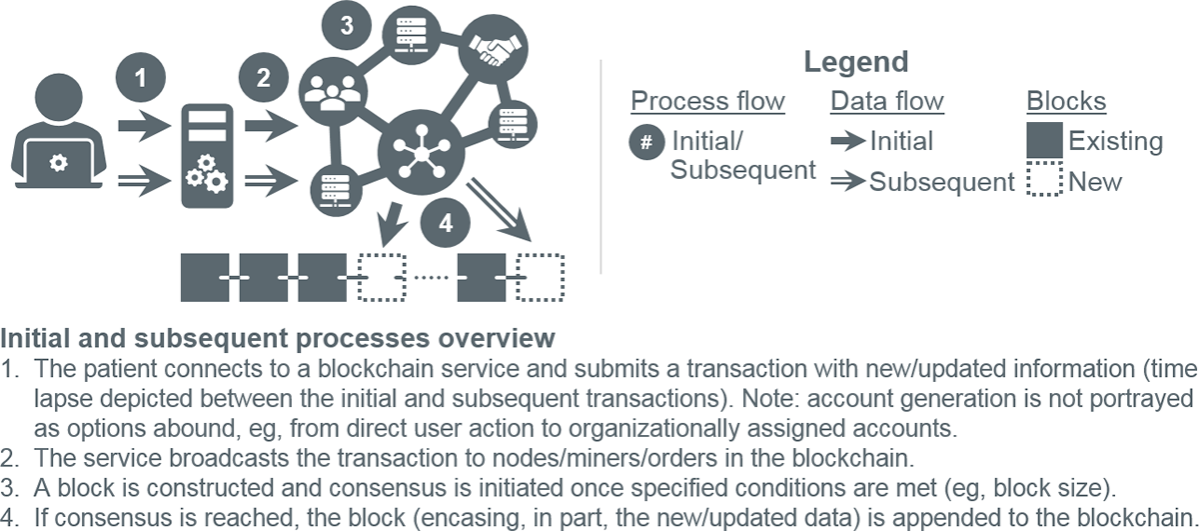
Initial and subsequent patient data entry in a traditional blockchain.

**Figure 7:**
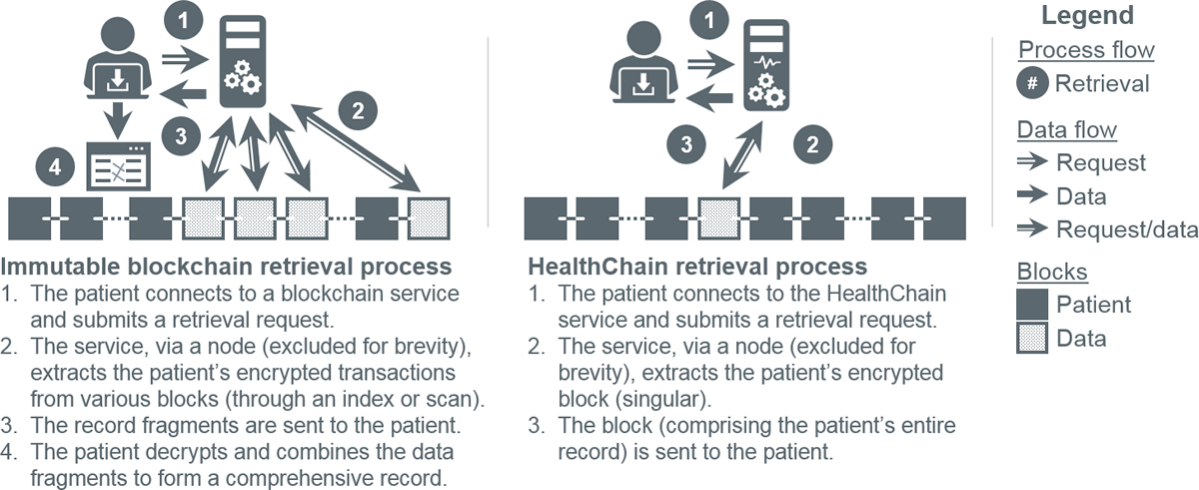
Information retrieval in an immutable blockchain versus HealthChain.

Redaction addresses 3 shortcomings of immutable blocks and patient transaction isolation. The first is data fragmentation. Immutable blockchains insert new transactions in contemporaneous blocks, splintering medical records, which are collections of temporal events, rather than isolated, for example, financial, transactions (Figure 6, initial and subsequent flow). Retrieval, consequently, necessitates (1) blockchain (or a potentially corruptible, off-chain index) scanning by nodes to recover and transmit encrypted fragments and (2) decrypting and reconstituting said fragments by requesters (Figure 7). The greater the fragmentation, the more resource intensive the process. Redaction, as implemented in HealthChain, colocates patient information (Figure 5, redact flow), minimizing overall system effort (Figure 7). Second is immutability itself. Legitimate modifications (eg, adding encounter notes, modifying medical histories, or removing incorrect user-generated data) are simulated in immutable blockchains through new transactions. Requesters must apply these transactions in the proper temporal order (eg, overwrite older data with newer) to reconstruct consistent records. Resources (ie, time, space, and bandwidth) are thus depleted with each modification. Redaction modifies data in place, nullifying this effect. The third, and final, shortcoming is consensus. Modifications produce new blocks necessitating consensus. Redactions to established blocks avoid this costly process, conserving time, effort, bandwidth, and storage for users and nodes.

###### Hashing Redactable Blocks

Once appended to the blockchain, a block’s hash cannot change; otherwise, the chain breaks. Redaction, therefore, precludes traditional hash functions (eg, SHA-256) as, by definition, different inputs produce distinct hashes. Hence, the deployment of chameleon hashing.

Ateniese et al [50] identified several chameleon hash formulations suitable for blockchains. Here, *public-coin chameleon hashing* (PCCH) is applied. PCCH uses public-key cryptography for verification (using public keys) and redaction (by generating collisions with private keys). In terms of security, PCCH hashes are as hard to forge as nontrapdoor functions. Hence, PCCH maintains a secure and valid state in redactable blockchains [50].

###### Block Encryption

Classified as *electronic protected health information* under the HIPAA, patient data must be encrypted (45 Code of Federal Regulations [CFR] section 164.304) per the US Department of Health and Human Services–issued guidelines (Health Information Technology for Economic and Clinical Health 13402(h)(2)). These guidelines reference National Institute of Standards and Technology Special Publication 800-111, which recommends the Advanced Encryption Standard (AES), although any FIPS-approved cryptosystem (eg, ECC) is acceptable [103]. Any selection must be mindful of the proposed framework, which includes information exchange.

Traditional symmetric (eg, AES) and asymmetric (eg, ECC) primitives are insufficient as they compel 1 of the 3 insecure or infeasible information exchange solutions [69]. The first is exchanging secret information. This jeopardizes data integrity (through altering, corrupting, or re-encrypting data) and digital identities (secured by private keys). Second, originators (eg, patients) can re-encrypt data under requester public keys. Although secure, originators must be omnipresent and dedicate considerable personal resources to the process; otherwise, sharing ceases. Finally, third parties can represent originators for re-encryption. However, this exposes plaintext and secret information to the said third parties. As a potential solution to these challenges, we endorse PRE.

PRE facilitates information exchange through dedicated third parties without exposing sensitive information. Beyond proposing PRE, we identified 4 operations critical to securing and validating stored and exchanged data in the proposed framework: (1) encryption and decryption, (2) re-encryption, (3) sign and verify (eg, digital signatures), and (4) encrypt sign and decrypt-verify (eg, encrypted sign and verify). *AFGH* (so named for the authors’ last names) [60] is the chosen PRE scheme as it fulfills operations 1 and 2 and encourages the formulation of 3 and 4. In addition, its re-encryption keys are *unidirectional* (decrypt only), *noninteractive* (no secret information exchanged), and *nontransitive* (cannot combine keys to forge privileges) [60]; Multimedia Appendix 1 provides a more thorough introduction.

Operations 1 and 2 are instrumental in securing and sharing sensitive information and are the foundation of any PRE scheme. Operation 3 facilitates message authentication and data integrity. A message *signed* (ie, encrypted) by a sender using its private key can be *verified* (ie, decrypted) by anyone with its public key. As only the sender has its private key, verification proves authenticity and integrity. Multimedia Appendix 1 offers our posited *sign and verify* AFGH modification. Operation 4 unites 1 and 3 to protect sensitive, signed messages. Signed messages are encrypted with the recipient’s public key, ensuring only it can decrypt before verification. Multimedia Appendix 1 presents the conceived *encrypt-sign* and *decrypt-verify* AFGH extension.

Moreover, some PRE-derived ciphertexts (eg, *ElGamal* based [104] in AFGH) are *malleable*. This is advantageous when optimizing, for instance, data rekeying, which amounts to multiplication in ElGamal, instead of decrypt-encrypt in AES. In addition, it is compulsory for *homomorphic encryption*, that is, computation on ciphertexts. Although this is an active area of research, *fully* homomorphic schemes (ie, those that add *and* multiply) are presently impractical [94,95,105-111]. The research, however, is progressing, with lattices emerging as a promising area [94,95,105,106]. Lattices, therefore, have the potential to bring postquantum, fully homomorphic encryption to this framework.

PRE is traditionally deployed for key exchange [60,76]. Data are encrypted using, for example, AES, and the key encrypted and exchanged through PRE. An alternative, as proposed herein, is PRE-encrypted data. Both have merits and were extensively tested. Table 1 compares these approaches by 4 properties. We focus on PRE implementations over elliptic curves (ECs) because of their prominence [76-80].

EC PRE is slower and larger than AES because of key and *bilinear map* sizes. EC keys are twice AES for the same cryptographic strength—for example, EC-256 equates to AES-128 [112]. Bilinear maps (see Multimedia Appendix 1) are even larger—for example, up to 3072 bits for EC-256 [112-115]. Consequently, the system uses more space and time, hindering performance. Furthermore, AFGH eliminates re-encryption data integrity concerns through decrypt-only keys [60], a property unsupported by AES. Finally, the malleable AFGH cipher supports dynamic rekeying (ie, altering encryption keys) through multiplication, instead of the delegator having to decrypt, encrypt, and retransmit all data as under AES.

**Table 1:** A comparison of Advanced Encryption Standard encrypted blocks with proxy re-encryption encrypted keys to proxy re-encryption encrypted blocks.

Property	AES^a^ block encryption	PRE^b^ block encryption
Encrypt and decrypt speed	Faster	Slower
Size of ciphertext	Smaller	Larger
Key operations	Decrypt and encrypt	Decrypt only
Rekeying ciphers	Decrypt then encrypt	Multiplication

^a^AES: Advanced Encryption Standard.

^b^PRE: proxy re-encryption.

#### Smart Contracts

Smart contracts herein enable conditional information exchange. Their mutability (from patient block storage) permits modification and revocation without duplicate, conflicting, or vulnerable contracts remaining on the blockchain. During instantiation, a template is automatically populated (once authorization is furnished); no programming is necessary. On execution, an engine hardcoded into the server platform applies a series of instructions, given a contract’s parameters. There are several reasons for this approach. The proposed smart contracts are structurally uniform (eg, identifiers and signatures of involved parties, terms, and re-encryption keys), eliminating the need for arbitrary code support. Furthermore, logical errors [56-58] and exploitable vulnerabilities [51,52,58,59] in programmed contracts can compromise patient data. An automated, templatized design with a parameterized, hardcoded engine eliminates these vulnerabilities.

**Figure 8:**
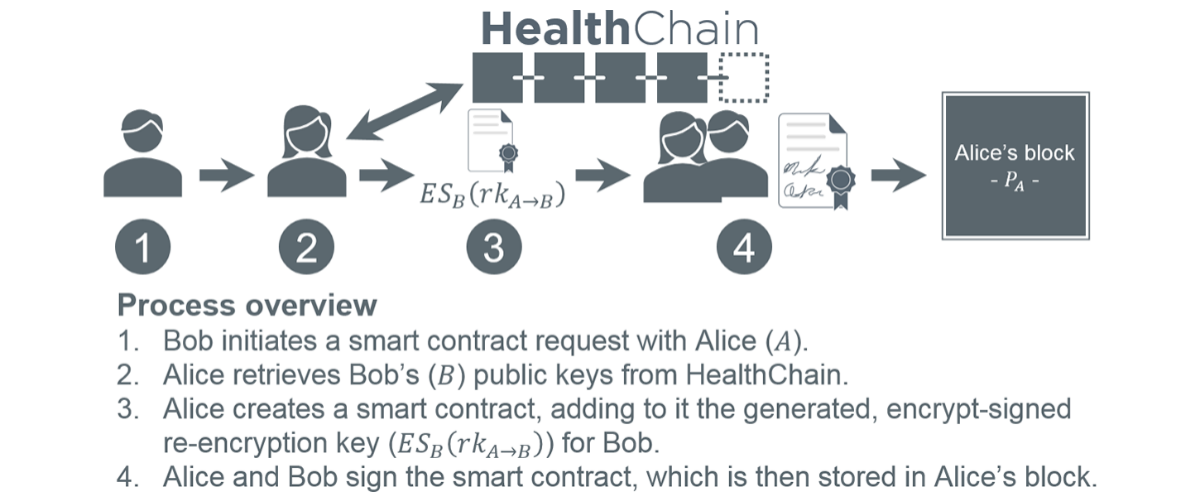
Smart contract initiation using standard proxy re-encryption.

**Figure 9:**
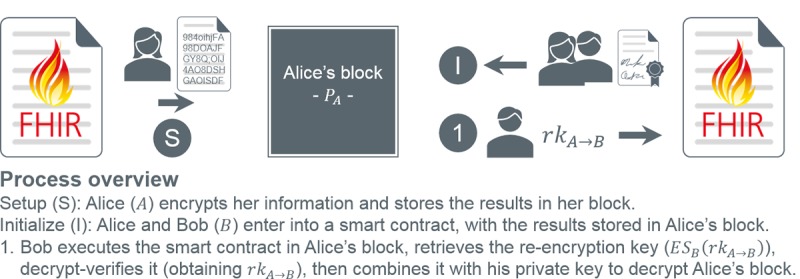
Smart contract execution using standard proxy re-encryption. FHIR: Fast Healthcare Interoperability Resources.

Figure 8 demonstrates the smart contract initiation process, in which a re-encryption key is created and stored in a signed contract on the delegator’s block. Figure 9 shows the execution of the contract generated in step 1 by the delegatee in step 2, facilitating data decryption. In this example (and proof of concept), data are stored as FHIR messages for direct interoperability with capable systems. This represents a basic application of PRE to smart contracts. It is, however, insecure.

Although granting access with PRE is simple, revoking it is not. Consider the following, 2 parties enter into a 1-week smart contract. If the delegatee notes the re-encryption key during valid execution, nothing explicitly prevents it from decrypting the delegator’s block after contract termination.

A naïve refinement to close this vulnerability is implementing PRE as originally defined—proxies decrypt delegator data, then encrypt for delegatees [61]. However, as numerous studies have concluded, proxies cannot be trusted with delegator private keys and plaintext [65,70,90,116]. Another approach is subkeying by, for instance, time [60,117-119]. Each period has a unique, random variable to which all ciphertexts and re-encryption keys are bound. It is argued that this ensures delegatees cannot access *new* information. However, if a contract is terminated within a period, new information *will* be available as access is not revoked, only confined. In addition, one must manage many keys, data are fragmented over time, and interperiod interoperability is cumbersome.

**Figure 10:**
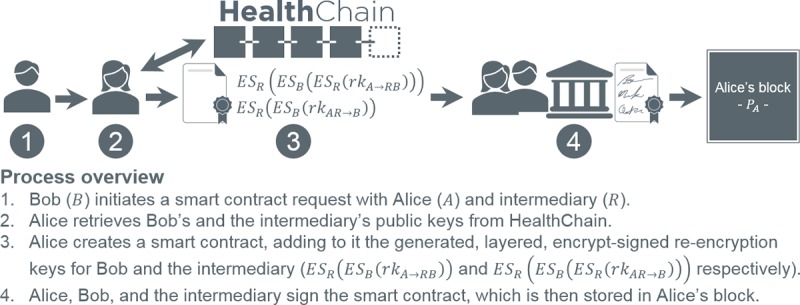
Smart contract initiation using 2-party proxy re-encryption decryption.

**Figure 11:**
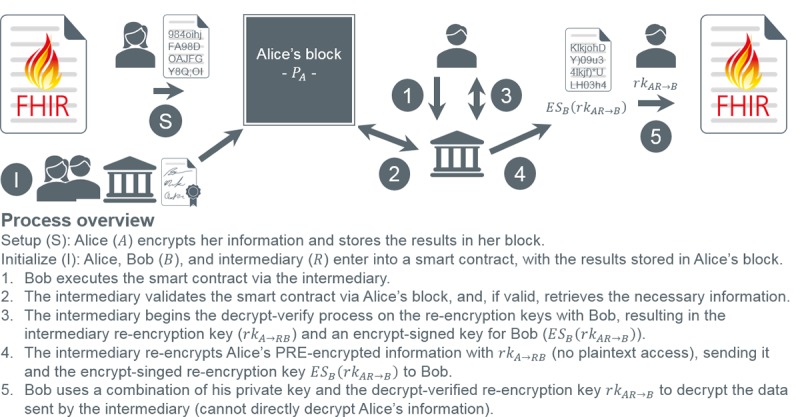
Smart contract execution using 2-party proxy re-encryption decryption. FHIR: Fast Healthcare Interoperability Resources; PRE: proxy re-encryption.

To address revocation, we submit 2PD (Multimedia Appendix 1), a variant of the original formulation [61] whereby data decryption requires 2 parties with complementary re-encryption keys. Figures 10 and 11 adapt Figures 8 and 9, respectively, to 2PD. In premise, the *intermediary* (eg, node; an augmented proxy, thus the distinction) applies its re-encryption key to the delegator’s data (as it is malleable), producing a ciphertext discernable to the delegatee alone. In terms of security, intermediaries no longer require private keys, and its re-encryption key does not expose plaintext. Furthermore, delegatee re-encryption keys cannot decrypt data on the blockchain, thus realizing revocation.

There are several additional 2PD factors to consider. First, it demands an intermediary be *semitrusted* in that it will not maliciously alter the hardcoded smart contract engine or distribute keys to unauthorized entities. As our framework implements a permissioned blockchain, intermediaries (ie, nodes) are inherently trustworthy. That said, private keys and plaintext are withheld to deter collusion and improper data use. Second, re-encryption keys must mathematically prohibit manipulation resulting in forged privileges. This is imparted by AFGH’s nontransitive property [60]. Finally, re-encryption key retrieval mandates intermediary and delegatee consent without revealing said keys. Encrypt-sign *layering*, as documented in Figures 10 and 11, provides such support, as 1 layer must be decrypt verified by the opposing entity (ie, consent) and the final layer by the intended recipient, simultaneously averting unilateral access and key exposure.

#### Health Insurance Portability and Accountability Act of 1996 and HealthChain

Administrative rule 45 CFR section 164.524 grants patients the right to request copies of their records, which are to be delivered *in the form and format requested by the individual, if they are readily producible in such as format* (45 CFR section 164.524(c)(2)(i)). With the growing acceptance of HL7 FHIR (section Interoperability), one can envisage a future with it being *readily producible*. Thus, the proposed framework can leverage patient access rights through FHIR to seamlessly communicate with health information systems, eliminating the burden of ad-hoc data extraction, manual data entry, and data transformation placed on users and providers.

Systems in this space must be HIPAA compliant. Table 2 maps facets of the posited framework to pertinent HIPAA administrative rules, suggesting compliance. A thorough assessment, however, must be conducted before deployment.

**Table 2:** Health Insurance Portability and Accountability Act of 1996 administrative rule specifications (privacy rule and security rule) and submitted HealthChain components supporting compliance.

Specification	Rule: 45 CFR^a^ section 164	HealthChain
Authorization and revocation (PR^b^)	508, 510	Smart contracts, sign and verify, and encrypt-sign and decrypt-verify: confidential communications and verifiable requests and authorizations
Restriction requests (PR)	522(a)(1)	Smart contracts, sign and verify, and encrypt-sign and decrypt-verify: confidential communications and verifiable requests and authorizations
Amendments (PR)	526	Smart contracts, sign and verify, and encrypt-sign and decrypt-verify: confidential communications and verifiable requests and authorizations
Confidential communications (PR)	522(b)(2)	Encrypt-sign and decrypt-verify: message integrity, verifiable identity, and encryption
Unique user authentication (SR^c^)	312(a)(2)(i)	Unique encryption and hashing key pairs, sign and verify, and encrypt-sign and decrypt-verify: verifiable identity (key possession and signing) and patient block hashes and patient block encryption
Encryption and decryption (SR)	312(a)(2)(iv)	Unique encryption and hashing key pairs, sign and verify, and encrypt-sign and decrypt-verify: verifiable identity (key possession and signing) and patient block hashes and patient block encryption
Integrity (SR)	312(c)(1)	Unique encryption and hashing key pairs, sign and verify, and encrypt-sign and decrypt-verify: verifiable identity (key possession and signing) and patient block hashes and patient block encryption
Audit controls (SR)	312(b)	Log blocks
Person or entity authentication (SR)	312(d)	Sign and verify, encrypt-sign and decrypt-verify, re-encryption key layering, and delegatee re-encryption: verifiable identity (verification algorithms and construction of the delegatee re-encryption process)
Transmission security—integrity controls and encryption (SR)	312(e)(1), (2)(i), and (2)(ii)	Patient block encryption, intermediary re-encryption, sign and verify, and encrypt-sign and decrypt-verify (layering): verifiable identity and transfer of encrypted data only by design

^a^CFR: 45: Code of Federal Regulations.

^b^PR: privacy rule.

^c^SR: security rule.

### Experimental Design

The experimental design facilitates the examination of HealthChain’s novel components. Additional services such as permissioning and consensus along with a comprehensive distributed network were not evaluated for the following reasons. First, although essential to the framework’s practical implementation (whereas here we are exploring new functionality), no improvements to those areas were proposed in this work; hence, testing is unwarranted. Second, ancillary services and an arbitrarily sized experimental network inject considerable overhead (potentially orders of magnitude above the measured item) into the process, rending the subject of analysis indistinguishable from noise. As such, we intentionally limited the components implemented in the proof of concept to only those necessary to successfully evaluate the processes defined in the subsequent sections.

Regarding experimental block operations, recall that as patient blocks are generated during account initialization, all subsequent actions necessitate redactions to said block section Patient Blocks). Therefore, all experiments conducted herein are redactions.

#### Configurable Modes

HealthChain operations are dictated by 4 configurable modes (see Multimedia Appendix 2 for a detailed summary). The first is the 2-option *block encryption mode*: (1) AES-encrypted data with PRE-encrypted key (denoted as A) and (2) PRE-encrypted data (signified by P), as defined and justified in section Block Encryption.

The second is the *storage mode* with 2 possibilities: *full block* (F) and *incremental* (I). Full block incorporates all data into a single block. Incremental, in all but 1 case (see next mode), transmits only new and modified entries or removal instructions. Table 3 compares the 2 options over 6 properties. Full block transmits 1 block (ie, transaction), which is more efficient than multiple blocks. Cipher padding in full block mode is negligible, whereas potentially considerable in incremental mode (cumulative padding). Record isolation is trivial in incremental mode as each is its own entry. Full block masks transactions in an encrypted block, making isolation and metadata attacks difficult. Regarding transmission speed and size, incremental mode may be smaller and thus faster if only transmitting minimal changes relative to the medical record. Finally, a single block is bound in size by the storage mechanism (eg, a database byte array attribute). Larger medical records may require multiple blocks, fragmenting the information and increasing the complexity of management. Incremental does not suffer from this limitation.

**Table 3:** Comparison of full block and incremental storage mode options.

Property	Full block	Incremental
Transactions	1	1 or more
Cipher padding	Negligible	Potentially considerable
Record isolation	No	Yes
Speed of transmission	Slow	Potentially fast
Size of transmission	Large	Potentially small

Third is *encryption key mode*, with *static* (S) and *dynamic* (D) choices. Static mode encrypts a patient block using the same key in perpetuity, whereas dynamic mode re-encrypts data under a new one with each update. Dynamic mode enhances security, as compromised information is of limited use, but consumes more resources. AES-encrypted data in incremental block storage (AI) mode requires the patient to re-encrypt and transmit all data. Being malleable, PRE-encrypted data in incremental storage (PI) mode encrypts updates using a new key, sending them and a scalar to a node for appending and rekeying, respectively.

*Server-side encryption mode* is the last mode: simply on (Y) or off (N). If enabled, the server encrypts (by block encryption mode) user data using an ephemeral key for each entry, renewed under the dynamic key policy (does not impact PRE operations). This protects against improper access by dynamically rekeying accessed entries.

Tables 4 and 5 present listings of configurable mode abbreviations and descriptions used in our experiments by AES and PRE encryption respectively. Refer to Multimedia Appendix 2 for more details.

**Table 4:** Advanced Encryption Standard (AES) configurable experimental mode abbreviations and descriptions.

Mode	Description
AF	AES-encrypted data, full block storage
AI	AES-encrypted data, incremental storage
ADF	AES-encrypted data, dynamic encryption key full block storage
ADI	AES-encrypted data, dynamic encryption key incremental storage
ASF	AES-encrypted data, static encryption key full block storage
ASI	AES-encrypted data, static encryption key incremental storage
AFN	AES-encrypted data, full block storage no server-side encryption
AFY	AES-encrypted data, full block storage server-side encryption
AIN	AES-encrypted data, incremental storage no server-side encryption
AIY	AES-encrypted data, incremental storage server-side encryption
ADFN	AES-encrypted data, dynamic encryption key, full block storage, no server-side encryption
ADFY	AES-encrypted data, dynamic encryption key, full block storage, server-side encryption
ADIN	AES-encrypted data, dynamic encryption key, incremental storage, no server-side encryption
ADIY	AES-encrypted data, dynamic encryption key, incremental storage, server-side encryption
ASFN	AES-encrypted data, static encryption key, full block storage, no server-side encryption
ASFY	AES-encrypted data, static encryption key, full block storage, server-side encryption
ASIN	AES-encrypted data, static encryption key, incremental storage, no server-side encryption
ASIY	AES-encrypted data, static encryption key, incremental storage, server-side encryption

**Table 5:** Proxy re-encryption (PRE) configurable experimental mode abbreviations and descriptions.

Mode	Description
PF	PRE-encrypted data, full block storage
PI	PRE-encrypted data, incremental storage
PDF	PRE-encrypted data, dynamic encryption key full block storage
PDI	PRE-encrypted data dynamic encryption key incremental storage
PSF	PRE-encrypted data, static encryption key full block storage
PSI	PRE-encrypted data, static encryption key incremental storage
PFN	PRE-encrypted data, full block storage no server-side encryption
PFY	PRE-encrypted data, full block storage server-side encryption
PIN	PRE-encrypted data, incremental storage no server-side encryption
PIY	PRE-encrypted data, incremental storage server-side encryption
PDFN	PRE-encrypted data, dynamic encryption key, full block storage, no server-side encryption
PDFY	PRE-encrypted data, dynamic encryption key, full block storage, server-side encryption
PDIN	PRE-encrypted data, dynamic encryption key, incremental storage, no server-side encryption
PDIY	PRE-encrypted data, dynamic encryption key, incremental storage, server-side encryption
PSFN	PRE-encrypted data, static encryption key, full block storage, no server-side encryption
PSFY	PRE-encrypted data, static encryption key, full block storage, server-side encryption
PSIN	PRE-encrypted data, static encryption key, incremental storage, no server-side encryption
PSIY	PRE-encrypted data, static encryption key, incremental storage, server-side encryption

#### Experiments

A total of 5 system dimensions are measured over 16 mode combinations for AES-128 and EC-256: transmission size, network latency, client processing time, server processing time, and smart contract execution.

*Transmission size* refers to the number of bytes sent from client to server. Typical consumer internet connections have low upload rates as households consume more content than contribute; thus, upload bandwidth is a concern. Scalability for users with metered connection is also of interest, as they may incur costs associated with overages or plan adjustments.

*Network latency* assesses the effect internet-based transmissions have on the proposed framework. The results are analyzed independently and integrated into client processing time and smart contract execution.

*Client processing time* represents the time devoted by clients to, for example, insert records, generate synchronization instructions, regenerate smart contracts (if dynamic keying), compute block hashes, and broadcast the previous to the blockchain.

*Server processing time* is the time incurred by servers during, for example, AES PRE key renewing (dynamic AES), instruction application (all), PRE scalar multiplication (PDIN/Y), and smart contract updating (all dynamic).

*Smart contract execution* measures the time required to run a smart contract. The process uses 2PD and writes the output to a zipped file on the delegatee’s machine.

#### Datasets

Each of the 16 mode combinations was evaluated by insertion and scaling costs. Insertion costs are determined by adding records en masse to a clean system instance (only contains the account request profile). Insights are garnered on cost amortization and limits associated with bulk and single record processing without existing record influences (eg, re-encryption and retransmission), which may direct policy on block synchronization. Moreover, 4 datasets of observations (1 per day) were synthetically generated for testing (Table 6). Each was statically sized at 400 bytes for record-level evaluation. As each record is of identical size, cryptographic processing time, cipher length, and bytes transmitted are comparable by record across the various configurations.

Scaling is scrutinized by gauging the effect existing records have on insertions. As this system accumulates records, these experiments facilitate the examination of existing medical records on overall performance. For insertion, care was taken to avoid interaction between new and existing data. Here, the reverse is of interest on how existing data affect record insertions. The results inform decisions on configuration selection and synchronization strategy. Testing began by instantiating a system with 1 of the 3 datasets (Table 6)—representative of small, medium, and large patient records from a deidentified instructional medical database [120]. Then, the 4 insertion datasets were applied, with the average per record reported as an indicator of general performance.

**Table 6:** Number of records and byte range per record by experimental dataset (all records were formatted as Health Level-7 Fast Healthcare Interoperability Resources [FHIR] JSON messages using HAPI FHIR).

Experiment	Records	Bytes/record (average)	Notes
Insertion	1	400	1 day
Insertion	30	400	30 days
Insertion	365	400	1 year
Insertion	1461	400	4 years
Scaling	334	395-761 (581)	26 encounters, 33 conditions, 145 medication requests, and 130 observations
Scaling	945	395-768 (675)	27 encounters, 159 conditions, 624 medication requests, and 135 observations
Scaling	2361	394-770 (732)	109 encounters, 119 conditions, 2029 medication requests, and 104 observations

#### Testing Environment

The testing environment aligns with the minimal requirements defined in the Experimental Design section. In its simplest form, HealthChain is a medium of information exchange between an entity (eg, patient) and a server (eg, node). Every process in HealthChain can be reduced to a series of entity-server interactions; therefore, our testing environment emulates this 2-machine structure.

The first machine is a Lenovo T540p running Windows 7 Enterprise with 16 GB of memory, an Intel i7-4800 MQ processor, and a wired, consumer internet connection. The second is a Dell Optiplex 9010 running Linux Mint 17.1 with 8 GB of memory, an Intel i7-3770 processor, and a wired, business internet connection. Communication rates (download/upload in megabits per second [Mbps]) are as follows: 32.1/5.9 and 955.4/176.3 Mbps for each respective machine [121].

To facilitate direct processing time comparisons between client and server, 1 machine (the Lenovo) assumed both roles. This colocation, however, failed to address networking concerns. Thus, experiments incorporating transmission costs (ie, client processing time and smart contract execution) were duplicated using both machines, which are situated 5 miles apart. These times replaced those in the 2 identified measures for a more realistic outcome while still affording client and server relative performance comparisons.

### Proof-of-Concept Implementation

The proof of concept facilitates the examination of the novel HealthChain elements as specified in section Experimental Design; it is not a production-ready blockchain system. The realization of the proposed framework requires the blending of the components defined herein with an existing blockchain technology such as Hyperledger Fabric or Ethereum. The proof of concept consists of 2 systems and 2 libraries written primarily in Java 8 and JSP and uses HAPI FHIR for document formatting [122] (Multimedia Appendix 3).

The first system, *HealthChainServer*, instantiates a single node that, for instance, establishes the blockchain, processes patient account requests, validates and manages patient identities and block transactions, supports 2PD, and transmits updates to patients (eg, the latest encounter). A multinode system (and therefore broadcasting capabilities) is unnecessary for testing. In addition, log blocks are not examined as they are a trivial extension to existing blockchain technologies.

The second system, *HealthChainWebClient*, is a simple JSP-enabled version of the Gentelella Alela template [123]. Through the Web portal, patients can, for example, request accounts, create and manage smart contracts, manually add records, import and export FHIR messages, and receive updates (eg, from a participating hospital). It was through the file upload feature that experimental data were added, which were then transmitted to the server over a socket connection. Although sufficient for testing, a robust, security-focused, application programming interface–driven approach (such as the one developed using SMART on FHIR [124]) should be implemented before use in production.

Regarding libraries, the first provides chameleon hash support by way of PCCH [50] as outlined in the section on Hashing Redactable Blocks. The second realizes PRE through AFGH [60] (using, as a foundation, the Java Pairing-Based Cryptography library [jPBC] [113]) as justified in the section on Block Encryption.

## Results

### Transmission Size

**Figure 12:**
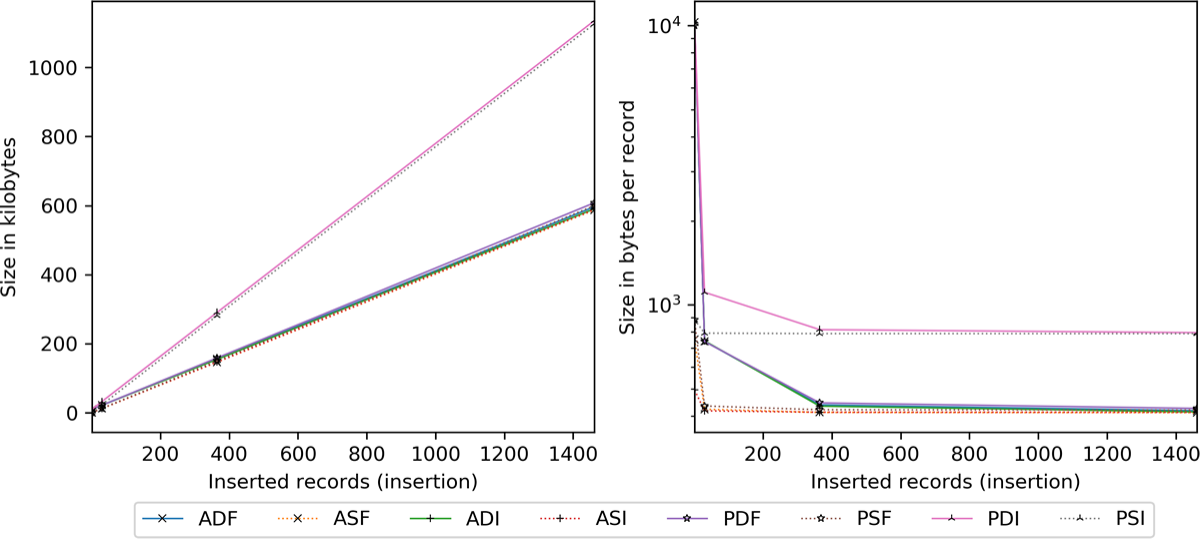
Transmission size in kilobytes and bytes per record by the number of records inserted. ADF: AES-encrypted data, dynamic keys, full block storage; ADI: AES-encrypted data, dynamic keys, incremental storage; AES: Advanced Encryption Standard; ASF: AES-encrypted data, static keys, full block storage; ASI: AES-encrypted data, static keys, incremental storage; PDF: PRE-encrypted data, dynamic keys, full block storage; PDI: PRE-encrypted data, dynamic keys, incremental storage; PRE: proxy re-encryption; PSF: PRE-encrypted data, static keys, full block storage; PSI: PRE-encrypted data, static keys, incremental storage.

**Figure 13:**
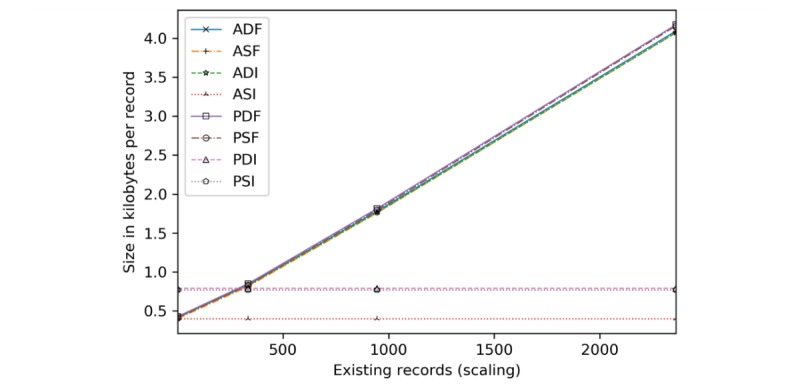
Transmission size in kilobytes per record added given an existing record set. ADF: AES-encrypted data, dynamic keys, full block storage; ADI: AES-encrypted data, dynamic keys, incremental storage; AES: Advanced Encryption Standard; ASF: AES-encrypted data, static keys, full block storage; ASI: AES-encrypted data, static keys, incremental storage; PDF: PRE-encrypted data, dynamic keys, full block storage; PDI: PRE-encrypted data, dynamic keys, incremental storage; PRE: proxy re-encryption; PSF: PRE-encrypted data, static keys, full block storage; PSI: PRE-encrypted data, static keys, incremental storage.

Here, transmission size is analyzed by insertion (Figure 12) and scaling (Figure 13). Server-side encryption does not impact transmission, hence its exclusion.

In Figure 12, PDI and PSI are nearly double the others (in overall and per record transmission size). Both have 789−400=389 bytes of padding (ie, wasted space) per record inserted, thus the disparity with all AES configurations. In addition, PDF and PSF are in line with AES (about 2% larger) rather than PDI and PSI. This is attributable to the full block generation process, as all records are fused into one, then divided into 789-byte ciphers, markedly reducing padding (see the section on Dataset Effects on Cipher Size). Furthermore, by 365, all ciphers near saturation (ie, negligible padding and amortized overhead).

Regarding overhead, dynamic options include 9137 bytes per smart contract (one here), ADI and ADF incorporate a new 397-byte AES PRE key, PDI uses a 384-byte scalar, and all send a 96-byte block hash.

To scaling (Figure 13), ASI, PDI, and PSI are unaffected by the number of existing records (only transmit modifications), requiring 416, 789, and 810 bytes on average of encrypted data per added record, respectively.

### Dataset Effects on Cipher Size

AES and PRE cipher sizes are dissected in Tables 7 and 8 for incremental and full block encryption modes, respectively. Comparisons are drawn at the record level; thus, overhead bytes were removed (see the section on Transmission Size and 231 bytes for the patient record generated during account activation).

AES is slightly larger on average than the underlying data (0.7%-4.1%) regardless of mode. PRE incremental is extensively padded (26.5%-97.3%), whereas minimal in full block mode (3.1%-5.2% beyond 1 insertion). Thus, PRE is subject to extreme variability, relative to AES, on input file size.

**Table 7:** Incremental storage: byte range per file and average Advanced Encryption Standard- and proxy re-encryption-encrypted cipher sizes by dataset (insertion and scaling).

Dataset	Bytes/file range (average)	AES^a^ average (difference, %)	PRE^b^ average (difference, %)
130,365, and 1461	400	416 (4.0)	789 (97.3)
334	395-761 (581)	585 (0.6)	802 (38.0)
945	395-768 (675)	679 (0.7)	840 (24.3)
2361	394-770 (732)	737 (0.7)	926 (26.5)

^a^AES: Advanced Encryption Standard.

^b^PRE: proxy re-encryption.

**Table 8:** Full block storage: total bytes per dataset and average Advanced Encryption Standard- and proxy re-encryption-encrypted cipher sizes by dataset (insertion and scaling).

Dataset	Total bytes	AES^a^ average (difference, %)	PRE^b^ average (difference, %)
1	400	416 (4.0)	789 (97.3)
30	12,000	12,368 (3.1)	12,624 (5.2)
365	146,000	150,112 (2.8)	153,066 (4.8)
1465	584,400	600,848 (2.8)	613,053 (4.9)
334	193,815	196,304 (1.3)	200,406 (3.4)
945	637,934	644,688 (1.1)	658,026 (3.1)
2361	1,727,714	1,744,032 (0.9)	1,779,981 (3.0)

^a^AES: Advanced Encryption Standard.

^b^PRE: proxy re-encryption.

### Network Latency

**Figure 14:**
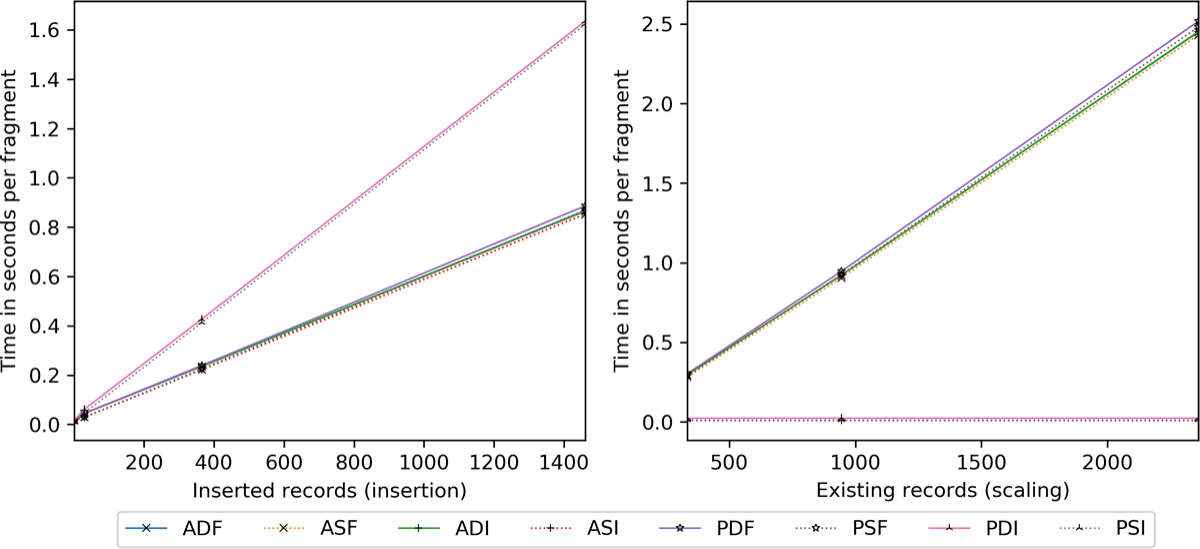
Client-to-server network latency in seconds per inserted record (ie, insertion) and seconds per record added given an existing record set (ie, scaling) — includes connection establishment, termination, and transmission time. ADF: AES-encrypted data, dynamic keys, full block storage; ADI: AES-encrypted data, dynamic keys, incremental storage; AES: Advanced Encryption Standard; ASF: AES-encrypted data, static keys, full block storage; ASI: AES-encrypted data, static keys, incremental storage; PDF: PRE-encrypted data, dynamic keys, full block storage; PDI: PRE-encrypted data, dynamic keys, incremental storage; PRE: proxy re-encryption; PSF: PRE-encrypted data, static keys, full block storage; PSI: PRE-encrypted data, static keys, incremental storage.

**Figure 15:**
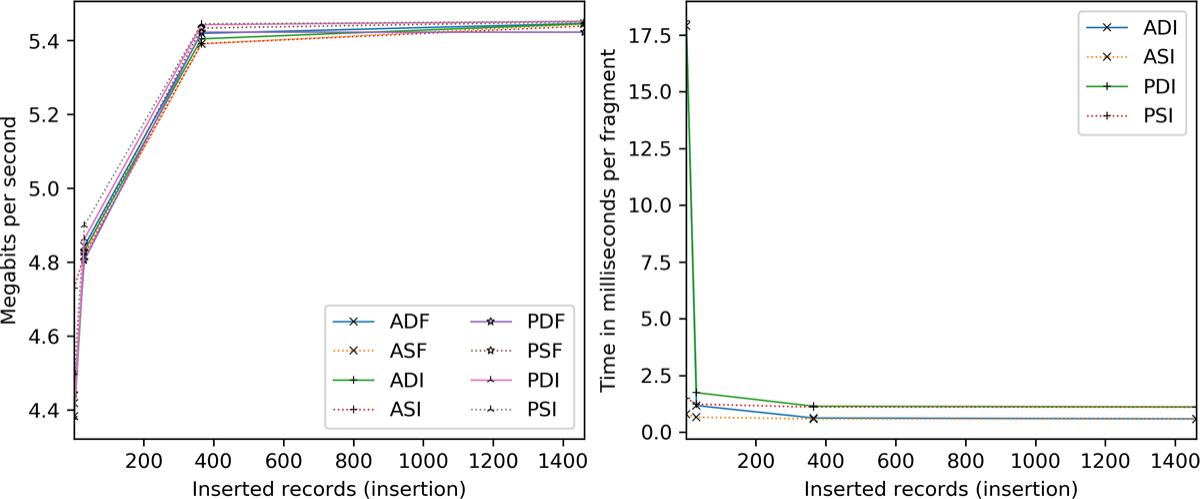
Client-to-server network latency (transmission only) measured in megabits per second and milliseconds per fragment by the number of records inserted. ADF: AES-encrypted data, dynamic keys, full block storage; ADI: AES-encrypted data, dynamic keys, incremental storage; AES: Advanced Encryption Standard; ASF: AES-encrypted data, static keys, full block storage; ASI: AES-encrypted data, static keys, incremental storage; PDF: PRE-encrypted data, dynamic keys, full block storage; PDI: PRE-encrypted data, dynamic keys, incremental storage; PRE: proxy re-encryption; PSF: PRE-encrypted data, static keys, full block storage; PSI: PRE-encrypted data, static keys, incremental storage.

**Figure 16:**
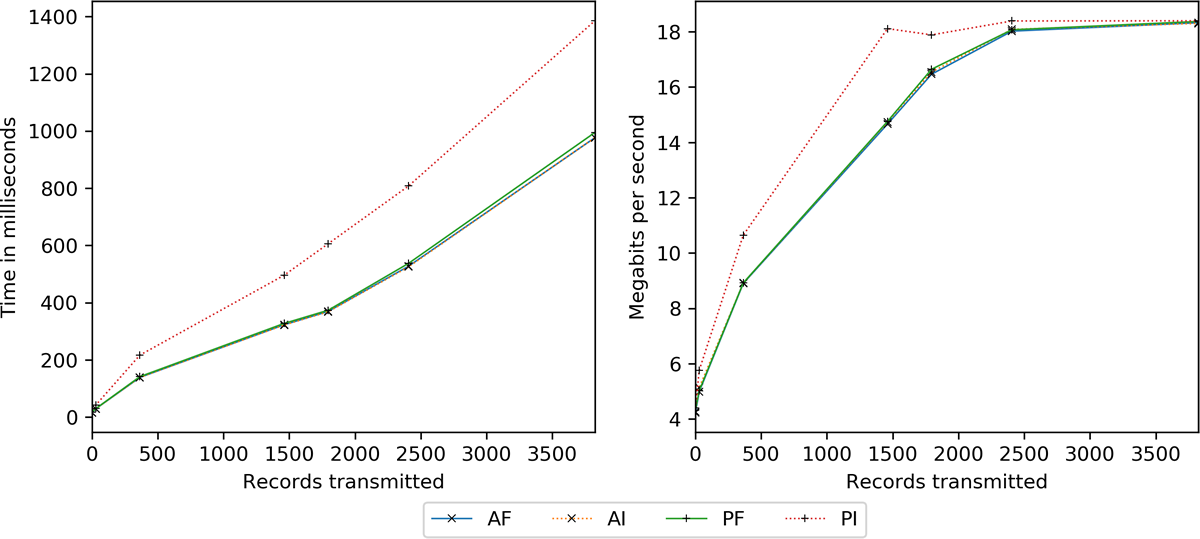
Server-to-client network latency (transmission only) measured in milliseconds and megabits per second by the number of records transmitted to the client. AES: Advanced Encryption Standard; AF: AES-encrypted data, full block storage; AI: AES-encrypted data, incremental block storage; PF: PRE-encrypted data, full block storage; PI: PRE-encrypted data, incremental storage; PRE: proxy re-encryption.

Network latency is analyzed in isolation to understand client-to-server (Figures 14 and 15) and server-to-client (Figure 16) effects. For insertion costs, Figure 14, incremental PRE grows at about twice the pace of others, proportional to cipher size (Tables 7 and 8). In addition, by 365 in Figure 15, the Mbps transmitted saturate the connection, whereupon ADI/ASI and PDI/PSI stabilize at 0.6 and 1.1 milliseconds per fragment (ie, a single record or full block), respectively. Regarding scaling (Figure 14), ASI, PDI, and PSI are constant, whereas full block and ADI grow as they reprocess all entries per update.

Figure 16 examines server-to-client transmissions as anticipated during record downloads from participating entities (eg, clinics) and intermediate smart contract results (if a delegatee). Data include the insertion sets as well as existing with 1461 additions to demonstrate scale. Transmission time depends on the block encryption (cipher size) and storage (padding effects) modes. AF, AI, and PF are indistinguishable from one another, whereas PI is on average 55% slower because of larger, excessively padded ciphers. Regarding bandwidth, a limit at approximately 18.4 Mbps (3.4 times the update limit in Figure 15), first experienced by the larger PI, is revealed. This corresponds to increased trajectories in time.

### Client Processing Time

**Figure 17:**
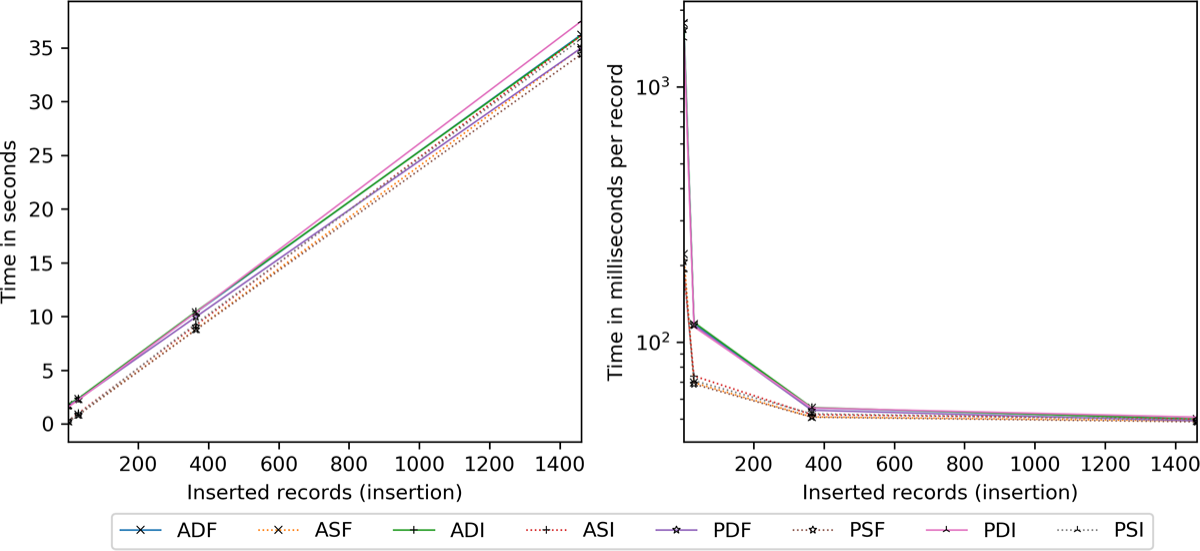
Client processing time in seconds and milliseconds per record by the number of records inserted. ADF: AES-encrypted data, dynamic keys, full block storage; ADI: AES-encrypted data, dynamic keys, incremental storage; AES: Advanced Encryption Standard; ASF: AES-encrypted data, static keys, full block storage; ASI: AES-encrypted data, static keys, incremental storage; PDF: PRE-encrypted data, dynamic keys, full block storage; PDI: PRE-encrypted data, dynamic keys, incremental storage; PRE: proxy re-encryption; PSF: PRE-encrypted data, static keys, full block storage; PSI: PRE-encrypted data, static keys, incremental storage.

**Figure 18:**
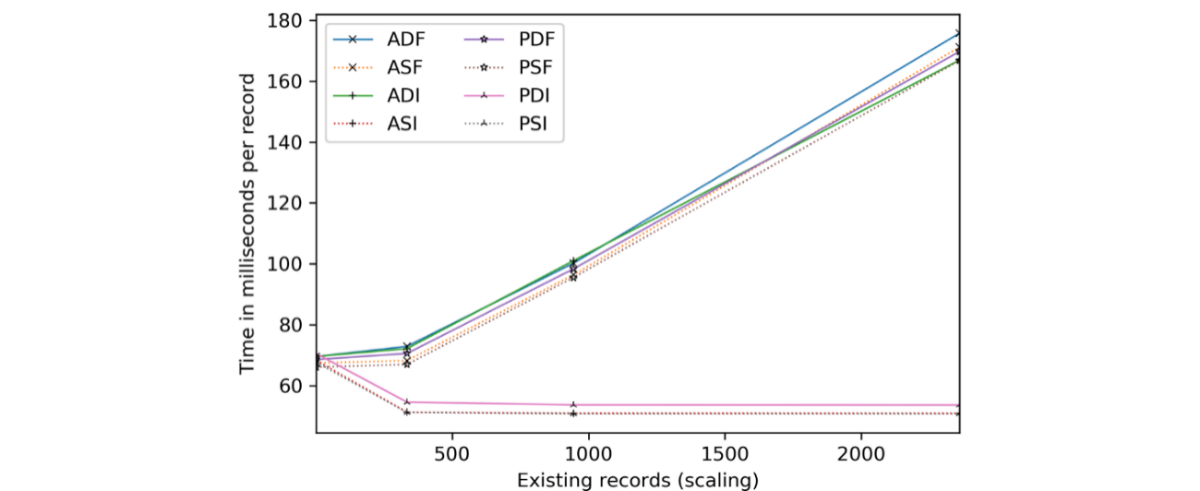
Client processing time in milliseconds per record added given an existing record set. ADF: AES-encrypted data, dynamic keys, full block storage; ADI: AES-encrypted data, dynamic keys, incremental storage; AES: Advanced Encryption Standard; ASF: AES-encrypted data, static keys, full block storage; ASI: AES-encrypted data, static keys, incremental storage; PDF: PRE-encrypted data, dynamic keys, full block storage; PDI: PRE-encrypted data, dynamic keys, incremental storage; PRE: proxy re-encryption; PSF: PRE-encrypted data, static keys, full block storage; PSI: PRE-encrypted data, static keys, incremental storage.

Client processing time is explored for insertions (Figure 17) and scaling (Figure 18). Server-side encryption does not impact client performance, hence its exclusion. For these tests, the client and server are the same machine (for relative comparison), whereas the transmission time is taken from the network latency experiments.

All configurations require a similar amount of time per Figure 17. The quickest are the static full block approaches, followed by dynamic full block (2% slower), static incremental (8% slower), and dynamic incremental (12% slower) approaches. Per record, by 365, all are within 5 milliseconds and narrowing.

Concerning scaling (Figure 18), network latency accounts for 24% to 27% of the overall cost at 1 existing record, dropping precipitously to 1% to 4% by 365. Beyond 365, ASI, PDI, and PSI are constant time as only new and modified information are processed. Although the others are 4 to 9 milliseconds apart, ASF and PSF tend to be slightly faster. However, by 2362, ASI, PDI, and PSI are 68% to 71% more efficient than the other methods.

### Server Processing Time

**Figure 19:**
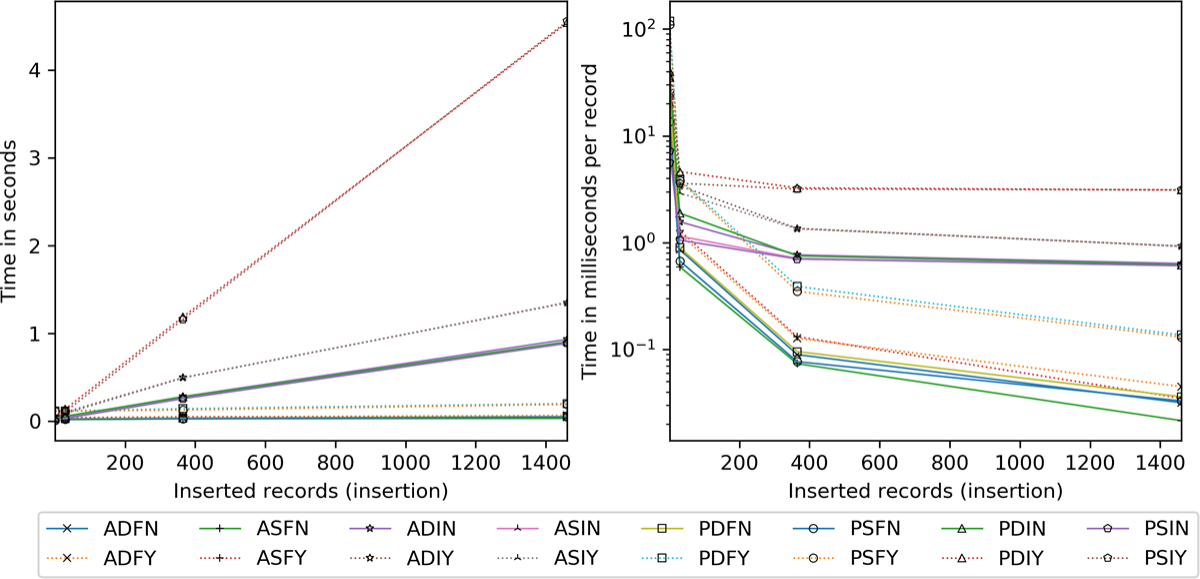
Server processing time in seconds and milliseconds per record by the number of records inserted. ADFN: AES-encrypted data, dynamic keys, full block storage, no server-side encryption; ADFY: AES-encrypted data, dynamic keys, full block storage, server-side encryption; ADIN: AES-encrypted data, dynamic keys, incremental storage, no server-side encryption; ADIY: AES-encrypted data, dynamic keys, incremental storage, server-side encryption; AES: Advanced Encryption Standard; ASFN: AES-encrypted data, static keys, full block storage, no server-side encryption; ASFY: AES-encrypted data, static keys, full block storage, server-side encryption; ASIN: AES-encrypted data, static keys, incremental storage, no server-side encryption; ASIY: AES-encrypted data, static keys, incremental storage, server-side encryption; PDFN: PRE-encrypted data, dynamic keys, full block storage, no server-side encryption; PDFY: PRE-encrypted data, dynamic keys, full block storage, server-side encryption; PDIN: PRE-encrypted data, dynamic keys, incremental storage, no server-side encryption; PDIY: PRE-encrypted data, dynamic keys, incremental storage, server-side encryption; PRE: proxy re-encryption; PSFN: PRE-encrypted data, static keys, full block storage, no server-side encryption; PSFY: PRE-encrypted data, static keys, full block storage, server-side encryption; PSIN: PRE-encrypted data, static keys, incremental storage, no server-side encryption; PSIY: PRE-encrypted data, static keys, incremental storage, server-side encryption.

Server insertion costs are presented in Figure 19. Full block approaches process insertions the fastest. The order is in tens of milliseconds for AES (19-65 milliseconds), and PRE without server-side encryption (14-53 milliseconds). PRE with server-side encryption is measured in the 111 to 199 milliseconds range. ASIN and PSIN extend nearly uniformly from 7 to 889 milliseconds over the sets, with a slight dynamic keying penalty of 12 to 42 milliseconds. ADIY and ASIY are roughly 40% to 70% costlier than ADIN and ASIN. PDIY and PSIY are the slowest, reaching 4.5 seconds at 1461 insertions. Per record, incremental methods level off at 365, whereas full block approaches continue to decline at a rate greater than 57% at 1461.

**Figure 20:**
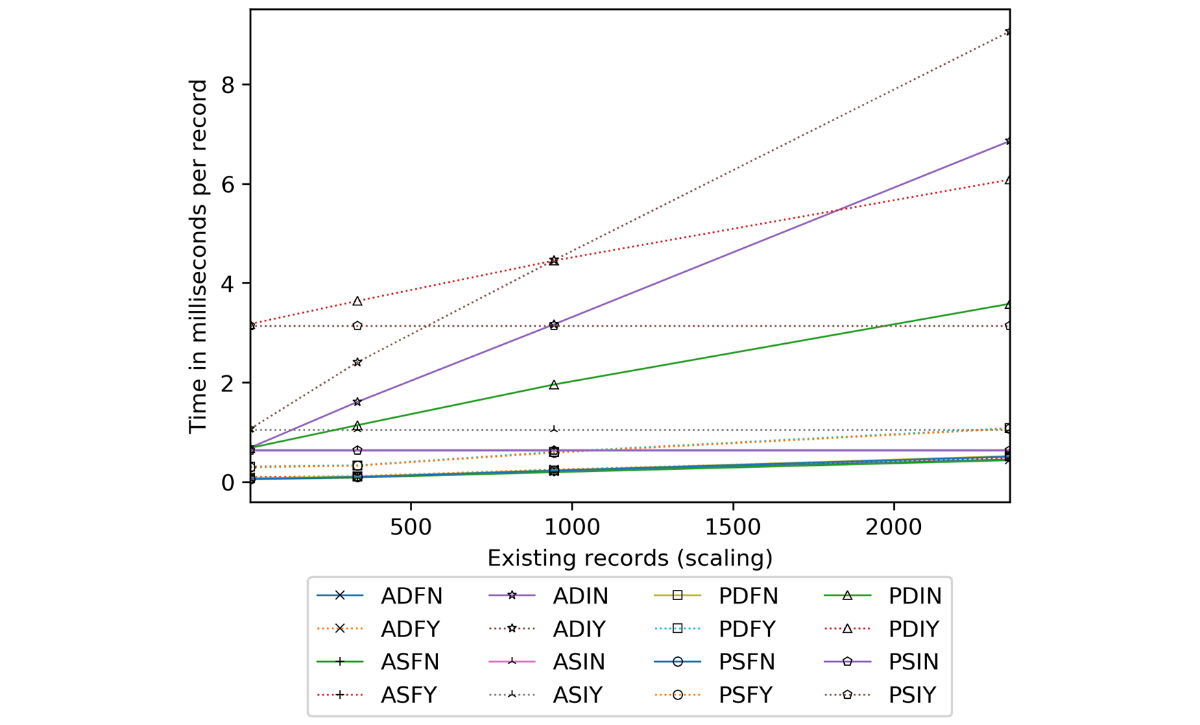
Server processing time in milliseconds per record added given an existing record set. ADFN: AES-encrypted data, dynamic keys, full block storage, no server-side encryption; ADFY: AES-encrypted data, dynamic keys, full block storage, server-side encryption; ADIN: AES-encrypted data, dynamic keys, incremental storage, no server-side encryption; ADIY: AES-encrypted data, dynamic keys, incremental storage, server-side encryption; AES: Advanced Encryption Standard; ASFN: AES-encrypted data, static keys, full block storage, no server-side encryption; ASFY: AES-encrypted data, static keys, full block storage, server-side encryption; ASIN: AES-encrypted data, static keys, incremental storage, no server-side encryption; ASIY: AES-encrypted data, static keys, incremental storage, server-side encryption; PDFN: PRE-encrypted data, dynamic keys, full block storage, no server-side encryption; PDFY: PRE-encrypted data, dynamic keys, full block storage, server-side encryption; PDIN: PRE-encrypted data, dynamic keys, incremental storage, no server-side encryption; PDIY: PRE-encrypted data, dynamic keys, incremental storage, server-side encryption; PRE: proxy re-encryption; PSFN: PRE-encrypted data, static keys, full block storage, no server-side encryption; PSFY: PRE-encrypted data, static keys, full block storage, server-side encryption; PSIN: PRE-encrypted data, static keys, incremental storage, no server-side encryption; PSIY: PRE-encrypted data, static keys, incremental storage, server-side encryption.

Regarding scaling (Figure 20), ASIN, ASIY, PSIN and PSIY are constant time. ASIN and PSIN take 0.6, ASIY 1.1, and PSIY 3.1 milliseconds per record. The rest are affected in various ways by existing records. The quickest configurations are ADFN, ADFY, ASFN, ASFY, PDFN, and PSFN. By 2362, they are just shy of ASIN and PSIN. PDFY and PSFY are minimally affected by existing records, with times ranging from 0.3 to 1.1 milliseconds. PDIN is next at roughly 0.7 to 3.6 milliseconds. ADIN and ADIY increase sharply from 0.7 to 6.9 milliseconds and 1.1 to 9.1 milliseconds, respectively. PDIY, at 3.5 to 6.1 milliseconds, is cheaper than ADIY at 945 and ADIN around 1800.

### Smart Contract Execution

**Figure 21:**
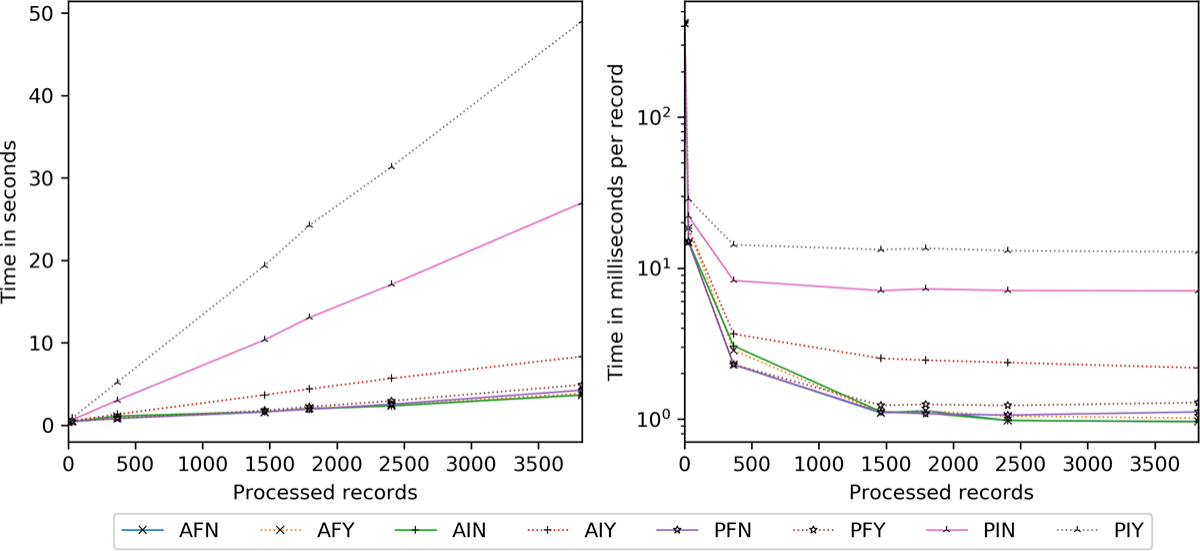
Smart contract execution time in seconds and milliseconds per record by the number of records processed. AES: Advanced Encryption Standard; AFY: AES-encrypted data, full block storage, server-side encryption; AFN: AES-encrypted data, full block storage, no server-side encryption; AIN: AES-encrypted data, incremental block storage, no server-side encryption; AIY: AES-encrypted data, incremental storage, server-side encryption; PFN: PRE-encrypted data, full block storage, no server-side encryption; PFY: PRE-encrypted data, full block storage, server-side encryption; PIN: PRE-encrypted data, incremental block storage, no server-side encryption; PIY: PRE-encrypted data, incremental storage, server-side encryption; PRE: proxy re-encryption.

Smart contract execution by time and per record is assessed in this section, with results conveyed in Figure 21. Encryption key mode is not a factor in smart contract execution, as there is only 1 re-encryption key for all data; thus, it is not reported. As with the server-to-client network latency experiments, data include insertion and existing with 1461 additions sets.

In absolute terms, AFN, AFY, and AIN are the fastest, followed by PFN and PFY (progressing from 1% to 20% slower), AIY (23%-183%), PIN (46%-854%), and PIY (94%-1679%). Incremental server-side encryption is considerably expensive, doubling PRE and tripling AES times. Network latency accounts for around 9% of incremental and 19% of full block time. Per record, AIY, PIN, and PIY noticeably level-off by 365, and AFY, PFN, and PFY by 1461. AFN and AIN, however, descend beyond 3822 (2361+1461) at 2%.

## Discussion

### Principal Findings

**Figure 22:**
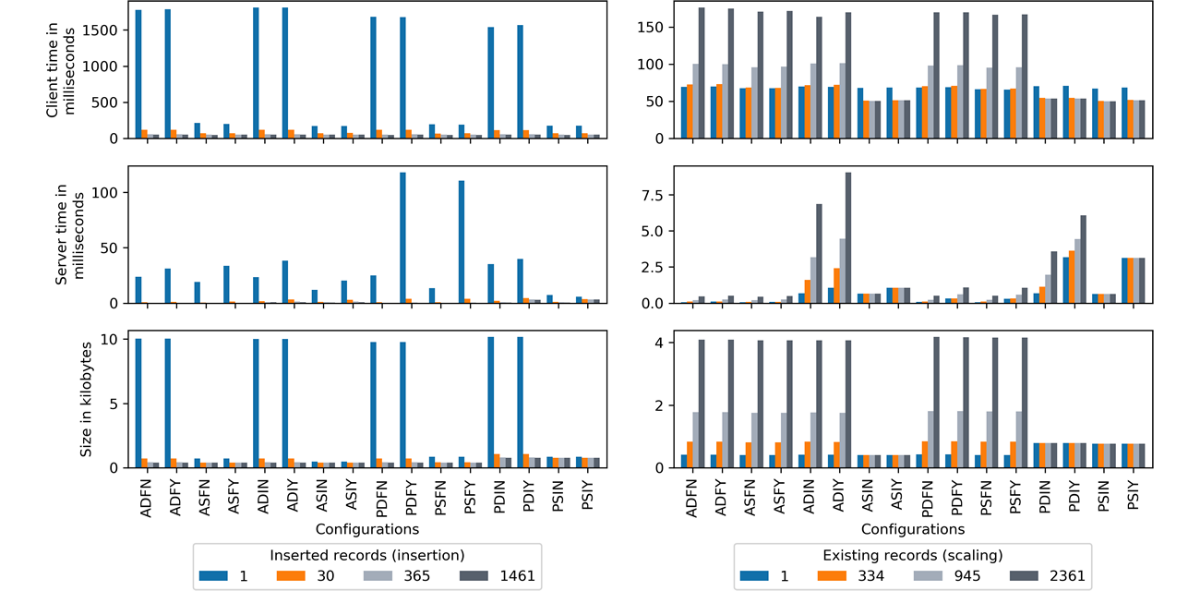
Relative comparison of client and server processing time in milliseconds and transmission size in kilobytes per record by insertion and scaling. ADFN: AES-encrypted data, dynamic keys, full block storage, no server-side encryption; ADFY: AES-encrypted data, dynamic keys, full block storage, server-side encryption; ADIN: AES-encrypted data, dynamic keys, incremental storage, no server-side encryption; ADIY: AES-encrypted data, dynamic keys, incremental storage, server-side encryption; AES: Advanced Encryption Standard; ASFN: AES-encrypted data, static keys, full block storage, no server-side encryption; ASFY: AES-encrypted data, static keys, full block storage, server-side encryption; ASIN: AES-encrypted data, static keys, incremental storage, no server-side encryption; ASIY: AES-encrypted data, static keys, incremental storage, server-side encryption; PDFN: PRE-encrypted data, dynamic keys, full block storage, no server-side encryption; PDFY: PRE-encrypted data, dynamic keys, full block storage, server-side encryption; PDIN: PRE-encrypted data, dynamic keys, incremental storage, no server-side encryption; PDIY: PRE-encrypted data, dynamic keys, incremental storage, server-side encryption; PRE: proxy re-encryption; PSFN: PRE-encrypted data, static keys, full block storage, no server-side encryption; PSFY: PRE-encrypted data, static keys, full block storage, server-side encryption; PSIN: PRE-encrypted data, static keys, incremental storage, no server-side encryption; PSIY: PRE-encrypted data, static keys, incremental storage, server-side encryption.

Figure 22 integrates pertinent results into a single visual for high-level performance analysis of client and server insertion (per record insertion cost given *n* loaded records) and scaling (per record insertion cost given *n* existing records) operations. Proceeding from top to bottom is client time in milliseconds (ie, section on Client Processing Time), server time in milliseconds (ie, section on Server Processing Time), and size in kilobytes (ie, section on Transmission Size).

The impracticality of full block approaches is apparent from the patient’s vantage point. Whether inserting or scaling, block formation and transmission are resource intensive. For those with basic computers or mobile devices, or those operating on metered or low-bandwidth networks, these options should be avoided.

Catering to constrained environments are ASIN, PSIY, PDIN, PDIY, PSIN, and PSIY. ASIN, ASIY, PSIN, and PSIY are constant in bytes and server processing time, with decreasing client processing time because of network latency amortization over an otherwise constant process. PDIN and PDIY are constant in bytes and amortize latency as do the previous but irregular for the server (ie, server-side rekeying). Overall, ASIN and ASIY are the top performers. They require minimal time and bandwidth for record insertion, hold constant when scaling, and quickly execute smart contracts (refer to the section on Smart Contract Execution for details). The compromise is security. Static approaches, although fast, are susceptible to attacks (see the section on Smart Contracts and Multimedia Appendix 1). Dynamic selections are more secure as data are continually rekeyed, preventing decryption by old or compromised keys. ADIN and ADIY are arguably the worst and operationally infeasible, as rekeying requires (1) the client to decrypt, encrypt, and transmit all information with each update and (2) the server to replace the existing block with the new, server-side–encrypted data.

PRE incremental methods are byte intensive because of excessive padding of the small experimental files and slow during smart contract execution. This is mostly mitigated through full block approaches. Unlike AES, the variability of PRE cipher size is vast. It has the potential to be compact and efficient or bloated and wasteful. PSIN and PSIY are subject to the same static key vulnerabilities as ASIN and ASIY and are more expensive. One must decide if cipher malleability justifies increased resource expenditure.

PDIN and PDIY are the only viable dynamic options. Server processing is insignificant for record insertions (a few milliseconds) but rises with scale. From 1 to 2361, PDIN is 1% to 7% and PDIY 4% to 11%, the magnitude of the client. Server processing is projected to eclipse client by around 40,000 and 37,000 records for PDIN and PDIY respectively. However, with appropriate hardware and in-memory databases, this cost can be reduced. Compared with ASIN and ASIY, both are marginally slower on the client, but roughly twice in bytes and latency.

Ultimately, several candidates emerge. ASIN and ASIY for speed, PSIN and PSIY for malleability, and PDIN and PDIY for malleability and security. Refer to Table 9 for a detailed comparison.

**Table 9:** Comparison of practical configurations by cipher malleability; security; insertion, scaling, and smart contract execution time; and transmitted bytes.

Property	ASIN^a^	ASIY^b^	PSIN^c^	PSIY^d^	PDIN^e^	PDIY^f^
Cipher malleability	No	No	Yes	Yes	Yes	Yes
Security—dynamic keying	No	No	No	No	Yes	Yes
Security—server-side encryption	No	Yes	No	Yes	No	Yes
Client insertions time	Fastest	Fastest	Fast	Fast	Fast if >30	Fast if >30
Client insertions bytes	Smallest	Smallest	Largest	Largest	Largest	Largest
Server insertions time	Fast	Slow	Slowest	Slow	Fast	Slowest
Client scaling time	Fastest, constant^g^	Fastest, constant^g^	Fastest, constant^g^	Fastest, constant^g^	Fast, constant^g^	Fast, constant^g^
Client scaling bytes	Smallest, constant	Smallest, constant	Small, constant	Small, constant	Small, constant	Small, constant
Server scaling time	Fast, constant	Slow, constant	Fast, constant	Slower, constant	Slower	Very slow
Smart contract execution	Fastest	Fastest	Very slow	Very slow	Slowest	Slowest

^a^ASIN: Advanced Encryption Standard–encrypted data, static keys, incremental storage, no server-side encryption.

^b^ASIY: Advanced Encryption Standard–encrypted data, static keys, incremental storage, server-side encryption.

^c^PSIN: proxy re-encryption–encrypted data, static keys, incremental storage, no server-side encryption.

^d^PSIY: proxy re-encryption–encrypted data, static keys, incremental storage, server-side encryption.

^e^PDIN: proxy re-encryption–encrypted data, dynamic keys, incremental storage, no server-side encryption.

^f^PDIY: proxy re-encryption–encrypted data, dynamic keys, incremental storage, server-side encryption.

^g^Constant time if latency, which is amortized over records, is not factored.

### Limitations

Our study has the following limitations. First, the results are consistent with AES-128 and EC-256 alone. It is impossible to extrapolate the effects a change may have. Second, the small experimental files resulted in excess PRE cipher padding. Although the records were legitimate, EHR data may produce different results. Third, only 1 smart contract, which dynamic options regenerate during an update, was present for testing. With many contracts and few existing records, overall performance may diminish. In addition, smart contract regeneration was not optimized as the entire contract was reproduced and transmitted instead of just the re-encryption keys. This modification has the potential to decrease the size by 41%. Finally, server-side encryption only operates in dynamic mode. A static or periodic (eg, daily or after *x* number of transactions) option would reduce server-side processing at the expense of security. This will especially benefit PRE schemes, as they suffer a tremendous penalty under the weight of rekeying data after each read.

### Conclusions and Future Work

In this study, a proof-of-concept patient-centered blockchain—HealthChain—was presented. The posited framework promotes patient engagement and facilitates secure, mediated information exchange between patients and providers. Redactable patient blocks, by way of chameleon hashing, were introduced to minimize data fragmentation, allow for in-place editing, and reduce resource consumption. PRE, smart contracts, and HL7 FHIR form the foundation of our proposed information exchange model, along with our 2PD PRE scheme and signature methods. A total of 16 experimental configurations were examined over 5 system dimensions by the cost of record insertion and scaling. Results indicate ASIN was the fastest and least bandwidth intensive, whereas PDIY was the best cryptographically, although the ultimate configuration rests with implementers and their desired level of speed and security.

Furthermore, 3 areas are targeted for future work. First, Barreto-Lynn-Scott [125] and Kachisa-Schaefer-Scott [126] EC families will be explored as potential replacements for the outdated jPBC curves. Second, as the proof-of-concept client tool is a deployed Web service on a client’s machine (sufficient for testing), practical application necessitates an architectural redesign, wherein clients access HealthChain through a hosted, browser-based system. Hence, the cryptographic services will be ported to JavaScript for client-side execution, ensuring plaintext and generated secrets remain unexposed to nodes. Finally, we integrate our solution into Hyperledger Fabric to make use of their consensus, permissioning, and communications infrastructure.

## Appendix

Multimedia Appendix 1 Proxy re-encryption mathematical foundation, proposed extensions, and proofs.

Multimedia Appendix 2 Experimental configurations.

Multimedia Appendix 3 Source code files.

## Abbreviations

2PD: 2-party proxy re-encryption decryption
AES: Advanced Encryption Standard
ADFN: AES-encrypted data, dynamic keys, full block storage, no server-side encryption
ADI: AES-encrypted data, dynamic keys, incremental storage
ADIN: AES-encrypted data, dynamic keys, incremental storage, no server-side encryption
ADIY: AES-encrypted data, dynamic keys, incremental storage, server-side encryption
AF: AES-encrypted data, full block storage
AFN: AES-encrypted data, full block storage, no server-side encryption
AIN: AES-encrypted data, incremental block storage, no server-side encryption
AIY: AES-encrypted data, incremental storage, server-side encryption
ASI: AES-encrypted data, static keys, incremental storage
ASIN: AES-encrypted data, static keys, incremental storage, no server-side encryption
ASIY: AES-encrypted data, static keys, incremental storage, server-side encryption
CFR: Code of Federal Regulations
EC: elliptic curves
ECC: elliptic curve cryptography
EHR: electronic health record
FHIR: Fast Healthcare Interoperability Resources
FIPS: Federal Information Processing Standard
HIPAA: Health Insurance Portability and Accountability Act of 1996
jPBC: Java Pairing-Based Cryptography library
Mbps: megabits per second
ONC: The Office of the National Coordinator for Health Information Technology
PCCH: Public-coin chameleon hash
PDF: PRE-encrypted data, dynamic encryption key full block storage
PDI: PRE-encrypted data, dynamic keys, incremental storage
PDIN: PRE-encrypted data, dynamic keys, incremental storage, no server-side encryption
PDIY: PRE-encrypted data, dynamic keys, incremental storage, server-side encryption
PF: PRE-encrypted data, full block storage
PFN: PRE-encrypted data, full block storage, no server-side encryption
PHR: personal health record
PI: PRE-encrypted data, incremental storage
PIN: PRE-encrypted data, incremental block storage, no server-side encryption
PIY: PRE-encrypted data, incremental storage, server-side encryption
PRE: proxy re-encryption
PSF: PRE-encrypted data, static keys, full block storage
PSI: PRE-encrypted data, static keys, incremental storage
PSIN: PRE-encrypted data, static keys, incremental storage, no server-side encryption
PSIY: PRE-encrypted data, static keys, incremental storage, server-side encryption
SHA: Secure Hash Algorithm
