# Supplementary material for: A Blockchain Framework for Patient-Centered Health Records and Exchange (HealthChain): Evaluation and Proof-of-Concept Study
Source: J Med Internet Res. 2019 Aug 31;21(8):e13592. doi: 10.2196/13592 (PMC6743266; doi:10.2196/13592)
Supplement: Multimedia Appendix 1 [file jmir_v21i8e13592_app1.pdf]

Multimedia Appendix 1

A – Introduction and Background

A1 – Approaches to Two-Party Decryption using PRE

In this article, smart contracts allow delegator $A \in \mathcal{U}$ to delegate controlled access to its encrypted data to delegatee $B \in \mathcal{U}$ by way of an enforcing, semi-trusted intermediary $R \in \mathcal{R}$ (e.g., nodes), where $\mathcal{U}$ and $\mathcal{R}$ are the set of all users and intermediaries respectively. The proxy re-encryption (PRE) scheme used herein – Ateniese et al.'s improved, second attempt [1] – can facilitate this function through a series of repeated encryption and decryption processes. However, this scheme, as well as others published, does not allow for secure two-party decryption. The following section illustrates various implementation possibilities and potential threats; thus, supporting the requirement of a new approach.

A1.1 – PRE Two-Party Decryption Scenarios

One implementation might be for R to decrypt the ciphertext c_A from A using the re-encryption key $rk_{A\to R}$, encrypt the resulting plaintext message m_A using B's public key pk_B, and transmit c_B to B. However, this requires full confidence in R as it now is in possession of m_A. In response, one can alter $rk_{A\to R}$ to $rk_{A\to B}$, necessitating the secret key of B (sk_B) to decrypt the message (secret is equivalent to private, but allows shorthand distinct as sk). This can be implemented in two ways; both with security concerns.

The first eliminates exposure of m_A to R by using R as a verification system that forwards ciphertexts and pk_B-encrypted re-encryption keys to B for decryption as valid under smart contract provisions. The issue is, once B has $rk_{A\to B}$, it can decrypt any c_A (this key does *not* support encryption) without verification. Meaning, B can decrypt, with impunity, blocks owned by A after, for instance, smart contract termination. Accounting for this, the second approach eliminates re-encryption key exposure to B by requiring sk_B be sent to R who facilitates decryption. However, R can now decrypt any c_A by way of $rk_{A\to B}$, decrypt and forge any c_B using sk_B, and masquerade as B in the system (e.g., sign contracts as B).

Clearly, two challenges persist. One, how to prevent R from gaining access to any message $m_{\mathcal{U}}$, and two, how to ensure B never possesses a re-encryption key $rk_{\mathcal{U}\to B}$. The remainder of this section provides an essentials-only overview of the PRE scheme used as well as our extensions to address the aforementioned concerns.

A2 – Improved Second Attempt PRE Scheme – Background

The modified PRE scheme is Ateniese et al.'s improved "Second Attempt" [1]. In this section we introduce relevant components and definitions, and refer the reader to Ateniese et al. [1] for complete details.

It is said that $e : G_1 \times \hat{G}_1 \to G_2$ is a bilinear map if (1) $G_1, \hat{G}_1$ are groups of the same prime order q; (2) for all $a, b \in \mathbb{Z}_q$ ($\mathbb{Z}_q$ is the set of all integers mod q), $g \in G_1$, and $h \in \hat{G}_1$, then

$e(g^a, h^b) = e(g,h)^{ab}$ is efficiently computable; and (3) the map is nondegenerate (i.e., if g generates G_1 and h generates $\hat{G}_1$, then $e(g,h)$ generates G_2).

The scheme itself is based on BBS [2] and ElGamal [3], operating over two groups G_1, G_2 of prime order q with a defined bilinear map $e : G_1 \times G_1 \rightarrow G_2$ (here, $\hat{G}_1 = G_1$). The system parameters are random generators $g \in G_1$ and $Z = e(g,g) \in G_2$.

The public/secret key pair for user A is in the form $pk_A = g^a$ and $sk_A = a$ respectively, where a is randomly selected from $\mathbb{Z}_q$. The relevant enciphering of message m_A is defined as the pairing $\varepsilon_A = (g^{ak_A}, c_A)$, where k_A is randomly selected from $\mathbb{Z}_q$ such that $k_A \neq a$, g^{ak_A} is the public cipher parameter, and $c_A = m_A Z^{k_A}$ is the encrypted message from A. Decrypting ε_A can be accomplished in several ways, two of which are examined. First, A can use sk_A in the following manner: $\frac{\varepsilon_A^1}{e(\varepsilon_A^0, g)^{-sk_A}} = \frac{c_A}{e(g^{ak_A}, g)^{-a}} = \frac{m_A Z^{k_A}}{e(g,g)^{ak_A/a}} = \frac{m_A Z^{k_A}}{Z^{k_A}} = m_A$. The second approach relies on the re-encryption key $rk_{A\rightarrow B} = g^{b/a}$, where A delegates decryption rights to delegatee B. A computes $rk_{A\rightarrow B} = pk_B^{-sk_A} = (g^b)^{-a} = g^{b/a}$. Decryption by B is as follows: $\frac{\varepsilon_A^1}{e(\varepsilon_A^0, rk_{A\rightarrow B})^{-sk_B}} = \frac{c_A}{e(g^{ak_A}, g^{b/a})^{-b}} = \frac{m_A Z^{k_A}}{e(g,g)^{ak_A b/ab}} = \frac{m_A Z^{k_A}}{Z^{k_A}} = m_A$.

B – Two-Party PRE Decryption Scheme

In this section, we submit a two-party PRE decryption scheme, which modifies the algorithms expressed in Section A2 to address the concerns raised in Section A1.1. This approach masks m_A to R and prevents B from learning $rk_{A\rightarrow B}$, and is available in *full* and *fragmented-block* configurations. The process is ordered, requiring R to complete its intermediate re-encryption prior to B's decryption.

B1 – Full-Block Configuration

Full-block configuration is an implementation option where block data are stored in a single container. All operations performed are done so over its entire contents. The following section describes the submitted PRE extensions and definitions necessary to achieve full-block two-party PRE.

To begin, the temporal nature of k_A is revised from fixed to dynamic. Each time a message is encrypted, k_A is randomly chosen – i.e., k_A^i for temporal index $i \in \mathcal{T}$. The next step is for A to compute $c_A^i = m_A^i Z^{k_A^i}$ as previously defined. A then calculates three parameters. The first is the public cipher parameter $pk_R^{sk_A l_A} = g^{ral_A}$. The second is the intermediate (R) re-encryption key $rk_{A\rightarrow RB}^i = \frac{e\left(g^{k_A^i}, pk_R\right)}{e\left(g^{k_A^i}, g\right)} = \frac{e\left(g^{k_A^i}, g^r\right)}{e\left(g^{k_A^i}, g\right)} = \frac{e(g,g)^{k_A^i r}}{e(g,g)^{k_A^i}} = \frac{Z^{k_A^i r}}{Z^{k_A^i}} = Z^{k_A^i (r-1)}$. The final parameter is the delegatee re-encryption key $rk_{AR\rightarrow B}^i = pk_B^{k_A^i / sk_A l_A} = g^{bk_A^i / al_A}$, where l_A is randomly selected from $\mathbb{Z}_q$ such that $l_A \neq a, k_A^i$. The addition of k_A^i to $rk_{AR\rightarrow B}^i$ binds the key temporally to c_A^i, eliminating the possibility of using the key to attack future or former

version of the ciphertext (refer to Propositions 1 and 2) and minimizing transitivity threats (Propositions 3 and 4).

Now that the keys have been established, R applies $rk^i_{A\to RB}$ to c^i_A as a simple scalar, resulting in the re-encrypted cipher $c^i_{A\to R} = m^i_A Z^{k^i_A r}$ (refer to Proposition 5). Thus, m^i_A remains hidden from R. Delegatee B recovers m^i_A through a normal decryption-by-proxy process where $\varepsilon^i_{A\to R} = \left(g^{ral_A}, c^i_{A\to R}\right)$ and the re-encryption key is $rk^i_{AR\to B} = g^{bk^i_A/al_A}$ (refer to Proposition 6). As the public cipher parameter contains r, B cannot compute $Z^{k^i_A}$, thus preventing it from directly decrypting c^i_A. Only the intermediate cipher $c^i_{A\to R}$ can be decrypted, ensuring R partakes in the transaction. Of note, if B has access to c^i_A, then after the first re-encryption, it is possible for B to deduce $rk^i_{A\to RB}$ (refer to Proposition 7); bypassing the intermediary in subsequent re-encryption events on c^i_A.

While B cannot use this deduced re-encryption key to decrypt any c^j_A where $j \neq i$, to ensure complete security, we propose an additional encryption layer imposed by R, one in which it encrypts and stores c^i_A with an ephemeral key x^i_R ($c^i_A Z^{x^i_R} = c^i_{AR}$). The intermediate and delegatee phases of two-party PRE must be slightly augmented as done in Propositions 8 and 9. As $Z^{x^i_R}$ is a simple scalar, it is still trivial to compute $rk^i_{A\to RB}$ as shown in Proposition 10. However, if R re-encrypts c^i_{AR} under a new x^j_R after access, this no longer poses a threat (see Proposition 11). This can be augmented to reduce the number of operations performed by the delegatee, who may be operating in a limited or constrained environment, without sacrificing security. The approach is quite intuitive and shown in Proposition 12. The ephemeral layer $Z^{x^i_R}$ is removed by R prior to re-encryption, then updated to x^j_R. This returns the two-party PRE process to the simplified version presented in Propositions 5 and 6, while holding to the security of Proposition 11. Of note: it is still possible for the encrypted value to change without the ephemeral key if A re-encrypts the data under a dynamic encryption key mode. However, this requires A's participation, which cannot be guaranteed. Conversely, R can perform this action on demand or periodically without access to avoid "stale" keys or if it suspects any x^*_R may have been compromised.

Proposition 1. For temporal index $i \in \mathcal{T}$, if ε^i_A consisting of $g^{ak^i_A}$ and c^i_A are public, B can decrypt any c^i_A upon learning $rk_{A\to B}$.

Proof. Let $\varepsilon^i_A = \left(g^{ak^i_A}, c^i_A\right)$, $c^i_A = m^i_A Z^{k^i_A}$, $rk_{A\to B} = g^{b/a}$, and $sk_B = b$. Thus:

$$\frac{\varepsilon^{i,1}_A}{e\left(\varepsilon^{i,0}_A, rk_{A\to B}\right)^{-sk_B}} = \frac{c^i_A}{e\left(g^{ak^i_A}, g^{b/a}\right)^{-b}} = \frac{m^i_A Z^{k^i_A}}{e(g,g)^{ak^i_A b/ab}} = \frac{m^i_A Z^{k^i_A}}{Z^{k^i_A}} = m^i_A \ \forall\ i \in \mathcal{T}$$

As $Z^{k_A^i}$ can be computed using $e\left(g^{k_A^i}, g\right)$, $g^{ak_A^i}$ requires only an inverse of a to satisfy the same equation. This is fulfilled by $g^{b/a}$, which is not bound to i. Thus, receipt of $rk_{A\to B}$ once grants permanent decryption rights to B regardless of the temporal index. ■

Proposition 2. Binding $rk_{A\to B}$ to i (i.e., $rk_{A\to B}^i$) addresses the problem identified in Proposition 1; namely that $rk_{A\to B}$ can decrypt any ciphertext regardless of temporality.

Proof. Let $\varepsilon_A^i = \left(g^{al_A}, c_A^i\right)$ where l_A is randomly selected from $\mathbb{Z}_q$ such that $l_A \neq a, k_A^i$; $c_A^i = m_A^i Z^{k_A^i}$; $rk_{A\to B}^i = g^{bk_A^i/al_A}$; and $sk_B = b$. Thus:

$$\frac{\varepsilon_A^{i,1}}{e\left(\varepsilon_A^{i,0}, rk_{A\to B}^i\right)^{-sk_B}} = \frac{c_A^i}{e\left(g^{al_A}, g^{bk_A^i/al_A}\right)^{-b}} = \frac{m_A^i Z^{k_A^i}}{e(g,g)^{al_A bk_A^i/al_A b}} = \frac{m_A^i Z^{k_A^i}}{Z^{k_A^i}} = m_A^i$$

shows $rk_{A\to B}^i$ still decrypts at temporal index i. The following proves this no longer holds when $j \neq i$:

$$\frac{\varepsilon_A^{j,1}}{e\left(\varepsilon_A^{j,0}, rk_{A\to B}^i\right)^{-sk_B}} = \frac{c_A^j}{e\left(g^{al_A}, g^{bk_A^i/al_A}\right)^{-b}} = \frac{m_A^j Z^{k_A^j}}{e(g,g)^{al_A bk_A^i/al_A b}} = \frac{m_A^j Z^{k_A^j}}{Z^{k_A^i}}$$
$$= m_A^j Z^{k_A^j - k_A^i} \neq m_A^j$$
■

Proposition 3. The re-encryption key $rk_{A\to B}$ is transitive if B colludes with C.

Proof. Let $sk_B = b$, $sk_C = c$, $rk_{A\to B} = g^{b/a}$, and $rk_{A\to C} = g^{c/a}$. Thus:

$$((rk_{A\to B})^{-sk_B})^{sk_C} = \left(\left(g^{b/a}\right)^{-b}\right)^c = \left(g^{b/ab}\right)^c = (g^{-a})^c = g^{c/a} = rk_{A\to C}$$

This can be done in a manner protective of sk_C by B computing and sending to C g^{-a}, who then calculates $g^{c/a}$. ■

Proposition 4. Binding $rk_{A\to B}$ to temporal index $i \in \mathcal{T}$ (Proposition 2) does not prevent re-encryption key transitivity from B to C, but renders it mute as access is not granted beyond what could have been given by B.

Proof. Let $sk_B = b$, $sk_C = c$, $rk_{A\to B}^i = g^{bk_A^i/a}$, and $rk_{A\to C}^i = g^{ck_A^i/a}$. Thus:

$$\left(\left(rk_{A\to B}^i\right)^{-sk_B}\right)^{sk_C} = \left(\left(g^{bk_A^i/a}\right)^{-b}\right)^c = \left(g^{bk_A^i/ab}\right)^c = \left(g^{k_A^i/a}\right)^c = g^{ck_A^i/a} = rk_{A\to C}^i$$

Though C is capable of decrypting c_A^i using $rk_{A\to C}^i$, it cannot do so with c_A^j where $j \neq i$ (Proposition 2). Thus, this is no better than B simply giving C m_A^i, as no privileges are extended to C beyond what could be shared directly. ■

Proposition 5. The result of the intermediate re-encryption of c_A^i given $rk_{A\to RB}^i$ is $c_{A\to R}^i = m_A^i Z^{k_A^i r} \neq c_A^i, m_A^i$.

Proof. Let $c_A^i = m_A^i Z^{k_A^i}$ and $rk_{A\to RB}^i = Z^{k_A^i(r-1)}$. Thus:

$$\begin{aligned} c_{A\to R}^i &= c_A^i\left(rk_{A\to RB}^i\right) = m_A^i Z^{k_A^i}\left(Z^{k_A^i(r-1)}\right) = m_A^i Z^{k_A^i}\left(Z^{k_A^i r - k_A^i}\right) = m_A^i Z^{k_A^i + k_A^i r - k_A^i} \\ &= m_A^i Z^{k_A^i r} \neq c_A^i, m_A^i \end{aligned}$$ ■

Proposition 6. The result of delegatee decryption on $c_{A\to R}^i$ given $\varepsilon_{A\to R}^i$, $rk_{AR\to B}^i$, and sk_B is m_A^i.

Proof. Let $\varepsilon_{A\to R}^i = \left(g^{ral_A}, c_{A\to R}^i\right)$, $c_{A\to R}^i = m_A^i Z^{k_A^i r}$, $rk_{AR\to B}^i = g^{bk_A^i/al_A}$, and $sk_B = b$. Thus:

$$\frac{\varepsilon_{A\to R}^{i,1}}{e\left(\varepsilon_{A\to R}^{i,0}, rk_{AR\to B}^i\right)^{-sk_B}} = \frac{c_{A\to R}^i}{e\left(g^{ral_A}, g^{bk_A^i/al_A}\right)^{-b}} = \frac{m_A^i Z^{k_A^i r}}{e(g,g)^{ral_A bk_A^i/al_A b}} = \frac{m_A^i Z^{k_A^i r}}{Z^{rk_A^i}} = m_A^i$$ ■

Proposition 7. Using c_A^i and $c_{A\to R}^i$, B can infer $rk_{A\to RB}^i$.

Proof. Let $c_A^i = m_A^i Z^{k_A^i}$, $c_{A\to R}^i = m_A^i Z^{k_A^i r}$, and $rk_{A\to RB}^i = Z^{k_A^i(r-1)}$. Thus:

$$\frac{c_{A\to R}^i}{c_A^i} = \frac{m_A^i Z^{k_A^i r}}{m_A^i Z^{k_A^i}} = \frac{Z^{k_A^i r}}{Z^{k_A^i}} = Z^{k_A^i r} Z^{-k_A^i} = Z^{k_A^i r - k_A^i} = Z^{k_A^i(r-1)} = rk_{A\to RB}^i$$ ■

Proposition 8. The result of the intermediate re-encryption of c_{AR}^i given $rk_{A\to RB}^i$ is $c_{AR\to R}^i = m_A^i Z^{k_A^i r + x_R^i} \neq c_A^i, c_{AR}^i, m_A^i$.

Proof. Let $c_A^i = m_A^i Z^{k_A^i}$, $c_{AR}^i = c_A^i Z^{x_R^i}$ where x_R^i is an ephemeral, randomly selected value in $\mathbb{Z}_q$ by R for each temporal index, and $rk_{A\to RB}^i = Z^{k_A^i(r-1)}$. Thus:

$$\begin{aligned} c_{AR\to R}^i &= c_{AR}^i\left(rk_{A\to RB}^i\right) = c_A^i Z^{x_R^i}\left(Z^{k_A^i(r-1)}\right) = \left(m_A^i Z^{k_A^i}\right) Z^{x_R^i}\left(Z^{k_A^i r - k_A^i}\right) = m_A^i Z^{k_A^i + x_R^i + k_A^i r - k_A^i} \\ &= m_A^i Z^{k_A^i r + x_R^i} \neq c_A^i, c_{AR}^i, m_A^i \end{aligned}$$ ■

Proposition 9. The result of delegatee decryption on $c_{AR\to R}^i$ given $rk_{AR\to B_1}^i$, $rk_{AR\to B_2}^i$, and sk_B is m_A^i.

Proof. Let $\varepsilon_{AR\to R}^i = \left(g^{ral_A}, c_{AR\to R}^i\right)$, $\varepsilon_{AR\to R_1}^i = \left(pk_R, c_{AR\to R_1}^i\right)$, $c_{AR\to R}^i = m_A^i Z^{k_A^i r + x_R^i}$, $c_{AR\to R_1}^i = m_A^i Z^{x_R^i}$, $rk_{AR\to B_1}^i = g^{bk_A^i/al_A}$, $rk_{AR\to B_2}^i = g^{bx_R^i/r}$, and $sk_B = b$. Thus:

Layer 1: $$\begin{aligned} c_{AR\to R_1}^i &= \frac{\varepsilon_{AR\to R}^{i,1}}{e\left(\varepsilon_{AR\to R}^{i,0}, rk_{AR\to B_1}^i\right)^{-sk_B}} = \frac{c_{AR\to R}^i}{e\left(g^{ral_A}, g^{bk_A^i/al_A}\right)^{-b}} = \frac{m_A^i Z^{k_A^i r + x_R^i}}{e(g,g)^{ral_A bk_A^i/al_A b}} = \frac{m_A^i Z^{k_A^i r + x_R^i}}{Z^{rk_A^i}} \\ &= m_A^i Z^{k_A^i r + x_R^i - rk_A^i} = m_A^i Z^{x_R^i} \end{aligned}$$

Layer 2: $\frac{\varepsilon^{i,1}_{AR\to R_1}}{e\left(\varepsilon^{i,0}_{AR\to R_1}, rk^i_{AR\to B_2}\right)^{-sk_B}} = \frac{c^i_{AR\to R_1}}{e\left(pk_R, g^{bx^i_R/r}\right)^{-b}} = \frac{m^i_A Z^{x^i_R}}{e\left(g^r, g^{bx^i_R/r}\right)^{-b}} = \frac{m^i_A Z^{x^i_R}}{e(g,g)^{rbx^i_R/rb}} = \frac{m^i_A Z^{x^i_R}}{Z^{x^i_R}}$

$= m^i_A$ ■

Proposition 10. Using c^i_{AR} and $c^i_{AR\to R}$, B can infer $rk^i_{A\to RB}$.

Proof. Let $c^i_{AR} = \left(m^i_A Z^{k^i_A}\right) Z^{x^i_R}$, $c^i_{AR\to R} = \left(m^i_A Z^{k^i_A r}\right) Z^{x^i_R}$, and $rk^i_{A\to RB} = Z^{k^i_A(r-1)}$. Thus:

$$\frac{c^i_{AR\to R}}{c^i_{AR}} = \frac{\left(m^i_A Z^{k^i_A r}\right) Z^{x^i_R}}{\left(m^i_A Z^{k^i_A}\right) Z^{x^i_R}} = \frac{Z^{k^i_A r}}{Z^{k^i_A}} = Z^{k^i_A r - k^i_A} = Z^{k^i_A(r-1)} = rk^i_{A\to RB}$$ ■

Proposition 11. Re-encrypting c^i_{AR} to $c^{i,j}_{AR}$ by R prevents B from computing the intermediate re-encryption step by way of $rk^i_{A\to RB}$ inferred via Proposition 10.

Proof. Let $c^i_{AR} = \left(m^i_A Z^{k^i_A}\right) Z^{x^i_R}$, $rk^i_{A\to RB} = Z^{k^i_A(r-1)}$ as derived by B (Proposition 10), and x^j_R is an ephemeral, randomly selected value in $\mathbb{Z}_q$ by R for each temporal index such that $x^j_R \neq x^i_R$. Thus:

R re-encrypts c^i_{AR} under x^j_R using the scalar $s^{i\to j}_R = Z^{x^j_R - x^i_R}$, yielding $c^{i,j}_{AR}$:

$$c^{i,j}_{AR} = c^i_{AR} s^{i\to j}_R = \left(m^i_A Z^{k^i_A}\right) Z^{x^i_R}\left(Z^{x^j_R - x^i_R}\right) = m^i_A Z^{k^i_A + x^i_R + x^j_R - x^i_R} = m^i_A Z^{k^i_A + x^j_R}$$

B performs the intermediate re-encryption step (Proposition 8) using $rk^i_{A\to RB}$ inferred by Proposition 10:

$$c^{i,j}_{AR\to R} = c^{i,j}_{AR}\left(Z^{k^i_A(r-1)}\right) = \left(m^i_A Z^{k^i_A + x^j_R}\right)\left(Z^{k^i_A r - k^i_A}\right) = m^i_A Z^{k^i_A + x^j_R + k^i_A r - k^i_A} = m^i_A Z^{x^j_R + k^i_A r}$$

B performs delegatee layer 1 decryption (Proposition 9):

$$c^{i,j}_{AR\to R_1} = \frac{c^{i,j}_{AR\to R}}{e\left(g^{ral_A}, g^{bk^i_A/al_A}\right)^{-b}} = \frac{m^i_A Z^{x^j_R + k^i_A r}}{e(g,g)^{ral_A bk^i_A/al_A b}} = \frac{m^i_A Z^{x^j_R + k^i_A r}}{Z^{rk^i_A}}$$

$$= m^i_A Z^{x^j_R + k^i_A r - rk^i_A} \quad = m_A Z^{x^j_R}$$

B performs delegatee layer 2 decryption (Proposition 9):

$$\frac{c^{i,j}_{AR\to R_1}}{e\left(pk_R, rk^i_{A\to RB}\right)^{-b}} \frac{m^i_A Z^{x^j_R}}{e\left(g^r, g^{bx^i_R/r}\right)^{-b}} = \frac{m^i_A Z^{x^j_R}}{e(g,g)^{rbx^i_R/rb}} = \frac{m^i_A Z^{x^j_R}}{Z^{x^i_R}} = m^i_A Z^{x^j_R - x^i_R} \neq m^i_A$$

Thus, even if B derives $rk^i_{A\to RB}$ via Proposition 10, if R re-encrypts c^i_{AR} to $c^{i,j}_{AR}$, B cannot retrieve m^i_A. ■

Proposition 12. The removal of $Z^{x_R^i}$ from c_{AR}^i and its subsequent update to $c_{AR}^{i,j}$ by R prior to r-encryption, results in a single-layer delegatee decryption process (Proposition 6 instead of 9) with the same strength as the dual (Proposition 11)

Proof. Let $c_A^i = m_A^i Z^{k_A^i}$, $c_{AR}^i = m_A^i Z^{k_A^i + x_R^i}$, $c_{A\to R}^i = m_A^i Z^{k_A^i r}$, and x_R^i and x_R^j be ephemeral, randomly selected values in $\mathbb{Z}_q$ by R for each temporal index such that $x_R^i \neq x_R^j$. Thus:

R removes $Z^{x_R^i}$:

$$\frac{c_{AR}^i}{Z^{x_R^i}} = \frac{m_A^i Z^{k_A^i + x_R^i}}{Z^{x_R^i}} = m_A^i Z^{k_A^i + x_R^i - x_R^i} = m_A^i Z^{k_A^i} = c_A^i$$

R re-encrypts following Proposition 5.

R updates c_{AR}^i to $c_{AR}^{i,j}$:

$$c_{AR}^{i,j} = c_{AR}^i \left(\frac{Z^{x_R^j}}{Z^{x_R^i}}\right) = m_A^i Z^{k_A^i + x_R^i} Z^{x_R^j - x_R^i} = m_A^i Z^{k_A^i + x_R^i + x_R^j - x_R^i} = m_A^i Z^{k_A^i + x_R^j}$$

B decrypts following Proposition 6.

Even if B calculates a scalar capable of converting c_{AR}^i to $c_{A\to R}^i$ (which B can decrypt):

$$s_{AR\to(A\to R)}^i = \frac{c_{A\to R}^i}{c_{AR}^i} = \frac{m_A^i Z^{k_A^i r}}{m_A^i Z^{k_A^i + x_R^i}} = \frac{Z^{k_A^i r}}{Z^{k_A^i + x_R^i}} = Z^{k_A^i r - k_A^i - x_R^i}$$

it cannot decrypt $c_{AR}^{i,j}$, thus exhibiting the same level of security as defined in Proposition 11:

$$c_{AR}^{i,j} s_{AR\to(A\to R)}^i = m_A^i Z^{k_A^i + x_R^j} Z^{k_A^i r - k_A^i - x_R^i} = m_A^i Z^{k_A^i + x_R^j + k_A^i r - k_A^i - x_R^i} = m_A^i Z^{x_R^j + k_A^i r - x_R^i} \neq c_{A\to R}^i \quad \blacksquare$$

B2 – Incremental Storage Mode

Incremental storage mode is an implementation option in which block data are stored in individual *fragments* and maintained incrementally (refer to Proposition 13). Instead of A's entire record being encrypted as one message, each element (e.g., an observation or encounter) is encrypted as an independent fragment. The benefit of this is three-fold. First, a logical block (i.e., the union all fragments) is of virtually limitless size. That is, it is no longer bound by the size of the structure storing the message (e.g., a byte array), forcing the creation of additional blocks. Second, it supports incremental updates, allowing A to add new and update/remove existing fragments. Thus, instead of re-encrypting and transmitting all data (which must then be propagated by R), only block modifications are processed. Lastly, it allows for scalar-based re-encryption. It is good practice to update encryption keys when data are re-encrypted, but this generally requires re-encrypting and transmitting all data – a prohibitively expensive step in terms of A's computation and

network bandwidth consumption. However, it is possible for A to re-encrypt all data via a simple scalar directly on R, avoiding the aforementioned pitfalls of key modification.

Proposition 14 is the foundation for fragment re-encryption by A. This new approach, however, exposes the encryption values $Z^{k_A^j}$ and $Z^{k_A^j+x_R^i}$ after a single decryption by B to B (refer to Proposition 15). Proposition 16 modifies the definition of R's ephemeral key x_R^i to fragment-based (i.e., $x_R^{f_i}$), which partially solves the problem. Instead of applying to all message fragments of temporal index j, the vulnerability affects only fragment f. This is removed by R re-encrypting the fragments after each access (refer to Proposition 17).

Proposition 13. A message m_A^i can be encrypted and decrypted as a set of fragments $\mathcal{F}$.

Proof. Let $m_A^{f_i}$ be a message fragment at temporal index i such that $\bigcup_{f\in\mathcal{F}} m_A^{f_i} = m_A^i$ and $\bigcap_{f\in\mathcal{F}} m_A^{f_i} = \emptyset$. Thus, encryption and decrypt is as follows.

Encryption of $m_A^{f_i}$ to $c_A^{f_i}$ and $c_{AR}^{f_i}$:

$$c_A^{f_i} = m_A^{f_i} Z^{k_A^i}$$

$$c_{AR}^{f_i} = m_A^{f_i} Z^{k_A^i} Z^{x_R^i} = m_A^{f_i} Z^{k_A^i + x_R^i}$$

Decryption of $c_A^{f_i}$ and $c_{AR}^{f_i}$ to $m_A^{f_i}$:

$$\frac{c_A^{f_i}}{e\left(g^{ak_A^i}, g\right)^{-a}} = \frac{m_A^{f_i} Z^{k_A^i}}{e(g,g)^{ak_A^i/a}} = \frac{m_A^{f_i} Z^{k_A^i}}{Z^{k_A^i}} = m_A^{f_i}$$

$$\frac{c_{AR}^{f_i}}{e\left(g^{ak_A^i}, g\right)^{-a} e(g,g)^{x_R^i}} = \frac{m_A^{f_i} Z^{k_A^i + x_R^i}}{e(g,g)^{ak_A^i/a} Z^{x_R^i}} = \frac{m_A^{f_i} Z^{k_A^i + x_R^i}}{Z^{k_A^i} Z^{x_R^i}} = \frac{m_A^{f_i} Z^{k_A^i + x_R^i}}{Z^{k_A^i + x_R^i}} = m_A^{f_i}$$

Therefore, full encryption of m_A^i is $c_A^i = \bigcup_{f\in\mathcal{F}} c_A^{f_i}$ and $c_{AR}^i = \bigcup_{f\in\mathcal{F}} c_{AR}^{f_i}$. Full decryption of c_A^i is $m_A^i = \bigcup_{f\in\mathcal{F}} \frac{c_A^{f_i}}{e\left(g^{ak_A^i}, g\right)^{-a}}$ and c_{AR}^i is $m_A^i = \bigcup_{f\in\mathcal{F}} \frac{c_{AR}^{f_i}}{\left(g^{ak_A^i}, g\right)^{-a} e(g,g)^{x_R^i}}$. ■

Proposition 14. The application of re-encryption scalar $s_A^{i\to j}$ (computed by A) by R to $c_A^{f_i}$ and $c_{AR}^{f_i}$ respectively, updates the encryption key associated with $m_A^{f_i}$ to $c_A^{f_{i,j}}$ and $c_{AR}^{f_{i,j,i}}$ respectively.

Proof. Let $c_A^{f_i} = m_A^{f_i} Z^{k_A^i}$ (Proposition 13), $c_{AR}^{f_i} = m_A^{f_i} Z^{k_A^i + x_R^i}$ (Proposition 13), and scalar $s_A^{i\to j} = Z^{k_A^j - k_A^i}$. Thus, re-encrypted $c_A^{f_i}$ is $c_A^{f_{i,j}}$ and $c_{AR}^{f_i}$ is $c_{AR}^{f_{i,j,i}}$:

$$c_A^{f_{i,j}} = c_A^{f_i} s_A^{i\to j} = \left(m_A^{f_i} Z^{k_A^i}\right) Z^{k_A^j - k_A^i} = m_A^{f_i} Z^{k_A^i + k_A^j - k_A^i} = m_A^{f_i} Z^{k_A^j}$$

$$c_{AR}^{f_{i,j,i}} = c_{AR}^{f_i} s_A^{i \to j} = \left(m_A^{f_i} Z^{k_A^i + x_R^i} \right) Z^{k_A^j - k_A^i} = m_A^{f_i} Z^{k_A^i + x_R^i + k_A^j - k_A^i} = m_A^{f_i} Z^{k_A^j + x_R^i} \quad \blacksquare$$

Proposition 15. Re-encrypting message fragments exposes $Z^{k_A^j}$ and $Z^{k_A^j + x_R^i}$ if $m_A^{f_i}$ is known, allowing for any message fragment m_A^* to be decrypted.

Proof. Let $c_A^{f_{i,j}} = m_A^{f_i} Z^{k_A^j}$ and $c_{AR}^{f_{i,j,i}} = m_A^{f_i} Z^{k_A^j + x_R^i}$ per Proposition 14. Once in possession of $m_A^{f_i}$ via Proposition 13, $Z^{k_A^j}$ and $Z^{k_A^j + x_R^i}$ can be computed as follows:

$$\frac{c_A^{f_{i,j}}}{m_A^{f_i}} = \frac{m_A^{f_i} Z^{k_A^j}}{m_A^{f_i}} = Z^{k_A^j}$$

$$\frac{c_{AR}^{f_{i,j,i}}}{m_A^{f_i}} = \frac{m_A^{f_i} Z^{k_A^j + x_R^i}}{m_A^{f_i}} = Z^{k_A^j + x_R^i}$$

Thus, any message fragment at any temporal index encrypted under j (i.e., m_A^*) can be decrypted:

$$\frac{c_A^{*_{*,j}}}{Z^{k_A^j}} = \frac{m_A^* Z^{k_A^j}}{Z^{k_A^j}} = m_A^*$$

$$\frac{c_{AR}^{*_{*,j,i}}}{Z^{k_A^j + x_R^i}} = \frac{m_A^* Z^{k_A^j + x_R^i}}{Z^{k_A^j + x_R^i}} = m_A^* \quad \blacksquare$$

Proposition 16. In a fragmented environment, assigning the ephemeral value to each fragment (i.e., $x_R^{f_*}$), prevents the negative outcome in Proposition 15 – i.e., the decryption of any message fragment at any temporal index encrypted under j.

Proof. Let $c_{AR}^{f_{i,j,i}} = m_A^{f_i} Z^{k_A^j + x_R^{f_i}}$. Once in possession of $m_A^{f_i}$ via Proposition 13, $Z^{k_A^j + x_R^{f_i}}$ can be computed as follows:

$$\frac{c_{AR}^{f_{i,j,i}}}{m_A^{f_i}} = \frac{m_A^{f_i} Z^{k_A^j + x_R^{f_i}}}{m_A^{f_i}} = Z^{k_A^j + x_R^{f_i}}$$

While this allows for the decryption of $m_A^{f_*}$ at any temporal index:

$$\frac{c_{AR}^{f_{*,j,i}}}{Z^{k_A^j + x_R^{f_i}}} = \frac{m_A^{f_*} Z^{k_A^j + x_R^{f_i}}}{Z^{k_A^j + x_R^{f_i}}} = m_A^{f_*}$$

it does not allow for the decryption of any other fragment $m_A^{t_*}$, where $t \neq f$:

$$\frac{c_{AR}^{t_{*,j,i}}}{Z^{k_A^j + x_R^{f_i}}} = \frac{m_A^{t_*} Z^{k_A^j + x_R^{t_i}}}{Z^{k_A^j + x_R^{f_i}}} = m_A^{t_*} Z^{k_A^j + x_R^{t_i} - k_A^j - x_R^{f_i}} = m_A^{t_*} Z^{x_R^{t_i} - x_R^{f_i}} \neq m_A^{t_*} \quad \blacksquare$$

Proposition 17. Re-encrypting $c_{AR}^{f_{i,j,i}}$ to $c_{AR}^{f_{i,j,j}}$ prevents the computation of $m_A^{f_*}$ from Proposition 16.

Proof. Let $c_{AR}^{f_{i,j,i}} = m_A^{f_i} Z^{k_A^j + x_R^{f_i}}$, $s_R^{i\to j} = Z^{x_R^{f_j} - x_R^{f_i}}$, and $Z^{k_A^j + x_R^{f_i}}$ be the decryption value computed in Proposition 16. Thus:

$$c_{AR}^{f_{i,j,j}} = c_{AR}^{f_{i,j,i}} s_R^{i\to j} = \left(m_A^{f_i} Z^{k_A^j + x_R^{f_i}}\right) Z^{x_R^{f_j} - x_R^{f_i}} = m_A^{f_i} Z^{k_A^j + x_R^{f_i} + x_R^{f_j} - x_R^{f_i}} = m_A^{f_i} Z^{k_A^j + x_R^{f_j}}$$

This cannot be decrypted by the computed scalar in Proposition 16:

$$\frac{c_{AR}^{f_{i,j,j}}}{Z^{k_A^j + x_R^{f_i}}} = \frac{m_A^{f_i} Z^{k_A^j + x_R^{f_j}}}{Z^{k_A^j + x_R^{f_i}}} = m_A^{f_i} Z^{k_A^j + x_R^{f_j} - k_A^j - x_R^{f_i}} = m_A^{t_*} Z^{x_R^{f_j} - x_R^{f_i}} \neq m_A^{f_i} \qquad \blacksquare$$

C – Sign-Verify Scheme

The sign and verify process ensures the integrity and origins of a message. Signing makes use of a value reasonably considered to be known only to the signee. Verification, on the other hand, is done using publicly held information on the signee. In proxy re-encryption, this corresponds to secret and public keys via encryption and decryption respectively. The logic behind this process is as follows. If the verifier can verify the message using the signee's public key and a hash of the message, then it must have originated with the signee – as only it has access to the secret key necessary to produce the signed message – and the message has not been altered.

The proposed scheme is effectively a digital signature algorithm. Digital signatures provide for *message integrity* (protects against message alteration), *authentication* (signed by the signee), and *non-repudiation* (the signee cannot claim the message was forged) through hashing and asymmetric primitives. The implemented process is as follows.

Given a message m_A^i, A generates the signed output $m_A^{\psi_i} = m_A^i Z^{x_A^i}$ (where x_A^i is an ephemeral, random number in $\mathbb{Z}_q$ such that $x_A^i \neq a, k_A^i, l_A$), a hash $H(m_A^i) = m_A^{\tau_i}$ of the message using any one-way cryptographic hashing function $H(\cdot)$ mapped to $\mathbb{Z}_q$, and a public verification parameter $g^{x_A^i/\left(sk_A + H\left(m_A^i\right)\right)} = g^{x_A^i/\left(a + m_A^{\tau_i}\right)}$. Signing with sk_A alone is insufficient as is incorporating the hash via the power of its inverse as opposed to addition (see Proposition 18 for the former and Propositions 19 and 20 for the latter).

Any user in $\mathcal{U}$ can verify $m_A^{\psi_i}$ by first computing the decryption parameter using A's public key and the message hash, $pk_A g^{m_A^{\tau_i}} = g^a g^{m_A^{\tau_i}} = g^{a + m_A^{\tau_i}}$, solving $\frac{m_A^i Z^{x_A^i}}{e\left(g^{x_A^i/\left(a + m_A^{\tau_i}\right)}, g^{a + m_A^{\tau_i}}\right)} = m_A^i$, then hashing the derived message and comparing it to the given hash. If they match, the message if verified. The verification process is formalized in Proposition 21.

Proposition 18. If A's signature is Z^{sk_A}, given only pk_A, any user in $\mathcal{U}$ can trivially compute Z^a and forge messages as if A.

Proof. Let A's signature be $Z^{sk_A} = Z^a$. Thus, any user (e.g., forger $F \in \mathcal{U} \neq A$) can compute $Z^{sk_A} = Z^a$:

$$e(pk_A, g) = e(g^a, g) = e(g, g)^a = Z^a$$

Hence, F can sign any message m_F^* as if A ($m_F^{\psi_*} = m_F^* Z^a$ and $H(m_F^*) = m_F^{\tau_*}$), which will verify as if originating with A:

$$\frac{m_F^{\psi_*}}{e(pk_A, g)} = \frac{m_F^* Z^a}{e(g^a, g)} = \frac{m_F^* Z^a}{e(g, g)^a} = \frac{m_F^* Z^a}{Z^a} = m_F^*$$

As $H(m_F^*)$ computed from the derived message is $m_F^{\tau_*}$, the message verifies as if from A. ■

Proposition 19. If A's public verification parameter incorporates $m_A^{\tau_i}$ using the power of its inverse, any user in $\mathcal{U}$ can trivially compute $Z^{x_A^i}$ and $g^{x_A^i/a}$ (if in possession of a signed message from A) and forge messages as if A.

Proof. Let $sk_A = a$ be the secret key for $A \in \mathcal{U}$; x_A^i an ephemeral key in $\mathbb{Z}_q$ such that $x_A^i \neq a, k_A^i, l_A$; $m_A^{\psi_i} = m_A^i Z^{x_A^i}$ the signed message; $pk_A = g^a$ the public key for A; $H(m_A^i) = m_A^{\tau_i}$ the message hash; and $\left(g^{x_A^i/sk_A}\right)^{-m_A^{\tau_i}} = g^{x_A^i/am_A^{\tau_i}}$ (which incorporates the hash using the power of its inverse) the public verification parameter. Thus, any user (e.g., forger $F \in \mathcal{U} \neq A$) can compute $Z^{x_A^i}$ and $g^{x_A^i/a}$ if in possession of a signed message from A:

$$e\left(g^{x_A^i/am_A^{\tau_i}}, pk_A^{m_A^{\tau_i}}\right) = e\left(g^{x_A^i/am_A^{\tau_i}}, g^{am_A^{\tau_i}}\right) = e(g, g)^{x_A^i am_A^{\tau_i}/am_A^{\tau_i}} = Z^{x_A^i}$$

F then removes the message binding from the public verification parameter:

$$\left(g^{x_A^i/am_A^{\tau_i}}\right)^{m_A^{\tau_i}} = g^{x_A^i m_A^{\tau_i}/am_A^{\tau_i}} = g^{x_A^i/a}$$

F can now forge any message m_F^* as if A ($m_F^{\psi_*} = m_F^* Z^{x_A^i}$, $H(m_F^*) = m_F^{\tau_*}$, and $\left(g^{x_A^i/a}\right)^{-m_F^{\tau_*}} = g^{x_A^i/am_F^{\tau_*}}$), which will verify as if originating with A:

$$\frac{m_F^{\psi_*}}{e\left(g^{x_A^i/am_F^{\tau_*}}, pk_A^{m_F^{\tau_*}}\right)} = \frac{m_F^* Z^{x_A^i}}{e\left(g^{x_A^i/am_F^{\tau_*}}, g^{am_F^{\tau_*}}\right)} = \frac{m_F^* Z^{x_A^i}}{e(g, g)^{x_A^i am_F^{\tau_*}/am_F^{\tau_*}}} \frac{m_F^* Z^{x_A^i}}{Z^{x_A^i}} = m_F^*$$

As $H(m_F^*)$ computed from the derived message is $m_F^{\tau_*}$, the message verifies as if from A. ■

Proposition 20. Incorporating the hash using the sum of sk_A and $m_A^{\tau_i}$, (resulting in $g^{x_A^i/\left(a+m_A^{\tau_i}\right)}$), prevents forging of message signatures without knowledge of sk_A and x_A^i.

Proof. Let $sk_A = a$ be the secret key for $A \in \mathcal{U}$; x_A^i an ephemeral key in $\mathbb{Z}_q$ such that $x_A^i \neq a, k_A^i, l_A$; $m_A^{\psi_i} = m_A^i Z^{x_A^i}$ the signed message; $pk_A = g^a$ the public key for A; $H\left(m_A^i\right) = m_A^{\tau_i}$ the message hash; g a public parameter; and $g^{x_A^i/\left(sk_A+H\left(m_A^i\right)\right)} = g^{x_A^i/\left(a+m_A^{\tau_i}\right)}$ (which incorporates the hash using the sum of sk_A and $m_A^{\tau_i}$) the public verification parameter. Thus, only users with knowledge of sk_A and x_A^i can sign messages as A:

$$\left(g^{x_A^i}\right)^{-\left(sk_A+H\left(m_A^i\right)\right)} = \left(g^{x_A^i}\right)^{-\left(a+m_A^{\tau_i}\right)} = g^{x_A^i/\left(a+m_A^{\tau_i}\right)}$$

Even though any user (e.g., forger $F \in \mathcal{U} \neq A$) can compute $Z^{x_A^i}$, which is used to encrypt the forged message m_F^* ($m_F^* Z^{x_A^i}$):

$$e\left(g^{x_A^i/\left(a+m_A^{\tau_i}\right)}, pk_A g^{m_A^{\tau_i}}\right) = e\left(g^{x_A^i/\left(a+m_A^{\tau_i}\right)}, g^a g^{m_A^{\tau_i}}\right) = e\left(g^{x_A^i/\left(a+m_A^{\tau_i}\right)}, g^{a+m_A^{\tau_i}}\right)$$
$$= e(g,g)^{x_A^i\left(a+m_A^{\tau_i}\right)/\left(a+m_A^{\tau_i}\right)} = Z^{x_A^i}$$

F does not possess sk_A or x_A^i and therefore cannot generate a valid public verification parameter. Hence, signed messages cannot be forged using the sum approach. ■

Proposition 21. The signed message $m_A^{\psi_i}$ is verifiable to any user in $\mathcal{U}$ given pk_A, a message hash $m_A^{\tau_i}$, and a public verification parameter $g^{x_A^i/\left(a+m_A^{\tau_i}\right)}$; the output of which is the verification set $m_A^{v_i}$.

Proof. Let $sk_A = a$ be the secret key for $A \in \mathcal{U}$; x_A^i an ephemeral key in $\mathbb{Z}_q$ such that $x_A^i \neq a, k_A^i, l_A$; $m_A^{\psi_i} = m_A^i Z^{x_A^i}$ the signed message; $pk_A = g^a$ the public key for A; $H\left(m_A^i\right) = m_A^{\tau_i}$ the message hash; g a public parameter; and $g^{x_A^i/\left(sk_A+H\left(m_A^i\right)\right)} = g^{x_A^i/\left(a+m_A^{\tau_i}\right)}$ the public verification parameter. Thus:

$$\frac{m_A^{\psi_i}}{e\left(g^{x_A^i/\left(sk_A+H(m_A^i)\right)}, pk_A g^{m_A^{\tau_i}}\right)} = \frac{m_A^i Z^{x_A^i}}{e\left(g^{x_A^i/\left(a+m_A^{\tau_i}\right)}, g^a g^{m_A^{\tau_i}}\right)} = \frac{m_A^i Z^{x_A^i}}{e\left(g^{x_A^i/\left(a+m_A^{\tau_i}\right)}, g^{a+m_A^{\tau_i}}\right)}$$
$$= \frac{m_A^i Z^{x_A^i}}{e(g,g)^{x_A^i\left(a+m_A^{\tau_i}\right)/\left(a+m_A^{\tau_i}\right)}} = \frac{m_A^i Z^{x_A^i}}{Z^{x_A^i}} = m_A^i$$

$$m_A^{v_i} = \begin{cases} \{\text{valid}, m_A^i\}, & \text{if } H\left(m_A^i\right) = m_A^{\tau_i} \\ \{\text{invalid}, \emptyset\}, & \text{if } H\left(m_A^i\right) \neq m_A^{\tau_i} \end{cases}$$ ■

This is trivially extended to an incremental storage configuration.

D – Encrypt-Sign and Decrypt-Verify Scheme

The sign-verify allows any user to verify the signed message $m_A^{\psi_i}$. This limits it use to public messages only, as one cannot designate a recipient of $m_A^{\psi_i}$ for verification. Consider the following example. A would like to send B a private message and B wants confirmation it is indeed from A. A can sign the message and send it to B, but anyone able to intercept $m_A^{\psi_i}$ can retrieve m_A^i; hence, m_A^i is not secured. The solution is for A to encrypt the signed message using pk_B. As only the holder of sk_B can decrypt the resulting cipher, A is relatively certain B alone has access to the message. B can then verify the authenticity of the message. The encrypt-sign and decrypt-verify processes can be achieved simultaneously with a simple modification to the sign-verify functions.

To encrypt-sign a message, A incorporates pk_B into the computation of the public verification parameter: $pk_B^{x_A^i/\left(sk_A+H\left(m_A^i\right)\right)} = g^{bx_A^i/\left(a+m_A^{\tau_i}\right)}$. The verification of the encrypt-signed cipher $c_A^{\psi_i} = m_A^i Z^{x_A^i}$ requires sk_B in addition to pk_A (refer to Proposition 22). As such, this configuration ensures only B can decrypt the message and it must have originated with A.

Proposition 22. The encrypt-signed message $c_A^{\psi_i}$ can only be decrypt-verified by B if signed by A; the output of which is the decrypt-verified set $m_A^{\upsilon_i}$.

Proof. Let $sk_B = b$ be the secret key for $B \in \mathcal{U}$, x_A^i an ephemeral key in $\mathbb{Z}_q$ such that $x_A^i \neq a, k_A^i, l_A$, $c_A^{\psi_i} = m_A^i Z^{x_A^i}$ the encrypt-signed cipher, $pk_A = g^a$ the public key for A, $pk_B = g^b$ the public key for B, $H\left(m_A^i\right) = m_A^{\tau_i}$ the message hash, g a public parameter, and $g^{pk_B x_A^i/\left(sk_A+H\left(m_A^i\right)\right)} = g^{bx_A^i/\left(a+m_A^{\tau_i}\right)}$ the public verification parameter. Thus:

$$\frac{c_A^{\psi_i}}{e\left(g^{pk_B x_A^i/\left(sk_A+H\left(m_A^i\right)\right)}, pk_A g^{m_A^{\tau_i}}\right)^{-sk_B}} = \frac{m_A^i Z^{x_A^i}}{e\left(g^{bx_A^i/\left(a+m_A^{\tau_i}\right)}, g^a g^{m_A^{\tau_i}}\right)^{-b}}$$

$$= \frac{m_A^i Z^{x_A^i}}{e\left(g^{bx_A^i/\left(a+m_A^{\tau_i}\right)}, g^{a+m_A^{\tau_i}}\right)^{-b}} = \frac{m_A^i Z^{x_A^i}}{e(g,g)^{bx_A^i\left(a+m_A^{\tau_i}\right)/b\left(a+m_A^{\tau_i}\right)}} = \frac{m_A^i Z^{x_A^i}}{Z^{x_A^i}} = m_A^i$$

$$m_A^{\upsilon_i} = \begin{cases} \{\text{valid}, m_A^i\}, & \text{if } H\left(m_A^i\right) = m_A^{\tau_i} \\ \{\text{invalid}, \emptyset\}, & \text{if } H\left(m_A^i\right) \neq m_A^{\tau_i} \end{cases} \quad \blacksquare$$

This is trivially extended to an incremental storage configuration.

References

1. Ateniese G, Fu K, Green M, Hohenberger S. Improved Proxy Re-encryption Schemes with Applications to Secure Distributed Storage. *ACM Transactions on Information and System Security*. 2006;9(1):1–30. doi:10.1145/1127345.1127346
2. Blaze M, Bleumer G, Strauss M. Divertible protocols and atomic proxy cryptography. In: *Advances in Cryptology — EUROCRYPT'98*. Lecture Notes in Computer Science. Springer, Berlin, Heidelberg; 1998:127-144. doi:10.1007/BFb0054122
3. Elgamal T. A public key cryptosystem and a signature scheme based on discrete logarithms. *IEEE Transactions on Information Theory*. 1985;31(4):469-472. doi:10.1109/TIT.1985.1057074
