# Supplementary material for: A Blockchain Framework for Patient-Centered Health Records and Exchange (HealthChain): Evaluation and Proof-of-Concept Study
Source: J Med Internet Res. 2019 Aug 31;21(8):e13592. doi: 10.2196/13592 (PMC6743266; doi:10.2196/13592)
Supplement: Multimedia Appendix 2 [file jmir_v21i8e13592_app2.pdf]

Multimedia Appendix 2

Experimental Configurations

HealthChain has four configurable modes, but not all are relevant to certain measures; hence, results list two-, three-, or four-letter abbreviations. Herein, an overview of the modes (Table 1) and abbreviations (Tables 2-5) are presented.

Table 1. Mode definitions and configuration options.

Mode	Option 1	Option 2
Block encryption	**A**: AES-encrypted data with a PRE-encrypted key	**P**: Proxy re-encryption (PRE) encrypted data
Storage	**F**: Full-block – all data stored in a single block	**I**: Incremental – each new entry is a new record
Encryption key	**S**: Static – one key for the life of the block	**D**: Dynamic – a new key for each action
Server-side encryption	**Y**: Yes – server encrypts its data using an ephemeral key in dynamic mode for each entry following the chosen block encryption mode	**N**: No – server does not encrypt its data (note: the data are still encrypted under the users' keys)

Table 2. Two-letter abbreviations: excludes key encryption and server-side encryption.

Abbr	Block Enc	Storage	Experiments
AF	AES+PRE	Full Block	**Network latency (server-to-client):** *Block encryption* determines cipher size. *Storage* accounts for cipher padding.
AI	AES+PRE	Incremental	
PF	PRE	Full Block	
PI	PRE	Incremental	

Table 3. Three-letter abbreviations: excludes server-side encryption.

Abbr	Block Enc	Enc Key	Storage	Experiments
ADF	AES+PRE	Dynamic	Full Block	**Transmission size/network latency (client-to-sever)**: *Block encryption* affects cipher size. *Dynamic encryption keys* bloat transactions with updated smart contracts, scalars, and keys. *Storage* accounts for cipher padding. **Client processing time**: *Block encryption* impacts performance. *Encryption key* determines rekeying and smart contract
ADI	AES+PRE	Dynamic	Incremental	
ASF	AES+PRE	Static	Full Block	
ASI	AES+PRE	Static	Incremental	
PDF	PRE	Dynamic	Full Block	
PDI	PRE	Dynamic	Incremental	
PSF	PRE	Static	Full Block	
PSI	PRE	Static	Incremental	

				regeneration. *Storage* affects the amount of data to be processed.

Table 4. Three-letter abbreviations: excludes encryption key mode.

Abbr	Block Enc	Storage	Server Enc	Experiments
AFN	AES+PRE	Full Block	No	**Smart contract execution**: *Block encryption* impacts performance. *Storage* affects the number of records to be decrypted. *Server-side encryption* impacts performance as a value of Y requires each record to first be decrypted by a unique ephemeral key.
AFY	AES+PRE	Full Block	Yes	
AIN	AES+PRE	Incremental	No	
AIY	AES+PRE	Incremental	Yes	
PFN	PRE	Full Block	No	
PFY	PRE	Full Block	Yes	
PIN	PRE	Incremental	No	
PIY	PRE	Incremental	Yes	

Table 5. Four-letter abbreviations.

Abbr	Block Enc	Enc Key	Storage	Server Enc	Experiments
ADFN	AES+PRE	Dynamic	Full Block	No	**Server processing time**: *Block encryption* has a significant impact on performance. *Encryption key* determines rekeying. *Storage* affects the amount of data to be processed. *Server-side encryption* impacts performance as a value of Y requires each record to be encrypted by a unique ephemeral key.
ADFY	AES+PRE	Dynamic	Full Block	Yes	
ADIN	AES+PRE	Dynamic	Incremental	No	
ADIY	AES+PRE	Dynamic	Incremental	Yes	
ASFN	AES+PRE	Static	Full Block	No	
ASFY	AES+PRE	Static	Full Block	Yes	
ASIN	AES+PRE	Static	Incremental	No	
ASIY	AES+PRE	Static	Incremental	Yes	
PDFN	PRE	Dynamic	Full Block	No	
PDFY	PRE	Dynamic	Full Block	Yes	
PDIN	PRE	Dynamic	Incremental	No	
PDIY	PRE	Dynamic	Incremental	Yes	
PSFN	PRE	Static	Full Block	No	
PSFY	PRE	Static	Full Block	Yes	
PSIN	PRE	Static	Incremental	No	
PSIY	PRE	Static	Incremental	Yes	
