# Supplementary material for: A Blockchain Framework for Patient-Centered Health Records and Exchange (HealthChain): Evaluation and Proof-of-Concept Study
Source: J Med Internet Res. 2019 Aug 31;21(8):e13592. doi: 10.2196/13592 (PMC6743266; doi:10.2196/13592)
Supplement: Multimedia Appendix 3 [file jmir_v21i8e13592_app3.zip › ChameleonHashing/javadoc/allclasses-frame.html]

All Classes


# All Classes

- ChameleonHash
- ChameleonHash.STORAGE
- Hash
- MessageDigest
- MessageDigest.Algorithms
- PublicCoinChameleonHash
- PublicCoinHash
- RSAChameleonHash
- RSAHash
