# Supplementary material for: A Blockchain Framework for Patient-Centered Health Records and Exchange (HealthChain): Evaluation and Proof-of-Concept Study
Source: J Med Internet Res. 2019 Aug 31;21(8):e13592. doi: 10.2196/13592 (PMC6743266; doi:10.2196/13592)
Supplement: Multimedia Appendix 3 [file jmir_v21i8e13592_app3.zip › ChameleonHashing/javadoc/constant-values.html]

Constant Field Values


JavaScript is disabled on your browser.


Skip navigation links


- Overview
- Package
- Class
- Use
- Tree
- Deprecated
- Index
- Help

- Prev
- Next

- Frames
- No Frames

- All Classes

# Constant Field Values

## Contents

- edu.ecu.\*

## edu.ecu.\*

- edu.ecu.hsim.ray.chameleonhash.ChameleonHash

  | Modifier and Type | Constant Field | Value |
  |  |  |  |
  | --- | --- | --- |
  | `protected static final int` | `DEFAULT_BIT_LENGTH` | `256` |

Skip navigation links


- Overview
- Package
- Class
- Use
- Tree
- Deprecated
- Index
- Help

- Prev
- Next

- Frames
- No Frames

- All Classes
