# Supplementary material for: A Blockchain Framework for Patient-Centered Health Records and Exchange (HealthChain): Evaluation and Proof-of-Concept Study
Source: J Med Internet Res. 2019 Aug 31;21(8):e13592. doi: 10.2196/13592 (PMC6743266; doi:10.2196/13592)
Supplement: Multimedia Appendix 3 [file jmir_v21i8e13592_app3.zip › ChameleonHashing/javadoc/edu/ecu/hsim/ray/chameleonhash/ChameleonHash.html]

ChameleonHash


JavaScript is disabled on your browser.


Skip navigation links


- Overview
- Package
- Class
- Use
- Tree
- Deprecated
- Index
- Help

- Prev Class
- Next Class

- Frames
- No Frames

- All Classes

- Summary:
- Nested |
- Field |
- Constr |
- Method

- Detail:
- Field |
- Constr |
- Method


edu.ecu.hsim.ray.chameleonhash

## Class ChameleonHash

- java.lang.Object
- - edu.ecu.hsim.ray.chameleonhash.ChameleonHash

- Direct Known Subclasses:
  :   PublicCoinChameleonHash, RSAChameleonHash

  ---

    

  ```
  public abstract class ChameleonHash
  extends java.lang.Object
  ```

  Abstract Chameleon Hash class. This manages `p`, `q` and the
  property files.

- - ### Nested Class Summary

    Nested Classes

    | Modifier and Type | Class and Description |
    | `protected static class` | `ChameleonHash.STORAGE` Storage volatility options: Storage volatility refers how the generated keys will be stored and read. |
  - ### Field Summary

    Fields

    | Modifier and Type | Field and Description |
    | `protected boolean` | `createProperties` Create properties file `boolean` parameter. |
    | `protected static int` | `DEFAULT_BIT_LENGTH` Default bit length. |
    | `static java.nio.charset.Charset` | `ENCODING` `Charset` encoding. |
    | `protected java.io.FileInputStream` | `fisPub` Public properties `FileInputStream`. |
    | `protected java.io.FileInputStream` | `fisSec` Secret properties `FileInputStream`. |
    | `protected java.io.FileOutputStream` | `fosPub` Public properties `FileOutputStream`. |
    | `protected java.io.FileOutputStream` | `fosSec` Secret properties `FileOutputStream`. |
    | `protected java.math.BigInteger` | `N` `N = pq`. |
    | `protected java.math.BigInteger` | `one` One as `BigInteger`. |
    | `protected java.math.BigInteger` | `p` `p = 2q+1`. |
    | `protected java.math.BigInteger` | `phiN` Phi of `N`. |
    | `protected java.math.BigInteger` | `pm1` . |
    | `protected java.util.Properties` | `propsPublic` Public `Properties` file. |
    | `protected java.util.Properties` | `propsSecret` Secret `Properties` file. |
    | `protected java.io.File` | `publicFile` Public properties `File`. |
    | `protected java.lang.String` | `publicStringProperties` Public properties as `String`. |
    | `protected java.math.BigInteger` | `q` `q`. |
    | `protected java.math.BigInteger` | `qm1` `q-1`. |
    | `protected java.io.File` | `secretFile` Secret properties `File`. |
    | `protected java.lang.String` | `secretStringProperties` Secret properties as `String`. |
    | `protected java.io.StringReader` | `sisPub` Public properties `StringReader`. |
    | `protected java.io.StringReader` | `sisSec` Secret properties `StringReader`. |
    | `protected java.io.StringWriter` | `sosPub` Public properties `StringWriter`. |
    | `protected java.io.StringWriter` | `sosSec` Secret properties `StringWriter`. |
    | `protected ChameleonHash.STORAGE` | `storage` `ChameleonHash.STORAGE` parameter. |
    | `protected java.math.BigInteger` | `two` Two as `BigInteger`. |
    | `protected java.math.BigInteger` | `zero` Zero as `BigInteger`. |
  - ### Constructor Summary

    Constructors

    | Modifier | Constructor and Description |
    | `protected` | `ChameleonHash(java.math.BigInteger p, java.math.BigInteger q, int bitLength, java.lang.String file)` Constructor. |
    | `protected` | `ChameleonHash(java.math.BigInteger p, java.math.BigInteger q, int bitLength, java.lang.String publicProperties, java.lang.String secretProperties)` Constructor. |
  - ### Method Summary

    All Methods Instance Methods Abstract Methods Concrete Methods

    | Modifier and Type | Method and Description |
    | `void` | `close()` Closes the file input and output streams. |
    | `abstract Hash` | `forge(byte[] message, Hash previousHash)` Forges the hash by creating new `r` and `s` parameters for the new message and prior hash. |
    | `abstract Hash` | `forge(java.lang.String message, Hash previousHash)` Forges the hash by creating new `r` and `s` parameters for the new message and prior hash. |
    | `java.io.File` | `getPublicFile()` Returns the public file if `storage` == `ChameleonHash.STORAGE.NONVOLATILE`. |
    | `java.lang.String` | `getPublicProperties()` Returns public properties as a `String`. |
    | `java.io.File` | `getSecretFile()` Returns the secret file if `storage` == `ChameleonHash.STORAGE.NONVOLATILE`. |
    | `java.lang.String` | `getSecretProperties()` Returns secret properties as a `String`. |
    | `abstract Hash` | `hash(byte[] message)` Hashes a message. |
    | `abstract Hash` | `hash(java.lang.String message)` Hashes a message. |
    | `protected java.math.BigInteger` | `random(java.math.BigInteger bound)` Generates a random `BigInteger` with upper bound. |
    | `protected java.math.BigInteger` | `random(java.math.BigInteger lower, java.math.BigInteger upper)` Generates a random `BigInteger` with upper and lower bounds. |
    | `abstract boolean` | `verify(byte[] message, Hash hash)` Verifies a message against a known `Hash`. |
    | `abstract boolean` | `verify(java.lang.String message, Hash hash)` Verifies a message against a known `Hash`. |

    - ### Methods inherited from class java.lang.Object

      `clone, equals, finalize, getClass, hashCode, notify, notifyAll, toString, wait, wait, wait`

- - ### Field Detail


    - #### ENCODING

      ```
      public static final java.nio.charset.Charset ENCODING
      ```

      `Charset` encoding.


    - #### DEFAULT\_BIT\_LENGTH

      ```
      protected static final int DEFAULT_BIT_LENGTH
      ```

      Default bit length.

      See Also:
      :   Constant Field Values


    - #### storage

      ```
      protected final ChameleonHash.STORAGE storage
      ```

      `ChameleonHash.STORAGE` parameter.


    - #### publicFile

      ```
      protected java.io.File publicFile
      ```

      Public properties `File`.


    - #### publicStringProperties

      ```
      protected java.lang.String publicStringProperties
      ```

      Public properties as `String`.


    - #### secretFile

      ```
      protected java.io.File secretFile
      ```

      Secret properties `File`.


    - #### secretStringProperties

      ```
      protected java.lang.String secretStringProperties
      ```

      Secret properties as `String`.


    - #### fisPub

      ```
      protected java.io.FileInputStream fisPub
      ```

      Public properties `FileInputStream`.


    - #### fisSec

      ```
      protected java.io.FileInputStream fisSec
      ```

      Secret properties `FileInputStream`.


    - #### fosPub

      ```
      protected java.io.FileOutputStream fosPub
      ```

      Public properties `FileOutputStream`.


    - #### fosSec

      ```
      protected java.io.FileOutputStream fosSec
      ```

      Secret properties `FileOutputStream`.


    - #### sisPub

      ```
      protected java.io.StringReader sisPub
      ```

      Public properties `StringReader`.


    - #### sisSec

      ```
      protected java.io.StringReader sisSec
      ```

      Secret properties `StringReader`.


    - #### sosPub

      ```
      protected java.io.StringWriter sosPub
      ```

      Public properties `StringWriter`.


    - #### sosSec

      ```
      protected java.io.StringWriter sosSec
      ```

      Secret properties `StringWriter`.


    - #### propsPublic

      ```
      protected final java.util.Properties propsPublic
      ```

      Public `Properties` file.


    - #### propsSecret

      ```
      protected final java.util.Properties propsSecret
      ```

      Secret `Properties` file.


    - #### createProperties

      ```
      protected boolean createProperties
      ```

      Create properties file `boolean` parameter.


    - #### q

      ```
      protected java.math.BigInteger q
      ```

      `q`.


    - #### p

      ```
      protected java.math.BigInteger p
      ```

      `p = 2q+1`.


    - #### N

      ```
      protected java.math.BigInteger N
      ```

      `N = pq`.


    - #### pm1

      ```
      protected java.math.BigInteger pm1
      ```

      .


    - #### qm1

      ```
      protected java.math.BigInteger qm1
      ```

      `q-1`.


    - #### phiN

      ```
      protected java.math.BigInteger phiN
      ```

      Phi of `N`.


    - #### zero

      ```
      protected final java.math.BigInteger zero
      ```

      Zero as `BigInteger`.


    - #### one

      ```
      protected final java.math.BigInteger one
      ```

      One as `BigInteger`.


    - #### two

      ```
      protected final java.math.BigInteger two
      ```

      Two as `BigInteger`.
  - ### Constructor Detail


    - #### ChameleonHash

      ```
      protected ChameleonHash(java.math.BigInteger p,
                              java.math.BigInteger q,
                              int bitLength,
                              java.lang.String file)
                       throws java.io.IOException
      ```

      Constructor. This generates the base `ChameleonHash.STORAGE.NONVOLATILE`
      structures and files used by all extending classes.

      Parameters:
      :   `p` - prime p or `null`
      :   `q` - prime q or `null`
      :   `bitLength` - bit length
      :   `file` - file name

      Throws:
      :   `java.io.IOException` - `IOException`


    - #### ChameleonHash

      ```
      protected ChameleonHash(java.math.BigInteger p,
                              java.math.BigInteger q,
                              int bitLength,
                              java.lang.String publicProperties,
                              java.lang.String secretProperties)
                       throws java.io.IOException
      ```

      Constructor. This generates the base `ChameleonHash.STORAGE.VOLATILE` structures
      and files used by all extending classes.

      Parameters:
      :   `p` - prime p or `null`
      :   `q` - prime q or `null`
      :   `bitLength` - bit length
      :   `publicProperties` - public properties in `String` form
      :   `secretProperties` - secret properties in `String` form

      Throws:
      :   `java.io.IOException` - `IOException`
  - ### Method Detail


    - #### getPublicFile

      ```
      public java.io.File getPublicFile()
      ```

      Returns the public file if `storage` ==
      `ChameleonHash.STORAGE.NONVOLATILE`.

      Returns:
      :   the public file


    - #### getSecretFile

      ```
      public java.io.File getSecretFile()
      ```

      Returns the secret file if `storage` ==
      `ChameleonHash.STORAGE.NONVOLATILE`.

      Returns:
      :   the secret file


    - #### getPublicProperties

      ```
      public java.lang.String getPublicProperties()
      ```

      Returns public properties as a `String`.

      Returns:
      :   public properties as a `String`


    - #### getSecretProperties

      ```
      public java.lang.String getSecretProperties()
      ```

      Returns secret properties as a `String`.

      Returns:
      :   secret properties as a `String`


    - #### close

      ```
      public void close()
                 throws java.io.IOException
      ```

      Closes the file input and output streams.

      Throws:
      :   `java.io.IOException` - `IOException`


    - #### hash

      ```
      public abstract Hash hash(java.lang.String message)
                         throws java.io.IOException,
                                java.security.NoSuchAlgorithmException
      ```

      Hashes a message.

      Parameters:
      :   `message` - message to hash

      Returns:
      :   `Hash` of message

      Throws:
      :   `java.io.IOException` - `IOException`
      :   `java.security.NoSuchAlgorithmException` - `NoSuchAlgorithmException`


    - #### hash

      ```
      public abstract Hash hash(byte[] message)
                         throws java.io.IOException,
                                java.security.NoSuchAlgorithmException
      ```

      Hashes a message.

      Parameters:
      :   `message` - message to hash

      Returns:
      :   `Hash` of message

      Throws:
      :   `java.io.IOException` - `IOException`
      :   `java.security.NoSuchAlgorithmException` - `NoSuchAlgorithmException`


    - #### verify

      ```
      public abstract boolean verify(java.lang.String message,
                                     Hash hash)
                              throws java.io.IOException,
                                     java.security.NoSuchAlgorithmException
      ```

      Verifies a message against a known `Hash`.

      Parameters:
      :   `message` - message to verify
      :   `hash` - `Hash`

      Returns:
      :   `true` if verified, `false` otherwise

      Throws:
      :   `java.io.IOException` - `IOException`
      :   `java.security.NoSuchAlgorithmException` - `NoSuchAlgorithmException`


    - #### verify

      ```
      public abstract boolean verify(byte[] message,
                                     Hash hash)
                              throws java.io.IOException,
                                     java.security.NoSuchAlgorithmException
      ```

      Verifies a message against a known `Hash`.

      Parameters:
      :   `message` - message to verify
      :   `hash` - `Hash`

      Returns:
      :   `true` if verified, `false` otherwise

      Throws:
      :   `java.io.IOException` - `IOException`
      :   `java.security.NoSuchAlgorithmException` - `NoSuchAlgorithmException`


    - #### forge

      ```
      public abstract Hash forge(java.lang.String message,
                                 Hash previousHash)
                          throws java.io.IOException,
                                 java.security.NoSuchAlgorithmException
      ```

      Forges the hash by creating new `r` and `s` parameters for
      the new message and prior hash.

      Parameters:
      :   `message` - message
      :   `previousHash` - previous `Hash`

      Returns:
      :   the forged `Hash`

      Throws:
      :   `java.io.IOException` - `IOException`
      :   `java.security.NoSuchAlgorithmException` - `NoSuchAlgorithmException`


    - #### forge

      ```
      public abstract Hash forge(byte[] message,
                                 Hash previousHash)
                          throws java.io.IOException,
                                 java.security.NoSuchAlgorithmException
      ```

      Forges the hash by creating new `r` and `s` parameters for
      the new message and prior hash.

      Parameters:
      :   `message` - message
      :   `previousHash` - previous `Hash`

      Returns:
      :   the forged `Hash`

      Throws:
      :   `java.io.IOException` - `IOException`
      :   `java.security.NoSuchAlgorithmException` - `NoSuchAlgorithmException`


    - #### random

      ```
      protected java.math.BigInteger random(java.math.BigInteger bound)
      ```

      Generates a random `BigInteger` with upper bound.

      Parameters:
      :   `bound` - upper bound

      Returns:
      :   a random `BigInteger`


    - #### random

      ```
      protected java.math.BigInteger random(java.math.BigInteger lower,
                                            java.math.BigInteger upper)
      ```

      Generates a random `BigInteger` with upper and lower bounds.

      Parameters:
      :   `lower` - lower bound
      :   `upper` - upper bound

      Returns:
      :   a random `BigInteger`


Skip navigation links


- Overview
- Package
- Class
- Use
- Tree
- Deprecated
- Index
- Help

- Prev Class
- Next Class

- Frames
- No Frames

- All Classes

- Summary:
- Nested |
- Field |
- Constr |
- Method

- Detail:
- Field |
- Constr |
- Method
