# Supplementary material for: A Blockchain Framework for Patient-Centered Health Records and Exchange (HealthChain): Evaluation and Proof-of-Concept Study
Source: J Med Internet Res. 2019 Aug 31;21(8):e13592. doi: 10.2196/13592 (PMC6743266; doi:10.2196/13592)
Supplement: Multimedia Appendix 3 [file jmir_v21i8e13592_app3.zip › ChameleonHashing/javadoc/edu/ecu/hsim/ray/chameleonhash/ChameleonHash.STORAGE.html]

ChameleonHash.STORAGE


JavaScript is disabled on your browser.


Skip navigation links


- Overview
- Package
- Class
- Use
- Tree
- Deprecated
- Index
- Help

- Prev Class
- Next Class

- Frames
- No Frames

- All Classes

- Summary:
- Nested |
- Enum Constants |
- Field |
- Method

- Detail:
- Enum Constants |
- Field |
- Method


edu.ecu.hsim.ray.chameleonhash

## Enum ChameleonHash.STORAGE

- java.lang.Object
- - java.lang.Enum<ChameleonHash.STORAGE>
  - - edu.ecu.hsim.ray.chameleonhash.ChameleonHash.STORAGE

- All Implemented Interfaces:
  :   java.io.Serializable, java.lang.Comparable<ChameleonHash.STORAGE>

  Enclosing class:
  :   ChameleonHash

  ---

    

  ```
  protected static enum ChameleonHash.STORAGE
  extends java.lang.Enum<ChameleonHash.STORAGE>
  ```

  Storage volatility options:
  Storage volatility refers how the generated keys will be stored and read.
  `NONVOLATILE` means they will be stored to and read from
  files, as indicated by either the default settings or a provided path and
  file template. This is best when one does not desire to manage keys.
  `VOLATILE` indicates a memory-only approach. This requires
  the user to maintain the generated public and secret keys as
  `String` objects, which are then to be provided during
  reinitialization.

- - ### Enum Constant Summary

    Enum Constants

    | Enum Constant and Description |
    | `NONVOLATILE` Nonvolatile storage. |
    | `VOLATILE` Volatile storage. |
  - ### Method Summary

    All Methods Static Methods Concrete Methods

    | Modifier and Type | Method and Description |
    | `static ChameleonHash.STORAGE` | `valueOf(java.lang.String name)` Returns the enum constant of this type with the specified name. |
    | `static ChameleonHash.STORAGE[]` | `values()` Returns an array containing the constants of this enum type, in the order they are declared. |

    - ### Methods inherited from class java.lang.Enum

      `clone, compareTo, equals, finalize, getDeclaringClass, hashCode, name, ordinal, toString, valueOf`
    - ### Methods inherited from class java.lang.Object

      `getClass, notify, notifyAll, wait, wait, wait`

- - ### Enum Constant Detail


    - #### NONVOLATILE

      ```
      public static final ChameleonHash.STORAGE NONVOLATILE
      ```

      Nonvolatile storage.


    - #### VOLATILE

      ```
      public static final ChameleonHash.STORAGE VOLATILE
      ```

      Volatile storage.
  - ### Method Detail


    - #### values

      ```
      public static ChameleonHash.STORAGE[] values()
      ```

      Returns an array containing the constants of this enum type, in
      the order they are declared. This method may be used to iterate
      over the constants as follows:

      ```
      for (ChameleonHash.STORAGE c : ChameleonHash.STORAGE.values())
          System.out.println(c);
      ```

      Returns:
      :   an array containing the constants of this enum type, in the order they are declared


    - #### valueOf

      ```
      public static ChameleonHash.STORAGE valueOf(java.lang.String name)
      ```

      Returns the enum constant of this type with the specified name.
      The string must match *exactly* an identifier used to declare an
      enum constant in this type. (Extraneous whitespace characters are
      not permitted.)

      Parameters:
      :   `name` - the name of the enum constant to be returned.

      Returns:
      :   the enum constant with the specified name

      Throws:
      :   `java.lang.IllegalArgumentException` - if this enum type has no constant with the specified name
      :   `java.lang.NullPointerException` - if the argument is null


Skip navigation links


- Overview
- Package
- Class
- Use
- Tree
- Deprecated
- Index
- Help

- Prev Class
- Next Class

- Frames
- No Frames

- All Classes

- Summary:
- Nested |
- Enum Constants |
- Field |
- Method

- Detail:
- Enum Constants |
- Field |
- Method
