# Supplementary material for: A Blockchain Framework for Patient-Centered Health Records and Exchange (HealthChain): Evaluation and Proof-of-Concept Study
Source: J Med Internet Res. 2019 Aug 31;21(8):e13592. doi: 10.2196/13592 (PMC6743266; doi:10.2196/13592)
Supplement: Multimedia Appendix 3 [file jmir_v21i8e13592_app3.zip › ChameleonHashing/javadoc/edu/ecu/hsim/ray/chameleonhash/class-use/RSAHash.html]

Uses of Class edu.ecu.hsim.ray.chameleonhash.RSAHash


JavaScript is disabled on your browser.


Skip navigation links


- Overview
- Package
- Class
- Use
- Tree
- Deprecated
- Index
- Help

- Prev
- Next

- Frames
- No Frames

- All Classes

## Uses of Class edu.ecu.hsim.ray.chameleonhash.RSAHash

- Packages that use RSAHash

  | Package | Description |
  |  |  |
  | --- | --- |
  | edu.ecu.hsim.ray.chameleonhash |  |
- - ### Uses of RSAHash in edu.ecu.hsim.ray.chameleonhash

    Methods in edu.ecu.hsim.ray.chameleonhash that return RSAHash

    | Modifier and Type | Method and Description |
    |  |  |
    | --- | --- |
    | `RSAHash` | RSAChameleonHash.`forge(byte[] message, Hash previousHash)` |
    | `RSAHash` | RSAChameleonHash.`forge(java.lang.String message, Hash previousHash)` |
    | `RSAHash` | RSAChameleonHash.`hash(byte[] message)` |
    | `RSAHash` | RSAChameleonHash.`hash(java.lang.String message)` |

Skip navigation links


- Overview
- Package
- Class
- Use
- Tree
- Deprecated
- Index
- Help

- Prev
- Next

- Frames
- No Frames

- All Classes
