# Supplementary material for: A Blockchain Framework for Patient-Centered Health Records and Exchange (HealthChain): Evaluation and Proof-of-Concept Study
Source: J Med Internet Res. 2019 Aug 31;21(8):e13592. doi: 10.2196/13592 (PMC6743266; doi:10.2196/13592)
Supplement: Multimedia Appendix 3 [file jmir_v21i8e13592_app3.zip › ChameleonHashing/javadoc/edu/ecu/hsim/ray/chameleonhash/Hash.html]

Hash


JavaScript is disabled on your browser.


Skip navigation links


- Overview
- Package
- Class
- Use
- Tree
- Deprecated
- Index
- Help

- Prev Class
- Next Class

- Frames
- No Frames

- All Classes

- Summary:
- Nested |
- Field |
- Constr |
- Method

- Detail:
- Field |
- Constr |
- Method


edu.ecu.hsim.ray.chameleonhash

## Class Hash

- java.lang.Object
- - edu.ecu.hsim.ray.chameleonhash.Hash

- Direct Known Subclasses:
  :   PublicCoinHash, RSAHash

  ---

    

  ```
  public class Hash
  extends java.lang.Object
  ```

  Stores a message hash and parameter `r`.

- - ### Constructor Summary

    Constructors

    | Modifier | Constructor and Description |
    | `protected` | `Hash(java.math.BigInteger hash, java.math.BigInteger r)` Constructs a new `Hash` object. |
  - ### Method Summary

    All Methods Instance Methods Concrete Methods

    | Modifier and Type | Method and Description |
    | `java.math.BigInteger` | `getHash()` Returns the hash. |
    | `java.math.BigInteger` | `getR()` Returns the parameter `r`. |

    - ### Methods inherited from class java.lang.Object

      `clone, equals, finalize, getClass, hashCode, notify, notifyAll, toString, wait, wait, wait`

- - ### Constructor Detail


    - #### Hash

      ```
      protected Hash(java.math.BigInteger hash,
                     java.math.BigInteger r)
      ```

      Constructs a new `Hash` object.

      Parameters:
      :   `hash` - hash
      :   `r` - parameter `r`
  - ### Method Detail


    - #### getHash

      ```
      public java.math.BigInteger getHash()
      ```

      Returns the hash.

      Returns:
      :   the hash


    - #### getR

      ```
      public java.math.BigInteger getR()
      ```

      Returns the parameter `r`.

      Returns:
      :   the parameter `r`


Skip navigation links


- Overview
- Package
- Class
- Use
- Tree
- Deprecated
- Index
- Help

- Prev Class
- Next Class

- Frames
- No Frames

- All Classes

- Summary:
- Nested |
- Field |
- Constr |
- Method

- Detail:
- Field |
- Constr |
- Method
