# Supplementary material for: A Blockchain Framework for Patient-Centered Health Records and Exchange (HealthChain): Evaluation and Proof-of-Concept Study
Source: J Med Internet Res. 2019 Aug 31;21(8):e13592. doi: 10.2196/13592 (PMC6743266; doi:10.2196/13592)
Supplement: Multimedia Appendix 3 [file jmir_v21i8e13592_app3.zip › ChameleonHashing/javadoc/edu/ecu/hsim/ray/chameleonhash/package-summary.html]

edu.ecu.hsim.ray.chameleonhash


JavaScript is disabled on your browser.


Skip navigation links


- Overview
- Package
- Class
- Use
- Tree
- Deprecated
- Index
- Help

- Prev Package
- Next Package

- Frames
- No Frames

- All Classes

# Package edu.ecu.hsim.ray.chameleonhash

- Class Summary

  | Class | Description |
  |  |  |
  | --- | --- |
  | ChameleonHash | Abstract Chameleon Hash class. |
  | Hash | Stores a message hash and parameter `r`. |
  | PublicCoinChameleonHash | Public-coin chameleon hash function as described in: G. |
  | PublicCoinHash | Hash container for `PublicCoinChameleonHash`. |
  | RSAChameleonHash | RSA-based chameleon hash function as described in Appendix A of: S. |
  | RSAHash | Hash container for `RSAChameleonHash`. |
- Enum Summary

  | Enum | Description |
  |  |  |
  | --- | --- |
  | ChameleonHash.STORAGE | Storage volatility options: Storage volatility refers how the generated keys will be stored and read. |

Skip navigation links


- Overview
- Package
- Class
- Use
- Tree
- Deprecated
- Index
- Help

- Prev Package
- Next Package

- Frames
- No Frames

- All Classes
