# Supplementary material for: A Blockchain Framework for Patient-Centered Health Records and Exchange (HealthChain): Evaluation and Proof-of-Concept Study
Source: J Med Internet Res. 2019 Aug 31;21(8):e13592. doi: 10.2196/13592 (PMC6743266; doi:10.2196/13592)
Supplement: Multimedia Appendix 3 [file jmir_v21i8e13592_app3.zip › ChameleonHashing/javadoc/edu/ecu/hsim/ray/chameleonhash/package-use.html]

Uses of Package edu.ecu.hsim.ray.chameleonhash


JavaScript is disabled on your browser.


Skip navigation links


- Overview
- Package
- Class
- Use
- Tree
- Deprecated
- Index
- Help

- Prev
- Next

- Frames
- No Frames

- All Classes

# Uses of Package edu.ecu.hsim.ray.chameleonhash

- Packages that use edu.ecu.hsim.ray.chameleonhash

  | Package | Description |
  |  |  |
  | --- | --- |
  | edu.ecu.hsim.ray.chameleonhash |  |
- Classes in edu.ecu.hsim.ray.chameleonhash used by edu.ecu.hsim.ray.chameleonhash

  | Class and Description |
  |  |
  | --- |
  | ChameleonHash Abstract Chameleon Hash class. |
  | ChameleonHash.STORAGE Storage volatility options: Storage volatility refers how the generated keys will be stored and read. |
  | Hash Stores a message hash and parameter `r`. |
  | PublicCoinHash Hash container for `PublicCoinChameleonHash`. |
  | RSAHash Hash container for `RSAChameleonHash`. |

Skip navigation links


- Overview
- Package
- Class
- Use
- Tree
- Deprecated
- Index
- Help

- Prev
- Next

- Frames
- No Frames

- All Classes
