# Supplementary material for: A Blockchain Framework for Patient-Centered Health Records and Exchange (HealthChain): Evaluation and Proof-of-Concept Study
Source: J Med Internet Res. 2019 Aug 31;21(8):e13592. doi: 10.2196/13592 (PMC6743266; doi:10.2196/13592)
Supplement: Multimedia Appendix 3 [file jmir_v21i8e13592_app3.zip › ChameleonHashing/javadoc/edu/ecu/hsim/ray/chameleonhash/RSAChameleonHash.html]

RSAChameleonHash


JavaScript is disabled on your browser.


Skip navigation links


- Overview
- Package
- Class
- Use
- Tree
- Deprecated
- Index
- Help

- Prev Class
- Next Class

- Frames
- No Frames

- All Classes

- Summary:
- Nested |
- Field |
- Constr |
- Method

- Detail:
- Field |
- Constr |
- Method


edu.ecu.hsim.ray.chameleonhash

## Class RSAChameleonHash

- java.lang.Object
- - edu.ecu.hsim.ray.chameleonhash.ChameleonHash
  - - edu.ecu.hsim.ray.chameleonhash.RSAChameleonHash

- ---

    

  ```
  public class RSAChameleonHash
  extends ChameleonHash
  ```

  RSA-based chameleon hash function as described in Appendix A of:
  > S. Hohenberger and B. Waters, "Realizing Hash-and-Sign Signatures under
  > Standard Assumptions," in Advances in Cryptology - EUROCRYPT 2009, 2009, pp.
  > 333–350. 
  > Paper.

  Of note: we do not constrain by `l` bits as suggested on page 20. This
  value affects the size of `e` and `H`, but has no impact on the
  output.

- - ### Nested Class Summary

    - ### Nested classes/interfaces inherited from class edu.ecu.hsim.ray.chameleonhash.ChameleonHash

      `ChameleonHash.STORAGE`
  - ### Field Summary

    - ### Fields inherited from class edu.ecu.hsim.ray.chameleonhash.ChameleonHash

      `createProperties, DEFAULT_BIT_LENGTH, ENCODING, fisPub, fisSec, fosPub, fosSec, N, one, p, phiN, pm1, propsPublic, propsSecret, publicFile, publicStringProperties, q, qm1, secretFile, secretStringProperties, sisPub, sisSec, sosPub, sosSec, storage, two, zero`
  - ### Constructor Summary

    Constructors

    | Constructor and Description |
    | `RSAChameleonHash()` Constructs a new `STORAGE#VOLATILE` RSA chameleon hash. |
    | `RSAChameleonHash(java.math.BigInteger p, java.math.BigInteger q)` Constructs a new `STORAGE#VOLATILE` RSA chameleon hash with the given primes. |
    | `RSAChameleonHash(java.math.BigInteger p, java.math.BigInteger q, java.lang.String file)` Constructs a new `STORAGE#NONVOLATILE` RSA chameleon hash with the given primes and stores it in `file`. |
    | `RSAChameleonHash(int bitLength)` Constructs a new `STORAGE#VOLATILE` RSA chameleon hash with hash key of length `bitLength`. |
    | `RSAChameleonHash(int bitLength, java.lang.String file)` Constructs a new `STORAGE#NONVOLATILE` RSA chameleon hash with hash key of length `bitLength` and stores it in `file`. |
    | `RSAChameleonHash(java.lang.String file)` Constructs a new `STORAGE#NONVOLATILE` RSA chameleon hash and stores it in `file`. |
    | `RSAChameleonHash(java.lang.String publicProperties, java.lang.String secretProperties)` Constructs a new `STORAGE#VOLATILE` RSA chameleon hash from existing `String`-based properties. |
  - ### Method Summary

    All Methods Instance Methods Concrete Methods

    | Modifier and Type | Method and Description |
    | `RSAHash` | `forge(byte[] message, Hash previousHash)` Forges the hash by creating new `r` and `s` parameters for the new message and prior hash. |
    | `RSAHash` | `forge(java.lang.String message, Hash previousHash)` Forges the hash by creating new `r` and `s` parameters for the new message and prior hash. |
    | `RSAHash` | `hash(byte[] message)` Hashes a message. |
    | `RSAHash` | `hash(java.lang.String message)` Hashes a message. |
    | `boolean` | `verify(byte[] message, Hash hash)` Verifies a message against a known `Hash`. |
    | `boolean` | `verify(java.lang.String message, Hash hash)` Verifies a message against a known `Hash`. |

    - ### Methods inherited from class edu.ecu.hsim.ray.chameleonhash.ChameleonHash

      `close, getPublicFile, getPublicProperties, getSecretFile, getSecretProperties, random, random`
    - ### Methods inherited from class java.lang.Object

      `clone, equals, finalize, getClass, hashCode, notify, notifyAll, toString, wait, wait, wait`

- - ### Constructor Detail


    - #### RSAChameleonHash

      ```
      public RSAChameleonHash()
                       throws java.io.IOException
      ```

      Constructs a new `STORAGE#VOLATILE` RSA chameleon hash.

      Throws:
      :   `java.io.IOException` - `IOException`


    - #### RSAChameleonHash

      ```
      public RSAChameleonHash(java.lang.String file)
                       throws java.io.IOException
      ```

      Constructs a new `STORAGE#NONVOLATILE` RSA chameleon hash and
      stores it in `file`. If `file` exists, then it reads in the
      parameters.

      Parameters:
      :   `file` - file name

      Throws:
      :   `java.io.IOException` - `IOException`


    - #### RSAChameleonHash

      ```
      public RSAChameleonHash(java.lang.String publicProperties,
                              java.lang.String secretProperties)
                       throws java.io.IOException
      ```

      Constructs a new `STORAGE#VOLATILE` RSA chameleon hash from
      existing `String`-based properties.

      Parameters:
      :   `publicProperties` - public properties in `String` form
      :   `secretProperties` - secret properties in `String` form

      Throws:
      :   `java.io.IOException` - `IOException`


    - #### RSAChameleonHash

      ```
      public RSAChameleonHash(int bitLength)
                       throws java.io.IOException
      ```

      Constructs a new `STORAGE#VOLATILE` RSA chameleon hash with hash
      key of length `bitLength`.

      Parameters:
      :   `bitLength` - length of the

      Throws:
      :   `java.io.IOException` - `IOException`


    - #### RSAChameleonHash

      ```
      public RSAChameleonHash(int bitLength,
                              java.lang.String file)
                       throws java.io.IOException
      ```

      Constructs a new `STORAGE#NONVOLATILE` RSA chameleon hash with hash
      key of length `bitLength` and stores it in `file`. If
      `file` exists, then it reads in the parameters, ignoring what is
      passed.

      Parameters:
      :   `bitLength` - length of the
      :   `file` - file name

      Throws:
      :   `java.io.IOException` - `IOException`


    - #### RSAChameleonHash

      ```
      public RSAChameleonHash(java.math.BigInteger p,
                              java.math.BigInteger q)
                       throws java.io.IOException
      ```

      Constructs a new `STORAGE#VOLATILE` RSA chameleon hash with the
      given primes. Note: `p = 2q-1`.

      Parameters:
      :   `p` - prime p
      :   `q` - prime q

      Throws:
      :   `java.io.IOException` - `IOException`


    - #### RSAChameleonHash

      ```
      public RSAChameleonHash(java.math.BigInteger p,
                              java.math.BigInteger q,
                              java.lang.String file)
                       throws java.io.IOException
      ```

      Constructs a new `STORAGE#NONVOLATILE` RSA chameleon hash with the
      given primes and stores it in `file`. If `file` exists, then
      it reads in the parameters, ignoring what is passed. Note:
      `p = 2q-1`.

      Parameters:
      :   `p` - prime p
      :   `q` - prime q
      :   `file` - file name

      Throws:
      :   `java.io.IOException` - `IOException`
  - ### Method Detail


    - #### hash

      ```
      public RSAHash hash(java.lang.String message)
      ```

      Description copied from class: `ChameleonHash`

      Hashes a message.

      Specified by:
      :   `hash` in class `ChameleonHash`

      Parameters:
      :   `message` - message to hash

      Returns:
      :   `Hash` of message


    - #### hash

      ```
      public RSAHash hash(byte[] message)
      ```

      Description copied from class: `ChameleonHash`

      Hashes a message.

      Specified by:
      :   `hash` in class `ChameleonHash`

      Parameters:
      :   `message` - message to hash

      Returns:
      :   `Hash` of message


    - #### verify

      ```
      public boolean verify(java.lang.String message,
                            Hash hash)
      ```

      Description copied from class: `ChameleonHash`

      Verifies a message against a known `Hash`.

      Specified by:
      :   `verify` in class `ChameleonHash`

      Parameters:
      :   `message` - message to verify
      :   `hash` - `Hash`

      Returns:
      :   `true` if verified, `false` otherwise


    - #### verify

      ```
      public boolean verify(byte[] message,
                            Hash hash)
      ```

      Description copied from class: `ChameleonHash`

      Verifies a message against a known `Hash`.

      Specified by:
      :   `verify` in class `ChameleonHash`

      Parameters:
      :   `message` - message to verify
      :   `hash` - `Hash`

      Returns:
      :   `true` if verified, `false` otherwise


    - #### forge

      ```
      public RSAHash forge(java.lang.String message,
                           Hash previousHash)
      ```

      Description copied from class: `ChameleonHash`

      Forges the hash by creating new `r` and `s` parameters for
      the new message and prior hash.

      Specified by:
      :   `forge` in class `ChameleonHash`

      Parameters:
      :   `message` - message
      :   `previousHash` - previous `Hash`

      Returns:
      :   the forged `Hash`


    - #### forge

      ```
      public RSAHash forge(byte[] message,
                           Hash previousHash)
      ```

      Description copied from class: `ChameleonHash`

      Forges the hash by creating new `r` and `s` parameters for
      the new message and prior hash.

      Specified by:
      :   `forge` in class `ChameleonHash`

      Parameters:
      :   `message` - message
      :   `previousHash` - previous `Hash`

      Returns:
      :   the forged `Hash`


Skip navigation links


- Overview
- Package
- Class
- Use
- Tree
- Deprecated
- Index
- Help

- Prev Class
- Next Class

- Frames
- No Frames

- All Classes

- Summary:
- Nested |
- Field |
- Constr |
- Method

- Detail:
- Field |
- Constr |
- Method
