# Supplementary material for: A Blockchain Framework for Patient-Centered Health Records and Exchange (HealthChain): Evaluation and Proof-of-Concept Study
Source: J Med Internet Res. 2019 Aug 31;21(8):e13592. doi: 10.2196/13592 (PMC6743266; doi:10.2196/13592)
Supplement: Multimedia Appendix 3 [file jmir_v21i8e13592_app3.zip › ChameleonHashing/javadoc/edu/ecu/hsim/ray/messagedigest/MessageDigest.Algorithms.html]

MessageDigest.Algorithms


JavaScript is disabled on your browser.


Skip navigation links


- Overview
- Package
- Class
- Use
- Tree
- Deprecated
- Index
- Help

- Prev Class
- Next Class

- Frames
- No Frames

- All Classes

- Summary:
- Nested |
- Enum Constants |
- Field |
- Method

- Detail:
- Enum Constants |
- Field |
- Method


edu.ecu.hsim.ray.messagedigest

## Enum MessageDigest.Algorithms

- java.lang.Object
- - java.lang.Enum<MessageDigest.Algorithms>
  - - edu.ecu.hsim.ray.messagedigest.MessageDigest.Algorithms

- All Implemented Interfaces:
  :   java.io.Serializable, java.lang.Comparable<MessageDigest.Algorithms>

  Enclosing class:
  :   MessageDigest

  ---

    

  ```
  public static enum MessageDigest.Algorithms
  extends java.lang.Enum<MessageDigest.Algorithms>
  ```

  Java 7+ compliant message digest algorithms:
  - Java 7:
    `MD2`, `MD5`, `SHA-1`, `SHA-256`, `SHA-384`, `SHA-512`
  - Java 8:
    `MD2`, `MD5`, `SHA-1`, `SHA-224`, `SHA-256`, `SHA-384`, `SHA-512`Algorithm details:
  - `MD2` (**BROKEN**) - 128 bit hash, arbitrarily large input (RFC 1319)
  - `MD5` (**BROKEN**) - 128 bit hash, arbitrarily large input (RFC 1321)
  - `SHA-1`\* (**BROKEN**) - 160 bit hash, hashes up to first 264-1 bits
  - `SHA-224`\* - SHA-2 standard, 224 bit hash, 264-1 bits ~ 2 exabytes (2\*10246)
  - `SHA-256`\* - SHA-2 standard, 256 bit hash, 264-1 bits ~ 2 exabytes (2\*10246)
  - `SHA-384`\* - SHA-2 standard, 384 bit hash, 2128-1 bits ~ 35 trillion yottabytes (32\*102412)
  - `SHA-512`\* (default) - SHA-2 standard, 512 bit hash, 2128-1 bits ~ 35 trillion yottabytes (32\*102412)
  - \*FIPS PUB 180-4, Secure Hash Standard, NSA

  Note: JEP 287 SHA-3 Hash Algorithms
  is slated for release in JDK 9. Implements NIST FIPS 202,
  which defines the current NIST approved hash algorithms.

- - ### Enum Constant Summary

    Enum Constants

    | Enum Constant and Description |
    | `MD2` |
    | `MD5` |
    | `SHA1` |
    | `SHA224` |
    | `SHA256` |
    | `SHA384` |
    | `SHA512` |
  - ### Method Summary

    All Methods Static Methods Concrete Methods

    | Modifier and Type | Method and Description |
    | `static MessageDigest.Algorithms` | `valueOf(java.lang.String name)` Returns the enum constant of this type with the specified name. |
    | `static MessageDigest.Algorithms[]` | `values()` Returns an array containing the constants of this enum type, in the order they are declared. |

    - ### Methods inherited from class java.lang.Enum

      `clone, compareTo, equals, finalize, getDeclaringClass, hashCode, name, ordinal, toString, valueOf`
    - ### Methods inherited from class java.lang.Object

      `getClass, notify, notifyAll, wait, wait, wait`

- - ### Enum Constant Detail


    - #### MD2

      ```
      public static final MessageDigest.Algorithms MD2
      ```


    - #### MD5

      ```
      public static final MessageDigest.Algorithms MD5
      ```


    - #### SHA1

      ```
      public static final MessageDigest.Algorithms SHA1
      ```


    - #### SHA224

      ```
      public static final MessageDigest.Algorithms SHA224
      ```


    - #### SHA256

      ```
      public static final MessageDigest.Algorithms SHA256
      ```


    - #### SHA384

      ```
      public static final MessageDigest.Algorithms SHA384
      ```


    - #### SHA512

      ```
      public static final MessageDigest.Algorithms SHA512
      ```
  - ### Method Detail


    - #### values

      ```
      public static MessageDigest.Algorithms[] values()
      ```

      Returns an array containing the constants of this enum type, in
      the order they are declared. This method may be used to iterate
      over the constants as follows:

      ```
      for (MessageDigest.Algorithms c : MessageDigest.Algorithms.values())
          System.out.println(c);
      ```

      Returns:
      :   an array containing the constants of this enum type, in the order they are declared


    - #### valueOf

      ```
      public static MessageDigest.Algorithms valueOf(java.lang.String name)
      ```

      Returns the enum constant of this type with the specified name.
      The string must match *exactly* an identifier used to declare an
      enum constant in this type. (Extraneous whitespace characters are
      not permitted.)

      Parameters:
      :   `name` - the name of the enum constant to be returned.

      Returns:
      :   the enum constant with the specified name

      Throws:
      :   `java.lang.IllegalArgumentException` - if this enum type has no constant with the specified name
      :   `java.lang.NullPointerException` - if the argument is null


Skip navigation links


- Overview
- Package
- Class
- Use
- Tree
- Deprecated
- Index
- Help

- Prev Class
- Next Class

- Frames
- No Frames

- All Classes

- Summary:
- Nested |
- Enum Constants |
- Field |
- Method

- Detail:
- Enum Constants |
- Field |
- Method
