# Supplementary material for: A Blockchain Framework for Patient-Centered Health Records and Exchange (HealthChain): Evaluation and Proof-of-Concept Study
Source: J Med Internet Res. 2019 Aug 31;21(8):e13592. doi: 10.2196/13592 (PMC6743266; doi:10.2196/13592)
Supplement: Multimedia Appendix 3 [file jmir_v21i8e13592_app3.zip › ChameleonHashing/javadoc/edu/ecu/hsim/ray/messagedigest/MessageDigest.html]

MessageDigest


JavaScript is disabled on your browser.


Skip navigation links


- Overview
- Package
- Class
- Use
- Tree
- Deprecated
- Index
- Help

- Prev Class
- Next Class

- Frames
- No Frames

- All Classes

- Summary:
- Nested |
- Field |
- Constr |
- Method

- Detail:
- Field |
- Constr |
- Method


edu.ecu.hsim.ray.messagedigest

## Class MessageDigest

- java.lang.Object
- - edu.ecu.hsim.ray.messagedigest.MessageDigest

- ---

    

  ```
  public class MessageDigest
  extends java.lang.Object
  ```

  Simple message digest class with various options - see `MessageDigest.Algorithms`.

- - ### Nested Class Summary

    Nested Classes

    | Modifier and Type | Class and Description |
    | `static class` | `MessageDigest.Algorithms` Java 7+ compliant message digest algorithms: Java 7: `MD2`, `MD5`, `SHA-1`, `SHA-256`, `SHA-384`, `SHA-512` Java 8: `MD2`, `MD5`, `SHA-1`, `SHA-224`, `SHA-256`, `SHA-384`, `SHA-512` Algorithm details: `MD2` (**BROKEN**) - 128 bit hash, arbitrarily large input (RFC 1319) `MD5` (**BROKEN**) - 128 bit hash, arbitrarily large input (RFC 1321) `SHA-1`\* (**BROKEN**) - 160 bit hash, hashes up to first 264-1 bits `SHA-224`\* - SHA-2 standard, 224 bit hash, 264-1 bits ~ 2 exabytes (2\*10246) `SHA-256`\* - SHA-2 standard, 256 bit hash, 264-1 bits ~ 2 exabytes (2\*10246) `SHA-384`\* - SHA-2 standard, 384 bit hash, 2128-1 bits ~ 35 trillion yottabytes (32\*102412) `SHA-512`\* (default) - SHA-2 standard, 512 bit hash, 2128-1 bits ~ 35 trillion yottabytes (32\*102412)- \*FIPS PUB 180-4, Secure Hash Standard, NSA |
  - ### Constructor Summary

    Constructors

    | Constructor and Description |
    | `MessageDigest()` |
  - ### Method Summary

    All Methods Instance Methods Concrete Methods

    | Modifier and Type | Method and Description |
    | `byte[]` | `hash(byte[] bytes)` Returns the message digest given the file. |
    | `byte[]` | `hash(MessageDigest.Algorithms algorithm, byte[] bytes)` Returns the message digest given the file. |

    - ### Methods inherited from class java.lang.Object

      `clone, equals, finalize, getClass, hashCode, notify, notifyAll, toString, wait, wait, wait`

- - ### Constructor Detail


    - #### MessageDigest

      ```
      public MessageDigest()
      ```
  - ### Method Detail


    - #### hash

      ```
      public byte[] hash(byte[] bytes)
                  throws java.io.IOException,
                         java.security.NoSuchAlgorithmException
      ```

      Returns the message digest given the file.

      Parameters:
      :   `bytes` - bytes to hash

      Returns:
      :   message digest as `byte[]` or `null` if any
          parameters are `null` or empty

      Throws:
      :   `java.io.IOException` - `IOException`
      :   `java.security.NoSuchAlgorithmException` - `NoSuchAlgorithmException`


    - #### hash

      ```
      public byte[] hash(MessageDigest.Algorithms algorithm,
                         byte[] bytes)
                  throws java.io.IOException,
                         java.security.NoSuchAlgorithmException
      ```

      Returns the message digest given the file.

      Parameters:
      :   `algorithm` - hashing algorithm from `MessageDigest.Algorithms`
      :   `bytes` - bytes to hash

      Returns:
      :   message digest as `byte[]` or `null` if any
          parameters are `null` or empty

      Throws:
      :   `java.io.IOException` - `IOException`
      :   `java.security.NoSuchAlgorithmException` - `NoSuchAlgorithmException`


Skip navigation links


- Overview
- Package
- Class
- Use
- Tree
- Deprecated
- Index
- Help

- Prev Class
- Next Class

- Frames
- No Frames

- All Classes

- Summary:
- Nested |
- Field |
- Constr |
- Method

- Detail:
- Field |
- Constr |
- Method
