# Supplementary material for: A Blockchain Framework for Patient-Centered Health Records and Exchange (HealthChain): Evaluation and Proof-of-Concept Study
Source: J Med Internet Res. 2019 Aug 31;21(8):e13592. doi: 10.2196/13592 (PMC6743266; doi:10.2196/13592)
Supplement: Multimedia Appendix 3 [file jmir_v21i8e13592_app3.zip › ChameleonHashing/javadoc/index-files/index-1.html]

C-Index


JavaScript is disabled on your browser.


Skip navigation links


- Overview
- Package
- Class
- Use
- Tree
- Deprecated
- Index
- Help

- Prev Letter
- Next Letter

- Frames
- No Frames

- All Classes

C D E F G H M N O P Q R S T V Z 


## C

ChameleonHash - Class in edu.ecu.hsim.ray.chameleonhash
:   Abstract Chameleon Hash class.

ChameleonHash(BigInteger, BigInteger, int, String) - Constructor for class edu.ecu.hsim.ray.chameleonhash.ChameleonHash
:   Constructor.

ChameleonHash(BigInteger, BigInteger, int, String, String) - Constructor for class edu.ecu.hsim.ray.chameleonhash.ChameleonHash
:   Constructor.

ChameleonHash.STORAGE - Enum in edu.ecu.hsim.ray.chameleonhash
:   Storage volatility options:
    Storage volatility refers how the generated keys will be stored and read.

close() - Method in class edu.ecu.hsim.ray.chameleonhash.ChameleonHash
:   Closes the file input and output streams.

createProperties - Variable in class edu.ecu.hsim.ray.chameleonhash.ChameleonHash
:   Create properties file `boolean` parameter.

C D E F G H M N O P Q R S T V Z

Skip navigation links


- Overview
- Package
- Class
- Use
- Tree
- Deprecated
- Index
- Help

- Prev Letter
- Next Letter

- Frames
- No Frames

- All Classes
