# Supplementary material for: A Blockchain Framework for Patient-Centered Health Records and Exchange (HealthChain): Evaluation and Proof-of-Concept Study
Source: J Med Internet Res. 2019 Aug 31;21(8):e13592. doi: 10.2196/13592 (PMC6743266; doi:10.2196/13592)
Supplement: Multimedia Appendix 3 [file jmir_v21i8e13592_app3.zip › ChameleonHashing/javadoc/index-files/index-10.html]

P-Index


JavaScript is disabled on your browser.


Skip navigation links


- Overview
- Package
- Class
- Use
- Tree
- Deprecated
- Index
- Help

- Prev Letter
- Next Letter

- Frames
- No Frames

- All Classes

C D E F G H M N O P Q R S T V Z 


## P

p - Variable in class edu.ecu.hsim.ray.chameleonhash.ChameleonHash
:   `p = 2q+1`.

phiN - Variable in class edu.ecu.hsim.ray.chameleonhash.ChameleonHash
:   Phi of `N`.

pm1 - Variable in class edu.ecu.hsim.ray.chameleonhash.ChameleonHash
:   .

propsPublic - Variable in class edu.ecu.hsim.ray.chameleonhash.ChameleonHash
:   Public `Properties` file.

propsSecret - Variable in class edu.ecu.hsim.ray.chameleonhash.ChameleonHash
:   Secret `Properties` file.

PublicCoinChameleonHash - Class in edu.ecu.hsim.ray.chameleonhash
:   Public-coin chameleon hash function as described in:
    G.

PublicCoinChameleonHash() - Constructor for class edu.ecu.hsim.ray.chameleonhash.PublicCoinChameleonHash
:   Constructs a new `STORAGE#VOLATILE` public-coin chameleon hash.

PublicCoinChameleonHash(String) - Constructor for class edu.ecu.hsim.ray.chameleonhash.PublicCoinChameleonHash
:   Constructs a new `STORAGE#NONVOLATILE` public-coin chameleon hash
    and stores it in `file`.

PublicCoinChameleonHash(byte[], byte[]) - Constructor for class edu.ecu.hsim.ray.chameleonhash.PublicCoinChameleonHash
:   Constructs a new `STORAGE#VOLATILE` public-coin chameleon hash from
    existing `String`-based properties.

PublicCoinChameleonHash(String, String) - Constructor for class edu.ecu.hsim.ray.chameleonhash.PublicCoinChameleonHash
:   Constructs a new `STORAGE#VOLATILE` public-coin chameleon hash from
    existing `String`-based properties.

PublicCoinChameleonHash(int) - Constructor for class edu.ecu.hsim.ray.chameleonhash.PublicCoinChameleonHash
:   Constructs a new `STORAGE#VOLATILE` public-coin chameleon hash with
    hash key of length `bitLength`.

PublicCoinChameleonHash(int, String) - Constructor for class edu.ecu.hsim.ray.chameleonhash.PublicCoinChameleonHash
:   Constructs a new `STORAGE#NONVOLATILE` public-coin chameleon hash
    with hash key of length `bitLength` and stores it in `file`.

PublicCoinChameleonHash(BigInteger, BigInteger) - Constructor for class edu.ecu.hsim.ray.chameleonhash.PublicCoinChameleonHash
:   Constructs a new `STORAGE#VOLATILE` public-coin chameleon hash with
    the given primes.

PublicCoinChameleonHash(BigInteger, BigInteger, String) - Constructor for class edu.ecu.hsim.ray.chameleonhash.PublicCoinChameleonHash
:   Constructs a new `STORAGE#NONVOLATILE` public-coin chameleon hash
    with the given primes and stores it in `file`.

PublicCoinHash - Class in edu.ecu.hsim.ray.chameleonhash
:   Hash container for `PublicCoinChameleonHash`.

PublicCoinHash(BigInteger, BigInteger, BigInteger) - Constructor for class edu.ecu.hsim.ray.chameleonhash.PublicCoinHash
:   Constructs a new `Hash` object.

publicFile - Variable in class edu.ecu.hsim.ray.chameleonhash.ChameleonHash
:   Public properties `File`.

publicStringProperties - Variable in class edu.ecu.hsim.ray.chameleonhash.ChameleonHash
:   Public properties as `String`.

C D E F G H M N O P Q R S T V Z

Skip navigation links


- Overview
- Package
- Class
- Use
- Tree
- Deprecated
- Index
- Help

- Prev Letter
- Next Letter

- Frames
- No Frames

- All Classes
