# Supplementary material for: A Blockchain Framework for Patient-Centered Health Records and Exchange (HealthChain): Evaluation and Proof-of-Concept Study
Source: J Med Internet Res. 2019 Aug 31;21(8):e13592. doi: 10.2196/13592 (PMC6743266; doi:10.2196/13592)
Supplement: Multimedia Appendix 3 [file jmir_v21i8e13592_app3.zip › ChameleonHashing/javadoc/index-files/index-12.html]

R-Index


JavaScript is disabled on your browser.


Skip navigation links


- Overview
- Package
- Class
- Use
- Tree
- Deprecated
- Index
- Help

- Prev Letter
- Next Letter

- Frames
- No Frames

- All Classes

C D E F G H M N O P Q R S T V Z 


## R

random(BigInteger) - Method in class edu.ecu.hsim.ray.chameleonhash.ChameleonHash
:   Generates a random `BigInteger` with upper bound.

random(BigInteger, BigInteger) - Method in class edu.ecu.hsim.ray.chameleonhash.ChameleonHash
:   Generates a random `BigInteger` with upper and lower bounds.

RSAChameleonHash - Class in edu.ecu.hsim.ray.chameleonhash
:   RSA-based chameleon hash function as described in Appendix A of:
    S.

RSAChameleonHash() - Constructor for class edu.ecu.hsim.ray.chameleonhash.RSAChameleonHash
:   Constructs a new `STORAGE#VOLATILE` RSA chameleon hash.

RSAChameleonHash(String) - Constructor for class edu.ecu.hsim.ray.chameleonhash.RSAChameleonHash
:   Constructs a new `STORAGE#NONVOLATILE` RSA chameleon hash and
    stores it in `file`.

RSAChameleonHash(String, String) - Constructor for class edu.ecu.hsim.ray.chameleonhash.RSAChameleonHash
:   Constructs a new `STORAGE#VOLATILE` RSA chameleon hash from
    existing `String`-based properties.

RSAChameleonHash(int) - Constructor for class edu.ecu.hsim.ray.chameleonhash.RSAChameleonHash
:   Constructs a new `STORAGE#VOLATILE` RSA chameleon hash with hash
    key of length `bitLength`.

RSAChameleonHash(int, String) - Constructor for class edu.ecu.hsim.ray.chameleonhash.RSAChameleonHash
:   Constructs a new `STORAGE#NONVOLATILE` RSA chameleon hash with hash
    key of length `bitLength` and stores it in `file`.

RSAChameleonHash(BigInteger, BigInteger) - Constructor for class edu.ecu.hsim.ray.chameleonhash.RSAChameleonHash
:   Constructs a new `STORAGE#VOLATILE` RSA chameleon hash with the
    given primes.

RSAChameleonHash(BigInteger, BigInteger, String) - Constructor for class edu.ecu.hsim.ray.chameleonhash.RSAChameleonHash
:   Constructs a new `STORAGE#NONVOLATILE` RSA chameleon hash with the
    given primes and stores it in `file`.

RSAHash - Class in edu.ecu.hsim.ray.chameleonhash
:   Hash container for `RSAChameleonHash`.

RSAHash(BigInteger, BigInteger, BigInteger, BigInteger, BigInteger, BigInteger) - Constructor for class edu.ecu.hsim.ray.chameleonhash.RSAHash
:   Constructs a new `Hash` object.

C D E F G H M N O P Q R S T V Z

Skip navigation links


- Overview
- Package
- Class
- Use
- Tree
- Deprecated
- Index
- Help

- Prev Letter
- Next Letter

- Frames
- No Frames

- All Classes
