# Supplementary material for: A Blockchain Framework for Patient-Centered Health Records and Exchange (HealthChain): Evaluation and Proof-of-Concept Study
Source: J Med Internet Res. 2019 Aug 31;21(8):e13592. doi: 10.2196/13592 (PMC6743266; doi:10.2196/13592)
Supplement: Multimedia Appendix 3 [file jmir_v21i8e13592_app3.zip › ChameleonHashing/javadoc/index-files/index-13.html]

S-Index


JavaScript is disabled on your browser.


Skip navigation links


- Overview
- Package
- Class
- Use
- Tree
- Deprecated
- Index
- Help

- Prev Letter
- Next Letter

- Frames
- No Frames

- All Classes

C D E F G H M N O P Q R S T V Z 


## S

secretFile - Variable in class edu.ecu.hsim.ray.chameleonhash.ChameleonHash
:   Secret properties `File`.

secretStringProperties - Variable in class edu.ecu.hsim.ray.chameleonhash.ChameleonHash
:   Secret properties as `String`.

sisPub - Variable in class edu.ecu.hsim.ray.chameleonhash.ChameleonHash
:   Public properties `StringReader`.

sisSec - Variable in class edu.ecu.hsim.ray.chameleonhash.ChameleonHash
:   Secret properties `StringReader`.

sosPub - Variable in class edu.ecu.hsim.ray.chameleonhash.ChameleonHash
:   Public properties `StringWriter`.

sosSec - Variable in class edu.ecu.hsim.ray.chameleonhash.ChameleonHash
:   Secret properties `StringWriter`.

storage - Variable in class edu.ecu.hsim.ray.chameleonhash.ChameleonHash
:   `ChameleonHash.STORAGE` parameter.

C D E F G H M N O P Q R S T V Z

Skip navigation links


- Overview
- Package
- Class
- Use
- Tree
- Deprecated
- Index
- Help

- Prev Letter
- Next Letter

- Frames
- No Frames

- All Classes
