# Supplementary material for: A Blockchain Framework for Patient-Centered Health Records and Exchange (HealthChain): Evaluation and Proof-of-Concept Study
Source: J Med Internet Res. 2019 Aug 31;21(8):e13592. doi: 10.2196/13592 (PMC6743266; doi:10.2196/13592)
Supplement: Multimedia Appendix 3 [file jmir_v21i8e13592_app3.zip › ChameleonHashing/javadoc/index-files/index-5.html]

G-Index


JavaScript is disabled on your browser.


Skip navigation links


- Overview
- Package
- Class
- Use
- Tree
- Deprecated
- Index
- Help

- Prev Letter
- Next Letter

- Frames
- No Frames

- All Classes

C D E F G H M N O P Q R S T V Z 


## G

getE() - Method in class edu.ecu.hsim.ray.chameleonhash.RSAHash
:   Returns the parameter `e`.

getHash() - Method in class edu.ecu.hsim.ray.chameleonhash.Hash
:   Returns the hash.

getJ() - Method in class edu.ecu.hsim.ray.chameleonhash.RSAHash
:   Returns the parameter `J`.

getM() - Method in class edu.ecu.hsim.ray.chameleonhash.RSAHash
:   Returns the message `m`.

getN() - Method in class edu.ecu.hsim.ray.chameleonhash.RSAHash
:   Returns the parameter `N`.

getPublicFile() - Method in class edu.ecu.hsim.ray.chameleonhash.ChameleonHash
:   Returns the public file if `ChameleonHash.storage` ==
    `ChameleonHash.STORAGE.NONVOLATILE`.

getPublicProperties() - Method in class edu.ecu.hsim.ray.chameleonhash.ChameleonHash
:   Returns public properties as a `String`.

getR() - Method in class edu.ecu.hsim.ray.chameleonhash.Hash
:   Returns the parameter `r`.

getS() - Method in class edu.ecu.hsim.ray.chameleonhash.PublicCoinHash
:   Returns the parameter `s`.

getSecretFile() - Method in class edu.ecu.hsim.ray.chameleonhash.ChameleonHash
:   Returns the secret file if `ChameleonHash.storage` ==
    `ChameleonHash.STORAGE.NONVOLATILE`.

getSecretProperties() - Method in class edu.ecu.hsim.ray.chameleonhash.ChameleonHash
:   Returns secret properties as a `String`.

C D E F G H M N O P Q R S T V Z

Skip navigation links


- Overview
- Package
- Class
- Use
- Tree
- Deprecated
- Index
- Help

- Prev Letter
- Next Letter

- Frames
- No Frames

- All Classes
