# Supplementary material for: A Blockchain Framework for Patient-Centered Health Records and Exchange (HealthChain): Evaluation and Proof-of-Concept Study
Source: J Med Internet Res. 2019 Aug 31;21(8):e13592. doi: 10.2196/13592 (PMC6743266; doi:10.2196/13592)
Supplement: Multimedia Appendix 3 [file jmir_v21i8e13592_app3.zip › ChameleonHashing/javadoc/index-files/index-6.html]

H-Index


JavaScript is disabled on your browser.


Skip navigation links


- Overview
- Package
- Class
- Use
- Tree
- Deprecated
- Index
- Help

- Prev Letter
- Next Letter

- Frames
- No Frames

- All Classes

C D E F G H M N O P Q R S T V Z 


## H

hash(String) - Method in class edu.ecu.hsim.ray.chameleonhash.ChameleonHash
:   Hashes a message.

hash(byte[]) - Method in class edu.ecu.hsim.ray.chameleonhash.ChameleonHash
:   Hashes a message.

Hash - Class in edu.ecu.hsim.ray.chameleonhash
:   Stores a message hash and parameter `r`.

Hash(BigInteger, BigInteger) - Constructor for class edu.ecu.hsim.ray.chameleonhash.Hash
:   Constructs a new `Hash` object.

hash(String) - Method in class edu.ecu.hsim.ray.chameleonhash.PublicCoinChameleonHash


hash(byte[]) - Method in class edu.ecu.hsim.ray.chameleonhash.PublicCoinChameleonHash


hash(String) - Method in class edu.ecu.hsim.ray.chameleonhash.RSAChameleonHash


hash(byte[]) - Method in class edu.ecu.hsim.ray.chameleonhash.RSAChameleonHash


hash(byte[]) - Method in class edu.ecu.hsim.ray.messagedigest.MessageDigest
:   Returns the message digest given the file.

hash(MessageDigest.Algorithms, byte[]) - Method in class edu.ecu.hsim.ray.messagedigest.MessageDigest
:   Returns the message digest given the file.

C D E F G H M N O P Q R S T V Z

Skip navigation links


- Overview
- Package
- Class
- Use
- Tree
- Deprecated
- Index
- Help

- Prev Letter
- Next Letter

- Frames
- No Frames

- All Classes
