# Supplementary material for: A Blockchain Framework for Patient-Centered Health Records and Exchange (HealthChain): Evaluation and Proof-of-Concept Study
Source: J Med Internet Res. 2019 Aug 31;21(8):e13592. doi: 10.2196/13592 (PMC6743266; doi:10.2196/13592)
Supplement: Multimedia Appendix 3 [file jmir_v21i8e13592_app3.zip › ChameleonHashing/javadoc/index-files/index-7.html]

M-Index


JavaScript is disabled on your browser.


Skip navigation links


- Overview
- Package
- Class
- Use
- Tree
- Deprecated
- Index
- Help

- Prev Letter
- Next Letter

- Frames
- No Frames

- All Classes

C D E F G H M N O P Q R S T V Z 


## M

MessageDigest - Class in edu.ecu.hsim.ray.messagedigest
:   Simple message digest class with various options - see `MessageDigest.Algorithms`.

MessageDigest() - Constructor for class edu.ecu.hsim.ray.messagedigest.MessageDigest


MessageDigest.Algorithms - Enum in edu.ecu.hsim.ray.messagedigest
:   Java 7+ compliant message digest algorithms:
    Java 7:
    `MD2`, `MD5`, `SHA-1`, `SHA-256`, `SHA-384`, `SHA-512`
    Java 8:
    `MD2`, `MD5`, `SHA-1`, `SHA-224`, `SHA-256`, `SHA-384`, `SHA-512`
    Algorithm details:
    `MD2` (**BROKEN**) - 128 bit hash, arbitrarily large input (RFC 1319)
    `MD5` (**BROKEN**) - 128 bit hash, arbitrarily large input (RFC 1321)
    `SHA-1`\* (**BROKEN**) - 160 bit hash, hashes up to first 264-1 bits
    `SHA-224`\* - SHA-2 standard, 224 bit hash, 264-1 bits ~ 2 exabytes (2\*10246)
    `SHA-256`\* - SHA-2 standard, 256 bit hash, 264-1 bits ~ 2 exabytes (2\*10246)
    `SHA-384`\* - SHA-2 standard, 384 bit hash, 2128-1 bits ~ 35 trillion yottabytes (32\*102412)
    `SHA-512`\* (default) - SHA-2 standard, 512 bit hash, 2128-1 bits ~ 35 trillion yottabytes (32\*102412)- \*FIPS PUB 180-4, Secure Hash Standard, NSA

C D E F G H M N O P Q R S T V Z

Skip navigation links


- Overview
- Package
- Class
- Use
- Tree
- Deprecated
- Index
- Help

- Prev Letter
- Next Letter

- Frames
- No Frames

- All Classes
