# Supplementary material for: A Blockchain Framework for Patient-Centered Health Records and Exchange (HealthChain): Evaluation and Proof-of-Concept Study
Source: J Med Internet Res. 2019 Aug 31;21(8):e13592. doi: 10.2196/13592 (PMC6743266; doi:10.2196/13592)
Supplement: Multimedia Appendix 3 [file jmir_v21i8e13592_app3.zip › ChameleonHashing/javadoc/overview-tree.html]

Class Hierarchy


JavaScript is disabled on your browser.


Skip navigation links


- Overview
- Package
- Class
- Use
- Tree
- Deprecated
- Index
- Help

- Prev
- Next

- Frames
- No Frames

- All Classes

# Hierarchy For All Packages

Package Hierarchies:

- edu.ecu.hsim.ray.chameleonhash,
- edu.ecu.hsim.ray.messagedigest

## Class Hierarchy

- java.lang.Object
  - edu.ecu.hsim.ray.chameleonhash.ChameleonHash
    - edu.ecu.hsim.ray.chameleonhash.PublicCoinChameleonHash
    - edu.ecu.hsim.ray.chameleonhash.RSAChameleonHash
  - edu.ecu.hsim.ray.chameleonhash.Hash
    - edu.ecu.hsim.ray.chameleonhash.PublicCoinHash
    - edu.ecu.hsim.ray.chameleonhash.RSAHash
  - edu.ecu.hsim.ray.messagedigest.MessageDigest

## Enum Hierarchy

- java.lang.Object
  - java.lang.Enum<E> (implements java.lang.Comparable<T>, java.io.Serializable)
    - edu.ecu.hsim.ray.chameleonhash.ChameleonHash.STORAGE
    - edu.ecu.hsim.ray.messagedigest.MessageDigest.Algorithms

Skip navigation links


- Overview
- Package
- Class
- Use
- Tree
- Deprecated
- Index
- Help

- Prev
- Next

- Frames
- No Frames

- All Classes
