# Supplementary material for: A Blockchain Framework for Patient-Centered Health Records and Exchange (HealthChain): Evaluation and Proof-of-Concept Study
Source: J Med Internet Res. 2019 Aug 31;21(8):e13592. doi: 10.2196/13592 (PMC6743266; doi:10.2196/13592)
Supplement: Multimedia Appendix 3 [file jmir_v21i8e13592_app3.zip › HealthChainWebClient/web/calendar.html]

Gentelella Alela! | 


### Calendar Click to add/edit events

Go!

## Calendar Events Sessions

- - Settings 1
  - Settings 2

Ã—

#### New Calendar Entry

Title

Description

Close
Save changes

Ã—

#### Edit Calendar Entry

Title

Description

Close
Save changes
