# Supplementary material for: A Blockchain Framework for Patient-Centered Health Records and Exchange (HealthChain): Evaluation and Proof-of-Concept Study
Source: J Med Internet Res. 2019 Aug 31;21(8):e13592. doi: 10.2196/13592 (PMC6743266; doi:10.2196/13592)
Supplement: Multimedia Appendix 3 [file jmir_v21i8e13592_app3.zip › HealthChainWebClient/web/page_500.html]

Gentallela Alela! | 


# 500

## Internal Server Error

We track these errors automatically, but if the problem persists feel free to contact us. In the meantime, try refreshing. Report this?

### Search

Go!
