# Supplementary material for: A Blockchain Framework for Patient-Centered Health Records and Exchange (HealthChain): Evaluation and Proof-of-Concept Study
Source: J Med Internet Res. 2019 Aug 31;21(8):e13592. doi: 10.2196/13592 (PMC6743266; doi:10.2196/13592)
Supplement: Multimedia Appendix 3 [file jmir_v21i8e13592_app3.zip › ProxyReEncryption/javadoc/allclasses-frame.html]

All Classes


# All Classes

- AFGH
- AFGH.LENGTH
- Cipher
- Cipher.LEVEL
- Cipher.PAIRING\_TYPE
- Constants
- Keys
- Message
- ProxyReEncryption
- ProxyReEncryption.ENCRYPTION\_MODE
- ProxyReEncryption.MESSAGE\_TYPE
- ProxyReEncryption.REENCRYPT
- ProxyReEncryption.SCALE
- ProxyReEncryption.STORAGE
- ReKeys
- TypeAPairingIO
