# Supplementary material for: A Blockchain Framework for Patient-Centered Health Records and Exchange (HealthChain): Evaluation and Proof-of-Concept Study
Source: J Med Internet Res. 2019 Aug 31;21(8):e13592. doi: 10.2196/13592 (PMC6743266; doi:10.2196/13592)
Supplement: Multimedia Appendix 3 [file jmir_v21i8e13592_app3.zip › ProxyReEncryption/javadoc/constant-values.html]

Constant Field Values


JavaScript is disabled on your browser.


Skip navigation links


- Package
- Class
- Use
- Tree
- Deprecated
- Index
- Help

- Prev
- Next

- Frames
- No Frames

- All Classes

# Constant Field Values

## Contents

- edu.ecu.\*

## edu.ecu.\*

- edu.ecu.hsim.ray.proxyreencryption.Constants

  | Modifier and Type | Constant Field | Value |
  | --- | --- | --- |
  |  |  |  |
  | --- | --- | --- |
  | `public static final java.lang.String` | `CIPHER_EXT` | `".cipher"` |
  | `public static final java.lang.String` | `CURVE_PARAMS_EXT` | `".params"` |
  | `public static final java.lang.String` | `DOI` | `"IN CONGRESS, JULY 4, 1776. \nTHE UNANIMOUS \nDECLARATION \nOF THE \nTHIRTEEN UNITED STATES OF AMERICA.\nWHEN, in the Course of human Events, it becomes necessary for one People to dissolve the Political Bands which have connected them with another, and to assume, among the Powers of the Earth, the separate and equal Station to which the Laws of Nature and of Nature\'s GOD entitle them, a decent Respect to the Opinions of Mankind requires that they should declare the Causes which impel them to the Separation.\nWe hold these Truths to be self-evident, that all Men are created equal, that they are endowed, by their CREATOR, with certain unalienable Rights, that among these are Life, Liberty, and the Pursuit of Happiness.--That to secure these Rights, Governments are instituted among Men, deriving their just Powers from the Consent of the Governed, that whenever any Form of Government becomes destructive of these Ends, it is the Right of the People to alter or to abolish it, and to institute new Government, laying its Foundation on such Principles, and organizing its Powers in such Form, as to them shall seem most likely to effect their Safety and Happiness. Prudence, indeed, will dictate, that Governments long established, should not be changed for light and transient Causes; and accordingly all Experience hath shewn, that Mankind are more disposed to suffer, while Evils are sufferable, than to right themselves by abolishing the Forms to which they are accustomed. But when a long Train of Abuses and Usurpations, pursuing invariably the same Object, evinces a Design to reduce them under absolute Despotism, it is their Right, it is their Duty, to throw off such Government, and to provide new Guards for their future Security. Such has been the patient Sufferance of these Colonies; and such is now the Necessity which constrains them to alter their former Systems of Government. The History of the present King of Great-Britain is a History of repeated Injuries and Usurpations, all having in direct Object the Establishment of an absolute Tyranny over these States. To prove this, let Facts be submitted to a candid World.\nHE has refused his Assent to Laws, the most wholesome and necessary for the public Good.\nHE has forbidden his Governors to pass Laws of immediate and pressing Importance, unless suspended in their Operation till his Assent should be obtained; and when so suspended, he has utterly neglected to attend to them.\nHE has refused to pass other Laws for the Accommodation of large Districts of People, unless those People would relinquish the Right of Representation in the Legislature, a Right inestimable to them, and formidable to Tyranny only.\nHE has called together Legislative Bodies at Places unusual, uncomfortable, and distant from the Depository of their public Records, for the sole Purpose of fatiguing them into Compliance with his Measures.\nHE has dissolved Representative Houses repeatedly, for opposing with manly Firmness his Invasions on the Rights of the People.\nHE has refused for a long Time, after such Dissolutions, to cause others to be elected; whereby the Legislative Powers, incapable of Annihilation, have returned to the People at large for their exercise; the State remaining, in the mean Time, exposed to all the Dangers of Invasion from without, and Convulsions within.\nHE has endeavoured to prevent the Population of these States; for that Purpose obstructing the Laws for Naturalization of Foreigners; refusing to pass others to encourage their Migrations hither, and raising the Conditions of new Appropriations of Lands.\nHE has obstructed the Administration of Justice, by refusing his Assent to Laws for establishing Judiciary Powers.\nHE has made Judges dependent on his Will alone, for the Tenure of their Offices, and the Amount and Payment of their Salaries.\nHE has erected a Multitude of new Offices, and sent hither Swarms of Officers to harrass our People, and eat out their Substance.\nHE has kept among us, in Times of Peace, Standing Armies, without the Consent of our Legislatures.\nHE has affected to render the Military independent of and superior to the Civil Power.\nHE has combined with others to subject us to a Jurisdiction foreign to our Constitution, and unacknowledged by our Laws; giving his Assent to their Acts of pretended Legislation:\nFOR quartering large Bodies of Armed Troops among us:\nFOR protecting them, by a mock Trial, from Punishment for any Murders which they should commit on the Inhabitants of these States:\nFOR cutting off our Trade with all Parts of the World:\nFOR imposing Taxes on us without our Consent:\nFOR depriving us, in many Cases, of the Benefits of Trial by Jury:\nFOR transporting us beyond Seas to be tried for pretended Offences:\nFOR abolishing the free System of English Laws in a neighbouring Province, establishing therein an arbitrary Government, and enlarging its Boundaries, so as to render it at once an Example and fit Instrument for introducing the same absolute Rule into these Colonies:\nFOR taking away our Charters, abolishing our most valuable Laws, and altering fundamentally the Forms of our Governments:\nFOR suspending our own Legislatures, and declaring themselves invested with Power to legislate for us in all Cases whatsoever.\nHE has abdicated Government here, by declaring us out of his Protection, and waging War against us.\nHE has plundered our Seas, ravaged our Coasts, burnt our Towns, and destroyed the Lives of our People.\nHE is, at this Time, transporting large Armies of foreign Mercenaries to complete the Works of Death, Desolation, and Tyranny, already begun with Circumstances of Cruelty and Perfidy, scarcely paralleled in the most barbarous Ages, and totally unworthy the Head of a civilized Nation.\nHE has constrained our Fellow-Citizens, taken Captive on the high Seas, to bear Arms against their Country, to become the Executioners of their Friends and Brethren, or to fall themselves by their Hands.\nHE has excited domestic Insurrections amongst us, and has endeavoured to bring on the Inhabitants of our Frontiers, the merciless Indian Savages, whose known Rule of Warfare, is an undistinguished Destruction, of all Ages, Sexes, and Conditions.\nIN every Stage of these Oppressions we have Petitioned for Redress in the most humble Terms: Our repeated Petitions have been answered only by repeated Injury. A Prince, whose Character is thus marked by every Act which may define a Tyrant, is unfit to be the Ruler of a free People.\nNOR have we been wanting in Attentions to our British Brethren. We have warned them, from Time to Time, of Attempts by their Legislature to extend an unwarrantable Jurisdiction over us. We have reminded them of the Circumstances of our Emigration and Settlement here. We have appealed to their native Justice and Magnanimity, and we have conjured them by the Ties of our common Kindred to disavow these Usurpations, which would inevitably interrupt our Connexions and Correspondence. They too have been deaf to the Voice of Justice and of Consanguinity. We must, therefore, acquiesce in the Necessity, which denounces our Separation, and hold them, as we hold the Rest of Mankind, Enemies in War, in Peace Friends.\nWE, therefore, the Representatives of the UNITED STATES OF AMERICA, in GENERAL CONGRESS Assembled, appealing to the Supreme Judge of the World for the Rectitude of our Intentions, do, in the Name, and by Authority of the good People of these Colonies, solemnly Publish and Declare, That these United Colonies are, and of Right ought to be, FREE AND INDEPENDENT STATES; that they are absolved from all Allegiance to the British Crown, and that all political Connexion between them and the State of Great-Britain, is, and ought to be, totally dissolved; and that as FREE AND INDEPENDENT STATES, they have full Power to levy War, conclude Peace, contract Alliances, establish Commerce, and to do all other Acts and Things which INDEPENDENT STATES may of Right do. And for the Support of this Declaration, with a firm Reliance on the Protection of DIVINE PROVIDENCE, we mutually pledge to each other our Lives, our Fortunes, and our sacred Honour."` |
  | `public static final java.lang.String` | `ENC_MODE_PREFIX` | `"em"` |
  | `public static final java.lang.String` | `EQ_PREFIX_I` | `"sp"` |
  | `public static final java.lang.String` | `EQ_PREFIX_N` | `"b64size"` |
  | `public static final java.lang.String` | `G1P_PREFIX` | `"g1p"` |
  | `public static final java.lang.String` | `G1X_PREFIX` | `"g1x"` |
  | `public static final java.lang.String` | `G1Y_PREFIX` | `"g1y"` |
  | `public static final java.lang.String` | `G2P_PREFIX` | `"g2p"` |
  | `public static final java.lang.String` | `G2X_PREFIX` | `"g2x"` |
  | `public static final java.lang.String` | `G2Y_PREFIX` | `"g2y"` |
  | `public static final java.lang.String` | `GEN_PREFIX_I` | `"gn"` |
  | `public static final java.lang.String` | `GEN_PREFIX_N` | `"gb64size"` |
  | `public static final java.lang.String` | `KEY_K` | `"k"` |
  | `public static final java.lang.String` | `KEY_L` | `"l"` |
  | `public static final java.lang.String` | `KEY_V` | `"v"` |
  | `public static final java.lang.String` | `KEY_Z` | `"z"` |
  | `public static final java.lang.String` | `MESSAGE_EXT` | `".message"` |
  | `public static final java.lang.String` | `PK_EXT` | `".pub"` |
  | `public static final java.lang.String` | `PK_PREFIX` | `"pk"` |
  | `public static final java.lang.String` | `RK_ENCP` | `"en"` |
  | `public static final java.lang.String` | `RK_EXT` | `".rek"` |
  | `public static final java.lang.String` | `RK_RKEY` | `"rk"` |
  | `public static final java.lang.String` | `RK_TPS` | `"tps"` |
  | `public static final java.lang.String` | `SK_EXT` | `".sec"` |
  | `public static final java.lang.String` | `ZP_PREFIX` | `"zp"` |
  | `public static final java.lang.String` | `ZR_FQ_PREFIX` | `"v"` |

Skip navigation links


- Package
- Class
- Use
- Tree
- Deprecated
- Index
- Help

- Prev
- Next

- Frames
- No Frames

- All Classes
