# Supplementary material for: A Blockchain Framework for Patient-Centered Health Records and Exchange (HealthChain): Evaluation and Proof-of-Concept Study
Source: J Med Internet Res. 2019 Aug 31;21(8):e13592. doi: 10.2196/13592 (PMC6743266; doi:10.2196/13592)
Supplement: Multimedia Appendix 3 [file jmir_v21i8e13592_app3.zip › ProxyReEncryption/javadoc/edu/ecu/hsim/ray/proxyreencryption/AFGH.html]

AFGH


JavaScript is disabled on your browser.


Skip navigation links


- Package
- Class
- Use
- Tree
- Deprecated
- Index
- Help

- Prev Class
- Next Class

- Frames
- No Frames

- All Classes

- Summary:
- Nested |
- Field |
- Constr |
- Method

- Detail:
- Field |
- Constr |
- Method


edu.ecu.hsim.ray.proxyreencryption

## Class AFGH

- java.lang.Object
- - edu.ecu.hsim.ray.proxyreencryption.ProxyReEncryption
  - - edu.ecu.hsim.ray.proxyreencryption.AFGH

- ---

    

  ```
  public class AFGH
  extends ProxyReEncryption
  ```

  ### Background

  Basic AFGH proxy re-encryption implementation (followed "A Second Attempt" on
  page 9) [1] using jPBC [2]. Uses elliptic curves with built-in support for
  160, 224, 256, 384, and 521-bit elliptic curve keys (see `AFGH.LENGTH` for
  more details).

  AFGH allows for two forms of encryption/decryption and proxy re-encryption.
  The first type of encryption/decryption is *first-level*. First-level
  does NOT allow for re-encryption. That is, a message encrypted using
  first-level can only be decrypted by the one who performed the encryption.
  Second-level allows for the previous *and* re-encryption. Using only the
  originator's (\(A\)) secret key (\(sk\_{A}\)) and recipient's (\(B\)) public
  key (\(pk\_{B}\)), a re-encryption key (\(rk\_{A\rightarrow{B}}\)) can be
  faceted. Any data encrypted by \(A\) can be decrypted by \(B\). However, this
  process is NOT invertible. That is, \(B\) cannot use \(rk\_{A\rightarrow{B}}\)
  to encrypt a message that \(A\) can decrypt. Similarly,
  \(rk\_{A\rightarrow{B}}\) is a first-level entity. Thus, \(B\) cannot
  re-encrypt the data from \(A\) and provide it to a third-party (e.g., \(C\))
  - a security feature. In essence, \(B\) is granted decrypt-only access by
  \(A\).

  #### Basic definitions

  Encryption: \(m\_{A}Z^{k\_{A}}=c\_{A}\) where \(m\_{A}\) is \(A\)'s message,
  \(Z=e(g,g)\), and \(k\_{A}\) is a random number in \(\mathbb{Z}\_{q}\). This
  represents the `ProxyReEncryption.ENCRYPTION_MODE.STATIC` case. For
  `ProxyReEncryption.ENCRYPTION_MODE.DYNAMIC`, \(k\_{A}\) is redefined as \(k\_{A}^{i}\). The
  encryption process is thus augmented to
  \(m\_{A}^{i}Z^{k\_{A}^{i}}=c\_{A}^{i}\).

  First-level decryption:
  \(\frac{m\_{A}Z^{k\_{A}}}{e\left(g^{a},g^{k\_{A}}\right)^{-a}}=m\_{A}\) where
  \(a\) is \(A\)'s secret key (\(sk\_{A}\)), \(g^{a}\) is \(A\)'s public key,
  (\(pk\_{A}\)) and \(g^{k\_{A}}\) is a secret parameter only \(A\) knows.
  Trivially adjusted to support a dynamic \(k\_{A}^{i}\).

  Second-level decryption:
  \(\frac{m\_{A}Z^{k\_{A}}}{e\left(g^{ak\_{A}},g\right)^{-a}}=m\_{A}\) where
  \(g^{ak\_{A}}\) is the public decryption parameter and accompanies the cipher
  when distributed. Trivially adjusted to support a dynamic \(k\_{A}^{i}\).

  Re-encryption:
  \(\frac{m\_{A}Z^{k\_{A}}}{e\left(g^{ak\_{A}},g^{\frac{b}{a}}\right)^{-b}}=m\_{A}\)
  where \(g^{b}\) is \(B\)'s public key (\(pk\_{B}\)), \(b\) is \(B\)'s secret
  key (\(sk\_{B}\)), and \(\left(pk\_{B}\right)^{-sk\_{A}}=\left(g^{b}\right)^{-a}
  = g^{\frac{b}{a}}\) is the re-encryption key \(rk\_{A\rightarrow B}\) only
  computable by \(A\). Trivially adjusted to support a dynamic
  \(k\_{A}^{i}\).

  Two-party re-encryption:

  - Encryption: \(m\_{A}Z^{k\_{A}}\). Trivially adjusted to support a dynamic
    \(k\_{A}^{i}\).
  - Intermediate (\(R\)) re-encryption:
    \(\left(m\_{A}Z^{k\_{A}}\right)\left(\frac{Z^{rk\_{A}}}{Z^{k\_{A}}}\right)=
    m\_{A}Z^{rk\_{A}}\) where \(Z^{rk\_{A}}=e\left(pk\_{R},g^{k\_{A}}\right)=
    e\left(g^{r},g^{k\_{A}}\right)\), which can only be computed by \(A\).
    Trivially adjusted to support a dynamic \(k\_{A}^{i}\).
  - Delegatee decryption:
    \(\frac{m\_{A}Z^{rk\_{A}}}{e\left(g^{ral\_{A}},g^{\frac{bk\_{A}}{al\_{A}}}\right)^{-b}}
    =m\_{A}\) where \(l\_{A}\) is a random number in \(\mathbb{Z}\_{q}\),
    \(\left(pk\_{R}\right)^{sk\_{A}l\_{A}}=\left(g^{r}\right)^{al\_{A}}=g^{ral\_{A}}\)
    can only be computed by \(A\), and \(\left(pk\_{B}\right)^{\frac{k\_{A}}{sk\_{A}l\_{A}}}
    =\left(g^{b}\right)^{\frac{k\_{A}}{al\_{A}}}=g^{\frac{bk\_{A}}{al\_{A}}}\) can
    also only be computed by \(A\). Trivially adjusted to support a dynamic
    \(k\_{A}^{i}\).

  Sign/verify:

  - Sign: signed message \(m\_{A}Z^{x\_{A}}\) (where \(x\_{A}\) is an ephemeral,
    random number in \(\mathbb{Z}\_{q}\) such that \(x\_{A}\neq a,k\_{A,}l\_{A}\)),
    hash of \(m\_{A}\) (\(m\_{A}^{\tau}=H\left(m\_{A}\right)\), where
    \(H\left(\cdot\right)\) is a one-way cryptographic hashing function), and
    public verification parameter \(g^{\frac{x\_{A}}{a+m\_{A}^{\tau}}}\). Trivially
    adjusted to support a dynamic \(x\_{A}^{i}\).
  - Verify: if \(H\left(\frac{m\_{A}Z^{x\_{A}}}{e\left(g^{\frac{x\_{A}}
    {a+m\_{A}^{\tau}}},pk\_{A}g^{m\_{A}^{\tau}}\right)}\right)=m\_{A}^{\tau}\), then
    verified. Trivially adjusted to support a dynamic \(x\_{A}^{i}\).

  Encrypt-sign/decrypt-verify:

  - Encrypt-sign: signed message \(m\_{A}Z^{x\_{A}}\) (where \(x\_{A}\) is an
    ephemeral, random number in \(\mathbb{Z}\_{q}\) such that \(x\_{A}\neq
    a,k\_{A,}l\_{A}\)), hash of \(m\_{A}\) (\(m\_{A}^{\tau}=H\left(m\_{A}\right)\),
    where \(H\left(\cdot\right)\) is a one-way cryptographic hashing function),
    and verification parameter \(pk\_{B}^{\frac{x\_{A}}{sk\_{A}+m\_{A}^{\tau}}}
    =g^{\frac{bx\_{A}}{a+m\_{A}^{\tau}}}\) encrypted for \(B\). Trivially adjusted
    to support a dynamic \(x\_{A}^{i}\).
  - Decrypt-verify: if \(H\left(\frac{m\_{A}Z^{x\_{A}}}{e\left(g^{\frac{bx\_{A}}
    {a+m\_{A}^{\tau}}},pk\_{A}g^{m\_{A}^{\tau}}\right)^{-b}}\right)=m\_{A}^{\tau}\),
    then decrypt-verified. Trivially adjusted to support a dynamic \(x\_{A}^{i}\).

  ### References

  [1] Ateniese, Giuseppe and Fu, Kevin and Green, Matthew and Hohenberger,
  Susan (2006). Improved proxy re-encryption schemes with applications to
  secure distributed storage. *ACM Transactions on Information and Systems
  Security*. 9(1), (February 2006), pp. 1-30.
  Link.

  [2] De Caro, Angelo and Iovino, Vincenzo (2011). jPBC: Java pairing based
  cryptography. *Proceedings of the 16th IEEE Symposium on Computers and
  Communications, ISCC 2011*. pp. 850-855.
  Link.

- - ### Nested Class Summary

    Nested Classes

    | Modifier and Type | Class and Description |
    | --- | --- |
    | `static class` | `AFGH.LENGTH` The employed curve is a Type A pairing Montgomery curve with embedding degree of \(k=2\). |

    - ### Nested classes/interfaces inherited from class edu.ecu.hsim.ray.proxyreencryption.ProxyReEncryption

      `ProxyReEncryption.ENCRYPTION_MODE, ProxyReEncryption.MESSAGE_TYPE, ProxyReEncryption.REENCRYPT, ProxyReEncryption.SCALE, ProxyReEncryption.STORAGE`
  - ### Field Summary

    - ### Fields inherited from class edu.ecu.hsim.ray.proxyreencryption.ProxyReEncryption

      `curveFile, curveParams, curveProperties, DEFAULT_ENCRYPTION_MODE, DEFAULT_REENCRYPT, e, encMode, encModeHM, g1, G1, g1p, g2, G2, g2p, GT, log, order, storage, Z, Zp, Zr`
  - ### Constructor Summary

    Constructors

    Constructor and Description

    `AFGH()`

    Constructs a new `STORAGE#VOLATILE` `AFGH` with elliptic
    curve key length of size `DEFAULT_LENGTH` and encryption mode of
    `ProxyReEncryption.DEFAULT_ENCRYPTION_MODE`.

    `AFGH(AFGH.LENGTH length)`

    Constructs a new `STORAGE#VOLATILE` `AFGH` with elliptic
    curve key length of size `length` and encryption mode of
    `ProxyReEncryption.DEFAULT_ENCRYPTION_MODE`.

    `AFGH(AFGH.LENGTH length,
    byte[] curveParams)`

    Constructs a new `STORAGE#NONVOLATILE` `AFGH`.

    `AFGH(AFGH.LENGTH length,
    byte[] curveParams,
    ProxyReEncryption.ENCRYPTION_MODE encMode)`

    Constructs a new `STORAGE#NONVOLATILE` `AFGH`.

    `AFGH(AFGH.LENGTH length,
    ProxyReEncryption.ENCRYPTION_MODE encMode)`

    Constructs a new `STORAGE#VOLATILE` `AFGH` with elliptic
    curve key length of size `length` and encryption mode of
    `encMode`.

    `AFGH(AFGH.LENGTH length,
    java.lang.String curveParams)`

    Constructs a new `STORAGE#NONVOLATILE` `AFGH`.

    `AFGH(AFGH.LENGTH length,
    java.lang.String curveParams,
    ProxyReEncryption.ENCRYPTION_MODE encMode)`

    Constructs a new `STORAGE#NONVOLATILE` `AFGH`.

    `AFGH(byte[] curveParams,
    ProxyReEncryption.STORAGE storage)`

    Constructs a new `storage` `AFGH`.

    `AFGH(byte[] curveParams,
    ProxyReEncryption.STORAGE storage,
    ProxyReEncryption.ENCRYPTION_MODE encMode)`

    Constructs a new `storage` `AFGH`.

    `AFGH(ProxyReEncryption.ENCRYPTION_MODE encMode)`

    Constructs a new `STORAGE#VOLATILE` `AFGH` with elliptic
    curve key length of size `DEFAULT_LENGTH` and k mode of
    `encMode`.

    `AFGH(java.lang.String curveParams,
    ProxyReEncryption.STORAGE storage)`

    Constructs a new `storage` `AFGH`.

    `AFGH(java.lang.String curveParams,
    ProxyReEncryption.STORAGE storage,
    ProxyReEncryption.ENCRYPTION_MODE encMode)`

    Constructs a new `storage` `AFGH`.
  - ### Method Summary

    All Methods Instance Methods Concrete Methods

    | Modifier and Type | Method and Description |
    | --- | --- |
    | `Message` | `decrypt(it.unisa.dia.gas.jpbc.Element x, Cipher cipher)` Decrypts the `Cipher` using the provided ephemeral key `x`. |
    | `Message` | `decrypt(it.unisa.dia.gas.jpbc.Element x, Message message)` Decrypts the `Message` using the provided ephemeral key `x`. |
    | `Message` | `decrypt(Keys key, Cipher cipher)` Decrypts the `Cipher` using the provided private key. |
    | `Message` | `decrypt(Keys key, Message message)` Decrypts the `Message` using the provided private key. |
    | `Message` | `decryptDelegatee(Keys key, Cipher cipher)` Decrypts the delegatee layer of a two-party re-encryption `Cipher` using the provided private key. |
    | `Cipher` | `decryptIntermediate(Keys key, Cipher cipher)` Decrypts the intermediate layer of a two-party re-encryption `Cipher` using the provided private key. |
    | `Cipher` | `decryptToCipher(it.unisa.dia.gas.jpbc.Element x, Cipher cipher)` Decrypts the `Cipher` to a `Cipher` using the provided ephemeral key `x`. |
    | `Cipher` | `decryptToCipher(Keys key, Cipher cipher)` Decrypts the `Cipher` to a `Cipher` using the provided private key. |
    | `Message` | `decryptVerifyImpl(Keys verify, Keys decrypt, Message cipher)` Decrypt-verifies the `cipher` using the private key of the recipient (`decrypt`) and public key of the signer (`verify`), returning the decrypt-verified cipher as a `Message`. |
    | `Cipher` | `encrypt(it.unisa.dia.gas.jpbc.Element x, byte[] message, ProxyReEncryption.MESSAGE_TYPE type)` Encrypts the `message` using the ephemeral key `x`. |
    | `Cipher` | `encrypt(it.unisa.dia.gas.jpbc.Element x, Cipher cipher)` Encrypts the `Cipher` using the ephemeral key `x`. |
    | `Cipher` | `encrypt(it.unisa.dia.gas.jpbc.Element x, Message message)` Encrypts the `Message` using the ephemeral key `x`. |
    | `Cipher` | `encrypt(it.unisa.dia.gas.jpbc.Element x, java.lang.String message)` Encrypts the `message` using the ephemeral key `x`. |
    | `Cipher` | `encrypt(Keys keys, byte[] message, ProxyReEncryption.MESSAGE_TYPE type)` Encrypts the `message` using the public key. |
    | `Cipher` | `encrypt(Keys keys, byte[] message, ProxyReEncryption.MESSAGE_TYPE type, ProxyReEncryption.REENCRYPT reencrypt)` Encrypts the `message` using the public key and `reencrypt` parameter. |
    | `Cipher` | `encrypt(Keys keys, Cipher cipher)` Encrypts the `Cipher` using the public key. |
    | `Cipher` | `encrypt(Keys keys, Cipher cipher, ProxyReEncryption.REENCRYPT reencrypt)` Encrypts the `Cipher` using the public key and `reencrypt` parameter. |
    | `Cipher` | `encrypt(Keys keys, Message message)` Encrypts the `Message` using the public key. |
    | `Cipher` | `encrypt(Keys keys, Message message, ProxyReEncryption.REENCRYPT reencrypt)` Encrypts the `Message` using the public key and `reencrypt` parameter. |
    | `Cipher` | `encrypt(Keys keys, java.lang.String message)` Encrypts the `message` using the public key. |
    | `Cipher` | `encrypt(Keys keys, java.lang.String message, ProxyReEncryption.REENCRYPT reencrypt)` Encrypts the `message` using the public key and `reencrypt` parameter. |
    | `Cipher` | `reencrypt(Keys rekey, Cipher cipher)` Re-encrypts a `Cipher` using the re-encryption `Keys`. |
    | `Cipher` | `reencrypt(Keys key, Keys rekey, Cipher cipher)` Re-encrypts a two-party `Cipher` using the re-encryption `Keys`. |
    | `void` | `scale(Cipher cipher, it.unisa.dia.gas.jpbc.Element from, it.unisa.dia.gas.jpbc.Element to, ProxyReEncryption.SCALE scaleType)` Transforms the encryption key of the `Cipher` from `from` to `to` by way of simple scaling. |
    | `void` | `scale(Cipher cipher, it.unisa.dia.gas.jpbc.Element scalar, ProxyReEncryption.SCALE scaleType)` Transforms the encryption key of the `Cipher` from `from` to `to` by way of simple scaling. |
    | `void` | `scale(Message message, it.unisa.dia.gas.jpbc.Element from, it.unisa.dia.gas.jpbc.Element to, ProxyReEncryption.SCALE scaleType)` Transforms the encryption key of the `Message` from `from` to `to` by way of simple scaling. |
    | `void` | `scale(Message message, it.unisa.dia.gas.jpbc.Element scalar, ProxyReEncryption.SCALE scaleType)` Transforms the encryption key of the `Message` from `from` to `to` by way of simple scaling. |
    | `Cipher` | `sign(Keys keys, byte[] message, ProxyReEncryption.MESSAGE_TYPE type)` Signs the `message` using an ephemeral key `x`, returning a `Cipher` that is verified by its hash and a private key-based decryption parameter (all contained within the `Cipher`). |
    | `Cipher` | `sign(Keys keys, Cipher cipher)` Signs the `Cipher` using an ephemeral key `x`, returning a `Cipher` that is verified by its hash and a private key-based decryption parameter (all contained within the `Cipher`). |
    | `Cipher` | `sign(Keys sign, Keys encrypt, byte[] message, ProxyReEncryption.MESSAGE_TYPE type)` Encrypt-signs the `message` using an ephemeral key `x`, returning a `Cipher` that is decrypt-verified by its hash and a decryption parameter constructed using the signer's private key and recipients public key (all contained within the `Cipher`). |
    | `Cipher` | `sign(Keys sign, Keys encrypt, Cipher cipher)` Encrypt-signs the `Cipher` using an ephemeral key `x`, returning a `Cipher` that is decrypt-verified by its hash and a decryption parameter constructed using the signer's private key and recipients public key (all contained within the `Cipher`). |
    | `Cipher` | `sign(Keys sign, Keys encrypt, Message message)` Encrypt-signs the `Message` using an ephemeral key `x`, returning a `Cipher` that is decrypt-verified by its hash and a decryption parameter constructed using the signer's private key and recipients public key (all contained within the `Cipher`). |
    | `Cipher` | `sign(Keys sign, Keys encrypt, java.lang.String message)` Encrypt-signs the `message` using an ephemeral key `x`, returning a `Cipher` that is decrypt-verified by its hash and a decryption parameter constructed using the signer's private key and recipients public key (all contained within the `Cipher`). |
    | `Cipher` | `sign(Keys keys, Message message)` Signs the `Message` using an ephemeral key `x`, returning a `Cipher` that is verified by its hash and a private key-based decryption parameter (all contained within the `Cipher`). |
    | `Cipher` | `sign(Keys keys, java.lang.String message)` Signs the `message` using an ephemeral key `x`, returning a `Cipher` that is verified by its hash and a private key-based decryption parameter (all contained within the `Cipher`). |
    | `Cipher` | `toCipher(byte[] bytes)` Converts a `byte[]` to a `Cipher`. |
    | `Message` | `toMessage(byte[] message, int bytePadding)` Converts a `byte[]` to a `Message` with padding. |
    | `Message` | `toMessage(byte[] message, ProxyReEncryption.MESSAGE_TYPE type)` Converts a `byte[]` to a `Message`. |
    | `Message` | `toMessage(java.lang.String message)` Converts a `String` to a `Message`. |
    | `boolean` | `verify(Keys keys, Cipher cipher)` Verifies a signed `Cipher` using the signer's private key, a message hash, and a decryption parameter generated using the signer's private key and the message hash. |
    | `boolean` | `verify(Keys keys, Cipher cipher, byte[] expected)` Verifies a signed `Cipher` using the signer's private key, a message hash, and a decryption parameter generated using the signer's private key and the message hash. |
    | `boolean` | `verify(Keys keys, Cipher cipher, Message expected)` Verifies a signed `Cipher` using the signer's private key, a message hash, and a decryption parameter generated using the signer's private key and the message hash. |
    | `boolean` | `verify(Keys keys, Cipher cipher, java.lang.String expected)` Verifies a signed `Cipher` using the signer's private key, a message hash, and a decryption parameter generated using the signer's private key and the message hash. |
    | `boolean` | `verify(Keys verify, Keys decrypt, Cipher cipher)` Decrypt-verifies a signed `Cipher` using the signer's private key, a message hash, and a decryption parameter generated using the signer's private key and the message hash. |
    | `boolean` | `verify(Keys verify, Keys decrypt, Cipher cipher, byte[] expected)` Decrypt-verifies a signed `Cipher` using the signer's private key, a message hash, and a decryption parameter generated using the signer's private key and the message hash. |
    | `boolean` | `verify(Keys verify, Keys decrypt, Cipher cipher, Message expected)` Decrypt-verifies a signed `Cipher` using the signer's private key, a message hash, and a decryption parameter generated using the signer's private key and the message hash. |
    | `boolean` | `verify(Keys verify, Keys decrypt, Cipher cipher, java.lang.String expected)` Decrypt-verifies a signed `Cipher` using the signer's private key, a message hash, and a decryption parameter generated using the signer's private key and the message hash. |
    | `boolean` | `verify(Keys verify, Keys decrypt, Message message)` Decrypt-verifies a signed `Message` using the signer's private key, a message hash, and a decryption parameter generated using the signer's private key and the message hash. |
    | `boolean` | `verify(Keys verify, Keys decrypt, Message message, byte[] expected)` Decrypt-verifies a signed `Message` using the signer's private key, a message hash, and a decryption parameter generated using the signer's private key and the message hash. |
    | `boolean` | `verify(Keys verify, Keys decrypt, Message message, Message expected)` Decrypt-verifies a signed `Message` using the signer's private key, a message hash, and a decryption parameter generated using the signer's private key and the message hash. |
    | `boolean` | `verify(Keys verify, Keys decrypt, Message message, java.lang.String expected)` Decrypt-verifies a signed `Message` using the signer's private key, a message hash, and a decryption parameter generated using the signer's private key and the message hash. |
    | `boolean` | `verify(Keys keys, Message message)` Verifies a signed `Message` using the signer's private key, a message hash, and a decryption parameter generated using the signer's private key and the message hash. |
    | `boolean` | `verify(Keys keys, Message message, byte[] expected)` Verifies a signed `Message` using the signer's private key, a message hash, and a decryption parameter generated using the signer's private key and the message hash. |
    | `boolean` | `verify(Keys keys, Message message, Message expected)` Verifies a signed `Message` using the signer's private key, a message hash, and a decryption parameter generated using the signer's private key and the message hash. |
    | `boolean` | `verify(Keys keys, Message message, java.lang.String expected)` Verifies a signed `Message` using the signer's private key, a message hash, and a decryption parameter generated using the signer's private key and the message hash. |
    | `Message` | `verifyAndGet(Keys keys, Cipher cipher)` Verifies a signed `Cipher` using the signer's private key, a message hash, and a decryption parameter generated using the signer's private key and the message hash. |
    | `Message` | `verifyAndGet(Keys verify, Keys decrypt, Cipher cipher)` Decrypt-verifies a signed `Cipher` using the signer's private key, a message hash, and a decryption parameter generated using the signer's private key and the message hash. |
    | `Message` | `verifyAndGet(Keys verify, Keys decrypt, Message message)` Decrypt-verifies a signed `Message` using the signer's private key, a message hash, and a decryption parameter generated using the signer's private key and the message hash. |
    | `Message` | `verifyAndGet(Keys keys, Message message)` Verifies a signed `Message` using the signer's private key, a message hash, and a decryption parameter generated using the signer's private key and the message hash. |

    - ### Methods inherited from class edu.ecu.hsim.ray.proxyreencryption.ProxyReEncryption

      `getCurveFile, getCurveParams, getCurveProperties, getDefaultReEncrypt, getE, getEncryptionMode, getG1Gen, getG1GenP, getG2Gen, getG2GenP, getGT, getInfFlag, getLogLevel, getOrder, getZP, getZr, newEphemeralKeyX, newEphemeralKeyX, newG1CurveElement, newG1CurveElement, newG1CurveElement, newG2CurveElement, newG2CurveElement, newG2CurveElement, newGTElement, newGTElement, newGTElement, newKeys, newKeys, newKeys, newKeys, newReKeys, newReKeys, newReKeys, newReKeys, newReKeys, newZrElement, newZrElement, newZrElement, readCipher, readMessage, setDefaultReEncrypt, setEncryptionMode, setInfFlagToZero, setLogLevel`
    - ### Methods inherited from class java.lang.Object

      `clone, equals, finalize, getClass, hashCode, notify, notifyAll, toString, wait, wait, wait`

- - ### Constructor Detail


    - #### AFGH

      ```
      public AFGH()
      ```

      Constructs a new `STORAGE#VOLATILE` `AFGH` with elliptic
      curve key length of size `DEFAULT_LENGTH` and encryption mode of
      `ProxyReEncryption.DEFAULT_ENCRYPTION_MODE`.


    - #### AFGH

      ```
      public AFGH(ProxyReEncryption.ENCRYPTION_MODE encMode)
      ```

      Constructs a new `STORAGE#VOLATILE` `AFGH` with elliptic
      curve key length of size `DEFAULT_LENGTH` and k mode of
      `encMode`.

      Parameters:
      :   `encMode` - `ProxyReEncryption.ENCRYPTION_MODE`


    - #### AFGH

      ```
      public AFGH(AFGH.LENGTH length)
      ```

      Constructs a new `STORAGE#VOLATILE` `AFGH` with elliptic
      curve key length of size `length` and encryption mode of
      `ProxyReEncryption.DEFAULT_ENCRYPTION_MODE`.

      Parameters:
      :   `length` - elliptic curve key length


    - #### AFGH

      ```
      public AFGH(AFGH.LENGTH length,
                  ProxyReEncryption.ENCRYPTION_MODE encMode)
      ```

      Constructs a new `STORAGE#VOLATILE` `AFGH` with elliptic
      curve key length of size `length` and encryption mode of
      `encMode`.

      Parameters:
      :   `length` - elliptic curve key length
      :   `encMode` - `ProxyReEncryption.ENCRYPTION_MODE`


    - #### AFGH

      ```
      public AFGH(byte[] curveParams,
                  ProxyReEncryption.STORAGE storage)
      ```

      Constructs a new `storage` `AFGH`. Requires `storage`
      because the meaning of `curveParams` is ambiguous. That is,
      `curveParams` can represent a `STORAGE#NONVOLATILE` file for
      storage/retrieval or a `STORAGE#VOLATILE` properties
      `String`.

      If `storage` == `STORAGE#NONVOLATILE`, then it
      first tries to instantiate from an existing source - `curveParams`.
      If `curveParams` does not exist, it constructs a new
      `STORAGE#NONVOLATILE` AFGH with elliptic curve key length of size
      `DEFAULT_LENGTH`, encryption mode of
      `ProxyReEncryption.DEFAULT_ENCRYPTION_MODE`, and stores the resulting parameters in
      `curveParams`.

      If `storage` == `STORAGE#VOLATILE`, then it first
      tries to instantiate from an existing source - `curveParams`. If
      `curveParams` is `null`, it constructs a new
      `STORAGE#VOLATILE` AFGH with elliptic curve key length of size
      `DEFAULT_LENGTH` and encryption mode of
      `ProxyReEncryption.DEFAULT_ENCRYPTION_MODE`.

      Parameters:
      :   `curveParams` - curve parameter file to read from, if exists, or create
      :   `storage` - `STORAGE` option


    - #### AFGH

      ```
      public AFGH(java.lang.String curveParams,
                  ProxyReEncryption.STORAGE storage)
      ```

      Constructs a new `storage` `AFGH`. Requires `storage`
      because the meaning of `curveParams` is ambiguous. That is,
      `curveParams` can represent a `STORAGE#NONVOLATILE` file for
      storage/retrieval or a `STORAGE#VOLATILE` properties
      `String`.

      If `storage` == `STORAGE#NONVOLATILE`, then it
      first tries to instantiate from an existing source - `curveParams`.
      If `curveParams` does not exist, it constructs a new
      `STORAGE#NONVOLATILE` AFGH with elliptic curve key length of size
      `DEFAULT_LENGTH`, encryption mode of
      `ProxyReEncryption.DEFAULT_ENCRYPTION_MODE`, and stores the resulting parameters in
      `curveParams`.

      If `storage` == `STORAGE#VOLATILE`, then it first
      tries to instantiate from an existing source - `curveParams`. If
      `curveParams` is `null`, it constructs a new
      `STORAGE#VOLATILE` AFGH with elliptic curve key length of size
      `DEFAULT_LENGTH` and encryption mode of
      `ProxyReEncryption.DEFAULT_ENCRYPTION_MODE`.

      Parameters:
      :   `curveParams` - curve parameter file to read from, if exists, or create
      :   `storage` - `STORAGE` option


    - #### AFGH

      ```
      public AFGH(byte[] curveParams,
                  ProxyReEncryption.STORAGE storage,
                  ProxyReEncryption.ENCRYPTION_MODE encMode)
      ```

      Constructs a new `storage` `AFGH`. Requires `storage`
      because the meaning of `curveParams` is ambiguous. That is,
      `curveParams` can represent a `STORAGE#NONVOLATILE` file for
      storage/retrieval or a `STORAGE#VOLATILE` properties
      `String`.

      If `storage` == `STORAGE#NONVOLATILE`, then it
      first tries to instantiate from an existing source - `curveParams`.
      If `curveParams` does not exist, it constructs a new
      `STORAGE#NONVOLATILE` AFGH with elliptic curve key length of size
      `DEFAULT_LENGTH`, encryption mode of `encMode`, and stores the
      resulting parameters in `curveParams`.

      If `storage` == `STORAGE#VOLATILE`, then it first
      tries to instantiate from an existing source - `curveParams`. If
      `curveParams` is `null`, it constructs a new
      `STORAGE#VOLATILE` AFGH with elliptic curve key length of size
      `DEFAULT_LENGTH` and encryption mode of `encMode`.

      Parameters:
      :   `curveParams` - curve parameter file to read from, if exists, or create
      :   `storage` - `STORAGE` option
      :   `encMode` - `ProxyReEncryption.ENCRYPTION_MODE`


    - #### AFGH

      ```
      public AFGH(java.lang.String curveParams,
                  ProxyReEncryption.STORAGE storage,
                  ProxyReEncryption.ENCRYPTION_MODE encMode)
      ```

      Constructs a new `storage` `AFGH`. Requires `storage`
      because the meaning of `curveParams` is ambiguous. That is,
      `curveParams` can represent a `STORAGE#NONVOLATILE` file for
      storage/retrieval or a `STORAGE#VOLATILE` properties
      `String`.

      If `storage` == `STORAGE#NONVOLATILE`, then it
      first tries to instantiate from an existing source - `curveParams`.
      If `curveParams` does not exist, it constructs a new
      `STORAGE#NONVOLATILE` AFGH with elliptic curve key length of size
      `DEFAULT_LENGTH`, encryption mode of `encMode`, and stores the
      resulting parameters in `curveParams`.

      If `storage` == `STORAGE#VOLATILE`, then it first
      tries to instantiate from an existing source - `curveParams`. If
      `curveParams` is `null`, it constructs a new
      `STORAGE#VOLATILE` AFGH with elliptic curve key length of size
      `DEFAULT_LENGTH` and encryption mode of `encMode`.

      Parameters:
      :   `curveParams` - curve parameter file to read from, if exists, or create
      :   `storage` - `STORAGE` option
      :   `encMode` - `ProxyReEncryption.ENCRYPTION_MODE`


    - #### AFGH

      ```
      public AFGH(AFGH.LENGTH length,
                  byte[] curveParams)
      ```

      Constructs a new `STORAGE#NONVOLATILE` `AFGH`. It first tries
      to instantiate from an existing source - `curveParams`. If
      `curveParams` does not exist, it constructs a new
      `STORAGE#NONVOLATILE` AFGH with elliptic curve key length of size
      `length`, encryption mode of `ProxyReEncryption.DEFAULT_ENCRYPTION_MODE`, and
      stores the resulting parameters in `curveParams`.

      Parameters:
      :   `length` - elliptic curve key length
      :   `curveParams` - curve parameter file to read from, if exists, or create


    - #### AFGH

      ```
      public AFGH(AFGH.LENGTH length,
                  java.lang.String curveParams)
      ```

      Constructs a new `STORAGE#NONVOLATILE` `AFGH`. It first tries
      to instantiate from an existing source - `curveParams`. If
      `curveParams` does not exist, it constructs a new
      `STORAGE#NONVOLATILE` AFGH with elliptic curve key length of size
      `length`, encryption mode of `ProxyReEncryption.DEFAULT_ENCRYPTION_MODE`, and
      stores the resulting parameters in `curveParams`.

      Parameters:
      :   `length` - elliptic curve key length
      :   `curveParams` - curve parameter file to read from, if exists, or create


    - #### AFGH

      ```
      public AFGH(AFGH.LENGTH length,
                  byte[] curveParams,
                  ProxyReEncryption.ENCRYPTION_MODE encMode)
      ```

      Constructs a new `STORAGE#NONVOLATILE` `AFGH`. It first tries
      to instantiate from an existing source - `curveParams`. If
      `curveParams` does not exist, it constructs a new
      `STORAGE#NONVOLATILE` AFGH with elliptic curve key length of size
      `length`, encryption mode of `encMode`, and stores the
      resulting parameters in `curveParams`.

      Parameters:
      :   `length` - elliptic curve key length
      :   `curveParams` - curve parameter file to read from, if exists, or create
      :   `encMode` - `ProxyReEncryption.ENCRYPTION_MODE`


    - #### AFGH

      ```
      public AFGH(AFGH.LENGTH length,
                  java.lang.String curveParams,
                  ProxyReEncryption.ENCRYPTION_MODE encMode)
      ```

      Constructs a new `STORAGE#NONVOLATILE` `AFGH`. It first tries
      to instantiate from an existing source - `curveParams`. If
      `curveParams` does not exist, it constructs a new
      `STORAGE#NONVOLATILE` AFGH with elliptic curve key length of size
      `length`, encryption mode of `encMode`, and stores the
      resulting parameters in `curveParams`.

      Parameters:
      :   `length` - elliptic curve key length
      :   `curveParams` - curve parameter file to read from, if exists, or create
      :   `encMode` - `ProxyReEncryption.ENCRYPTION_MODE`
  - ### Method Detail


    - #### encrypt

      ```
      public Cipher encrypt(Keys keys,
                            java.lang.String message)
      ```

      Description copied from class: `ProxyReEncryption`

      Encrypts the `message` using the public key. Uses the
      `ProxyReEncryption.DEFAULT_REENCRYPT` parameter, which is initialized to
      `ProxyReEncryption.REENCRYPT.NO` and can be altered using
      `ProxyReEncryption.setDefaultReEncrypt(REENCRYPT)`. This issues new encryption
      parameters if `ProxyReEncryption.encMode` is `ProxyReEncryption.ENCRYPTION_MODE.DYNAMIC`.

      Specified by:
      :   `encrypt` in class `ProxyReEncryption`

      Parameters:
      :   `keys` - encryption `Keys`
      :   `message` - message to encrypt

      Returns:
      :   encrypted `Cipher`


    - #### encrypt

      ```
      public Cipher encrypt(Keys keys,
                            byte[] message,
                            ProxyReEncryption.MESSAGE_TYPE type)
      ```

      Description copied from class: `ProxyReEncryption`

      Encrypts the `message` using the public key. Uses the
      `ProxyReEncryption.DEFAULT_REENCRYPT` parameter, which is initialized to
      `ProxyReEncryption.REENCRYPT.NO` and can be altered using
      `ProxyReEncryption.setDefaultReEncrypt(REENCRYPT)`. This issues new encryption
      parameters if `ProxyReEncryption.encMode` is `ProxyReEncryption.ENCRYPTION_MODE.DYNAMIC`.

      Specified by:
      :   `encrypt` in class `ProxyReEncryption`

      Parameters:
      :   `keys` - encryption `Keys`
      :   `message` - message to encrypt
      :   `type` - `ProxyReEncryption.MESSAGE_TYPE.FORMATTED` if the passed bytes are
          the result of a `Message.toBytes()` call, as this
          result is formatted; otherwise, use
          `ProxyReEncryption.MESSAGE_TYPE.UNFORMATTED`

      Returns:
      :   encrypted `Cipher`


    - #### encrypt

      ```
      public Cipher encrypt(Keys keys,
                            Message message)
      ```

      Description copied from class: `ProxyReEncryption`

      Encrypts the `Message` using the public key. Uses the
      `ProxyReEncryption.DEFAULT_REENCRYPT` parameter, which is initialized to
      `ProxyReEncryption.REENCRYPT.NO` and can be altered using
      `ProxyReEncryption.setDefaultReEncrypt(REENCRYPT)`. This issues new encryption
      parameters if `ProxyReEncryption.encMode` is `ProxyReEncryption.ENCRYPTION_MODE.DYNAMIC`.

      Specified by:
      :   `encrypt` in class `ProxyReEncryption`

      Parameters:
      :   `keys` - encryption `Keys`
      :   `message` - `Message` to encrypt

      Returns:
      :   encrypted `Cipher`


    - #### encrypt

      ```
      public Cipher encrypt(Keys keys,
                            Cipher cipher)
      ```

      Description copied from class: `ProxyReEncryption`

      Encrypts the `Cipher` using the public key. Uses the
      `ProxyReEncryption.DEFAULT_REENCRYPT` parameter, which is initialized to
      `ProxyReEncryption.REENCRYPT.NO` and can be altered using
      `ProxyReEncryption.setDefaultReEncrypt(REENCRYPT)`. This issues new encryption
      parameters if `ProxyReEncryption.encMode` is `ProxyReEncryption.ENCRYPTION_MODE.DYNAMIC`.

      Specified by:
      :   `encrypt` in class `ProxyReEncryption`

      Parameters:
      :   `keys` - encryption `Keys`
      :   `cipher` - `Cipher` to encrypt

      Returns:
      :   encrypted `Cipher`


    - #### encrypt

      ```
      public Cipher encrypt(Keys keys,
                            java.lang.String message,
                            ProxyReEncryption.REENCRYPT reencrypt)
      ```

      Description copied from class: `ProxyReEncryption`

      Encrypts the `message` using the public key and `reencrypt`
      parameter. If `reencrypt` is `ProxyReEncryption.REENCRYPT.NO`, then first-level
      encryption is used (i.e., encryption that CANNOT be re-encrypted); else
      second-level (i.e., encryption that CAN be re-encrypted). This issues new
      encryption parameters if `ProxyReEncryption.encMode` is
      `ProxyReEncryption.ENCRYPTION_MODE.DYNAMIC`.

      Specified by:
      :   `encrypt` in class `ProxyReEncryption`

      Parameters:
      :   `keys` - encryption `Keys`
      :   `message` - message to encrypt
      :   `reencrypt` - `ProxyReEncryption.REENCRYPT` parameter

      Returns:
      :   encrypted `Cipher`


    - #### encrypt

      ```
      public Cipher encrypt(Keys keys,
                            byte[] message,
                            ProxyReEncryption.MESSAGE_TYPE type,
                            ProxyReEncryption.REENCRYPT reencrypt)
      ```

      Description copied from class: `ProxyReEncryption`

      Encrypts the `message` using the public key and `reencrypt`
      parameter. If `reencrypt` is `ProxyReEncryption.REENCRYPT.NO`, then first-level
      encryption is used (i.e., encryption that CANNOT be re-encrypted); else
      second-level (i.e., encryption that CAN be re-encrypted). This issues new
      encryption parameters if `ProxyReEncryption.encMode` is
      `ProxyReEncryption.ENCRYPTION_MODE.DYNAMIC`.

      Specified by:
      :   `encrypt` in class `ProxyReEncryption`

      Parameters:
      :   `keys` - encryption `Keys`
      :   `message` - message to encrypt
      :   `type` - `ProxyReEncryption.MESSAGE_TYPE.FORMATTED` if the passed bytes are
          the result of a `Message.toBytes()` call, as this
          result is formatted; otherwise, use
          `ProxyReEncryption.MESSAGE_TYPE.UNFORMATTED`
      :   `reencrypt` - `ProxyReEncryption.REENCRYPT` parameter

      Returns:
      :   encrypted `Cipher`


    - #### encrypt

      ```
      public Cipher encrypt(Keys keys,
                            Message message,
                            ProxyReEncryption.REENCRYPT reencrypt)
      ```

      Description copied from class: `ProxyReEncryption`

      Encrypts the `Message` using the public key and `reencrypt`
      parameter. If `reencrypt` is `ProxyReEncryption.REENCRYPT.NO`, then first-level
      encryption is used (i.e., encryption that CANNOT be re-encrypted); else
      second-level (i.e., encryption that CAN be re-encrypted). This issues new
      encryption parameters if `ProxyReEncryption.encMode` is
      `ProxyReEncryption.ENCRYPTION_MODE.DYNAMIC`.

      Specified by:
      :   `encrypt` in class `ProxyReEncryption`

      Parameters:
      :   `keys` - encryption `Keys`
      :   `message` - `Message` to encrypt
      :   `reencrypt` - `ProxyReEncryption.REENCRYPT` parameter

      Returns:
      :   encrypted `Cipher`


    - #### encrypt

      ```
      public Cipher encrypt(Keys keys,
                            Cipher cipher,
                            ProxyReEncryption.REENCRYPT reencrypt)
      ```

      Description copied from class: `ProxyReEncryption`

      Encrypts the `Cipher` using the public key and `reencrypt`
      parameter. If `reencrypt` is `ProxyReEncryption.REENCRYPT.NO`, then first-level
      encryption is used (i.e., encryption that CANNOT be re-encrypted); else
      second-level (i.e., encryption that CAN be re-encrypted). This issues new
      encryption parameters if `ProxyReEncryption.encMode` is
      `ProxyReEncryption.ENCRYPTION_MODE.DYNAMIC`.

      Specified by:
      :   `encrypt` in class `ProxyReEncryption`

      Parameters:
      :   `keys` - encryption `Keys`
      :   `cipher` - `Cipher` to encrypt
      :   `reencrypt` - `ProxyReEncryption.REENCRYPT` parameter

      Returns:
      :   encrypted `Cipher`


    - #### encrypt

      ```
      public Cipher encrypt(it.unisa.dia.gas.jpbc.Element x,
                            java.lang.String message)
      ```

      Description copied from class: `ProxyReEncryption`

      Encrypts the `message` using the ephemeral key `x`.

      Specified by:
      :   `encrypt` in class `ProxyReEncryption`

      Parameters:
      :   `x` - ephemeral key `Element`
      :   `message` - message to encrypt

      Returns:
      :   encrypted `Cipher`


    - #### encrypt

      ```
      public Cipher encrypt(it.unisa.dia.gas.jpbc.Element x,
                            byte[] message,
                            ProxyReEncryption.MESSAGE_TYPE type)
      ```

      Description copied from class: `ProxyReEncryption`

      Encrypts the `message` using the ephemeral key `x`.

      Specified by:
      :   `encrypt` in class `ProxyReEncryption`

      Parameters:
      :   `x` - ephemeral key `Element`
      :   `message` - message to encrypt
      :   `type` - `ProxyReEncryption.MESSAGE_TYPE.FORMATTED` if the passed bytes are
          the result of a `Message.toBytes()` call, as this
          result is formatted; otherwise, use
          `ProxyReEncryption.MESSAGE_TYPE.UNFORMATTED`

      Returns:
      :   encrypted `Cipher`


    - #### encrypt

      ```
      public Cipher encrypt(it.unisa.dia.gas.jpbc.Element x,
                            Message message)
      ```

      Description copied from class: `ProxyReEncryption`

      Encrypts the `Message` using the ephemeral key `x`.

      Specified by:
      :   `encrypt` in class `ProxyReEncryption`

      Parameters:
      :   `x` - ephemeral key `Element`
      :   `message` - `Message` to encrypt

      Returns:
      :   encrypted `Cipher`


    - #### encrypt

      ```
      public Cipher encrypt(it.unisa.dia.gas.jpbc.Element x,
                            Cipher cipher)
      ```

      Description copied from class: `ProxyReEncryption`

      Encrypts the `Cipher` using the ephemeral key `x`.

      Specified by:
      :   `encrypt` in class `ProxyReEncryption`

      Parameters:
      :   `x` - ephemeral key `Element`
      :   `cipher` - `Cipher` to encrypt

      Returns:
      :   encrypted `Cipher`


    - #### decrypt

      ```
      public Message decrypt(Keys key,
                             Cipher cipher)
      ```

      Description copied from class: `ProxyReEncryption`

      Decrypts the `Cipher` using the provided private key.

      Specified by:
      :   `decrypt` in class `ProxyReEncryption`

      Parameters:
      :   `key` - decryption `Keys`
      :   `cipher` - `Cipher` to decrypt

      Returns:
      :   decrypted `Cipher` as a `Message`


    - #### decrypt

      ```
      public Message decrypt(Keys key,
                             Message message)
      ```

      Description copied from class: `ProxyReEncryption`

      Decrypts the `Message` using the provided private key.

      Specified by:
      :   `decrypt` in class `ProxyReEncryption`

      Parameters:
      :   `key` - decryption `Keys`
      :   `message` - `Message` to decrypt

      Returns:
      :   decrypted `Cipher` as a `Message`


    - #### decryptToCipher

      ```
      public Cipher decryptToCipher(Keys key,
                                    Cipher cipher)
      ```

      Description copied from class: `ProxyReEncryption`

      Decrypts the `Cipher` to a `Cipher` using the provided
      private key.

      Specified by:
      :   `decryptToCipher` in class `ProxyReEncryption`

      Parameters:
      :   `key` - decryption `Keys`
      :   `cipher` - `Cipher` to decrypt

      Returns:
      :   decrypted `Cipher` as a `Cipher`


    - #### decryptIntermediate

      ```
      public Cipher decryptIntermediate(Keys key,
                                        Cipher cipher)
      ```

      Description copied from class: `ProxyReEncryption`

      Decrypts the intermediate layer of a two-party re-encryption
      `Cipher` using the provided private key.

      Specified by:
      :   `decryptIntermediate` in class `ProxyReEncryption`

      Parameters:
      :   `key` - decryption `Keys`
      :   `cipher` - `Cipher` to decrypt at the middle layer

      Returns:
      :   decrypted `Cipher` as a `Cipher`


    - #### decryptDelegatee

      ```
      public Message decryptDelegatee(Keys key,
                                      Cipher cipher)
      ```

      Description copied from class: `ProxyReEncryption`

      Decrypts the delegatee layer of a two-party re-encryption `Cipher`
      using the provided private key.

      Specified by:
      :   `decryptDelegatee` in class `ProxyReEncryption`

      Parameters:
      :   `key` - decryption `Keys`
      :   `cipher` - `Cipher` to decrypt at the final layer

      Returns:
      :   decrypted `Cipher` as a `Message`


    - #### decrypt

      ```
      public Message decrypt(it.unisa.dia.gas.jpbc.Element x,
                             Cipher cipher)
      ```

      Description copied from class: `ProxyReEncryption`

      Decrypts the `Cipher` using the provided ephemeral key `x`.

      Specified by:
      :   `decrypt` in class `ProxyReEncryption`

      Parameters:
      :   `x` - ephemeral key `Element`
      :   `cipher` - `Cipher` to decrypt

      Returns:
      :   decrypted `Cipher` as a `Message`


    - #### decrypt

      ```
      public Message decrypt(it.unisa.dia.gas.jpbc.Element x,
                             Message message)
      ```

      Description copied from class: `ProxyReEncryption`

      Decrypts the `Message` using the provided ephemeral key `x`.

      Specified by:
      :   `decrypt` in class `ProxyReEncryption`

      Parameters:
      :   `x` - ephemeral key `Element`
      :   `message` - `Message` to decrypt

      Returns:
      :   decrypted `Cipher` as a `Message`


    - #### decryptToCipher

      ```
      public Cipher decryptToCipher(it.unisa.dia.gas.jpbc.Element x,
                                    Cipher cipher)
      ```

      Description copied from class: `ProxyReEncryption`

      Decrypts the `Cipher` to a `Cipher` using the provided
      ephemeral key `x`.

      Specified by:
      :   `decryptToCipher` in class `ProxyReEncryption`

      Parameters:
      :   `x` - ephemeral key `Element`
      :   `cipher` - `Cipher` to decrypt

      Returns:
      :   decrypted `Cipher` as a `Cipher`


    - #### reencrypt

      ```
      public Cipher reencrypt(Keys rekey,
                              Cipher cipher)
      ```

      Re-encrypts a `Cipher` using the re-encryption `Keys`.
      Re-encryption is a misnomer in this implementation as the encrypted
      elements do not change, only the decryption keys. These keys are stored
      separate from the elements in this implementation, so the `Cipher`
      returned here simply references the original (`cipher`), but
      includes the new decryption keys.

      Specified by:
      :   `reencrypt` in class `ProxyReEncryption`

      Parameters:
      :   `rekey` - re-encryption `Keys`
      :   `cipher` - `Cipher` to re-encrypt

      Returns:
      :   re-encrypted `Cipher` - does not overwrite `cipher`


    - #### reencrypt

      ```
      public Cipher reencrypt(Keys key,
                              Keys rekey,
                              Cipher cipher)
      ```

      Re-encrypts a two-party `Cipher` using the re-encryption
      `Keys`. Re-encryption is a misnomer in this implementation as the
      encrypted elements do not change, only the decryption keys. These keys
      are stored separate from the elements in this implementation, so the
      `Cipher` returned here simply references the original
      (`cipher`), but includes the new decryption keys.

      Specified by:
      :   `reencrypt` in class `ProxyReEncryption`

      Parameters:
      :   `key` - from `Keys`
      :   `rekey` - re-encryption `Keys`
      :   `cipher` - `Cipher` to re-encrypt

      Returns:
      :   re-encrypted `Cipher` - does not overwrite `cipher`


    - #### sign

      ```
      public Cipher sign(Keys keys,
                         java.lang.String message)
      ```

      Description copied from class: `ProxyReEncryption`

      Signs the `message` using an ephemeral key `x`, returning a
      `Cipher` that is verified by its hash and a private key-based
      decryption parameter (all contained within the `Cipher`).

      Specified by:
      :   `sign` in class `ProxyReEncryption`

      Parameters:
      :   `keys` - `Keys` for signing
      :   `message` - message to sign

      Returns:
      :   the signed `message` as a `Cipher`


    - #### sign

      ```
      public Cipher sign(Keys keys,
                         byte[] message,
                         ProxyReEncryption.MESSAGE_TYPE type)
      ```

      Description copied from class: `ProxyReEncryption`

      Signs the `message` using an ephemeral key `x`, returning a
      `Cipher` that is verified by its hash and a private key-based
      decryption parameter (all contained within the `Cipher`).

      Specified by:
      :   `sign` in class `ProxyReEncryption`

      Parameters:
      :   `keys` - `Keys` for signing
      :   `message` - message to sign
      :   `type` - `ProxyReEncryption.MESSAGE_TYPE.FORMATTED` if the passed bytes are
          the result of a `Message.toBytes()` call, as this
          result is formatted; otherwise, use
          `ProxyReEncryption.MESSAGE_TYPE.UNFORMATTED`

      Returns:
      :   the signed `message` as a `Cipher`


    - #### sign

      ```
      public Cipher sign(Keys keys,
                         Message message)
      ```

      Description copied from class: `ProxyReEncryption`

      Signs the `Message` using an ephemeral key `x`, returning a
      `Cipher` that is verified by its hash and a private key-based
      decryption parameter (all contained within the `Cipher`).

      Specified by:
      :   `sign` in class `ProxyReEncryption`

      Parameters:
      :   `keys` - `Keys` for signing
      :   `message` - `Message` to sign

      Returns:
      :   the signed `Message` as a `Cipher`


    - #### sign

      ```
      public Cipher sign(Keys keys,
                         Cipher cipher)
      ```

      Description copied from class: `ProxyReEncryption`

      Signs the `Cipher` using an ephemeral key `x`, returning a
      `Cipher` that is verified by its hash and a private key-based
      decryption parameter (all contained within the `Cipher`).

      Specified by:
      :   `sign` in class `ProxyReEncryption`

      Parameters:
      :   `keys` - `Keys` for signing
      :   `cipher` - `Cipher` to sign

      Returns:
      :   the signed `Cipher` as a `Cipher`


    - #### verifyAndGet

      ```
      public Message verifyAndGet(Keys keys,
                                  Message message)
      ```

      Description copied from class: `ProxyReEncryption`

      Verifies a signed `Message` using the signer's private key, a
      message hash, and a decryption parameter generated using the signer's
      private key and the message hash. This is an intermediate verification
      step, as the message contents must be the result of a verified
      `Cipher`. The contents of the message are therefore, if compounded
      signatures, a `Cipher`. This returns a verified message.

      Specified by:
      :   `verifyAndGet` in class `ProxyReEncryption`

      Parameters:
      :   `keys` - `Keys` for verifying
      :   `message` - `Message` to verify

      Returns:
      :   the verified `Message` as a `Message`


    - #### verify

      ```
      public boolean verify(Keys keys,
                            Message message)
      ```

      Description copied from class: `ProxyReEncryption`

      Verifies a signed `Message` using the signer's private key, a
      message hash, and a decryption parameter generated using the signer's
      private key and the message hash. This is an intermediate verification
      step, as the message contents must be the result of a verified
      `Cipher`. The contents of the message are therefore, if compounded
      signatures, a `Cipher`. This returns `true` if verified,
      `false` otherwise.

      Specified by:
      :   `verify` in class `ProxyReEncryption`

      Parameters:
      :   `keys` - `Keys` for verifying
      :   `message` - `Message` to verify

      Returns:
      :   `true` if verified, `false` otherwise


    - #### verify

      ```
      public boolean verify(Keys keys,
                            Message message,
                            Message expected)
      ```

      Description copied from class: `ProxyReEncryption`

      Verifies a signed `Message` using the signer's private key, a
      message hash, and a decryption parameter generated using the signer's
      private key and the message hash. This is an intermediate verification
      step, as the message contents must be the result of a verified
      `Cipher`. The contents of the message are therefore, if compounded
      signatures, a `Cipher`. This returns `true` if the contents
      of the signed message match `expected`, `false` otherwise.

      Specified by:
      :   `verify` in class `ProxyReEncryption`

      Parameters:
      :   `keys` - `Keys` for verifying
      :   `message` - `Message` to verify
      :   `expected` - the expected result

      Returns:
      :   `true` if verified, `false` otherwise


    - #### verify

      ```
      public boolean verify(Keys keys,
                            Message message,
                            java.lang.String expected)
      ```

      Description copied from class: `ProxyReEncryption`

      Verifies a signed `Message` using the signer's private key, a
      message hash, and a decryption parameter generated using the signer's
      private key and the message hash. This is an intermediate verification
      step, as the message contents must be the result of a verified
      `Cipher`. The contents of the message are therefore, if compounded
      signatures, a `Cipher`. This returns `true` if the contents
      of the signed message match `expected`, `false` otherwise.

      Specified by:
      :   `verify` in class `ProxyReEncryption`

      Parameters:
      :   `keys` - `Keys` for verifying
      :   `message` - `Message` to verify
      :   `expected` - the expected result

      Returns:
      :   `true` if verified, `false` otherwise


    - #### verify

      ```
      public boolean verify(Keys keys,
                            Message message,
                            byte[] expected)
      ```

      Description copied from class: `ProxyReEncryption`

      Verifies a signed `Message` using the signer's private key, a
      message hash, and a decryption parameter generated using the signer's
      private key and the message hash. This is an intermediate verification
      step, as the message contents must be the result of a verified
      `Cipher`. The contents of the message are therefore, if compounded
      signatures, a `Cipher`. This returns `true` if the contents
      of the signed message match `expected`, `false` otherwise.

      Specified by:
      :   `verify` in class `ProxyReEncryption`

      Parameters:
      :   `keys` - `Keys` for verifying
      :   `message` - `Message` to verify
      :   `expected` - the expected result

      Returns:
      :   `true` if verified, `false` otherwise


    - #### verifyAndGet

      ```
      public Message verifyAndGet(Keys keys,
                                  Cipher cipher)
      ```

      Description copied from class: `ProxyReEncryption`

      Verifies a signed `Cipher` using the signer's private key, a
      message hash, and a decryption parameter generated using the signer's
      private key and the message hash. This returns a verified message.

      Specified by:
      :   `verifyAndGet` in class `ProxyReEncryption`

      Parameters:
      :   `keys` - `Keys` for verifying
      :   `cipher` - `Cipher` to verify

      Returns:
      :   the verified `Cipher` as a `Message`


    - #### verify

      ```
      public boolean verify(Keys keys,
                            Cipher cipher)
      ```

      Description copied from class: `ProxyReEncryption`

      Verifies a signed `Cipher` using the signer's private key, a
      message hash, and a decryption parameter generated using the signer's
      private key and the message hash. This returns `true` if verified,
      `false` otherwise.

      Specified by:
      :   `verify` in class `ProxyReEncryption`

      Parameters:
      :   `keys` - `Keys` for verifying
      :   `cipher` - `Cipher` to verify

      Returns:
      :   `true` if verified, `false` otherwise


    - #### verify

      ```
      public boolean verify(Keys keys,
                            Cipher cipher,
                            Message expected)
      ```

      Description copied from class: `ProxyReEncryption`

      Verifies a signed `Cipher` using the signer's private key, a
      message hash, and a decryption parameter generated using the signer's
      private key and the message hash. This returns `true` if the
      contents of the signed message match `expected`, `false`
      otherwise.

      Specified by:
      :   `verify` in class `ProxyReEncryption`

      Parameters:
      :   `keys` - `Keys` for verifying
      :   `cipher` - `Cipher` to verify
      :   `expected` - the expected result

      Returns:
      :   `true` if verified, `false` otherwise


    - #### verify

      ```
      public boolean verify(Keys keys,
                            Cipher cipher,
                            java.lang.String expected)
      ```

      Description copied from class: `ProxyReEncryption`

      Verifies a signed `Cipher` using the signer's private key, a
      message hash, and a decryption parameter generated using the signer's
      private key and the message hash. This returns `true` if the
      contents of the signed message match `expected`, `false`
      otherwise.

      Specified by:
      :   `verify` in class `ProxyReEncryption`

      Parameters:
      :   `keys` - `Keys` for verifying
      :   `cipher` - `Cipher` to verify
      :   `expected` - the expected result

      Returns:
      :   `true` if verified, `false` otherwise


    - #### verify

      ```
      public boolean verify(Keys keys,
                            Cipher cipher,
                            byte[] expected)
      ```

      Description copied from class: `ProxyReEncryption`

      Verifies a signed `Cipher` using the signer's private key, a
      message hash, and a decryption parameter generated using the signer's
      private key and the message hash. This returns `true` if the
      contents of the signed message match `expected`, `false`
      otherwise.

      Specified by:
      :   `verify` in class `ProxyReEncryption`

      Parameters:
      :   `keys` - `Keys` for verifying
      :   `cipher` - `Cipher` to verify
      :   `expected` - the expected result

      Returns:
      :   `true` if verified, `false` otherwise


    - #### sign

      ```
      public Cipher sign(Keys sign,
                         Keys encrypt,
                         java.lang.String message)
      ```

      Description copied from class: `ProxyReEncryption`

      Encrypt-signs the `message` using an ephemeral key `x`,
      returning a `Cipher` that is decrypt-verified by its hash and a
      decryption parameter constructed using the signer's private key and
      recipients public key (all contained within the `Cipher`). This
      does NOT issue new encryption parameters, even if `ProxyReEncryption.encMode` is
      `ProxyReEncryption.ENCRYPTION_MODE.DYNAMIC`, as it is being used for encrypting an
      EXISTING message, not a new one.

      Specified by:
      :   `sign` in class `ProxyReEncryption`

      Parameters:
      :   `sign` - signer's `Keys`
      :   `encrypt` - recipient's `Keys` for encrypting
      :   `message` - message to sign

      Returns:
      :   the encrypt-signed `message` as a `Cipher`


    - #### sign

      ```
      public Cipher sign(Keys sign,
                         Keys encrypt,
                         byte[] message,
                         ProxyReEncryption.MESSAGE_TYPE type)
      ```

      Description copied from class: `ProxyReEncryption`

      Encrypt-signs the `message` using an ephemeral key `x`,
      returning a `Cipher` that is decrypt-verified by its hash and a
      decryption parameter constructed using the signer's private key and
      recipients public key (all contained within the `Cipher`). This
      does NOT issue new encryption parameters, even if `ProxyReEncryption.encMode` is
      `ProxyReEncryption.ENCRYPTION_MODE.DYNAMIC`, as it is being used for encrypting an
      EXISTING message, not a new one.

      Specified by:
      :   `sign` in class `ProxyReEncryption`

      Parameters:
      :   `sign` - signer's `Keys`
      :   `encrypt` - recipient's `Keys` for encrypting
      :   `message` - message to sign
      :   `type` - `ProxyReEncryption.MESSAGE_TYPE.FORMATTED` if the passed bytes are
          the result of a `Message.toBytes()` call, as this
          result is formatted; otherwise, use
          `ProxyReEncryption.MESSAGE_TYPE.UNFORMATTED`

      Returns:
      :   the encrypt-signed `message` as a `Cipher`


    - #### sign

      ```
      public Cipher sign(Keys sign,
                         Keys encrypt,
                         Message message)
      ```

      Description copied from class: `ProxyReEncryption`

      Encrypt-signs the `Message` using an ephemeral key `x`,
      returning a `Cipher` that is decrypt-verified by its hash and a
      decryption parameter constructed using the signer's private key and
      recipients public key (all contained within the `Cipher`). This
      does NOT issue new encryption parameters, even if `ProxyReEncryption.encMode` is
      `ProxyReEncryption.ENCRYPTION_MODE.DYNAMIC`, as it is being used for encrypting an
      EXISTING message, not a new one.

      Specified by:
      :   `sign` in class `ProxyReEncryption`

      Parameters:
      :   `sign` - sign `Keys`
      :   `encrypt` - encryption `Keys`
      :   `message` - `Message` to encrypt

      Returns:
      :   the encrypt-signed `Message` as a `Cipher`


    - #### sign

      ```
      public Cipher sign(Keys sign,
                         Keys encrypt,
                         Cipher cipher)
      ```

      Description copied from class: `ProxyReEncryption`

      Encrypt-signs the `Cipher` using an ephemeral key `x`,
      returning a `Cipher` that is decrypt-verified by its hash and a
      decryption parameter constructed using the signer's private key and
      recipients public key (all contained within the `Cipher`). This
      does NOT issue new encryption parameters, even if `ProxyReEncryption.encMode` is
      `ProxyReEncryption.ENCRYPTION_MODE.DYNAMIC`, as it is being used for encrypting an
      EXISTING message, not a new one.

      Specified by:
      :   `sign` in class `ProxyReEncryption`

      Parameters:
      :   `sign` - sign `Keys`
      :   `encrypt` - encryption `Keys`
      :   `cipher` - `Cipher` to encrypt

      Returns:
      :   the encrypt-signed `Cipher` as a `Cipher`


    - #### verifyAndGet

      ```
      public Message verifyAndGet(Keys verify,
                                  Keys decrypt,
                                  Message message)
      ```

      Description copied from class: `ProxyReEncryption`

      Decrypt-verifies a signed `Message` using the signer's private key,
      a message hash, and a decryption parameter generated using the signer's
      private key and the message hash. This is an intermediate verification
      step, as the message contents must be the result of a decrypt-verified
      `Cipher`. The contents of the message are therefore, if compounded
      signatures, a `Cipher`. This returns a decrypt-verified message.

      Specified by:
      :   `verifyAndGet` in class `ProxyReEncryption`

      Parameters:
      :   `verify` - verify `Keys`
      :   `decrypt` - decryption `Keys`
      :   `message` - `Message` to decrypt

      Returns:
      :   decrypted-verified `Message` as a `Message`


    - #### verify

      ```
      public boolean verify(Keys verify,
                            Keys decrypt,
                            Message message)
      ```

      Description copied from class: `ProxyReEncryption`

      Decrypt-verifies a signed `Message` using the signer's private key,
      a message hash, and a decryption parameter generated using the signer's
      private key and the message hash. This is an intermediate verification
      step, as the message contents must be the result of a decrypt-verified
      `Cipher`. The contents of the message are therefore, if compounded
      signatures, a `Cipher`. This returns `true` if verified,
      `false` otherwise.

      Specified by:
      :   `verify` in class `ProxyReEncryption`

      Parameters:
      :   `verify` - verify `Keys`
      :   `decrypt` - decryption `Keys`
      :   `message` - `Message` to decrypt

      Returns:
      :   `true` if verified, `false` otherwise


    - #### verify

      ```
      public boolean verify(Keys verify,
                            Keys decrypt,
                            Message message,
                            Message expected)
      ```

      Description copied from class: `ProxyReEncryption`

      Decrypt-verifies a signed `Message` using the signer's private key,
      a message hash, and a decryption parameter generated using the signer's
      private key and the message hash. This is an intermediate verification
      step, as the message contents must be the result of a decrypt-verified
      `Cipher`. The contents of the message are therefore, if compounded
      signatures, a `Cipher`. This returns `true` if the contents
      of the signed message match `expected`, `false` otherwise.

      Specified by:
      :   `verify` in class `ProxyReEncryption`

      Parameters:
      :   `verify` - verify `Keys`
      :   `decrypt` - decryption `Keys`
      :   `message` - `Message` to decrypt
      :   `expected` - the expected result

      Returns:
      :   `true` if verified, `false` otherwise


    - #### verify

      ```
      public boolean verify(Keys verify,
                            Keys decrypt,
                            Message message,
                            java.lang.String expected)
      ```

      Description copied from class: `ProxyReEncryption`

      Decrypt-verifies a signed `Message` using the signer's private key,
      a message hash, and a decryption parameter generated using the signer's
      private key and the message hash. This is an intermediate verification
      step, as the message contents must be the result of a decrypt-verified
      `Cipher`. The contents of the message are therefore, if compounded
      signatures, a `Cipher`. This returns `true` if the contents
      of the signed message match `expected`, `false` otherwise.

      Specified by:
      :   `verify` in class `ProxyReEncryption`

      Parameters:
      :   `verify` - verify `Keys`
      :   `decrypt` - decryption `Keys`
      :   `message` - `Message` to decrypt
      :   `expected` - the expected result

      Returns:
      :   `true` if verified, `false` otherwise


    - #### verify

      ```
      public boolean verify(Keys verify,
                            Keys decrypt,
                            Message message,
                            byte[] expected)
      ```

      Description copied from class: `ProxyReEncryption`

      Decrypt-verifies a signed `Message` using the signer's private key,
      a message hash, and a decryption parameter generated using the signer's
      private key and the message hash. This is an intermediate verification
      step, as the message contents must be the result of a decrypt-verified
      `Cipher`. The contents of the message are therefore, if compounded
      signatures, a `Cipher`. This returns `true` if the contents
      of the signed message match `expected`, `false` otherwise.

      Specified by:
      :   `verify` in class `ProxyReEncryption`

      Parameters:
      :   `verify` - verify `Keys`
      :   `decrypt` - decryption `Keys`
      :   `message` - `Message` to decrypt
      :   `expected` - the expected result

      Returns:
      :   `true` if verified, `false` otherwise


    - #### verifyAndGet

      ```
      public Message verifyAndGet(Keys verify,
                                  Keys decrypt,
                                  Cipher cipher)
      ```

      Description copied from class: `ProxyReEncryption`

      Decrypt-verifies a signed `Cipher` using the signer's private key,
      a message hash, and a decryption parameter generated using the signer's
      private key and the message hash. This returns a decrypt-verified
      message.

      Specified by:
      :   `verifyAndGet` in class `ProxyReEncryption`

      Parameters:
      :   `verify` - verify `Keys`
      :   `decrypt` - decryption `Keys`
      :   `cipher` - `Cipher` to decrypt

      Returns:
      :   decrypted-verified `Cipher` as a `Message`


    - #### verify

      ```
      public boolean verify(Keys verify,
                            Keys decrypt,
                            Cipher cipher)
      ```

      Description copied from class: `ProxyReEncryption`

      Decrypt-verifies a signed `Cipher` using the signer's private key,
      a message hash, and a decryption parameter generated using the signer's
      private key and the message hash. This returns `true` if verified,
      `false` otherwise.

      Specified by:
      :   `verify` in class `ProxyReEncryption`

      Parameters:
      :   `verify` - verify `Keys`
      :   `decrypt` - decryption `Keys`
      :   `cipher` - `Cipher` to decrypt

      Returns:
      :   `true` if verified, `false` otherwise


    - #### verify

      ```
      public boolean verify(Keys verify,
                            Keys decrypt,
                            Cipher cipher,
                            Message expected)
      ```

      Description copied from class: `ProxyReEncryption`

      Decrypt-verifies a signed `Cipher` using the signer's private key,
      a message hash, and a decryption parameter generated using the signer's
      private key and the message hash. This returns `true` if the
      contents of the signed message match `expected`, `false`
      otherwise.

      Specified by:
      :   `verify` in class `ProxyReEncryption`

      Parameters:
      :   `verify` - verify `Keys`
      :   `decrypt` - decryption `Keys`
      :   `cipher` - `Cipher` to decrypt
      :   `expected` - the expected result

      Returns:
      :   `true` if verified, `false` otherwise


    - #### verify

      ```
      public boolean verify(Keys verify,
                            Keys decrypt,
                            Cipher cipher,
                            java.lang.String expected)
      ```

      Description copied from class: `ProxyReEncryption`

      Decrypt-verifies a signed `Cipher` using the signer's private key,
      a message hash, and a decryption parameter generated using the signer's
      private key and the message hash. This returns `true` if the
      contents of the signed message match `expected`, `false`
      otherwise.

      Specified by:
      :   `verify` in class `ProxyReEncryption`

      Parameters:
      :   `verify` - verify `Keys`
      :   `decrypt` - decryption `Keys`
      :   `cipher` - `Cipher` to decrypt
      :   `expected` - the expected result

      Returns:
      :   `true` if verified, `false` otherwise


    - #### verify

      ```
      public boolean verify(Keys verify,
                            Keys decrypt,
                            Cipher cipher,
                            byte[] expected)
      ```

      Description copied from class: `ProxyReEncryption`

      Decrypt-verifies a signed `Cipher` using the signer's private key,
      a message hash, and a decryption parameter generated using the signer's
      private key and the message hash. This returns `true` if the
      contents of the signed message match `expected`, `false`
      otherwise.

      Specified by:
      :   `verify` in class `ProxyReEncryption`

      Parameters:
      :   `verify` - verify `Keys`
      :   `decrypt` - decryption `Keys`
      :   `cipher` - `Cipher` to decrypt
      :   `expected` - the expected result

      Returns:
      :   `true` if verified, `false` otherwise


    - #### decryptVerifyImpl

      ```
      public Message decryptVerifyImpl(Keys verify,
                                       Keys decrypt,
                                       Message cipher)
      ```

      Decrypt-verifies the `cipher` using the private key of the
      recipient (`decrypt`) and public key of the signer
      (`verify`), returning the decrypt-verified cipher as a
      `Message`. Decrypt-verified results are returned as
      `Message`s with `Cipher`s encoded as bytes to preserve the
      entire structure of the `Cipher`. That is, the parameters stored in
      the `Cipher` are used during that process, but for this, the
      parameters are used to validate the next. Thus, the full structure must
      be preserved.

      Parameters:
      :   `verify` - verify `Keys`
      :   `decrypt` - decryption `Keys`
      :   `cipher` - cipher as a `Message` to decrypt

      Returns:
      :   decrypted-verified `cipher` as a `Message`


    - #### scale

      ```
      public void scale(Cipher cipher,
                        it.unisa.dia.gas.jpbc.Element scalar,
                        ProxyReEncryption.SCALE scaleType)
      ```

      Description copied from class: `ProxyReEncryption`

      Transforms the encryption key of the `Cipher` from `from` to
      `to` by way of simple scaling.

      Specified by:
      :   `scale` in class `ProxyReEncryption`

      Parameters:
      :   `cipher` - `Cipher` to transform via scaling
      :   `scalar` - scalar `Element`
      :   `scaleType` - `ProxyReEncryption.SCALE`


    - #### scale

      ```
      public void scale(Message message,
                        it.unisa.dia.gas.jpbc.Element scalar,
                        ProxyReEncryption.SCALE scaleType)
      ```

      Description copied from class: `ProxyReEncryption`

      Transforms the encryption key of the `Message` from `from` to
      `to` by way of simple scaling.

      Specified by:
      :   `scale` in class `ProxyReEncryption`

      Parameters:
      :   `message` - `Message` to transform via scaling
      :   `scalar` - scalar `Element`
      :   `scaleType` - `ProxyReEncryption.SCALE`


    - #### scale

      ```
      public void scale(Cipher cipher,
                        it.unisa.dia.gas.jpbc.Element from,
                        it.unisa.dia.gas.jpbc.Element to,
                        ProxyReEncryption.SCALE scaleType)
      ```

      Description copied from class: `ProxyReEncryption`

      Transforms the encryption key of the `Cipher` from `from` to
      `to` by way of simple scaling.

      Specified by:
      :   `scale` in class `ProxyReEncryption`

      Parameters:
      :   `cipher` - `Cipher` to transform via scaling
      :   `from` - from `Element`
      :   `to` - to `Element`
      :   `scaleType` - `ProxyReEncryption.SCALE`


    - #### scale

      ```
      public void scale(Message message,
                        it.unisa.dia.gas.jpbc.Element from,
                        it.unisa.dia.gas.jpbc.Element to,
                        ProxyReEncryption.SCALE scaleType)
      ```

      Description copied from class: `ProxyReEncryption`

      Transforms the encryption key of the `Message` from `from` to
      `to` by way of simple scaling.

      Specified by:
      :   `scale` in class `ProxyReEncryption`

      Parameters:
      :   `message` - `Message` to transform via scaling
      :   `from` - from `Element`
      :   `to` - to `Element`
      :   `scaleType` - `ProxyReEncryption.SCALE`


    - #### toCipher

      ```
      public Cipher toCipher(byte[] bytes)
      ```

      Description copied from class: `ProxyReEncryption`

      Converts a `byte[]` to a `Cipher`.

      Specified by:
      :   `toCipher` in class `ProxyReEncryption`

      Parameters:
      :   `bytes` - `byte[]` to convert

      Returns:
      :   a `Cipher` object


    - #### toMessage

      ```
      public Message toMessage(java.lang.String message)
      ```

      Description copied from class: `ProxyReEncryption`

      Converts a `String` to a `Message`.

      Specified by:
      :   `toMessage` in class `ProxyReEncryption`

      Parameters:
      :   `message` - `String` to convert

      Returns:
      :   `String` as a `Message`


    - #### toMessage

      ```
      public Message toMessage(byte[] message,
                               ProxyReEncryption.MESSAGE_TYPE type)
      ```

      Description copied from class: `ProxyReEncryption`

      Converts a `byte[]` to a `Message`. If `type` is
      `ProxyReEncryption.MESSAGE_TYPE.FORMATTED`, the `message` will be treated as a
      formatted message (i.e., the result of a `Message.toBytes()` call)
      and the structure recovered; otherwise, it will treat the `message`
      as pure data.

      Specified by:
      :   `toMessage` in class `ProxyReEncryption`

      Parameters:
      :   `message` - `Message` as `byte[]` to convert
      :   `type` - `ProxyReEncryption.MESSAGE_TYPE.FORMATTED` if the passed bytes are
          the result of a `Message.toBytes()` call, as this
          result is formatted; otherwise, use
          `ProxyReEncryption.MESSAGE_TYPE.UNFORMATTED`

      Returns:
      :   `byte[]` as a `Message`


    - #### toMessage

      ```
      public Message toMessage(byte[] message,
                               int bytePadding)
      ```

      Description copied from class: `ProxyReEncryption`

      Converts a `byte[]` to a `Message` with padding. The
      `message` is always treated as `ProxyReEncryption.MESSAGE_TYPE.UNFORMATTED`.
      Use this method if the data are arbitrary bytes that are not bound to
      `ASCII`. When converted to an element, they may be outside the
      range of the `order` of the elliptic curve. As such, they will be
      subject to a `mod` operation, rending the data unrecoverable. This
      allows for padding to be added in the for of a `(byte)0` to the
      beginning of each element or element set component (e.g., `X/Y`
      coordinates). This is generally used when taking the results of a
      `Message` or `Cipher` and storing it as message bytes.

      Specified by:
      :   `toMessage` in class `ProxyReEncryption`

      Parameters:
      :   `message` - `Message` as `byte[]` to convert
      :   `bytePadding` - the number of leading bytes in a converted element
          (either single chunk or X/Y pairs) to pad with a
          `0`

      Returns:
      :   `byte[]` as a `Message` or `null` for error


Skip navigation links


- Package
- Class
- Use
- Tree
- Deprecated
- Index
- Help

- Prev Class
- Next Class

- Frames
- No Frames

- All Classes

- Summary:
- Nested |
- Field |
- Constr |
- Method

- Detail:
- Field |
- Constr |
- Method
