# Supplementary material for: A Blockchain Framework for Patient-Centered Health Records and Exchange (HealthChain): Evaluation and Proof-of-Concept Study
Source: J Med Internet Res. 2019 Aug 31;21(8):e13592. doi: 10.2196/13592 (PMC6743266; doi:10.2196/13592)
Supplement: Multimedia Appendix 3 [file jmir_v21i8e13592_app3.zip › ProxyReEncryption/javadoc/edu/ecu/hsim/ray/proxyreencryption/AFGH.LENGTH.html]

AFGH.LENGTH


JavaScript is disabled on your browser.


Skip navigation links


- Package
- Class
- Use
- Tree
- Deprecated
- Index
- Help

- Prev Class
- Next Class

- Frames
- No Frames

- All Classes

- Summary:
- Nested |
- Enum Constants |
- Field |
- Method

- Detail:
- Enum Constants |
- Field |
- Method


edu.ecu.hsim.ray.proxyreencryption

## Enum AFGH.LENGTH

- java.lang.Object
- - java.lang.Enum<AFGH.LENGTH>
  - - edu.ecu.hsim.ray.proxyreencryption.AFGH.LENGTH

- All Implemented Interfaces:
  :   java.io.Serializable, java.lang.Comparable<AFGH.LENGTH>

  Enclosing class:
  :   AFGH

  ---

    

  ```
  public static enum AFGH.LENGTH
  extends java.lang.Enum<AFGH.LENGTH>
  ```

  The employed curve is a Type A pairing Montgomery curve with embedding
  degree of \(k=2\). Since the curve is super-singular, the hardness is
  tied not to the ECDLP (i.e., the group order bits \(r\)), but the DLP
  (i.e., the base field size bits \(q\)). For pairing cryptography, \(k\)
  must be very low, otherwise operations on \(GT\) (which is a subgroup of
  \(F\_{r^{k}}\)) will be infeasible. Thus, \(q\) must be a large number to
  compensate, as \(qk\) equals the discrete log security in bits.
  Additionally, as \(r\equiv3\mod4\), the number of rational points on
  elliptic curve \(E\) given field \(F\_{r}\), \(E\left(F\_{r}\right)\), is
  \(\#E\left(F\_{r}\right)=r+1\).

  There are five supported elliptic curve key lengths^1^:

  - EC\_160
    - Group order: 160-bits, equivalent in strength to 2TDEA^2^ and RSA-1024
    - Base size: 512-bits (512k = 512\*2 = 1024-bits of discrete log strength)
  - EC\_224
    - Group order: 224-bits, equivalent in strength to 3TDEA^3^ and RSA-2048
    - Base size: 1024-bits (1024k = 1024\*2 = 2048-bits of discrete log strength)
  - EC\_256
    - Group order: 256-bits, equivalent in strength to AES-128 and RSA-3072
    - Base size: 1536-bits (1536k = 1536\*2 = 3072-bits of discrete log strength)
  - EC\_384
    - Group order: 384-bits, equivalent in strength to AES-192 and RSA-7680
    - Base size: 3840-bits (3840k = 3840\*2 = 7680-bits of discrete log strength)
  - EC\_521^4^
    - Group order: 521-bits, equivalent in strength to AES-256 and RSA-15360
    - Base size: 7680-bits (7680k = 7680\*2 = 15360-bits of discrete log strength)
  ^1^ 
  NIST reference  
  ^2^ 2TDEA: two-key, triple data encryption algorithm (DES) - 80-bits of strength  
  ^3^ 3TDEA: three-key, triple data encryption algorithm (DES) - 112-bits of strength  
  ^4^ It is 521, not 512 as it relates to Solinas primes.

- - ### Enum Constant Summary

    Enum Constants

    Enum Constant and Description

    `EC_160`

    160 group bits and 512 base size.

    `EC_224`

    224 group bits and 1024 base size.

    `EC_256`

    256 group bits and 1536 base size.

    `EC_384`

    384 group bits and 3840 base size.

    `EC_521`

    521 group bits and 7680 base size.
  - ### Method Summary

    All Methods Static Methods Concrete Methods

    | Modifier and Type | Method and Description |
    | --- | --- |
    | `static AFGH.LENGTH` | `valueOf(java.lang.String name)` Returns the enum constant of this type with the specified name. |
    | `static AFGH.LENGTH[]` | `values()` Returns an array containing the constants of this enum type, in the order they are declared. |

    - ### Methods inherited from class java.lang.Enum

      `clone, compareTo, equals, finalize, getDeclaringClass, hashCode, name, ordinal, toString, valueOf`
    - ### Methods inherited from class java.lang.Object

      `getClass, notify, notifyAll, wait, wait, wait`

- - ### Enum Constant Detail


    - #### EC\_160

      ```
      public static final AFGH.LENGTH EC_160
      ```

      160 group bits and 512 base size.


    - #### EC\_224

      ```
      public static final AFGH.LENGTH EC_224
      ```

      224 group bits and 1024 base size.


    - #### EC\_256

      ```
      public static final AFGH.LENGTH EC_256
      ```

      256 group bits and 1536 base size.


    - #### EC\_384

      ```
      public static final AFGH.LENGTH EC_384
      ```

      384 group bits and 3840 base size.


    - #### EC\_521

      ```
      public static final AFGH.LENGTH EC_521
      ```

      521 group bits and 7680 base size.
  - ### Method Detail


    - #### values

      ```
      public static AFGH.LENGTH[] values()
      ```

      Returns an array containing the constants of this enum type, in
      the order they are declared. This method may be used to iterate
      over the constants as follows:

      ```
      for (AFGH.LENGTH c : AFGH.LENGTH.values())
          System.out.println(c);
      ```

      Returns:
      :   an array containing the constants of this enum type, in the order they are declared


    - #### valueOf

      ```
      public static AFGH.LENGTH valueOf(java.lang.String name)
      ```

      Returns the enum constant of this type with the specified name.
      The string must match *exactly* an identifier used to declare an
      enum constant in this type. (Extraneous whitespace characters are
      not permitted.)

      Parameters:
      :   `name` - the name of the enum constant to be returned.

      Returns:
      :   the enum constant with the specified name

      Throws:
      :   `java.lang.IllegalArgumentException` - if this enum type has no constant with the specified name
      :   `java.lang.NullPointerException` - if the argument is null


Skip navigation links


- Package
- Class
- Use
- Tree
- Deprecated
- Index
- Help

- Prev Class
- Next Class

- Frames
- No Frames

- All Classes

- Summary:
- Nested |
- Enum Constants |
- Field |
- Method

- Detail:
- Enum Constants |
- Field |
- Method
