# Supplementary material for: A Blockchain Framework for Patient-Centered Health Records and Exchange (HealthChain): Evaluation and Proof-of-Concept Study
Source: J Med Internet Res. 2019 Aug 31;21(8):e13592. doi: 10.2196/13592 (PMC6743266; doi:10.2196/13592)
Supplement: Multimedia Appendix 3 [file jmir_v21i8e13592_app3.zip › ProxyReEncryption/javadoc/edu/ecu/hsim/ray/proxyreencryption/Cipher.html]

Cipher


JavaScript is disabled on your browser.


Skip navigation links


- Package
- Class
- Use
- Tree
- Deprecated
- Index
- Help

- Prev Class
- Next Class

- Frames
- No Frames

- All Classes

- Summary:
- Nested |
- Field |
- Constr |
- Method

- Detail:
- Field |
- Constr |
- Method


edu.ecu.hsim.ray.proxyreencryption

## Class Cipher

- java.lang.Object
- - edu.ecu.hsim.ray.proxyreencryption.Cipher

- ---

    

  ```
  public class Cipher
  extends java.lang.Object
  ```

  Stores enciphered data as a list of elements and lengths. Each
  `Element` has a maximum capacity of the length in bytes of the
  `Field` `ProxyReEncryption.G2`. Appending to an existing
  `Cipher` is trivially accomplished via `add(Element, int)`.

- - ### Nested Class Summary

    Nested Classes

    | Modifier and Type | Class and Description |
    | --- | --- |
    | `protected static class` | `Cipher.LEVEL` Flags each `Cipher` with the appropriate level. |
    | `static class` | `Cipher.PAIRING_TYPE` When loading a pairing `Element`, one can specify if it is a formatted or unformatted byte array. |
  - ### Field Summary

    Fields

    | Modifier and Type | Field and Description |
    | --- | --- |
    | `protected it.unisa.dia.gas.jpbc.Element` | `decryptionParameter` Decryption parameter - re-encryption/two-party/sign/encrypt-sign |
    | `protected edu.ecu.hsim.ray.proxyreencryption.Elements` | `elements` Cipher `Elements`. |
    | `protected Cipher.LEVEL` | `level` `Cipher.LEVEL` of cipher. |
    | `protected ProxyReEncryption` | `proxy` `ProxyReEncryption` object. |
    | `protected it.unisa.dia.gas.jpbc.Element` | `reencryptionPairing` Re-encryption pairing - re-encryption/two-party/sign/encrypt-sign |
    | `protected it.unisa.dia.gas.jpbc.Element` | `reencryptionScalar` Re-encryption scalar - two-party/sign/encrypt-sign |
    | `protected it.unisa.dia.gas.jpbc.Element` | `signedHash` Signed hash - two-party/sign/encrypt-sign |
  - ### Constructor Summary

    Constructors

    | Modifier | Constructor and Description |
    | --- | --- |
    | `protected` | `Cipher(ProxyReEncryption proxy, Cipher.LEVEL level)` Constructor. |
    | `protected` | `Cipher(ProxyReEncryption proxy, Cipher.LEVEL level, Cipher cipher, it.unisa.dia.gas.jpbc.Element reencryptionPairing)` Re-encryption constructor. |
    | `protected` | `Cipher(ProxyReEncryption proxy, Cipher.LEVEL level, Cipher cipher, it.unisa.dia.gas.jpbc.Element reencryptionScalar, it.unisa.dia.gas.jpbc.Element reencryptionPairing)` Re-encryption constructor for two-party decryption This sets the intermediate's scalar and delegatee's re-encryption, which is used during the decryption process by the recipient. |
    | `protected` | `Cipher(ProxyReEncryption proxy, Cipher.LEVEL level, it.unisa.dia.gas.jpbc.Element reencryptionPairing)` Re-encryption constructor. |
    | `protected` | `Cipher(ProxyReEncryption proxy, Cipher.LEVEL level, it.unisa.dia.gas.jpbc.Element signedHash, it.unisa.dia.gas.jpbc.Element decryptionParameter)` Re-encryption constructor for sign/encrypt-sign constructor. |
  - ### Method Summary

    All Methods Instance Methods Concrete Methods

    | Modifier and Type | Method and Description |
    | --- | --- |
    | `protected void` | `add(it.unisa.dia.gas.jpbc.Element element, int length)` Add an `Element`. |
    | `boolean` | `canReEncrypt()` Returns `true` if can re-encrypt, `false` otherwise. |
    | `it.unisa.dia.gas.jpbc.Element` | `getDecryptionParameter()` Returns the decryption parameter element (for decrypt-verify). |
    | `java.util.List<it.unisa.dia.gas.jpbc.Element>` | `getElements()` Returns the list of elements. |
    | `java.util.List<java.lang.Integer>` | `getLengths()` Returns the list of lengths. |
    | `Cipher.LEVEL` | `getLevel()` Returns the `Cipher.LEVEL`. |
    | `it.unisa.dia.gas.jpbc.Element` | `getReencryptionPairing()` Returns the re-encryption pairing element. |
    | `it.unisa.dia.gas.jpbc.Element` | `getReencryptionScalar()` Returns the intermediate re-encryption scalar element. |
    | `it.unisa.dia.gas.jpbc.Element` | `getSignedHash()` Returns the hash for signed/encrypt-signed messages. |
    | `void` | `setDecryptionParameter(it.unisa.dia.gas.jpbc.Element element)` Sets the X/Y values for the decryption parameter pairing element. |
    | `void` | `setReencryptionPairing(byte[] bytes)` Sets the X/Y values for the re-encryption pairing element. |
    | `void` | `setReencryptionPairing(byte[] bytes, Cipher.PAIRING_TYPE type)` Sets the X/Y values for the re-encryption pairing element. |
    | `void` | `setReencryptionPairing(it.unisa.dia.gas.jpbc.Element element)` Sets the X/Y values for the re-encryption pairing element. |
    | `void` | `setReencryptionScalar(byte[] bytes)` Sets the value of the intermediate re-encryption scalar element. |
    | `void` | `setReencryptionScalar(byte[] bytes, Cipher.PAIRING_TYPE type)` Sets the value of the intermediate re-encryption scalar element. |
    | `void` | `setReencryptionScalar(it.unisa.dia.gas.jpbc.Element element)` Sets the value of the intermediate re-encryption scalar element. |
    | `void` | `setSignedHash(it.unisa.dia.gas.jpbc.Element element)` Sets the hash value of a signed/encrypt-signed message. |
    | `void` | `setToTwoParty()` Sets the `Cipher.LEVEL` to `Cipher.LEVEL.TWOPARTY`. |
    | `byte[]` | `toBytes()` Converts a `Cipher` to a `byte[]`. |
    | `byte[]` | `toHashBytes()` Converts a `Cipher` to a `byte[]` suitable for hashing. |
    | `java.lang.String` | `toString()` |
    | `void` | `write(java.lang.String file)` Writes `this` to a file. |

    - ### Methods inherited from class java.lang.Object

      `clone, equals, finalize, getClass, hashCode, notify, notifyAll, wait, wait, wait`

- - ### Field Detail


    - #### level

      ```
      protected Cipher.LEVEL level
      ```

      `Cipher.LEVEL` of cipher.


    - #### elements

      ```
      protected edu.ecu.hsim.ray.proxyreencryption.Elements elements
      ```

      Cipher `Elements`.


    - #### proxy

      ```
      protected ProxyReEncryption proxy
      ```

      `ProxyReEncryption` object.


    - #### reencryptionPairing

      ```
      protected it.unisa.dia.gas.jpbc.Element reencryptionPairing
      ```

      Re-encryption pairing - re-encryption/two-party/sign/encrypt-sign


    - #### decryptionParameter

      ```
      protected it.unisa.dia.gas.jpbc.Element decryptionParameter
      ```

      Decryption parameter - re-encryption/two-party/sign/encrypt-sign


    - #### reencryptionScalar

      ```
      protected it.unisa.dia.gas.jpbc.Element reencryptionScalar
      ```

      Re-encryption scalar - two-party/sign/encrypt-sign


    - #### signedHash

      ```
      protected it.unisa.dia.gas.jpbc.Element signedHash
      ```

      Signed hash - two-party/sign/encrypt-sign
  - ### Constructor Detail


    - #### Cipher

      ```
      protected Cipher(ProxyReEncryption proxy,
                       Cipher.LEVEL level)
      ```

      Constructor.

      Parameters:
      :   `proxy` - `ProxyReEncryption`
      :   `level` - `Cipher.LEVEL` level


    - #### Cipher

      ```
      protected Cipher(ProxyReEncryption proxy,
                       Cipher.LEVEL level,
                       it.unisa.dia.gas.jpbc.Element reencryptionPairing)
      ```

      Re-encryption constructor.

      Parameters:
      :   `proxy` - `ProxyReEncryption`
      :   `level` - `Cipher.LEVEL` level
      :   `reencryptionPairing` - re-encryption pairing


    - #### Cipher

      ```
      protected Cipher(ProxyReEncryption proxy,
                       Cipher.LEVEL level,
                       Cipher cipher,
                       it.unisa.dia.gas.jpbc.Element reencryptionPairing)
      ```

      Re-encryption constructor. This sets `reencryptionPairing`, which
      is used during the decryption process by the recipient. As the encrypted
      contents are the same, this method simply references the elements in the
      `Cipher` (`cipher`) being re-encrypted.

      Parameters:
      :   `proxy` - `ProxyReEncryption`
      :   `level` - `Cipher.LEVEL` level
      :   `cipher` - `Cipher` to reference
      :   `reencryptionPairing` - re-encryption pairing


    - #### Cipher

      ```
      protected Cipher(ProxyReEncryption proxy,
                       Cipher.LEVEL level,
                       it.unisa.dia.gas.jpbc.Element signedHash,
                       it.unisa.dia.gas.jpbc.Element decryptionParameter)
      ```

      Re-encryption constructor for sign/encrypt-sign constructor. This sets
      signed/encrypt-signed hash and signed/encrypt-signed pairing, which is
      used during the verification process by the recipient.

      Parameters:
      :   `proxy` - `ProxyReEncryption`
      :   `level` - `Cipher.LEVEL` level
      :   `signedHash` - hash
      :   `decryptionParameter` - decryption parameter


    - #### Cipher

      ```
      protected Cipher(ProxyReEncryption proxy,
                       Cipher.LEVEL level,
                       Cipher cipher,
                       it.unisa.dia.gas.jpbc.Element reencryptionScalar,
                       it.unisa.dia.gas.jpbc.Element reencryptionPairing)
      ```

      Re-encryption constructor for two-party decryption This sets the
      intermediate's scalar and delegatee's re-encryption, which is used during
      the decryption process by the recipient. As the encrypted contents are
      the same, this method simply references the elements in the
      `Cipher` (`cipher`) being re-encrypted.

      Parameters:
      :   `proxy` - `ProxyReEncryption`
      :   `level` - `Cipher.LEVEL` level
      :   `cipher` - `Cipher` to reference
      :   `reencryptionScalar` - re-encryption intermediate scalar
      :   `reencryptionPairing` - re-encryption delegatee pairing
  - ### Method Detail


    - #### add

      ```
      protected void add(it.unisa.dia.gas.jpbc.Element element,
                         int length)
      ```

      Add an `Element`.

      Parameters:
      :   `element` - `Element` to add
      :   `length` - number of characters


    - #### getElements

      ```
      public java.util.List<it.unisa.dia.gas.jpbc.Element> getElements()
      ```

      Returns the list of elements.

      Returns:
      :   the list of elements


    - #### getLengths

      ```
      public java.util.List<java.lang.Integer> getLengths()
      ```

      Returns the list of lengths.

      Returns:
      :   the list of lengths


    - #### canReEncrypt

      ```
      public boolean canReEncrypt()
      ```

      Returns `true` if can re-encrypt, `false` otherwise.

      Returns:
      :   `true` if can re-encrypt, `false` otherwise


    - #### getDecryptionParameter

      ```
      public it.unisa.dia.gas.jpbc.Element getDecryptionParameter()
      ```

      Returns the decryption parameter element (for decrypt-verify).

      Returns:
      :   the decryption parameter element (for decrypt-verify)


    - #### getLevel

      ```
      public Cipher.LEVEL getLevel()
      ```

      Returns the `Cipher.LEVEL`.

      Returns:
      :   the `Cipher.LEVEL`


    - #### getReencryptionPairing

      ```
      public it.unisa.dia.gas.jpbc.Element getReencryptionPairing()
      ```

      Returns the re-encryption pairing element.

      Returns:
      :   the re-encryption pairing element


    - #### getReencryptionScalar

      ```
      public it.unisa.dia.gas.jpbc.Element getReencryptionScalar()
      ```

      Returns the intermediate re-encryption scalar element.

      Returns:
      :   the intermediate re-encryption scalar element


    - #### getSignedHash

      ```
      public it.unisa.dia.gas.jpbc.Element getSignedHash()
      ```

      Returns the hash for signed/encrypt-signed messages.

      Returns:
      :   the hash for signed/encrypt-signed messages


    - #### setDecryptionParameter

      ```
      public void setDecryptionParameter(it.unisa.dia.gas.jpbc.Element element)
      ```

      Sets the X/Y values for the decryption parameter pairing element.

      Parameters:
      :   `element` - `Element` to set


    - #### setReencryptionPairing

      ```
      public void setReencryptionPairing(byte[] bytes)
      ```

      Sets the X/Y values for the re-encryption pairing element.

      Parameters:
      :   `bytes` - bytes to set


    - #### setReencryptionPairing

      ```
      public void setReencryptionPairing(byte[] bytes,
                                         Cipher.PAIRING_TYPE type)
      ```

      Sets the X/Y values for the re-encryption pairing element.

      Parameters:
      :   `bytes` - bytes to set
      :   `type` - `Cipher.PAIRING_TYPE.FORMATTED` or
          `Cipher.PAIRING_TYPE.UNFORMATTED` bytes


    - #### setReencryptionPairing

      ```
      public void setReencryptionPairing(it.unisa.dia.gas.jpbc.Element element)
      ```

      Sets the X/Y values for the re-encryption pairing element.

      Parameters:
      :   `element` - `Element` to set


    - #### setReencryptionScalar

      ```
      public void setReencryptionScalar(byte[] bytes)
      ```

      Sets the value of the intermediate re-encryption scalar element.

      Parameters:
      :   `bytes` - bytes to set


    - #### setReencryptionScalar

      ```
      public void setReencryptionScalar(byte[] bytes,
                                        Cipher.PAIRING_TYPE type)
      ```

      Sets the value of the intermediate re-encryption scalar element.

      Parameters:
      :   `bytes` - bytes to set
      :   `type` - `Cipher.PAIRING_TYPE.FORMATTED` or
          `Cipher.PAIRING_TYPE.UNFORMATTED` bytes


    - #### setReencryptionScalar

      ```
      public void setReencryptionScalar(it.unisa.dia.gas.jpbc.Element element)
      ```

      Sets the value of the intermediate re-encryption scalar element.

      Parameters:
      :   `element` - `Element` to set


    - #### setSignedHash

      ```
      public void setSignedHash(it.unisa.dia.gas.jpbc.Element element)
      ```

      Sets the hash value of a signed/encrypt-signed message.

      Parameters:
      :   `element` - `Element` to set


    - #### setToTwoParty

      ```
      public void setToTwoParty()
      ```

      Sets the `Cipher.LEVEL` to `Cipher.LEVEL.TWOPARTY`. Used when manually
      converting a regular `Cipher` to a two-party `Cipher`.


    - #### toString

      ```
      public java.lang.String toString()
      ```

      Overrides:
      :   `toString` in class `java.lang.Object`


    - #### toBytes

      ```
      public byte[] toBytes()
      ```

      Converts a `Cipher` to a `byte[]`.

      Returns:
      :   `Cipher` as a `byte[]`


    - #### toHashBytes

      ```
      public byte[] toHashBytes()
      ```

      Converts a `Cipher` to a `byte[]` suitable for hashing. This
      CANNOT be reversed.

      Returns:
      :   `Cipher` as a `byte[]`


    - #### write

      ```
      public void write(java.lang.String file)
      ```

      Writes `this` to a file.

      Parameters:
      :   `file` - file name


Skip navigation links


- Package
- Class
- Use
- Tree
- Deprecated
- Index
- Help

- Prev Class
- Next Class

- Frames
- No Frames

- All Classes

- Summary:
- Nested |
- Field |
- Constr |
- Method

- Detail:
- Field |
- Constr |
- Method
