# Supplementary material for: A Blockchain Framework for Patient-Centered Health Records and Exchange (HealthChain): Evaluation and Proof-of-Concept Study
Source: J Med Internet Res. 2019 Aug 31;21(8):e13592. doi: 10.2196/13592 (PMC6743266; doi:10.2196/13592)
Supplement: Multimedia Appendix 3 [file jmir_v21i8e13592_app3.zip › ProxyReEncryption/javadoc/edu/ecu/hsim/ray/proxyreencryption/Cipher.LEVEL.html]

Cipher.LEVEL


JavaScript is disabled on your browser.


Skip navigation links


- Package
- Class
- Use
- Tree
- Deprecated
- Index
- Help

- Prev Class
- Next Class

- Frames
- No Frames

- All Classes

- Summary:
- Nested |
- Enum Constants |
- Field |
- Method

- Detail:
- Enum Constants |
- Field |
- Method


edu.ecu.hsim.ray.proxyreencryption

## Enum Cipher.LEVEL

- java.lang.Object
- - java.lang.Enum<Cipher.LEVEL>
  - - edu.ecu.hsim.ray.proxyreencryption.Cipher.LEVEL

- All Implemented Interfaces:
  :   java.io.Serializable, java.lang.Comparable<Cipher.LEVEL>

  Enclosing class:
  :   Cipher

  ---

    

  ```
  protected static enum Cipher.LEVEL
  extends java.lang.Enum<Cipher.LEVEL>
  ```

  Flags each `Cipher` with the appropriate level. Note: encryption,
  decryption, and re-encryption are mathematically defined and not subject
  to manual annotation (i.e., a miss-flag will not allow, e.g., a
  first-level encrypted message to be re-encrypted). This flag simplifies
  `Cipher` processing, as it allows the use of `switch`
  statements instead of `try/catch` blocks or `Element`
  mappings. First/second-level encryptions are decrypted using their
  respective decryption methods. Re-encrypted messages require the original
  message to be second-level encrypted; decryption using the
  \(rk\_{A\rightarrow{B}}\) re-encryption key is first-level. Two-party
  re-encryption requires the original message to be second-level encrypted.
  Intermediate re-encryption scales this while decryption using
  \(rk\_{AR\rightarrow{B}}\) is first-level.

- - ### Enum Constant Summary

    Enum Constants

    Enum Constant and Description

    `ENCRYPTSIGN`

    Encrypt-sign cipher.

    `FIRST`

    First-level cipher.

    `REENCRYPTED`

    Re-encrypted cipher.

    `SECOND`

    Second-level cipher.

    `SIGN`

    Sign cipher.

    `TWOPARTY`

    Two-party cipher.
  - ### Method Summary

    All Methods Static Methods Concrete Methods

    | Modifier and Type | Method and Description |
    | --- | --- |
    | `static Cipher.LEVEL` | `valueOf(java.lang.String name)` Returns the enum constant of this type with the specified name. |
    | `static Cipher.LEVEL[]` | `values()` Returns an array containing the constants of this enum type, in the order they are declared. |

    - ### Methods inherited from class java.lang.Enum

      `clone, compareTo, equals, finalize, getDeclaringClass, hashCode, name, ordinal, toString, valueOf`
    - ### Methods inherited from class java.lang.Object

      `getClass, notify, notifyAll, wait, wait, wait`

- - ### Enum Constant Detail


    - #### ENCRYPTSIGN

      ```
      public static final Cipher.LEVEL ENCRYPTSIGN
      ```

      Encrypt-sign cipher.


    - #### FIRST

      ```
      public static final Cipher.LEVEL FIRST
      ```

      First-level cipher.


    - #### REENCRYPTED

      ```
      public static final Cipher.LEVEL REENCRYPTED
      ```

      Re-encrypted cipher.


    - #### SECOND

      ```
      public static final Cipher.LEVEL SECOND
      ```

      Second-level cipher.


    - #### SIGN

      ```
      public static final Cipher.LEVEL SIGN
      ```

      Sign cipher.


    - #### TWOPARTY

      ```
      public static final Cipher.LEVEL TWOPARTY
      ```

      Two-party cipher.
  - ### Method Detail


    - #### values

      ```
      public static Cipher.LEVEL[] values()
      ```

      Returns an array containing the constants of this enum type, in
      the order they are declared. This method may be used to iterate
      over the constants as follows:

      ```
      for (Cipher.LEVEL c : Cipher.LEVEL.values())
          System.out.println(c);
      ```

      Returns:
      :   an array containing the constants of this enum type, in the order they are declared


    - #### valueOf

      ```
      public static Cipher.LEVEL valueOf(java.lang.String name)
      ```

      Returns the enum constant of this type with the specified name.
      The string must match *exactly* an identifier used to declare an
      enum constant in this type. (Extraneous whitespace characters are
      not permitted.)

      Parameters:
      :   `name` - the name of the enum constant to be returned.

      Returns:
      :   the enum constant with the specified name

      Throws:
      :   `java.lang.IllegalArgumentException` - if this enum type has no constant with the specified name
      :   `java.lang.NullPointerException` - if the argument is null


Skip navigation links


- Package
- Class
- Use
- Tree
- Deprecated
- Index
- Help

- Prev Class
- Next Class

- Frames
- No Frames

- All Classes

- Summary:
- Nested |
- Enum Constants |
- Field |
- Method

- Detail:
- Enum Constants |
- Field |
- Method
