# Supplementary material for: A Blockchain Framework for Patient-Centered Health Records and Exchange (HealthChain): Evaluation and Proof-of-Concept Study
Source: J Med Internet Res. 2019 Aug 31;21(8):e13592. doi: 10.2196/13592 (PMC6743266; doi:10.2196/13592)
Supplement: Multimedia Appendix 3 [file jmir_v21i8e13592_app3.zip › ProxyReEncryption/javadoc/edu/ecu/hsim/ray/proxyreencryption/Cipher.PAIRING_TYPE.html]

Cipher.PAIRING\_TYPE


JavaScript is disabled on your browser.


Skip navigation links


- Package
- Class
- Use
- Tree
- Deprecated
- Index
- Help

- Prev Class
- Next Class

- Frames
- No Frames

- All Classes

- Summary:
- Nested |
- Enum Constants |
- Field |
- Method

- Detail:
- Enum Constants |
- Field |
- Method


edu.ecu.hsim.ray.proxyreencryption

## Enum Cipher.PAIRING\_TYPE

- java.lang.Object
- - java.lang.Enum<Cipher.PAIRING\_TYPE>
  - - edu.ecu.hsim.ray.proxyreencryption.Cipher.PAIRING\_TYPE

- All Implemented Interfaces:
  :   java.io.Serializable, java.lang.Comparable<Cipher.PAIRING\_TYPE>

  Enclosing class:
  :   Cipher

  ---

    

  ```
  public static enum Cipher.PAIRING_TYPE
  extends java.lang.Enum<Cipher.PAIRING_TYPE>
  ```

  When loading a pairing `Element`, one can specify if it is a
  formatted or unformatted byte array.

- - ### Enum Constant Summary

    Enum Constants

    Enum Constant and Description

    `FORMATTED`

    Load formatted bytes

    `UNFORMATTED`

    Load unformatted bytes
  - ### Method Summary

    All Methods Static Methods Concrete Methods

    | Modifier and Type | Method and Description |
    | --- | --- |
    | `static Cipher.PAIRING_TYPE` | `valueOf(java.lang.String name)` Returns the enum constant of this type with the specified name. |
    | `static Cipher.PAIRING_TYPE[]` | `values()` Returns an array containing the constants of this enum type, in the order they are declared. |

    - ### Methods inherited from class java.lang.Enum

      `clone, compareTo, equals, finalize, getDeclaringClass, hashCode, name, ordinal, toString, valueOf`
    - ### Methods inherited from class java.lang.Object

      `getClass, notify, notifyAll, wait, wait, wait`

- - ### Enum Constant Detail


    - #### FORMATTED

      ```
      public static final Cipher.PAIRING_TYPE FORMATTED
      ```

      Load formatted bytes


    - #### UNFORMATTED

      ```
      public static final Cipher.PAIRING_TYPE UNFORMATTED
      ```

      Load unformatted bytes
  - ### Method Detail


    - #### values

      ```
      public static Cipher.PAIRING_TYPE[] values()
      ```

      Returns an array containing the constants of this enum type, in
      the order they are declared. This method may be used to iterate
      over the constants as follows:

      ```
      for (Cipher.PAIRING_TYPE c : Cipher.PAIRING_TYPE.values())
          System.out.println(c);
      ```

      Returns:
      :   an array containing the constants of this enum type, in the order they are declared


    - #### valueOf

      ```
      public static Cipher.PAIRING_TYPE valueOf(java.lang.String name)
      ```

      Returns the enum constant of this type with the specified name.
      The string must match *exactly* an identifier used to declare an
      enum constant in this type. (Extraneous whitespace characters are
      not permitted.)

      Parameters:
      :   `name` - the name of the enum constant to be returned.

      Returns:
      :   the enum constant with the specified name

      Throws:
      :   `java.lang.IllegalArgumentException` - if this enum type has no constant with the specified name
      :   `java.lang.NullPointerException` - if the argument is null


Skip navigation links


- Package
- Class
- Use
- Tree
- Deprecated
- Index
- Help

- Prev Class
- Next Class

- Frames
- No Frames

- All Classes

- Summary:
- Nested |
- Enum Constants |
- Field |
- Method

- Detail:
- Enum Constants |
- Field |
- Method
