# Supplementary material for: A Blockchain Framework for Patient-Centered Health Records and Exchange (HealthChain): Evaluation and Proof-of-Concept Study
Source: J Med Internet Res. 2019 Aug 31;21(8):e13592. doi: 10.2196/13592 (PMC6743266; doi:10.2196/13592)
Supplement: Multimedia Appendix 3 [file jmir_v21i8e13592_app3.zip › ProxyReEncryption/javadoc/edu/ecu/hsim/ray/proxyreencryption/class-use/AFGH.LENGTH.html]

Uses of Class edu.ecu.hsim.ray.proxyreencryption.AFGH.LENGTH


JavaScript is disabled on your browser.


Skip navigation links


- Package
- Class
- Use
- Tree
- Deprecated
- Index
- Help

- Prev
- Next

- Frames
- No Frames

- All Classes

## Uses of Class edu.ecu.hsim.ray.proxyreencryption.AFGH.LENGTH

- - ### Uses of AFGH.LENGTH in edu.ecu.hsim.ray.proxyreencryption

    Methods in edu.ecu.hsim.ray.proxyreencryption that return AFGH.LENGTH

    | Modifier and Type | Method and Description |
    | --- | --- |
    |  |  |
    | --- | --- |
    | `static AFGH.LENGTH` | AFGH.LENGTH.`valueOf(java.lang.String name)` Returns the enum constant of this type with the specified name. |
    | `static AFGH.LENGTH[]` | AFGH.LENGTH.`values()` Returns an array containing the constants of this enum type, in the order they are declared. |

    Constructors in edu.ecu.hsim.ray.proxyreencryption with parameters of type AFGH.LENGTH

    Constructor and Description

    `AFGH(AFGH.LENGTH length)`

    Constructs a new `STORAGE#VOLATILE` `AFGH` with elliptic
    curve key length of size `length` and encryption mode of
    `ProxyReEncryption.DEFAULT_ENCRYPTION_MODE`.

    `AFGH(AFGH.LENGTH length,
    byte[] curveParams)`

    Constructs a new `STORAGE#NONVOLATILE` `AFGH`.

    `AFGH(AFGH.LENGTH length,
    byte[] curveParams,
    ProxyReEncryption.ENCRYPTION_MODE encMode)`

    Constructs a new `STORAGE#NONVOLATILE` `AFGH`.

    `AFGH(AFGH.LENGTH length,
    ProxyReEncryption.ENCRYPTION_MODE encMode)`

    Constructs a new `STORAGE#VOLATILE` `AFGH` with elliptic
    curve key length of size `length` and encryption mode of
    `encMode`.

    `AFGH(AFGH.LENGTH length,
    java.lang.String curveParams)`

    Constructs a new `STORAGE#NONVOLATILE` `AFGH`.

    `AFGH(AFGH.LENGTH length,
    java.lang.String curveParams,
    ProxyReEncryption.ENCRYPTION_MODE encMode)`

    Constructs a new `STORAGE#NONVOLATILE` `AFGH`.

Skip navigation links


- Package
- Class
- Use
- Tree
- Deprecated
- Index
- Help

- Prev
- Next

- Frames
- No Frames

- All Classes
