# Supplementary material for: A Blockchain Framework for Patient-Centered Health Records and Exchange (HealthChain): Evaluation and Proof-of-Concept Study
Source: J Med Internet Res. 2019 Aug 31;21(8):e13592. doi: 10.2196/13592 (PMC6743266; doi:10.2196/13592)
Supplement: Multimedia Appendix 3 [file jmir_v21i8e13592_app3.zip › ProxyReEncryption/javadoc/edu/ecu/hsim/ray/proxyreencryption/class-use/Cipher.html]

Uses of Class edu.ecu.hsim.ray.proxyreencryption.Cipher


JavaScript is disabled on your browser.


Skip navigation links


- Package
- Class
- Use
- Tree
- Deprecated
- Index
- Help

- Prev
- Next

- Frames
- No Frames

- All Classes

## Uses of Class edu.ecu.hsim.ray.proxyreencryption.Cipher

- - ### Uses of Cipher in edu.ecu.hsim.ray.proxyreencryption

    Methods in edu.ecu.hsim.ray.proxyreencryption that return Cipher

    | Modifier and Type | Method and Description |
    | --- | --- |
    |  |  |
    | --- | --- |
    | `Cipher` | AFGH.`decryptIntermediate(Keys key, Cipher cipher)` |
    | `abstract Cipher` | ProxyReEncryption.`decryptIntermediate(Keys key, Cipher cipher)` Decrypts the intermediate layer of a two-party re-encryption `Cipher` using the provided private key. |
    | `Cipher` | AFGH.`decryptToCipher(it.unisa.dia.gas.jpbc.Element x, Cipher cipher)` |
    | `abstract Cipher` | ProxyReEncryption.`decryptToCipher(it.unisa.dia.gas.jpbc.Element x, Cipher cipher)` Decrypts the `Cipher` to a `Cipher` using the provided ephemeral key `x`. |
    | `Cipher` | AFGH.`decryptToCipher(Keys key, Cipher cipher)` |
    | `abstract Cipher` | ProxyReEncryption.`decryptToCipher(Keys keys, Cipher cipher)` Decrypts the `Cipher` to a `Cipher` using the provided private key. |
    | `Cipher` | AFGH.`encrypt(it.unisa.dia.gas.jpbc.Element x, byte[] message, ProxyReEncryption.MESSAGE_TYPE type)` |
    | `abstract Cipher` | ProxyReEncryption.`encrypt(it.unisa.dia.gas.jpbc.Element x, byte[] message, ProxyReEncryption.MESSAGE_TYPE type)` Encrypts the `message` using the ephemeral key `x`. |
    | `Cipher` | AFGH.`encrypt(it.unisa.dia.gas.jpbc.Element x, Cipher cipher)` |
    | `abstract Cipher` | ProxyReEncryption.`encrypt(it.unisa.dia.gas.jpbc.Element x, Cipher cipher)` Encrypts the `Cipher` using the ephemeral key `x`. |
    | `Cipher` | AFGH.`encrypt(it.unisa.dia.gas.jpbc.Element x, Message message)` |
    | `abstract Cipher` | ProxyReEncryption.`encrypt(it.unisa.dia.gas.jpbc.Element x, Message message)` Encrypts the `Message` using the ephemeral key `x`. |
    | `Cipher` | AFGH.`encrypt(it.unisa.dia.gas.jpbc.Element x, java.lang.String message)` |
    | `abstract Cipher` | ProxyReEncryption.`encrypt(it.unisa.dia.gas.jpbc.Element x, java.lang.String message)` Encrypts the `message` using the ephemeral key `x`. |
    | `Cipher` | AFGH.`encrypt(Keys keys, byte[] message, ProxyReEncryption.MESSAGE_TYPE type)` |
    | `abstract Cipher` | ProxyReEncryption.`encrypt(Keys keys, byte[] message, ProxyReEncryption.MESSAGE_TYPE type)` Encrypts the `message` using the public key. |
    | `Cipher` | AFGH.`encrypt(Keys keys, byte[] message, ProxyReEncryption.MESSAGE_TYPE type, ProxyReEncryption.REENCRYPT reencrypt)` |
    | `abstract Cipher` | ProxyReEncryption.`encrypt(Keys keys, byte[] message, ProxyReEncryption.MESSAGE_TYPE type, ProxyReEncryption.REENCRYPT reencrypt)` Encrypts the `message` using the public key and `reencrypt` parameter. |
    | `Cipher` | AFGH.`encrypt(Keys keys, Cipher cipher)` |
    | `abstract Cipher` | ProxyReEncryption.`encrypt(Keys keys, Cipher cipher)` Encrypts the `Cipher` using the public key. |
    | `Cipher` | AFGH.`encrypt(Keys keys, Cipher cipher, ProxyReEncryption.REENCRYPT reencrypt)` |
    | `abstract Cipher` | ProxyReEncryption.`encrypt(Keys keys, Cipher cipher, ProxyReEncryption.REENCRYPT reencrypt)` Encrypts the `Cipher` using the public key and `reencrypt` parameter. |
    | `Cipher` | AFGH.`encrypt(Keys keys, Message message)` |
    | `abstract Cipher` | ProxyReEncryption.`encrypt(Keys keys, Message message)` Encrypts the `Message` using the public key. |
    | `Cipher` | AFGH.`encrypt(Keys keys, Message message, ProxyReEncryption.REENCRYPT reencrypt)` |
    | `abstract Cipher` | ProxyReEncryption.`encrypt(Keys keys, Message message, ProxyReEncryption.REENCRYPT reencrypt)` Encrypts the `Message` using the public key and `reencrypt` parameter. |
    | `Cipher` | AFGH.`encrypt(Keys keys, java.lang.String message)` |
    | `abstract Cipher` | ProxyReEncryption.`encrypt(Keys keys, java.lang.String message)` Encrypts the `message` using the public key. |
    | `Cipher` | AFGH.`encrypt(Keys keys, java.lang.String message, ProxyReEncryption.REENCRYPT reencrypt)` |
    | `abstract Cipher` | ProxyReEncryption.`encrypt(Keys keys, java.lang.String message, ProxyReEncryption.REENCRYPT reencrypt)` Encrypts the `message` using the public key and `reencrypt` parameter. |
    | `Cipher` | ProxyReEncryption.`readCipher(java.lang.String file)` Reads a `Cipher` from a file. |
    | `Cipher` | AFGH.`reencrypt(Keys rekey, Cipher cipher)` Re-encrypts a `Cipher` using the re-encryption `Keys`. |
    | `abstract Cipher` | ProxyReEncryption.`reencrypt(Keys rekey, Cipher cipher)` Re-encrypts a `Cipher` using the re-encryption `Keys`. |
    | `Cipher` | AFGH.`reencrypt(Keys key, Keys rekey, Cipher cipher)` Re-encrypts a two-party `Cipher` using the re-encryption `Keys`. |
    | `abstract Cipher` | ProxyReEncryption.`reencrypt(Keys key, Keys rekey, Cipher cipher)` Re-encrypts a two-party `Cipher` using the re-encryption `Keys`. |
    | `Cipher` | AFGH.`sign(Keys keys, byte[] message, ProxyReEncryption.MESSAGE_TYPE type)` |
    | `abstract Cipher` | ProxyReEncryption.`sign(Keys keys, byte[] message, ProxyReEncryption.MESSAGE_TYPE type)` Signs the `message` using an ephemeral key `x`, returning a `Cipher` that is verified by its hash and a private key-based decryption parameter (all contained within the `Cipher`). |
    | `Cipher` | AFGH.`sign(Keys keys, Cipher cipher)` |
    | `abstract Cipher` | ProxyReEncryption.`sign(Keys keys, Cipher cipher)` Signs the `Cipher` using an ephemeral key `x`, returning a `Cipher` that is verified by its hash and a private key-based decryption parameter (all contained within the `Cipher`). |
    | `Cipher` | AFGH.`sign(Keys sign, Keys encrypt, byte[] message, ProxyReEncryption.MESSAGE_TYPE type)` |
    | `abstract Cipher` | ProxyReEncryption.`sign(Keys sign, Keys encrypt, byte[] message, ProxyReEncryption.MESSAGE_TYPE type)` Encrypt-signs the `message` using an ephemeral key `x`, returning a `Cipher` that is decrypt-verified by its hash and a decryption parameter constructed using the signer's private key and recipients public key (all contained within the `Cipher`). |
    | `Cipher` | AFGH.`sign(Keys sign, Keys encrypt, Cipher cipher)` |
    | `abstract Cipher` | ProxyReEncryption.`sign(Keys sign, Keys encrypt, Cipher cipher)` Encrypt-signs the `Cipher` using an ephemeral key `x`, returning a `Cipher` that is decrypt-verified by its hash and a decryption parameter constructed using the signer's private key and recipients public key (all contained within the `Cipher`). |
    | `Cipher` | AFGH.`sign(Keys sign, Keys encrypt, Message message)` |
    | `abstract Cipher` | ProxyReEncryption.`sign(Keys sign, Keys encrypt, Message message)` Encrypt-signs the `Message` using an ephemeral key `x`, returning a `Cipher` that is decrypt-verified by its hash and a decryption parameter constructed using the signer's private key and recipients public key (all contained within the `Cipher`). |
    | `Cipher` | AFGH.`sign(Keys sign, Keys encrypt, java.lang.String message)` |
    | `abstract Cipher` | ProxyReEncryption.`sign(Keys sign, Keys encrypt, java.lang.String message)` Encrypt-signs the `message` using an ephemeral key `x`, returning a `Cipher` that is decrypt-verified by its hash and a decryption parameter constructed using the signer's private key and recipients public key (all contained within the `Cipher`). |
    | `Cipher` | AFGH.`sign(Keys keys, Message message)` |
    | `abstract Cipher` | ProxyReEncryption.`sign(Keys keys, Message message)` Signs the `Message` using an ephemeral key `x`, returning a `Cipher` that is verified by its hash and a private key-based decryption parameter (all contained within the `Cipher`). |
    | `Cipher` | AFGH.`sign(Keys keys, java.lang.String message)` |
    | `abstract Cipher` | ProxyReEncryption.`sign(Keys keys, java.lang.String message)` Signs the `message` using an ephemeral key `x`, returning a `Cipher` that is verified by its hash and a private key-based decryption parameter (all contained within the `Cipher`). |
    | `Cipher` | AFGH.`toCipher(byte[] bytes)` |
    | `abstract Cipher` | ProxyReEncryption.`toCipher(byte[] bytes)` Converts a `byte[]` to a `Cipher`. |

    Methods in edu.ecu.hsim.ray.proxyreencryption with parameters of type Cipher

    | Modifier and Type | Method and Description |
    | --- | --- |
    |  |  |
    | --- | --- |
    | `Message` | AFGH.`decrypt(it.unisa.dia.gas.jpbc.Element x, Cipher cipher)` |
    | `abstract Message` | ProxyReEncryption.`decrypt(it.unisa.dia.gas.jpbc.Element x, Cipher cipher)` Decrypts the `Cipher` using the provided ephemeral key `x`. |
    | `Message` | AFGH.`decrypt(Keys key, Cipher cipher)` |
    | `abstract Message` | ProxyReEncryption.`decrypt(Keys keys, Cipher cipher)` Decrypts the `Cipher` using the provided private key. |
    | `Message` | AFGH.`decryptDelegatee(Keys key, Cipher cipher)` |
    | `abstract Message` | ProxyReEncryption.`decryptDelegatee(Keys key, Cipher cipher)` Decrypts the delegatee layer of a two-party re-encryption `Cipher` using the provided private key. |
    | `Cipher` | AFGH.`decryptIntermediate(Keys key, Cipher cipher)` |
    | `abstract Cipher` | ProxyReEncryption.`decryptIntermediate(Keys key, Cipher cipher)` Decrypts the intermediate layer of a two-party re-encryption `Cipher` using the provided private key. |
    | `Cipher` | AFGH.`decryptToCipher(it.unisa.dia.gas.jpbc.Element x, Cipher cipher)` |
    | `abstract Cipher` | ProxyReEncryption.`decryptToCipher(it.unisa.dia.gas.jpbc.Element x, Cipher cipher)` Decrypts the `Cipher` to a `Cipher` using the provided ephemeral key `x`. |
    | `Cipher` | AFGH.`decryptToCipher(Keys key, Cipher cipher)` |
    | `abstract Cipher` | ProxyReEncryption.`decryptToCipher(Keys keys, Cipher cipher)` Decrypts the `Cipher` to a `Cipher` using the provided private key. |
    | `Cipher` | AFGH.`encrypt(it.unisa.dia.gas.jpbc.Element x, Cipher cipher)` |
    | `abstract Cipher` | ProxyReEncryption.`encrypt(it.unisa.dia.gas.jpbc.Element x, Cipher cipher)` Encrypts the `Cipher` using the ephemeral key `x`. |
    | `Cipher` | AFGH.`encrypt(Keys keys, Cipher cipher)` |
    | `abstract Cipher` | ProxyReEncryption.`encrypt(Keys keys, Cipher cipher)` Encrypts the `Cipher` using the public key. |
    | `Cipher` | AFGH.`encrypt(Keys keys, Cipher cipher, ProxyReEncryption.REENCRYPT reencrypt)` |
    | `abstract Cipher` | ProxyReEncryption.`encrypt(Keys keys, Cipher cipher, ProxyReEncryption.REENCRYPT reencrypt)` Encrypts the `Cipher` using the public key and `reencrypt` parameter. |
    | `Cipher` | AFGH.`reencrypt(Keys rekey, Cipher cipher)` Re-encrypts a `Cipher` using the re-encryption `Keys`. |
    | `abstract Cipher` | ProxyReEncryption.`reencrypt(Keys rekey, Cipher cipher)` Re-encrypts a `Cipher` using the re-encryption `Keys`. |
    | `Cipher` | AFGH.`reencrypt(Keys key, Keys rekey, Cipher cipher)` Re-encrypts a two-party `Cipher` using the re-encryption `Keys`. |
    | `abstract Cipher` | ProxyReEncryption.`reencrypt(Keys key, Keys rekey, Cipher cipher)` Re-encrypts a two-party `Cipher` using the re-encryption `Keys`. |
    | `void` | AFGH.`scale(Cipher cipher, it.unisa.dia.gas.jpbc.Element from, it.unisa.dia.gas.jpbc.Element to, ProxyReEncryption.SCALE scaleType)` |
    | `abstract void` | ProxyReEncryption.`scale(Cipher cipher, it.unisa.dia.gas.jpbc.Element from, it.unisa.dia.gas.jpbc.Element to, ProxyReEncryption.SCALE scaleType)` Transforms the encryption key of the `Cipher` from `from` to `to` by way of simple scaling. |
    | `void` | AFGH.`scale(Cipher cipher, it.unisa.dia.gas.jpbc.Element scalar, ProxyReEncryption.SCALE scaleType)` |
    | `abstract void` | ProxyReEncryption.`scale(Cipher cipher, it.unisa.dia.gas.jpbc.Element scalar, ProxyReEncryption.SCALE scaleType)` Transforms the encryption key of the `Cipher` from `from` to `to` by way of simple scaling. |
    | `Cipher` | AFGH.`sign(Keys keys, Cipher cipher)` |
    | `abstract Cipher` | ProxyReEncryption.`sign(Keys keys, Cipher cipher)` Signs the `Cipher` using an ephemeral key `x`, returning a `Cipher` that is verified by its hash and a private key-based decryption parameter (all contained within the `Cipher`). |
    | `Cipher` | AFGH.`sign(Keys sign, Keys encrypt, Cipher cipher)` |
    | `abstract Cipher` | ProxyReEncryption.`sign(Keys sign, Keys encrypt, Cipher cipher)` Encrypt-signs the `Cipher` using an ephemeral key `x`, returning a `Cipher` that is decrypt-verified by its hash and a decryption parameter constructed using the signer's private key and recipients public key (all contained within the `Cipher`). |
    | `boolean` | AFGH.`verify(Keys keys, Cipher cipher)` |
    | `abstract boolean` | ProxyReEncryption.`verify(Keys keys, Cipher cipher)` Verifies a signed `Cipher` using the signer's private key, a message hash, and a decryption parameter generated using the signer's private key and the message hash. |
    | `boolean` | AFGH.`verify(Keys keys, Cipher cipher, byte[] expected)` |
    | `abstract boolean` | ProxyReEncryption.`verify(Keys keys, Cipher cipher, byte[] expected)` Verifies a signed `Cipher` using the signer's private key, a message hash, and a decryption parameter generated using the signer's private key and the message hash. |
    | `boolean` | AFGH.`verify(Keys keys, Cipher cipher, Message expected)` |
    | `abstract boolean` | ProxyReEncryption.`verify(Keys keys, Cipher cipher, Message expected)` Verifies a signed `Cipher` using the signer's private key, a message hash, and a decryption parameter generated using the signer's private key and the message hash. |
    | `boolean` | AFGH.`verify(Keys keys, Cipher cipher, java.lang.String expected)` |
    | `abstract boolean` | ProxyReEncryption.`verify(Keys keys, Cipher cipher, java.lang.String expected)` Verifies a signed `Cipher` using the signer's private key, a message hash, and a decryption parameter generated using the signer's private key and the message hash. |
    | `boolean` | AFGH.`verify(Keys verify, Keys decrypt, Cipher cipher)` |
    | `abstract boolean` | ProxyReEncryption.`verify(Keys verify, Keys decrypt, Cipher cipher)` Decrypt-verifies a signed `Cipher` using the signer's private key, a message hash, and a decryption parameter generated using the signer's private key and the message hash. |
    | `boolean` | AFGH.`verify(Keys verify, Keys decrypt, Cipher cipher, byte[] expected)` |
    | `abstract boolean` | ProxyReEncryption.`verify(Keys verify, Keys decrypt, Cipher cipher, byte[] expected)` Decrypt-verifies a signed `Cipher` using the signer's private key, a message hash, and a decryption parameter generated using the signer's private key and the message hash. |
    | `boolean` | AFGH.`verify(Keys verify, Keys decrypt, Cipher cipher, Message expected)` |
    | `abstract boolean` | ProxyReEncryption.`verify(Keys verify, Keys decrypt, Cipher cipher, Message expected)` Decrypt-verifies a signed `Cipher` using the signer's private key, a message hash, and a decryption parameter generated using the signer's private key and the message hash. |
    | `boolean` | AFGH.`verify(Keys verify, Keys decrypt, Cipher cipher, java.lang.String expected)` |
    | `abstract boolean` | ProxyReEncryption.`verify(Keys verify, Keys decrypt, Cipher cipher, java.lang.String expected)` Decrypt-verifies a signed `Cipher` using the signer's private key, a message hash, and a decryption parameter generated using the signer's private key and the message hash. |
    | `Message` | AFGH.`verifyAndGet(Keys keys, Cipher cipher)` |
    | `abstract Message` | ProxyReEncryption.`verifyAndGet(Keys keys, Cipher cipher)` Verifies a signed `Cipher` using the signer's private key, a message hash, and a decryption parameter generated using the signer's private key and the message hash. |
    | `Message` | AFGH.`verifyAndGet(Keys verify, Keys decrypt, Cipher cipher)` |
    | `abstract Message` | ProxyReEncryption.`verifyAndGet(Keys verify, Keys decrypt, Cipher cipher)` Decrypt-verifies a signed `Cipher` using the signer's private key, a message hash, and a decryption parameter generated using the signer's private key and the message hash. |

    Constructors in edu.ecu.hsim.ray.proxyreencryption with parameters of type Cipher

    Constructor and Description

    `Cipher(ProxyReEncryption proxy,
    Cipher.LEVEL level,
    Cipher cipher,
    it.unisa.dia.gas.jpbc.Element reencryptionPairing)`

    Re-encryption constructor.

    `Cipher(ProxyReEncryption proxy,
    Cipher.LEVEL level,
    Cipher cipher,
    it.unisa.dia.gas.jpbc.Element reencryptionScalar,
    it.unisa.dia.gas.jpbc.Element reencryptionPairing)`

    Re-encryption constructor for two-party decryption This sets the
    intermediate's scalar and delegatee's re-encryption, which is used during
    the decryption process by the recipient.

Skip navigation links


- Package
- Class
- Use
- Tree
- Deprecated
- Index
- Help

- Prev
- Next

- Frames
- No Frames

- All Classes
