# Supplementary material for: A Blockchain Framework for Patient-Centered Health Records and Exchange (HealthChain): Evaluation and Proof-of-Concept Study
Source: J Med Internet Res. 2019 Aug 31;21(8):e13592. doi: 10.2196/13592 (PMC6743266; doi:10.2196/13592)
Supplement: Multimedia Appendix 3 [file jmir_v21i8e13592_app3.zip › ProxyReEncryption/javadoc/edu/ecu/hsim/ray/proxyreencryption/class-use/Cipher.LEVEL.html]

Uses of Class edu.ecu.hsim.ray.proxyreencryption.Cipher.LEVEL


JavaScript is disabled on your browser.


Skip navigation links


- Package
- Class
- Use
- Tree
- Deprecated
- Index
- Help

- Prev
- Next

- Frames
- No Frames

- All Classes

## Uses of Class edu.ecu.hsim.ray.proxyreencryption.Cipher.LEVEL

- - ### Uses of Cipher.LEVEL in edu.ecu.hsim.ray.proxyreencryption

    Fields in edu.ecu.hsim.ray.proxyreencryption declared as Cipher.LEVEL

    | Modifier and Type | Field and Description |
    | --- | --- |
    |  |  |
    | --- | --- |
    | `protected Cipher.LEVEL` | Cipher.`level` `Cipher.LEVEL` of cipher. |

    Methods in edu.ecu.hsim.ray.proxyreencryption that return Cipher.LEVEL

    | Modifier and Type | Method and Description |
    | --- | --- |
    |  |  |
    | --- | --- |
    | `Cipher.LEVEL` | Cipher.`getLevel()` Returns the `Cipher.LEVEL`. |
    | `Cipher.LEVEL` | ProxyReEncryption.REENCRYPT.`getLevel()` Returns the `Cipher.LEVEL`. |
    | `static Cipher.LEVEL` | Cipher.LEVEL.`valueOf(java.lang.String name)` Returns the enum constant of this type with the specified name. |
    | `static Cipher.LEVEL[]` | Cipher.LEVEL.`values()` Returns an array containing the constants of this enum type, in the order they are declared. |

    Constructors in edu.ecu.hsim.ray.proxyreencryption with parameters of type Cipher.LEVEL

    Constructor and Description

    `Cipher(ProxyReEncryption proxy,
    Cipher.LEVEL level)`

    Constructor.

    `Cipher(ProxyReEncryption proxy,
    Cipher.LEVEL level,
    Cipher cipher,
    it.unisa.dia.gas.jpbc.Element reencryptionPairing)`

    Re-encryption constructor.

    `Cipher(ProxyReEncryption proxy,
    Cipher.LEVEL level,
    Cipher cipher,
    it.unisa.dia.gas.jpbc.Element reencryptionScalar,
    it.unisa.dia.gas.jpbc.Element reencryptionPairing)`

    Re-encryption constructor for two-party decryption This sets the
    intermediate's scalar and delegatee's re-encryption, which is used during
    the decryption process by the recipient.

    `Cipher(ProxyReEncryption proxy,
    Cipher.LEVEL level,
    it.unisa.dia.gas.jpbc.Element reencryptionPairing)`

    Re-encryption constructor.

    `Cipher(ProxyReEncryption proxy,
    Cipher.LEVEL level,
    it.unisa.dia.gas.jpbc.Element signedHash,
    it.unisa.dia.gas.jpbc.Element decryptionParameter)`

    Re-encryption constructor for sign/encrypt-sign constructor.

Skip navigation links


- Package
- Class
- Use
- Tree
- Deprecated
- Index
- Help

- Prev
- Next

- Frames
- No Frames

- All Classes
