# Supplementary material for: A Blockchain Framework for Patient-Centered Health Records and Exchange (HealthChain): Evaluation and Proof-of-Concept Study
Source: J Med Internet Res. 2019 Aug 31;21(8):e13592. doi: 10.2196/13592 (PMC6743266; doi:10.2196/13592)
Supplement: Multimedia Appendix 3 [file jmir_v21i8e13592_app3.zip › ProxyReEncryption/javadoc/edu/ecu/hsim/ray/proxyreencryption/class-use/Cipher.PAIRING_TYPE.html]

Uses of Class edu.ecu.hsim.ray.proxyreencryption.Cipher.PAIRING\_TYPE


JavaScript is disabled on your browser.


Skip navigation links


- Package
- Class
- Use
- Tree
- Deprecated
- Index
- Help

- Prev
- Next

- Frames
- No Frames

- All Classes

## Uses of Class edu.ecu.hsim.ray.proxyreencryption.Cipher.PAIRING\_TYPE

- - ### Uses of Cipher.PAIRING\_TYPE in edu.ecu.hsim.ray.proxyreencryption

    Methods in edu.ecu.hsim.ray.proxyreencryption that return Cipher.PAIRING\_TYPE

    | Modifier and Type | Method and Description |
    | --- | --- |
    |  |  |
    | --- | --- |
    | `static Cipher.PAIRING_TYPE` | Cipher.PAIRING\_TYPE.`valueOf(java.lang.String name)` Returns the enum constant of this type with the specified name. |
    | `static Cipher.PAIRING_TYPE[]` | Cipher.PAIRING\_TYPE.`values()` Returns an array containing the constants of this enum type, in the order they are declared. |

    Methods in edu.ecu.hsim.ray.proxyreencryption with parameters of type Cipher.PAIRING\_TYPE

    | Modifier and Type | Method and Description |
    | --- | --- |
    |  |  |
    | --- | --- |
    | `void` | Cipher.`setReencryptionPairing(byte[] bytes, Cipher.PAIRING_TYPE type)` Sets the X/Y values for the re-encryption pairing element. |
    | `void` | Cipher.`setReencryptionScalar(byte[] bytes, Cipher.PAIRING_TYPE type)` Sets the value of the intermediate re-encryption scalar element. |

Skip navigation links


- Package
- Class
- Use
- Tree
- Deprecated
- Index
- Help

- Prev
- Next

- Frames
- No Frames

- All Classes
