# Supplementary material for: A Blockchain Framework for Patient-Centered Health Records and Exchange (HealthChain): Evaluation and Proof-of-Concept Study
Source: J Med Internet Res. 2019 Aug 31;21(8):e13592. doi: 10.2196/13592 (PMC6743266; doi:10.2196/13592)
Supplement: Multimedia Appendix 3 [file jmir_v21i8e13592_app3.zip › ProxyReEncryption/javadoc/edu/ecu/hsim/ray/proxyreencryption/class-use/Message.html]

Uses of Class edu.ecu.hsim.ray.proxyreencryption.Message


JavaScript is disabled on your browser.


Skip navigation links


- Package
- Class
- Use
- Tree
- Deprecated
- Index
- Help

- Prev
- Next

- Frames
- No Frames

- All Classes

## Uses of Class edu.ecu.hsim.ray.proxyreencryption.Message

- - ### Uses of Message in edu.ecu.hsim.ray.proxyreencryption

    Methods in edu.ecu.hsim.ray.proxyreencryption that return Message

    | Modifier and Type | Method and Description |
    | --- | --- |
    |  |  |
    | --- | --- |
    | `Message` | AFGH.`decrypt(it.unisa.dia.gas.jpbc.Element x, Cipher cipher)` |
    | `abstract Message` | ProxyReEncryption.`decrypt(it.unisa.dia.gas.jpbc.Element x, Cipher cipher)` Decrypts the `Cipher` using the provided ephemeral key `x`. |
    | `Message` | AFGH.`decrypt(it.unisa.dia.gas.jpbc.Element x, Message message)` |
    | `abstract Message` | ProxyReEncryption.`decrypt(it.unisa.dia.gas.jpbc.Element x, Message message)` Decrypts the `Message` using the provided ephemeral key `x`. |
    | `Message` | AFGH.`decrypt(Keys key, Cipher cipher)` |
    | `abstract Message` | ProxyReEncryption.`decrypt(Keys keys, Cipher cipher)` Decrypts the `Cipher` using the provided private key. |
    | `Message` | AFGH.`decrypt(Keys key, Message message)` |
    | `abstract Message` | ProxyReEncryption.`decrypt(Keys keys, Message message)` Decrypts the `Message` using the provided private key. |
    | `Message` | AFGH.`decryptDelegatee(Keys key, Cipher cipher)` |
    | `abstract Message` | ProxyReEncryption.`decryptDelegatee(Keys key, Cipher cipher)` Decrypts the delegatee layer of a two-party re-encryption `Cipher` using the provided private key. |
    | `Message` | AFGH.`decryptVerifyImpl(Keys verify, Keys decrypt, Message cipher)` Decrypt-verifies the `cipher` using the private key of the recipient (`decrypt`) and public key of the signer (`verify`), returning the decrypt-verified cipher as a `Message`. |
    | `Message` | ProxyReEncryption.`readMessage(java.lang.String file)` Reads a `Message` from a file. |
    | `Message` | AFGH.`toMessage(byte[] message, int bytePadding)` |
    | `abstract Message` | ProxyReEncryption.`toMessage(byte[] message, int bytePadding)` Converts a `byte[]` to a `Message` with padding. |
    | `Message` | AFGH.`toMessage(byte[] message, ProxyReEncryption.MESSAGE_TYPE type)` |
    | `abstract Message` | ProxyReEncryption.`toMessage(byte[] message, ProxyReEncryption.MESSAGE_TYPE type)` Converts a `byte[]` to a `Message`. |
    | `Message` | AFGH.`toMessage(java.lang.String message)` |
    | `abstract Message` | ProxyReEncryption.`toMessage(java.lang.String message)` Converts a `String` to a `Message`. |
    | `Message` | AFGH.`verifyAndGet(Keys keys, Cipher cipher)` |
    | `abstract Message` | ProxyReEncryption.`verifyAndGet(Keys keys, Cipher cipher)` Verifies a signed `Cipher` using the signer's private key, a message hash, and a decryption parameter generated using the signer's private key and the message hash. |
    | `Message` | AFGH.`verifyAndGet(Keys verify, Keys decrypt, Cipher cipher)` |
    | `abstract Message` | ProxyReEncryption.`verifyAndGet(Keys verify, Keys decrypt, Cipher cipher)` Decrypt-verifies a signed `Cipher` using the signer's private key, a message hash, and a decryption parameter generated using the signer's private key and the message hash. |
    | `Message` | AFGH.`verifyAndGet(Keys verify, Keys decrypt, Message message)` |
    | `abstract Message` | ProxyReEncryption.`verifyAndGet(Keys verify, Keys decrypt, Message message)` Decrypt-verifies a signed `Message` using the signer's private key, a message hash, and a decryption parameter generated using the signer's private key and the message hash. |
    | `Message` | AFGH.`verifyAndGet(Keys keys, Message message)` |
    | `abstract Message` | ProxyReEncryption.`verifyAndGet(Keys keys, Message message)` Verifies a signed `Message` using the signer's private key, a message hash, and a decryption parameter generated using the signer's private key and the message hash. |

    Methods in edu.ecu.hsim.ray.proxyreencryption with parameters of type Message

    | Modifier and Type | Method and Description |
    | --- | --- |
    |  |  |
    | --- | --- |
    | `Message` | AFGH.`decrypt(it.unisa.dia.gas.jpbc.Element x, Message message)` |
    | `abstract Message` | ProxyReEncryption.`decrypt(it.unisa.dia.gas.jpbc.Element x, Message message)` Decrypts the `Message` using the provided ephemeral key `x`. |
    | `Message` | AFGH.`decrypt(Keys key, Message message)` |
    | `abstract Message` | ProxyReEncryption.`decrypt(Keys keys, Message message)` Decrypts the `Message` using the provided private key. |
    | `Message` | AFGH.`decryptVerifyImpl(Keys verify, Keys decrypt, Message cipher)` Decrypt-verifies the `cipher` using the private key of the recipient (`decrypt`) and public key of the signer (`verify`), returning the decrypt-verified cipher as a `Message`. |
    | `Cipher` | AFGH.`encrypt(it.unisa.dia.gas.jpbc.Element x, Message message)` |
    | `abstract Cipher` | ProxyReEncryption.`encrypt(it.unisa.dia.gas.jpbc.Element x, Message message)` Encrypts the `Message` using the ephemeral key `x`. |
    | `Cipher` | AFGH.`encrypt(Keys keys, Message message)` |
    | `abstract Cipher` | ProxyReEncryption.`encrypt(Keys keys, Message message)` Encrypts the `Message` using the public key. |
    | `Cipher` | AFGH.`encrypt(Keys keys, Message message, ProxyReEncryption.REENCRYPT reencrypt)` |
    | `abstract Cipher` | ProxyReEncryption.`encrypt(Keys keys, Message message, ProxyReEncryption.REENCRYPT reencrypt)` Encrypts the `Message` using the public key and `reencrypt` parameter. |
    | `void` | AFGH.`scale(Message message, it.unisa.dia.gas.jpbc.Element from, it.unisa.dia.gas.jpbc.Element to, ProxyReEncryption.SCALE scaleType)` |
    | `abstract void` | ProxyReEncryption.`scale(Message message, it.unisa.dia.gas.jpbc.Element from, it.unisa.dia.gas.jpbc.Element to, ProxyReEncryption.SCALE scaleType)` Transforms the encryption key of the `Message` from `from` to `to` by way of simple scaling. |
    | `void` | AFGH.`scale(Message message, it.unisa.dia.gas.jpbc.Element scalar, ProxyReEncryption.SCALE scaleType)` |
    | `abstract void` | ProxyReEncryption.`scale(Message message, it.unisa.dia.gas.jpbc.Element scalar, ProxyReEncryption.SCALE scaleType)` Transforms the encryption key of the `Message` from `from` to `to` by way of simple scaling. |
    | `Cipher` | AFGH.`sign(Keys sign, Keys encrypt, Message message)` |
    | `abstract Cipher` | ProxyReEncryption.`sign(Keys sign, Keys encrypt, Message message)` Encrypt-signs the `Message` using an ephemeral key `x`, returning a `Cipher` that is decrypt-verified by its hash and a decryption parameter constructed using the signer's private key and recipients public key (all contained within the `Cipher`). |
    | `Cipher` | AFGH.`sign(Keys keys, Message message)` |
    | `abstract Cipher` | ProxyReEncryption.`sign(Keys keys, Message message)` Signs the `Message` using an ephemeral key `x`, returning a `Cipher` that is verified by its hash and a private key-based decryption parameter (all contained within the `Cipher`). |
    | `boolean` | AFGH.`verify(Keys keys, Cipher cipher, Message expected)` |
    | `abstract boolean` | ProxyReEncryption.`verify(Keys keys, Cipher cipher, Message expected)` Verifies a signed `Cipher` using the signer's private key, a message hash, and a decryption parameter generated using the signer's private key and the message hash. |
    | `boolean` | AFGH.`verify(Keys verify, Keys decrypt, Cipher cipher, Message expected)` |
    | `abstract boolean` | ProxyReEncryption.`verify(Keys verify, Keys decrypt, Cipher cipher, Message expected)` Decrypt-verifies a signed `Cipher` using the signer's private key, a message hash, and a decryption parameter generated using the signer's private key and the message hash. |
    | `boolean` | AFGH.`verify(Keys verify, Keys decrypt, Message message)` |
    | `abstract boolean` | ProxyReEncryption.`verify(Keys verify, Keys decrypt, Message message)` Decrypt-verifies a signed `Message` using the signer's private key, a message hash, and a decryption parameter generated using the signer's private key and the message hash. |
    | `boolean` | AFGH.`verify(Keys verify, Keys decrypt, Message message, byte[] expected)` |
    | `abstract boolean` | ProxyReEncryption.`verify(Keys verify, Keys decrypt, Message message, byte[] expected)` Decrypt-verifies a signed `Message` using the signer's private key, a message hash, and a decryption parameter generated using the signer's private key and the message hash. |
    | `boolean` | AFGH.`verify(Keys verify, Keys decrypt, Message message, Message expected)` |
    | `abstract boolean` | ProxyReEncryption.`verify(Keys verify, Keys decrypt, Message message, Message expected)` Decrypt-verifies a signed `Message` using the signer's private key, a message hash, and a decryption parameter generated using the signer's private key and the message hash. |
    | `boolean` | AFGH.`verify(Keys verify, Keys decrypt, Message message, java.lang.String expected)` |
    | `abstract boolean` | ProxyReEncryption.`verify(Keys verify, Keys decrypt, Message message, java.lang.String expected)` Decrypt-verifies a signed `Message` using the signer's private key, a message hash, and a decryption parameter generated using the signer's private key and the message hash. |
    | `boolean` | AFGH.`verify(Keys keys, Message message)` |
    | `abstract boolean` | ProxyReEncryption.`verify(Keys keys, Message message)` Verifies a signed `Message` using the signer's private key, a message hash, and a decryption parameter generated using the signer's private key and the message hash. |
    | `boolean` | AFGH.`verify(Keys keys, Message message, byte[] expected)` |
    | `abstract boolean` | ProxyReEncryption.`verify(Keys keys, Message message, byte[] expected)` Verifies a signed `Message` using the signer's private key, a message hash, and a decryption parameter generated using the signer's private key and the message hash. |
    | `boolean` | AFGH.`verify(Keys keys, Message message, Message expected)` |
    | `abstract boolean` | ProxyReEncryption.`verify(Keys keys, Message message, Message expected)` Verifies a signed `Message` using the signer's private key, a message hash, and a decryption parameter generated using the signer's private key and the message hash. |
    | `boolean` | AFGH.`verify(Keys keys, Message message, java.lang.String expected)` |
    | `abstract boolean` | ProxyReEncryption.`verify(Keys keys, Message message, java.lang.String expected)` Verifies a signed `Message` using the signer's private key, a message hash, and a decryption parameter generated using the signer's private key and the message hash. |
    | `Message` | AFGH.`verifyAndGet(Keys verify, Keys decrypt, Message message)` |
    | `abstract Message` | ProxyReEncryption.`verifyAndGet(Keys verify, Keys decrypt, Message message)` Decrypt-verifies a signed `Message` using the signer's private key, a message hash, and a decryption parameter generated using the signer's private key and the message hash. |
    | `Message` | AFGH.`verifyAndGet(Keys keys, Message message)` |
    | `abstract Message` | ProxyReEncryption.`verifyAndGet(Keys keys, Message message)` Verifies a signed `Message` using the signer's private key, a message hash, and a decryption parameter generated using the signer's private key and the message hash. |

Skip navigation links


- Package
- Class
- Use
- Tree
- Deprecated
- Index
- Help

- Prev
- Next

- Frames
- No Frames

- All Classes
