# Supplementary material for: A Blockchain Framework for Patient-Centered Health Records and Exchange (HealthChain): Evaluation and Proof-of-Concept Study
Source: J Med Internet Res. 2019 Aug 31;21(8):e13592. doi: 10.2196/13592 (PMC6743266; doi:10.2196/13592)
Supplement: Multimedia Appendix 3 [file jmir_v21i8e13592_app3.zip › ProxyReEncryption/javadoc/edu/ecu/hsim/ray/proxyreencryption/class-use/ProxyReEncryption.ENCRYPTION_MODE.html]

Uses of Class edu.ecu.hsim.ray.proxyreencryption.ProxyReEncryption.ENCRYPTION\_MODE


JavaScript is disabled on your browser.


Skip navigation links


- Package
- Class
- Use
- Tree
- Deprecated
- Index
- Help

- Prev
- Next

- Frames
- No Frames

- All Classes

## Uses of Class edu.ecu.hsim.ray.proxyreencryption.ProxyReEncryption.ENCRYPTION\_MODE

- - ### Uses of ProxyReEncryption.ENCRYPTION\_MODE in edu.ecu.hsim.ray.proxyreencryption

    Fields in edu.ecu.hsim.ray.proxyreencryption declared as ProxyReEncryption.ENCRYPTION\_MODE

    | Modifier and Type | Field and Description |
    | --- | --- |
    |  |  |
    | --- | --- |
    | `protected static ProxyReEncryption.ENCRYPTION_MODE` | ProxyReEncryption.`DEFAULT_ENCRYPTION_MODE` Default `ProxyReEncryption.ENCRYPTION_MODE`. |
    | `protected ProxyReEncryption.ENCRYPTION_MODE` | ProxyReEncryption.`encMode` `ProxyReEncryption.ENCRYPTION_MODE` parameter. |

    Fields in edu.ecu.hsim.ray.proxyreencryption with type parameters of type ProxyReEncryption.ENCRYPTION\_MODE

    | Modifier and Type | Field and Description |
    | --- | --- |
    |  |  |
    | --- | --- |
    | `static java.util.HashMap<java.lang.Integer,ProxyReEncryption.ENCRYPTION_MODE>` | ProxyReEncryption.`encModeHM` Maps `code` to `ProxyReEncryption.ENCRYPTION_MODE`. |

    Methods in edu.ecu.hsim.ray.proxyreencryption that return ProxyReEncryption.ENCRYPTION\_MODE

    | Modifier and Type | Method and Description |
    | --- | --- |
    |  |  |
    | --- | --- |
    | `ProxyReEncryption.ENCRYPTION_MODE` | ProxyReEncryption.`getEncryptionMode()` Returns the `ProxyReEncryption.ENCRYPTION_MODE`. |
    | `static ProxyReEncryption.ENCRYPTION_MODE` | ProxyReEncryption.ENCRYPTION\_MODE.`valueOf(java.lang.String name)` Returns the enum constant of this type with the specified name. |
    | `static ProxyReEncryption.ENCRYPTION_MODE[]` | ProxyReEncryption.ENCRYPTION\_MODE.`values()` Returns an array containing the constants of this enum type, in the order they are declared. |

    Methods in edu.ecu.hsim.ray.proxyreencryption with parameters of type ProxyReEncryption.ENCRYPTION\_MODE

    | Modifier and Type | Method and Description |
    | --- | --- |
    |  |  |
    | --- | --- |
    | `void` | ProxyReEncryption.`setEncryptionMode(ProxyReEncryption.ENCRYPTION_MODE encMode)` Set the `ProxyReEncryption.ENCRYPTION_MODE`. |

    Constructors in edu.ecu.hsim.ray.proxyreencryption with parameters of type ProxyReEncryption.ENCRYPTION\_MODE

    Constructor and Description

    `AFGH(AFGH.LENGTH length,
    byte[] curveParams,
    ProxyReEncryption.ENCRYPTION_MODE encMode)`

    Constructs a new `STORAGE#NONVOLATILE` `AFGH`.

    `AFGH(AFGH.LENGTH length,
    ProxyReEncryption.ENCRYPTION_MODE encMode)`

    Constructs a new `STORAGE#VOLATILE` `AFGH` with elliptic
    curve key length of size `length` and encryption mode of
    `encMode`.

    `AFGH(AFGH.LENGTH length,
    java.lang.String curveParams,
    ProxyReEncryption.ENCRYPTION_MODE encMode)`

    Constructs a new `STORAGE#NONVOLATILE` `AFGH`.

    `AFGH(byte[] curveParams,
    ProxyReEncryption.STORAGE storage,
    ProxyReEncryption.ENCRYPTION_MODE encMode)`

    Constructs a new `storage` `AFGH`.

    `AFGH(ProxyReEncryption.ENCRYPTION_MODE encMode)`

    Constructs a new `STORAGE#VOLATILE` `AFGH` with elliptic
    curve key length of size `AFGH.DEFAULT_LENGTH` and k mode of
    `encMode`.

    `AFGH(java.lang.String curveParams,
    ProxyReEncryption.STORAGE storage,
    ProxyReEncryption.ENCRYPTION_MODE encMode)`

    Constructs a new `storage` `AFGH`.

Skip navigation links


- Package
- Class
- Use
- Tree
- Deprecated
- Index
- Help

- Prev
- Next

- Frames
- No Frames

- All Classes
