# Supplementary material for: A Blockchain Framework for Patient-Centered Health Records and Exchange (HealthChain): Evaluation and Proof-of-Concept Study
Source: J Med Internet Res. 2019 Aug 31;21(8):e13592. doi: 10.2196/13592 (PMC6743266; doi:10.2196/13592)
Supplement: Multimedia Appendix 3 [file jmir_v21i8e13592_app3.zip › ProxyReEncryption/javadoc/edu/ecu/hsim/ray/proxyreencryption/class-use/ProxyReEncryption.html]

Uses of Class edu.ecu.hsim.ray.proxyreencryption.ProxyReEncryption


JavaScript is disabled on your browser.


Skip navigation links


- Package
- Class
- Use
- Tree
- Deprecated
- Index
- Help

- Prev
- Next

- Frames
- No Frames

- All Classes

## Uses of Class edu.ecu.hsim.ray.proxyreencryption.ProxyReEncryption

- - ### Uses of ProxyReEncryption in edu.ecu.hsim.ray.proxyreencryption

    Subclasses of ProxyReEncryption in edu.ecu.hsim.ray.proxyreencryption

    | Modifier and Type | Class and Description |
    | --- | --- |
    |  |  |
    | --- | --- |
    | `class` | `AFGH` Background |

    Fields in edu.ecu.hsim.ray.proxyreencryption declared as ProxyReEncryption

    | Modifier and Type | Field and Description |
    | --- | --- |
    |  |  |
    | --- | --- |
    | `protected ProxyReEncryption` | Cipher.`proxy` `ProxyReEncryption` object. |

    Methods in edu.ecu.hsim.ray.proxyreencryption that return ProxyReEncryption

    | Modifier and Type | Method and Description |
    | --- | --- |
    |  |  |
    | --- | --- |
    | `ProxyReEncryption` | Keys.`getProxy()` Returns the `ProxyReEncryption` object associated with this `Keys` object. |

    Constructors in edu.ecu.hsim.ray.proxyreencryption with parameters of type ProxyReEncryption

    Constructor and Description

    `Cipher(ProxyReEncryption proxy,
    Cipher.LEVEL level)`

    Constructor.

    `Cipher(ProxyReEncryption proxy,
    Cipher.LEVEL level,
    Cipher cipher,
    it.unisa.dia.gas.jpbc.Element reencryptionPairing)`

    Re-encryption constructor.

    `Cipher(ProxyReEncryption proxy,
    Cipher.LEVEL level,
    Cipher cipher,
    it.unisa.dia.gas.jpbc.Element reencryptionScalar,
    it.unisa.dia.gas.jpbc.Element reencryptionPairing)`

    Re-encryption constructor for two-party decryption This sets the
    intermediate's scalar and delegatee's re-encryption, which is used during
    the decryption process by the recipient.

    `Cipher(ProxyReEncryption proxy,
    Cipher.LEVEL level,
    it.unisa.dia.gas.jpbc.Element reencryptionPairing)`

    Re-encryption constructor.

    `Cipher(ProxyReEncryption proxy,
    Cipher.LEVEL level,
    it.unisa.dia.gas.jpbc.Element signedHash,
    it.unisa.dia.gas.jpbc.Element decryptionParameter)`

    Re-encryption constructor for sign/encrypt-sign constructor.

    `Keys(ProxyReEncryption proxy)`

    Constructs a new `ProxyReEncryption.STORAGE.VOLATILE` set of `Keys`.

    `Keys(ProxyReEncryption proxy,
    boolean placeholder)`

    Constructor for curve parameter generation testing.

    `Keys(ProxyReEncryption proxy,
    byte[] publicParams,
    byte[] secretParams)`

    Constructs a new `ProxyReEncryption.STORAGE.VOLATILE` set of `Keys`.

    `Keys(ProxyReEncryption proxy,
    java.lang.String paramsFile)`

    Constructs a new `ProxyReEncryption.STORAGE.NONVOLATILE` set of `Keys`.

    `Keys(ProxyReEncryption proxy,
    java.lang.String publicParams,
    java.lang.String secretParams)`

    Constructs a new `ProxyReEncryption.STORAGE.VOLATILE` set of `Keys`.

    `ReKeys(ProxyReEncryption proxy,
    java.lang.String params,
    ProxyReEncryption.STORAGE storage)`

    Constructs a new `storage` re-encryption key from an existing
    source - `params`.

Skip navigation links


- Package
- Class
- Use
- Tree
- Deprecated
- Index
- Help

- Prev
- Next

- Frames
- No Frames

- All Classes
