# Supplementary material for: A Blockchain Framework for Patient-Centered Health Records and Exchange (HealthChain): Evaluation and Proof-of-Concept Study
Source: J Med Internet Res. 2019 Aug 31;21(8):e13592. doi: 10.2196/13592 (PMC6743266; doi:10.2196/13592)
Supplement: Multimedia Appendix 3 [file jmir_v21i8e13592_app3.zip › ProxyReEncryption/javadoc/edu/ecu/hsim/ray/proxyreencryption/class-use/ProxyReEncryption.MESSAGE_TYPE.html]

Uses of Class edu.ecu.hsim.ray.proxyreencryption.ProxyReEncryption.MESSAGE\_TYPE


JavaScript is disabled on your browser.


Skip navigation links


- Package
- Class
- Use
- Tree
- Deprecated
- Index
- Help

- Prev
- Next

- Frames
- No Frames

- All Classes

## Uses of Class edu.ecu.hsim.ray.proxyreencryption.ProxyReEncryption.MESSAGE\_TYPE

- - ### Uses of ProxyReEncryption.MESSAGE\_TYPE in edu.ecu.hsim.ray.proxyreencryption

    Methods in edu.ecu.hsim.ray.proxyreencryption that return ProxyReEncryption.MESSAGE\_TYPE

    | Modifier and Type | Method and Description |
    | --- | --- |
    |  |  |
    | --- | --- |
    | `static ProxyReEncryption.MESSAGE_TYPE` | ProxyReEncryption.MESSAGE\_TYPE.`valueOf(java.lang.String name)` Returns the enum constant of this type with the specified name. |
    | `static ProxyReEncryption.MESSAGE_TYPE[]` | ProxyReEncryption.MESSAGE\_TYPE.`values()` Returns an array containing the constants of this enum type, in the order they are declared. |

    Methods in edu.ecu.hsim.ray.proxyreencryption with parameters of type ProxyReEncryption.MESSAGE\_TYPE

    | Modifier and Type | Method and Description |
    | --- | --- |
    |  |  |
    | --- | --- |
    | `Cipher` | AFGH.`encrypt(it.unisa.dia.gas.jpbc.Element x, byte[] message, ProxyReEncryption.MESSAGE_TYPE type)` |
    | `abstract Cipher` | ProxyReEncryption.`encrypt(it.unisa.dia.gas.jpbc.Element x, byte[] message, ProxyReEncryption.MESSAGE_TYPE type)` Encrypts the `message` using the ephemeral key `x`. |
    | `Cipher` | AFGH.`encrypt(Keys keys, byte[] message, ProxyReEncryption.MESSAGE_TYPE type)` |
    | `abstract Cipher` | ProxyReEncryption.`encrypt(Keys keys, byte[] message, ProxyReEncryption.MESSAGE_TYPE type)` Encrypts the `message` using the public key. |
    | `Cipher` | AFGH.`encrypt(Keys keys, byte[] message, ProxyReEncryption.MESSAGE_TYPE type, ProxyReEncryption.REENCRYPT reencrypt)` |
    | `abstract Cipher` | ProxyReEncryption.`encrypt(Keys keys, byte[] message, ProxyReEncryption.MESSAGE_TYPE type, ProxyReEncryption.REENCRYPT reencrypt)` Encrypts the `message` using the public key and `reencrypt` parameter. |
    | `Cipher` | AFGH.`sign(Keys keys, byte[] message, ProxyReEncryption.MESSAGE_TYPE type)` |
    | `abstract Cipher` | ProxyReEncryption.`sign(Keys keys, byte[] message, ProxyReEncryption.MESSAGE_TYPE type)` Signs the `message` using an ephemeral key `x`, returning a `Cipher` that is verified by its hash and a private key-based decryption parameter (all contained within the `Cipher`). |
    | `Cipher` | AFGH.`sign(Keys sign, Keys encrypt, byte[] message, ProxyReEncryption.MESSAGE_TYPE type)` |
    | `abstract Cipher` | ProxyReEncryption.`sign(Keys sign, Keys encrypt, byte[] message, ProxyReEncryption.MESSAGE_TYPE type)` Encrypt-signs the `message` using an ephemeral key `x`, returning a `Cipher` that is decrypt-verified by its hash and a decryption parameter constructed using the signer's private key and recipients public key (all contained within the `Cipher`). |
    | `Message` | AFGH.`toMessage(byte[] message, ProxyReEncryption.MESSAGE_TYPE type)` |
    | `abstract Message` | ProxyReEncryption.`toMessage(byte[] message, ProxyReEncryption.MESSAGE_TYPE type)` Converts a `byte[]` to a `Message`. |

Skip navigation links


- Package
- Class
- Use
- Tree
- Deprecated
- Index
- Help

- Prev
- Next

- Frames
- No Frames

- All Classes
