# Supplementary material for: A Blockchain Framework for Patient-Centered Health Records and Exchange (HealthChain): Evaluation and Proof-of-Concept Study
Source: J Med Internet Res. 2019 Aug 31;21(8):e13592. doi: 10.2196/13592 (PMC6743266; doi:10.2196/13592)
Supplement: Multimedia Appendix 3 [file jmir_v21i8e13592_app3.zip › ProxyReEncryption/javadoc/edu/ecu/hsim/ray/proxyreencryption/class-use/ProxyReEncryption.REENCRYPT.html]

Uses of Class edu.ecu.hsim.ray.proxyreencryption.ProxyReEncryption.REENCRYPT


JavaScript is disabled on your browser.


Skip navigation links


- Package
- Class
- Use
- Tree
- Deprecated
- Index
- Help

- Prev
- Next

- Frames
- No Frames

- All Classes

## Uses of Class edu.ecu.hsim.ray.proxyreencryption.ProxyReEncryption.REENCRYPT

- - ### Uses of ProxyReEncryption.REENCRYPT in edu.ecu.hsim.ray.proxyreencryption

    Fields in edu.ecu.hsim.ray.proxyreencryption declared as ProxyReEncryption.REENCRYPT

    | Modifier and Type | Field and Description |
    | --- | --- |
    |  |  |
    | --- | --- |
    | `protected ProxyReEncryption.REENCRYPT` | ProxyReEncryption.`DEFAULT_REENCRYPT` Default `ProxyReEncryption.REENCRYPT`. |

    Methods in edu.ecu.hsim.ray.proxyreencryption that return ProxyReEncryption.REENCRYPT

    | Modifier and Type | Method and Description |
    | --- | --- |
    |  |  |
    | --- | --- |
    | `ProxyReEncryption.REENCRYPT` | ProxyReEncryption.`getDefaultReEncrypt()` Returns the default re-encryption parameter. |
    | `static ProxyReEncryption.REENCRYPT` | ProxyReEncryption.REENCRYPT.`valueOf(java.lang.String name)` Returns the enum constant of this type with the specified name. |
    | `static ProxyReEncryption.REENCRYPT[]` | ProxyReEncryption.REENCRYPT.`values()` Returns an array containing the constants of this enum type, in the order they are declared. |

    Methods in edu.ecu.hsim.ray.proxyreencryption with parameters of type ProxyReEncryption.REENCRYPT

    | Modifier and Type | Method and Description |
    | --- | --- |
    |  |  |
    | --- | --- |
    | `Cipher` | AFGH.`encrypt(Keys keys, byte[] message, ProxyReEncryption.MESSAGE_TYPE type, ProxyReEncryption.REENCRYPT reencrypt)` |
    | `abstract Cipher` | ProxyReEncryption.`encrypt(Keys keys, byte[] message, ProxyReEncryption.MESSAGE_TYPE type, ProxyReEncryption.REENCRYPT reencrypt)` Encrypts the `message` using the public key and `reencrypt` parameter. |
    | `Cipher` | AFGH.`encrypt(Keys keys, Cipher cipher, ProxyReEncryption.REENCRYPT reencrypt)` |
    | `abstract Cipher` | ProxyReEncryption.`encrypt(Keys keys, Cipher cipher, ProxyReEncryption.REENCRYPT reencrypt)` Encrypts the `Cipher` using the public key and `reencrypt` parameter. |
    | `Cipher` | AFGH.`encrypt(Keys keys, Message message, ProxyReEncryption.REENCRYPT reencrypt)` |
    | `abstract Cipher` | ProxyReEncryption.`encrypt(Keys keys, Message message, ProxyReEncryption.REENCRYPT reencrypt)` Encrypts the `Message` using the public key and `reencrypt` parameter. |
    | `Cipher` | AFGH.`encrypt(Keys keys, java.lang.String message, ProxyReEncryption.REENCRYPT reencrypt)` |
    | `abstract Cipher` | ProxyReEncryption.`encrypt(Keys keys, java.lang.String message, ProxyReEncryption.REENCRYPT reencrypt)` Encrypts the `message` using the public key and `reencrypt` parameter. |
    | `void` | ProxyReEncryption.`setDefaultReEncrypt(ProxyReEncryption.REENCRYPT reencrypt)` Sets the default re-encryption parameter. |

Skip navigation links


- Package
- Class
- Use
- Tree
- Deprecated
- Index
- Help

- Prev
- Next

- Frames
- No Frames

- All Classes
