# Supplementary material for: A Blockchain Framework for Patient-Centered Health Records and Exchange (HealthChain): Evaluation and Proof-of-Concept Study
Source: J Med Internet Res. 2019 Aug 31;21(8):e13592. doi: 10.2196/13592 (PMC6743266; doi:10.2196/13592)
Supplement: Multimedia Appendix 3 [file jmir_v21i8e13592_app3.zip › ProxyReEncryption/javadoc/edu/ecu/hsim/ray/proxyreencryption/class-use/ReKeys.html]

Uses of Class edu.ecu.hsim.ray.proxyreencryption.ReKeys


JavaScript is disabled on your browser.


Skip navigation links


- Package
- Class
- Use
- Tree
- Deprecated
- Index
- Help

- Prev
- Next

- Frames
- No Frames

- All Classes

## Uses of Class edu.ecu.hsim.ray.proxyreencryption.ReKeys

- - ### Uses of ReKeys in edu.ecu.hsim.ray.proxyreencryption

    Methods in edu.ecu.hsim.ray.proxyreencryption that return ReKeys

    | Modifier and Type | Method and Description |
    | --- | --- |
    |  |  |
    | --- | --- |
    | `ReKeys` | ProxyReEncryption.`newReKeys(Keys delegator, Keys delegatee)` Constructs a new `ProxyReEncryption.STORAGE.VOLATILE` re-encryption key. |
    | `ReKeys` | ProxyReEncryption.`newReKeys(Keys delegator, Keys intermediary, Keys delegatee)` Constructs a new two-party `ProxyReEncryption.STORAGE.VOLATILE` re-encryption key. |
    | `ReKeys` | ProxyReEncryption.`newReKeys(Keys delegator, Keys intermediary, Keys delegatee, java.lang.String params)` Constructs a new two-party `ProxyReEncryption.STORAGE.NONVOLATILE` re-encryption key. |
    | `ReKeys` | ProxyReEncryption.`newReKeys(Keys delegator, Keys delegatee, java.lang.String params)` Constructs a new `ProxyReEncryption.STORAGE.NONVOLATILE` re-encryption key. |
    | `ReKeys` | ProxyReEncryption.`newReKeys(java.lang.String params, ProxyReEncryption.STORAGE storage)` Constructs a new `storage` re-encryption key from an existing source - `params`. |

Skip navigation links


- Package
- Class
- Use
- Tree
- Deprecated
- Index
- Help

- Prev
- Next

- Frames
- No Frames

- All Classes
