# Supplementary material for: A Blockchain Framework for Patient-Centered Health Records and Exchange (HealthChain): Evaluation and Proof-of-Concept Study
Source: J Med Internet Res. 2019 Aug 31;21(8):e13592. doi: 10.2196/13592 (PMC6743266; doi:10.2196/13592)
Supplement: Multimedia Appendix 3 [file jmir_v21i8e13592_app3.zip › ProxyReEncryption/javadoc/edu/ecu/hsim/ray/proxyreencryption/Constants.html]

Constants


JavaScript is disabled on your browser.


Skip navigation links


- Package
- Class
- Use
- Tree
- Deprecated
- Index
- Help

- Prev Class
- Next Class

- Frames
- No Frames

- All Classes

- Summary:
- Nested |
- Field |
- Constr |
- Method

- Detail:
- Field |
- Constr |
- Method


edu.ecu.hsim.ray.proxyreencryption

## Class Constants

- java.lang.Object
- - edu.ecu.hsim.ray.proxyreencryption.Constants

- ---

    

  ```
  public class Constants
  extends java.lang.Object
  ```

  Constants.

- - ### Field Summary

    Fields

    | Modifier and Type | Field and Description |
    | --- | --- |
    | `static java.lang.String` | `CIPHER_EXT` Cipher file extension. |
    | `static java.lang.String` | `CURVE_PARAMS_EXT` Curve parameters file extension. |
    | `static java.lang.String` | `DOI` Sample text - the Declaration of Independence. |
    | `static java.lang.String` | `ENC_MODE_PREFIX` Encryption mode key. |
    | `static java.nio.charset.Charset` | `ENCODING` `Charset` encoding. |
    | `static java.lang.String` | `EQ_PREFIX_I` Eq data key. |
    | `static java.lang.String` | `EQ_PREFIX_N` Eq Base64 size key. |
    | `static java.lang.String` | `G1P_PREFIX` G1 preprocessing key |
    | `static java.lang.String` | `G1X_PREFIX` G1.x key. |
    | `static java.lang.String` | `G1Y_PREFIX` G1.y key. |
    | `static java.lang.String` | `G2P_PREFIX` G2 preprocessing key. |
    | `static java.lang.String` | `G2X_PREFIX` G2.x key. |
    | `static java.lang.String` | `G2Y_PREFIX` G2.y key. |
    | `static java.lang.String` | `GEN_PREFIX_I` Generator data key. |
    | `static java.lang.String` | `GEN_PREFIX_N` Generator Base64 size key. |
    | `static java.lang.String` | `KEY_K` K key. |
    | `static java.lang.String` | `KEY_L` L key. |
    | `static java.lang.String` | `KEY_V` Secret key `BigInteger` key. |
    | `static java.lang.String` | `KEY_Z` Secret key Z field order key. |
    | `static java.lang.String` | `MESSAGE_EXT` Message file extension. |
    | `static java.lang.String` | `PK_EXT` Public properties file extensions. |
    | `static java.lang.String` | `PK_PREFIX` Public key key. |
    | `static java.lang.String` | `RK_ENCP` Re-encryption key encryption parameter key. |
    | `static java.lang.String` | `RK_EXT` Re-encryption key file extension. |
    | `static java.lang.String` | `RK_RKEY` Re-encryption key key. |
    | `static java.lang.String` | `RK_TPS` Two-party scalar. |
    | `static java.lang.String` | `SK_EXT` Secret properties file extension. |
    | `static java.lang.String` | `ZP_PREFIX` Zp key. |
    | `static java.lang.String` | `ZR_FQ_PREFIX` Zr key. |
  - ### Constructor Summary

    Constructors

    Constructor and Description

    `Constants()`
  - ### Method Summary

    - ### Methods inherited from class java.lang.Object

      `clone, equals, finalize, getClass, hashCode, notify, notifyAll, toString, wait, wait, wait`

- - ### Field Detail


    - #### ENCODING

      ```
      public static final java.nio.charset.Charset ENCODING
      ```

      `Charset` encoding.


    - #### CIPHER\_EXT

      ```
      public static final java.lang.String CIPHER_EXT
      ```

      Cipher file extension.

      See Also:
      :   Constant Field Values


    - #### MESSAGE\_EXT

      ```
      public static final java.lang.String MESSAGE_EXT
      ```

      Message file extension.

      See Also:
      :   Constant Field Values


    - #### CURVE\_PARAMS\_EXT

      ```
      public static final java.lang.String CURVE_PARAMS_EXT
      ```

      Curve parameters file extension.

      See Also:
      :   Constant Field Values


    - #### PK\_EXT

      ```
      public static final java.lang.String PK_EXT
      ```

      Public properties file extensions.

      See Also:
      :   Constant Field Values


    - #### RK\_EXT

      ```
      public static final java.lang.String RK_EXT
      ```

      Re-encryption key file extension.

      See Also:
      :   Constant Field Values


    - #### SK\_EXT

      ```
      public static final java.lang.String SK_EXT
      ```

      Secret properties file extension.

      See Also:
      :   Constant Field Values


    - #### ZR\_FQ\_PREFIX

      ```
      public static final java.lang.String ZR_FQ_PREFIX
      ```

      Zr key.

      See Also:
      :   Constant Field Values


    - #### EQ\_PREFIX\_N

      ```
      public static final java.lang.String EQ_PREFIX_N
      ```

      Eq Base64 size key.

      See Also:
      :   Constant Field Values


    - #### EQ\_PREFIX\_I

      ```
      public static final java.lang.String EQ_PREFIX_I
      ```

      Eq data key.

      See Also:
      :   Constant Field Values


    - #### ENC\_MODE\_PREFIX

      ```
      public static final java.lang.String ENC_MODE_PREFIX
      ```

      Encryption mode key.

      See Also:
      :   Constant Field Values


    - #### GEN\_PREFIX\_N

      ```
      public static final java.lang.String GEN_PREFIX_N
      ```

      Generator Base64 size key.

      See Also:
      :   Constant Field Values


    - #### GEN\_PREFIX\_I

      ```
      public static final java.lang.String GEN_PREFIX_I
      ```

      Generator data key.

      See Also:
      :   Constant Field Values


    - #### G1X\_PREFIX

      ```
      public static final java.lang.String G1X_PREFIX
      ```

      G1.x key.

      See Also:
      :   Constant Field Values


    - #### G1Y\_PREFIX

      ```
      public static final java.lang.String G1Y_PREFIX
      ```

      G1.y key.

      See Also:
      :   Constant Field Values


    - #### G2X\_PREFIX

      ```
      public static final java.lang.String G2X_PREFIX
      ```

      G2.x key.

      See Also:
      :   Constant Field Values


    - #### G2Y\_PREFIX

      ```
      public static final java.lang.String G2Y_PREFIX
      ```

      G2.y key.

      See Also:
      :   Constant Field Values


    - #### G1P\_PREFIX

      ```
      public static final java.lang.String G1P_PREFIX
      ```

      G1 preprocessing key

      See Also:
      :   Constant Field Values


    - #### G2P\_PREFIX

      ```
      public static final java.lang.String G2P_PREFIX
      ```

      G2 preprocessing key.

      See Also:
      :   Constant Field Values


    - #### ZP\_PREFIX

      ```
      public static final java.lang.String ZP_PREFIX
      ```

      Zp key.

      See Also:
      :   Constant Field Values


    - #### PK\_PREFIX

      ```
      public static final java.lang.String PK_PREFIX
      ```

      Public key key.

      See Also:
      :   Constant Field Values


    - #### RK\_RKEY

      ```
      public static final java.lang.String RK_RKEY
      ```

      Re-encryption key key.

      See Also:
      :   Constant Field Values


    - #### RK\_ENCP

      ```
      public static final java.lang.String RK_ENCP
      ```

      Re-encryption key encryption parameter key.

      See Also:
      :   Constant Field Values


    - #### RK\_TPS

      ```
      public static final java.lang.String RK_TPS
      ```

      Two-party scalar.

      See Also:
      :   Constant Field Values


    - #### KEY\_Z

      ```
      public static final java.lang.String KEY_Z
      ```

      Secret key Z field order key.

      See Also:
      :   Constant Field Values


    - #### KEY\_V

      ```
      public static final java.lang.String KEY_V
      ```

      Secret key `BigInteger` key.

      See Also:
      :   Constant Field Values


    - #### KEY\_K

      ```
      public static final java.lang.String KEY_K
      ```

      K key.

      See Also:
      :   Constant Field Values


    - #### KEY\_L

      ```
      public static final java.lang.String KEY_L
      ```

      L key.

      See Also:
      :   Constant Field Values


    - #### DOI

      ```
      public static final java.lang.String DOI
      ```

      Sample text - the Declaration of Independence.

      See Also:
      :   Constant Field Values
  - ### Constructor Detail


    - #### Constants

      ```
      public Constants()
      ```


Skip navigation links


- Package
- Class
- Use
- Tree
- Deprecated
- Index
- Help

- Prev Class
- Next Class

- Frames
- No Frames

- All Classes

- Summary:
- Nested |
- Field |
- Constr |
- Method

- Detail:
- Field |
- Constr |
- Method
