# Supplementary material for: A Blockchain Framework for Patient-Centered Health Records and Exchange (HealthChain): Evaluation and Proof-of-Concept Study
Source: J Med Internet Res. 2019 Aug 31;21(8):e13592. doi: 10.2196/13592 (PMC6743266; doi:10.2196/13592)
Supplement: Multimedia Appendix 3 [file jmir_v21i8e13592_app3.zip › ProxyReEncryption/javadoc/edu/ecu/hsim/ray/proxyreencryption/Keys.html]

Keys


JavaScript is disabled on your browser.


Skip navigation links


- Package
- Class
- Use
- Tree
- Deprecated
- Index
- Help

- Prev Class
- Next Class

- Frames
- No Frames

- All Classes

- Summary:
- Nested |
- Field |
- Constr |
- Method

- Detail:
- Field |
- Constr |
- Method


edu.ecu.hsim.ray.proxyreencryption

## Class Keys

- java.lang.Object
- - edu.ecu.hsim.ray.proxyreencryption.Keys

- Direct Known Subclasses:
  :   ReKeys

  ---

    

  ```
  public class Keys
  extends java.lang.Object
  ```

  Generates/reads public and secret (private) keys. For situations when only
  a public key is known (e.g., Alice has Bob's public key, but not his
  secret key), `publicKey` will be populated and `secretKey`
  will be `null`.

- - ### Field Summary

    Fields

    | Modifier and Type | Field and Description |
    | --- | --- |
    | `protected it.unisa.dia.gas.jpbc.Element` | `firstLevel` First-level `Element`. |
    | `protected java.io.FileInputStream` | `fisPub` Public properties `FileInputStream`. |
    | `protected java.io.FileInputStream` | `fisSec` Secret properties `FileInputStream`. |
    | `protected java.io.FileOutputStream` | `fosPub` Public properties `FileOutputStream`. |
    | `protected java.io.FileOutputStream` | `fosSec` Secret properties `FileOutputStream`. |
    | `protected it.unisa.dia.gas.jpbc.Element` | `k` Secret parameter `k` `Element`. |
    | `protected it.unisa.dia.gas.jpbc.Element` | `l` Secret parameter `l` `Element`. |
    | `protected java.io.File` | `publicFile` Public properties `File`. |
    | `protected it.unisa.dia.gas.jpbc.Element` | `publicKey` Public key `Element`. |
    | `protected java.util.Properties` | `publicProps` Public `Properties` file. |
    | `protected java.lang.String` | `publicStringProperties` Public properties as `String`. |
    | `protected it.unisa.dia.gas.jpbc.Element` | `secondLevel` Second-level `Element`. |
    | `protected java.io.File` | `secretFile` Secret properties `File`. |
    | `protected it.unisa.dia.gas.jpbc.Element` | `secretKey` Secrete key `Element`. |
    | `protected it.unisa.dia.gas.jpbc.Element` | `secretKeyI` Secret key inverse `Element`. |
    | `protected java.util.Properties` | `secretProps` Secret `Properties` file. |
    | `protected java.lang.String` | `secretStringProperties` Secret properties as `String`. |
    | `protected java.io.StringReader` | `sisPub` Public properties `StringReader`. |
    | `protected java.io.StringReader` | `sisSec` Secret properties `StringReader`. |
    | `protected java.io.StringWriter` | `sosPub` Public properties `StringWriter`. |
    | `protected java.io.StringWriter` | `sosSec` Secret properties `StringWriter`. |
    | `protected ProxyReEncryption.STORAGE` | `storage` `ProxyReEncryption.STORAGE` parameter. |
    | `protected it.unisa.dia.gas.jpbc.Element` | `twoPartyScalar` Two-party re-encryption scalar `Element`. |
  - ### Constructor Summary

    Constructors

    | Modifier | Constructor and Description |
    | --- | --- |
    | `protected` | `Keys()` Empty constructor used by `ReKeys` as it follows a different process during its construction. |
    | `protected` | `Keys(ProxyReEncryption proxy)` Constructs a new `ProxyReEncryption.STORAGE.VOLATILE` set of `Keys`. |
    | `protected` | `Keys(ProxyReEncryption proxy, boolean placeholder)` Constructor for curve parameter generation testing. |
    | `protected` | `Keys(ProxyReEncryption proxy, byte[] publicParams, byte[] secretParams)` Constructs a new `ProxyReEncryption.STORAGE.VOLATILE` set of `Keys`. |
    | `protected` | `Keys(ProxyReEncryption proxy, java.lang.String paramsFile)` Constructs a new `ProxyReEncryption.STORAGE.NONVOLATILE` set of `Keys`. |
    | `protected` | `Keys(ProxyReEncryption proxy, java.lang.String publicParams, java.lang.String secretParams)` Constructs a new `ProxyReEncryption.STORAGE.VOLATILE` set of `Keys`. |
  - ### Method Summary

    All Methods Instance Methods Concrete Methods

    | Modifier and Type | Method and Description |
    | --- | --- |
    | `void` | `close()` Closes the file input and output streams. |
    | `it.unisa.dia.gas.jpbc.Element` | `getDecryptFirstLevelDenominator()` Returns the first-level decryption denominator as an immutable `Element`. |
    | `it.unisa.dia.gas.jpbc.Element` | `getDecryptSecondLevelDenominator()` Returns the second-level decryption denominator as an immutable `Element`. |
    | `it.unisa.dia.gas.jpbc.Element` | `getFirstLevel()` Returns `firstLevel`. |
    | `it.unisa.dia.gas.jpbc.Element` | `getK()` Returns a static `k`. |
    | `it.unisa.dia.gas.jpbc.Element` | `getL()` Returns `l`. |
    | `ProxyReEncryption` | `getProxy()` Returns the `ProxyReEncryption` object associated with this `Keys` object. |
    | `java.io.File` | `getPublicFile()` Returns the public file if `storage` == `ProxyReEncryption.STORAGE.NONVOLATILE`. |
    | `it.unisa.dia.gas.jpbc.Element` | `getPublicKey()` Returns the public key. |
    | `java.lang.String` | `getPublicProperties()` Returns public properties as a `String`. |
    | `it.unisa.dia.gas.jpbc.Element` | `getSecondLevel()` Returns `secondLevel`. |
    | `java.io.File` | `getSecretFile()` Returns the secret file if `storage` == `ProxyReEncryption.STORAGE.NONVOLATILE`. |
    | `it.unisa.dia.gas.jpbc.Element` | `getSecretKey()` Returns the secret key. |
    | `it.unisa.dia.gas.jpbc.Element` | `getSecretKeyInverse()` Returns the inverse of the secret key. |
    | `java.lang.String` | `getSecretProperties()` Returns secret properties as a `String`. |
    | `it.unisa.dia.gas.jpbc.Element` | `getTwoPartyScalar()` Returns `twoPartyScalar`. |
    | `it.unisa.dia.gas.jpbc.Element` | `newK()` Generates, saves, and returns a new `k`. |

    - ### Methods inherited from class java.lang.Object

      `clone, equals, finalize, getClass, hashCode, notify, notifyAll, toString, wait, wait, wait`

- - ### Field Detail


    - #### secretKey

      ```
      protected it.unisa.dia.gas.jpbc.Element secretKey
      ```

      Secrete key `Element`.


    - #### secretKeyI

      ```
      protected it.unisa.dia.gas.jpbc.Element secretKeyI
      ```

      Secret key inverse `Element`.


    - #### publicKey

      ```
      protected it.unisa.dia.gas.jpbc.Element publicKey
      ```

      Public key `Element`.


    - #### k

      ```
      protected it.unisa.dia.gas.jpbc.Element k
      ```

      Secret parameter `k` `Element`.


    - #### l

      ```
      protected it.unisa.dia.gas.jpbc.Element l
      ```

      Secret parameter `l` `Element`.


    - #### firstLevel

      ```
      protected it.unisa.dia.gas.jpbc.Element firstLevel
      ```

      First-level `Element`.


    - #### secondLevel

      ```
      protected it.unisa.dia.gas.jpbc.Element secondLevel
      ```

      Second-level `Element`.


    - #### twoPartyScalar

      ```
      protected it.unisa.dia.gas.jpbc.Element twoPartyScalar
      ```

      Two-party re-encryption scalar `Element`.


    - #### publicProps

      ```
      protected java.util.Properties publicProps
      ```

      Public `Properties` file.


    - #### secretProps

      ```
      protected java.util.Properties secretProps
      ```

      Secret `Properties` file.


    - #### publicFile

      ```
      protected java.io.File publicFile
      ```

      Public properties `File`.


    - #### secretFile

      ```
      protected java.io.File secretFile
      ```

      Secret properties `File`.


    - #### fisPub

      ```
      protected java.io.FileInputStream fisPub
      ```

      Public properties `FileInputStream`.


    - #### fisSec

      ```
      protected java.io.FileInputStream fisSec
      ```

      Secret properties `FileInputStream`.


    - #### fosPub

      ```
      protected java.io.FileOutputStream fosPub
      ```

      Public properties `FileOutputStream`.


    - #### fosSec

      ```
      protected java.io.FileOutputStream fosSec
      ```

      Secret properties `FileOutputStream`.


    - #### publicStringProperties

      ```
      protected java.lang.String publicStringProperties
      ```

      Public properties as `String`.


    - #### secretStringProperties

      ```
      protected java.lang.String secretStringProperties
      ```

      Secret properties as `String`.


    - #### sisPub

      ```
      protected java.io.StringReader sisPub
      ```

      Public properties `StringReader`.


    - #### sisSec

      ```
      protected java.io.StringReader sisSec
      ```

      Secret properties `StringReader`.


    - #### sosPub

      ```
      protected java.io.StringWriter sosPub
      ```

      Public properties `StringWriter`.


    - #### sosSec

      ```
      protected java.io.StringWriter sosSec
      ```

      Secret properties `StringWriter`.


    - #### storage

      ```
      protected ProxyReEncryption.STORAGE storage
      ```

      `ProxyReEncryption.STORAGE` parameter.
  - ### Constructor Detail


    - #### Keys

      ```
      protected Keys()
      ```

      Empty constructor used by `ReKeys` as it follows a different
      process during its construction.


    - #### Keys

      ```
      protected Keys(ProxyReEncryption proxy,
                     boolean placeholder)
      ```

      Constructor for curve parameter generation testing. NOT to be used for
      any other purpose.

      Parameters:
      :   `proxy` - `ProxyReEncryption`
      :   `placeholder` - distinguish this from `Keys(ProxyReEncryption)`


    - #### Keys

      ```
      protected Keys(ProxyReEncryption proxy)
      ```

      Constructs a new `ProxyReEncryption.STORAGE.VOLATILE` set of `Keys`.

      Parameters:
      :   `proxy` - `ProxyReEncryption`


    - #### Keys

      ```
      protected Keys(ProxyReEncryption proxy,
                     java.lang.String paramsFile)
      ```

      Constructs a new `ProxyReEncryption.STORAGE.NONVOLATILE` set of `Keys`.

      Parameters:
      :   `proxy` - `ProxyReEncryption`
      :   `paramsFile` - parameters file name


    - #### Keys

      ```
      protected Keys(ProxyReEncryption proxy,
                     byte[] publicParams,
                     byte[] secretParams)
      ```

      Constructs a new `ProxyReEncryption.STORAGE.VOLATILE` set of `Keys`.

      Parameters:
      :   `proxy` - `ProxyReEncryption`
      :   `publicParams` - public parameters in `byte[]` form
      :   `secretParams` - secret parameters in `byte[]` form


    - #### Keys

      ```
      protected Keys(ProxyReEncryption proxy,
                     java.lang.String publicParams,
                     java.lang.String secretParams)
      ```

      Constructs a new `ProxyReEncryption.STORAGE.VOLATILE` set of `Keys`.

      Parameters:
      :   `proxy` - `ProxyReEncryption`
      :   `publicParams` - public parameters in `String` form
      :   `secretParams` - secret parameters in `String` form
  - ### Method Detail


    - #### close

      ```
      public void close()
                 throws java.io.IOException
      ```

      Closes the file input and output streams.

      Throws:
      :   `java.io.IOException` - `IOException`


    - #### getDecryptFirstLevelDenominator

      ```
      public it.unisa.dia.gas.jpbc.Element getDecryptFirstLevelDenominator()
      ```

      Returns the first-level decryption denominator as an immutable
      `Element`.

      Returns:
      :   the first-level decryption denominator as an immutable
          `Element`.


    - #### getDecryptSecondLevelDenominator

      ```
      public it.unisa.dia.gas.jpbc.Element getDecryptSecondLevelDenominator()
      ```

      Returns the second-level decryption denominator as an immutable
      `Element`.

      Returns:
      :   the second-level decryption denominator as an immutable
          `Element`.


    - #### getFirstLevel

      ```
      public it.unisa.dia.gas.jpbc.Element getFirstLevel()
      ```

      Returns `firstLevel`.

      Returns:
      :   `firstLevel`


    - #### getSecondLevel

      ```
      public it.unisa.dia.gas.jpbc.Element getSecondLevel()
      ```

      Returns `secondLevel`.

      Returns:
      :   `secondLevel`


    - #### getTwoPartyScalar

      ```
      public it.unisa.dia.gas.jpbc.Element getTwoPartyScalar()
      ```

      Returns `twoPartyScalar`.

      Returns:
      :   `twoPartyScalar`


    - #### getK

      ```
      public it.unisa.dia.gas.jpbc.Element getK()
      ```

      Returns a static `k`.

      Returns:
      :   a static `k`


    - #### newK

      ```
      public it.unisa.dia.gas.jpbc.Element newK()
      ```

      Generates, saves, and returns a new `k`.

      Returns:
      :   a new `k`


    - #### getL

      ```
      public it.unisa.dia.gas.jpbc.Element getL()
      ```

      Returns `l`.

      Returns:
      :   `l`


    - #### getPublicKey

      ```
      public it.unisa.dia.gas.jpbc.Element getPublicKey()
      ```

      Returns the public key.

      Returns:
      :   the public key


    - #### getSecretKey

      ```
      public it.unisa.dia.gas.jpbc.Element getSecretKey()
      ```

      Returns the secret key.

      Returns:
      :   the secret key


    - #### getSecretKeyInverse

      ```
      public it.unisa.dia.gas.jpbc.Element getSecretKeyInverse()
      ```

      Returns the inverse of the secret key.

      Returns:
      :   the inverse of the secret key


    - #### getPublicFile

      ```
      public java.io.File getPublicFile()
      ```

      Returns the public file if `storage` ==
      `ProxyReEncryption.STORAGE.NONVOLATILE`.

      Returns:
      :   the public file


    - #### getSecretFile

      ```
      public java.io.File getSecretFile()
      ```

      Returns the secret file if `storage` ==
      `ProxyReEncryption.STORAGE.NONVOLATILE`.

      Returns:
      :   the secret file


    - #### getPublicProperties

      ```
      public java.lang.String getPublicProperties()
      ```

      Returns public properties as a `String`.

      Returns:
      :   public properties as a `String`


    - #### getSecretProperties

      ```
      public java.lang.String getSecretProperties()
      ```

      Returns secret properties as a `String`.

      Returns:
      :   secret properties as a `String`


    - #### getProxy

      ```
      public ProxyReEncryption getProxy()
      ```

      Returns the `ProxyReEncryption` object associated with this
      `Keys` object.

      Returns:
      :   associated `ProxyReEncryption`


Skip navigation links


- Package
- Class
- Use
- Tree
- Deprecated
- Index
- Help

- Prev Class
- Next Class

- Frames
- No Frames

- All Classes

- Summary:
- Nested |
- Field |
- Constr |
- Method

- Detail:
- Field |
- Constr |
- Method
