# Supplementary material for: A Blockchain Framework for Patient-Centered Health Records and Exchange (HealthChain): Evaluation and Proof-of-Concept Study
Source: J Med Internet Res. 2019 Aug 31;21(8):e13592. doi: 10.2196/13592 (PMC6743266; doi:10.2196/13592)
Supplement: Multimedia Appendix 3 [file jmir_v21i8e13592_app3.zip › ProxyReEncryption/javadoc/edu/ecu/hsim/ray/proxyreencryption/Message.html]

Message


JavaScript is disabled on your browser.


Skip navigation links


- Package
- Class
- Use
- Tree
- Deprecated
- Index
- Help

- Prev Class
- Next Class

- Frames
- No Frames

- All Classes

- Summary:
- Nested |
- Field |
- Constr |
- Method

- Detail:
- Field |
- Constr |
- Method


edu.ecu.hsim.ray.proxyreencryption

## Class Message

- java.lang.Object
- - edu.ecu.hsim.ray.proxyreencryption.Message

- ---

    

  ```
  public class Message
  extends java.lang.Object
  ```

  Stores message data as a list of elements and lengths. Each `Element`
  has a maximum capacity of the length in bytes of the
  `Field` `ProxyReEncryption.G2`. Appending to an existing
  `Message` is trivially accomplished via `add(Element, int)`.

- - ### Constructor Summary

    Constructors

    Constructor and Description

    `Message(int byteLength)`

    Constructor.
  - ### Method Summary

    All Methods Instance Methods Concrete Methods

    | Modifier and Type | Method and Description |
    | --- | --- |
    | `void` | `add(it.unisa.dia.gas.jpbc.Element element, int length)` Add an `Element`. |
    | `int` | `getByteLength()` Returns the byte length. |
    | `java.util.List<it.unisa.dia.gas.jpbc.Element>` | `getElements()` Returns the list of elements. |
    | `int` | `getLength()` Returns the total length of all elements. |
    | `java.util.List<java.lang.Integer>` | `getLengths()` Returns the list of lengths. |
    | `byte[]` | `toBytes()` Converts a `Message` to a `byte[]`. |
    | `byte[]` | `toBytesDataOnly()` Converts a `Message` to a `byte[]` of the data only (no metadata). |
    | `protected void` | `toBytesDataOnly(java.io.ByteArrayOutputStream baos)` Converts a `Message` to a `byte[]` of the data only (no metadata), stored in the `baos` `ByteArrayOutputStream` parameter. |
    | `byte[]` | `toBytesDataOnly(int bytePadding)` Converts a `Message` to a `byte[]` of the data only (no metadata), extracting any added padding via `ProxyReEncryption.toMessage(byte[], int)`. |
    | `byte[]` | `toHashBytes()` Converts a `Message` to a `byte[]` suitable for hashing. |
    | `java.lang.String` | `toString()` |
    | `void` | `write(java.lang.String file)` Writes `this` to a file. |

    - ### Methods inherited from class java.lang.Object

      `clone, equals, finalize, getClass, hashCode, notify, notifyAll, wait, wait, wait`

- - ### Constructor Detail


    - #### Message

      ```
      public Message(int byteLength)
      ```

      Constructor.

      Parameters:
      :   `byteLength` - the number of bytes in each element
  - ### Method Detail


    - #### add

      ```
      public void add(it.unisa.dia.gas.jpbc.Element element,
                      int length)
      ```

      Add an `Element`.

      Parameters:
      :   `element` - `Element` to add
      :   `length` - number of characters


    - #### getByteLength

      ```
      public int getByteLength()
      ```

      Returns the byte length.

      Returns:
      :   the byte length


    - #### getElements

      ```
      public java.util.List<it.unisa.dia.gas.jpbc.Element> getElements()
      ```

      Returns the list of elements.

      Returns:
      :   the list of elements


    - #### getLength

      ```
      public int getLength()
      ```

      Returns the total length of all elements.

      Returns:
      :   the total length of all elements


    - #### getLengths

      ```
      public java.util.List<java.lang.Integer> getLengths()
      ```

      Returns the list of lengths.

      Returns:
      :   the list of lengths


    - #### toBytes

      ```
      public byte[] toBytes()
      ```

      Converts a `Message` to a `byte[]`. This includes the
      metadata associated with the `Message` object. Useful for
      serialization of the object. For just the data elements (i.e., no
      metadata), use `toBytesDataOnly()` or
      `toBytesDataOnly(ByteArrayOutputStream)`.

      Returns:
      :   `Message` as a `byte[]`


    - #### toBytesDataOnly

      ```
      public byte[] toBytesDataOnly()
      ```

      Converts a `Message` to a `byte[]` of the data only (no
      metadata). This CANNOT be reversed. It is useful when encrypting
      parameters and all you need from the decryption step is the value, not
      a `Message` object. If serialization and deserialization of the
      `Message` is required, call `toBytes()`.

      Returns:
      :   `Message` as a `byte[]`


    - #### toBytesDataOnly

      ```
      public byte[] toBytesDataOnly(int bytePadding)
      ```

      Converts a `Message` to a `byte[]` of the data only (no
      metadata), extracting any added padding via
      `ProxyReEncryption.toMessage(byte[], int)`. This CANNOT be
      reversed. It is useful when encrypting parameters and all you need from
      the decryption step is the value, not a `Message` object. If
      serialization and deserialization of the `Message` is required,
      call `toBytes()`.

      Parameters:
      :   `bytePadding` - the number of leading bytes of padding to remove from
          a converted element (either single chunk or X/Y pairs)

      Returns:
      :   `Message` as a `byte[]`


    - #### toBytesDataOnly

      ```
      protected void toBytesDataOnly(java.io.ByteArrayOutputStream baos)
      ```

      Converts a `Message` to a `byte[]` of the data only (no
      metadata), stored in the `baos` `ByteArrayOutputStream`
      parameter. This CANNOT be reversed. It is useful when encrypting
      parameters and all you need from the decryption step is the value, not a
      `Message` object. If serialization and deserialization of the
      `Message` is required, call `toBytes()`.

      Parameters:
      :   `baos` - `ByteArrayOutputStream`


    - #### toHashBytes

      ```
      public byte[] toHashBytes()
      ```

      Converts a `Message` to a `byte[]` suitable for hashing. This
      CANNOT be reversed.

      Returns:
      :   `Message` as a `byte[]`


    - #### toString

      ```
      public java.lang.String toString()
      ```

      Overrides:
      :   `toString` in class `java.lang.Object`


    - #### write

      ```
      public void write(java.lang.String file)
      ```

      Writes `this` to a file.

      Parameters:
      :   `file` - file name


Skip navigation links


- Package
- Class
- Use
- Tree
- Deprecated
- Index
- Help

- Prev Class
- Next Class

- Frames
- No Frames

- All Classes

- Summary:
- Nested |
- Field |
- Constr |
- Method

- Detail:
- Field |
- Constr |
- Method
