# Supplementary material for: A Blockchain Framework for Patient-Centered Health Records and Exchange (HealthChain): Evaluation and Proof-of-Concept Study
Source: J Med Internet Res. 2019 Aug 31;21(8):e13592. doi: 10.2196/13592 (PMC6743266; doi:10.2196/13592)
Supplement: Multimedia Appendix 3 [file jmir_v21i8e13592_app3.zip › ProxyReEncryption/javadoc/edu/ecu/hsim/ray/proxyreencryption/package-summary.html]

edu.ecu.hsim.ray.proxyreencryption


JavaScript is disabled on your browser.


Skip navigation links


- Package
- Class
- Use
- Tree
- Deprecated
- Index
- Help

- Prev Package
- Next Package

- Frames
- No Frames

- All Classes

# Package edu.ecu.hsim.ray.proxyreencryption

- Class Summary

  | Class | Description |
  | --- | --- |
  |  |  |
  | --- | --- |
  | AFGH | Background |
  | Cipher | Stores enciphered data as a list of elements and lengths. |
  | Constants | Constants. |
  | Keys | Generates/reads public and secret (private) keys. |
  | Message | Stores message data as a list of elements and lengths. |
  | ProxyReEncryption | Proxy re-encryption |
  | ReKeys | Re-encryption key generator, writer, and reader. |
  | TypeAPairingIO | Extension to `TypeAPairing` allowing for reading in parameters. |
- Enum Summary

  | Enum | Description |
  | --- | --- |
  |  |  |
  | --- | --- |
  | AFGH.LENGTH | The employed curve is a Type A pairing Montgomery curve with embedding degree of \(k=2\). |
  | Cipher.LEVEL | Flags each `Cipher` with the appropriate level. |
  | Cipher.PAIRING\_TYPE | When loading a pairing `Element`, one can specify if it is a formatted or unformatted byte array. |
  | ProxyReEncryption.ENCRYPTION\_MODE | Encryption mode options: Encryption mode refers to how the encryption keys will be treated. |
  | ProxyReEncryption.MESSAGE\_TYPE | Message `byte[]` type. |
  | ProxyReEncryption.REENCRYPT | Re-encryption type. |
  | ProxyReEncryption.SCALE | Scale input type. |
  | ProxyReEncryption.STORAGE | Storage volatility options: Storage volatility refers how the generated keys will be stored and read. |

Skip navigation links


- Package
- Class
- Use
- Tree
- Deprecated
- Index
- Help

- Prev Package
- Next Package

- Frames
- No Frames

- All Classes
