# Supplementary material for: A Blockchain Framework for Patient-Centered Health Records and Exchange (HealthChain): Evaluation and Proof-of-Concept Study
Source: J Med Internet Res. 2019 Aug 31;21(8):e13592. doi: 10.2196/13592 (PMC6743266; doi:10.2196/13592)
Supplement: Multimedia Appendix 3 [file jmir_v21i8e13592_app3.zip › ProxyReEncryption/javadoc/edu/ecu/hsim/ray/proxyreencryption/package-use.html]

Uses of Package edu.ecu.hsim.ray.proxyreencryption


JavaScript is disabled on your browser.


Skip navigation links


- Package
- Class
- Use
- Tree
- Deprecated
- Index
- Help

- Prev
- Next

- Frames
- No Frames

- All Classes

# Uses of Package edu.ecu.hsim.ray.proxyreencryption

- Classes in edu.ecu.hsim.ray.proxyreencryption used by edu.ecu.hsim.ray.proxyreencryption

  Class and Description

  AFGH.LENGTH

  The employed curve is a Type A pairing Montgomery curve with embedding
  degree of \(k=2\).

  Cipher

  Stores enciphered data as a list of elements and lengths.

  Cipher.LEVEL

  Flags each `Cipher` with the appropriate level.

  Cipher.PAIRING\_TYPE

  When loading a pairing `Element`, one can specify if it is a
  formatted or unformatted byte array.

  Keys

  Generates/reads public and secret (private) keys.

  Message

  Stores message data as a list of elements and lengths.

  ProxyReEncryption

  Proxy re-encryption

  ProxyReEncryption.ENCRYPTION\_MODE

  Encryption mode options:
  Encryption mode refers to how the encryption keys will be treated.

  ProxyReEncryption.MESSAGE\_TYPE

  Message `byte[]` type.

  ProxyReEncryption.REENCRYPT

  Re-encryption type.

  ProxyReEncryption.SCALE

  Scale input type.

  ProxyReEncryption.STORAGE

  Storage volatility options:
  Storage volatility refers how the generated keys will be stored and read.

  ReKeys

  Re-encryption key generator, writer, and reader.

Skip navigation links


- Package
- Class
- Use
- Tree
- Deprecated
- Index
- Help

- Prev
- Next

- Frames
- No Frames

- All Classes
