# Supplementary material for: A Blockchain Framework for Patient-Centered Health Records and Exchange (HealthChain): Evaluation and Proof-of-Concept Study
Source: J Med Internet Res. 2019 Aug 31;21(8):e13592. doi: 10.2196/13592 (PMC6743266; doi:10.2196/13592)
Supplement: Multimedia Appendix 3 [file jmir_v21i8e13592_app3.zip › ProxyReEncryption/javadoc/edu/ecu/hsim/ray/proxyreencryption/ProxyReEncryption.html]

ProxyReEncryption


JavaScript is disabled on your browser.


Skip navigation links


- Package
- Class
- Use
- Tree
- Deprecated
- Index
- Help

- Prev Class
- Next Class

- Frames
- No Frames

- All Classes

- Summary:
- Nested |
- Field |
- Constr |
- Method

- Detail:
- Field |
- Constr |
- Method


edu.ecu.hsim.ray.proxyreencryption

## Class ProxyReEncryption

- java.lang.Object
- - edu.ecu.hsim.ray.proxyreencryption.ProxyReEncryption

- Direct Known Subclasses:
  :   AFGH

  ---

    

  ```
  public abstract class ProxyReEncryption
  extends java.lang.Object
  ```

  ### Proxy re-encryption

  This class supports first-level (i.e., encrypted messages that CANNOT be
  re-encrypted) and second-level (i.e., encrypted messages that CAN be
  re-encrypted) encryption, first-level and second-level decryption, and
  re-encryption of second-level encrypted messages. The results of an
  encrypted/re-encrypted message is a `Cipher` object. The results of a
  decrypted message is a `Message` object.

  The default encryption level is first (`DEFAULT_REENCRYPT`). This can
  be overwritten either locally or globally. Locally: when encrypting a
  message, set the `ProxyReEncryption.REENCRYPT` parameter. Globally: set using
  `setDefaultReEncrypt(REENCRYPT)`. Note, the global status does NOT
  affect re-encryption; rather the method used during encryption. For instance,
  if the default is set to level-one (i.e., `ProxyReEncryption.REENCRYPT.NO`) but a message
  was encrypted allowing for re-encryption, then the message will ALWAYS allow
  re-encryption.

  ### Addenda

  Three addenda are included in this implementation: two-party re-encryption,
  sign/verify, and encrypt-sign/decrypt-verify. A discussion of each
  follows.

  #### Two-Party Re-encryption

  Two-party re-encryption extends the re-encryption process whereby the
  intermediary (or proxy) re-encrypts the message in such a way that it (1)
  cannot retrieve the original message and (2) does not learn any re-encryption
  key. The delegatee, following re-encryption principles, decrypts the
  intermediary's re-encrypted message using a re-encryption key that does not
  allow for decryption of the original message.

  #### Sign/Verify

  Sign/verify is a simple extension whereby the message is encrypted using the
  signer's private key. Therefore, anyone with access to the signer's public
  key can decrypt the message, indicating it came from the signer.

  #### Encrypt-Sign/Decrypt-Verify

  Encrypt-sign encrypts the message using the sender's private key (i.e.,
  signs) then encrypts the results with the recipient's public key. The
  recipient decrypts the message using its private key then again with the
  sender's public key (i.e., verify). Thus, the sender can be assured that only
  the intended recipient can decrypt the message and the recipient is assured
  the message came from the sender.

  ### Sub-Class Implementation

  The encryption, decryption, re-encryption, two-party re-encryption,
  sign/verify, encrypt-sign/decrypt-verify, and converter components are to be
  implemented by sub-classes (e.g., `AFGH`).

- - ### Nested Class Summary

    Nested Classes

    | Modifier and Type | Class and Description |
    | --- | --- |
    | `static class` | `ProxyReEncryption.ENCRYPTION_MODE` Encryption mode options: Encryption mode refers to how the encryption keys will be treated. |
    | `static class` | `ProxyReEncryption.MESSAGE_TYPE` Message `byte[]` type. |
    | `static class` | `ProxyReEncryption.REENCRYPT` Re-encryption type. |
    | `static class` | `ProxyReEncryption.SCALE` Scale input type. |
    | `static class` | `ProxyReEncryption.STORAGE` Storage volatility options: Storage volatility refers how the generated keys will be stored and read. |
  - ### Field Summary

    Fields

    | Modifier and Type | Field and Description |
    | --- | --- |
    | `protected java.io.File` | `curveFile` Curve `File` - if nonvolatile. |
    | `protected it.unisa.dia.gas.jpbc.PairingParameters` | `curveParams` Curve `PairingParameters`. |
    | `protected java.lang.String` | `curveProperties` Curve properties - if volatile. |
    | `protected static ProxyReEncryption.ENCRYPTION_MODE` | `DEFAULT_ENCRYPTION_MODE` Default `ProxyReEncryption.ENCRYPTION_MODE`. |
    | `protected ProxyReEncryption.REENCRYPT` | `DEFAULT_REENCRYPT` Default `ProxyReEncryption.REENCRYPT`. |
    | `protected it.unisa.dia.gas.jpbc.Pairing` | `e` `Pairing` `e`. |
    | `protected ProxyReEncryption.ENCRYPTION_MODE` | `encMode` `ProxyReEncryption.ENCRYPTION_MODE` parameter. |
    | `static java.util.HashMap<java.lang.Integer,ProxyReEncryption.ENCRYPTION_MODE>` | `encModeHM` Maps `ProxyReEncryption.ENCRYPTION_MODE.code` to `ProxyReEncryption.ENCRYPTION_MODE`. |
    | `protected it.unisa.dia.gas.jpbc.Element` | `g1` `Element` `g1` generator. |
    | `protected it.unisa.dia.gas.jpbc.Field` | `G1` `Field` `G1`. |
    | `protected it.unisa.dia.gas.jpbc.ElementPowPreProcessing` | `g1p` Generator 1 power pre-processing (`ElementPowPreProcessing`). |
    | `protected it.unisa.dia.gas.jpbc.Element` | `g2` `Element` `g2` generator. |
    | `protected it.unisa.dia.gas.jpbc.Field` | `G2` `Field` `G2`. |
    | `protected it.unisa.dia.gas.jpbc.ElementPowPreProcessing` | `g2p` Generator 2 power pre-processing (`ElementPowPreProcessing`). |
    | `protected it.unisa.dia.gas.jpbc.Field` | `GT` `Field` `GT`. |
    | `static edu.ecu.hsim.ray.logger.Logger` | `log` Global logging. |
    | `protected java.math.BigInteger` | `order` Order. |
    | `protected ProxyReEncryption.STORAGE` | `storage` `ProxyReEncryption.STORAGE` parameter. |
    | `protected it.unisa.dia.gas.jpbc.Element` | `Z` `Element` `Z = e(g1, g2)`. |
    | `protected it.unisa.dia.gas.jpbc.ElementPowPreProcessing` | `Zp` `Z` power pre-processing (`ElementPowPreProcessing`). |
    | `protected it.unisa.dia.gas.jpbc.Field` | `Zr` `Field` `Zr`. |
  - ### Constructor Summary

    Constructors

    | Modifier | Constructor and Description |
    | --- | --- |
    | `protected` | `ProxyReEncryption()` Constructor. |
  - ### Method Summary

    All Methods Instance Methods Abstract Methods Concrete Methods

    | Modifier and Type | Method and Description |
    | --- | --- |
    | `abstract Message` | `decrypt(it.unisa.dia.gas.jpbc.Element x, Cipher cipher)` Decrypts the `Cipher` using the provided ephemeral key `x`. |
    | `abstract Message` | `decrypt(it.unisa.dia.gas.jpbc.Element x, Message message)` Decrypts the `Message` using the provided ephemeral key `x`. |
    | `abstract Message` | `decrypt(Keys keys, Cipher cipher)` Decrypts the `Cipher` using the provided private key. |
    | `abstract Message` | `decrypt(Keys keys, Message message)` Decrypts the `Message` using the provided private key. |
    | `abstract Message` | `decryptDelegatee(Keys key, Cipher cipher)` Decrypts the delegatee layer of a two-party re-encryption `Cipher` using the provided private key. |
    | `abstract Cipher` | `decryptIntermediate(Keys key, Cipher cipher)` Decrypts the intermediate layer of a two-party re-encryption `Cipher` using the provided private key. |
    | `abstract Cipher` | `decryptToCipher(it.unisa.dia.gas.jpbc.Element x, Cipher cipher)` Decrypts the `Cipher` to a `Cipher` using the provided ephemeral key `x`. |
    | `abstract Cipher` | `decryptToCipher(Keys keys, Cipher cipher)` Decrypts the `Cipher` to a `Cipher` using the provided private key. |
    | `abstract Cipher` | `encrypt(it.unisa.dia.gas.jpbc.Element x, byte[] message, ProxyReEncryption.MESSAGE_TYPE type)` Encrypts the `message` using the ephemeral key `x`. |
    | `abstract Cipher` | `encrypt(it.unisa.dia.gas.jpbc.Element x, Cipher cipher)` Encrypts the `Cipher` using the ephemeral key `x`. |
    | `abstract Cipher` | `encrypt(it.unisa.dia.gas.jpbc.Element x, Message message)` Encrypts the `Message` using the ephemeral key `x`. |
    | `abstract Cipher` | `encrypt(it.unisa.dia.gas.jpbc.Element x, java.lang.String message)` Encrypts the `message` using the ephemeral key `x`. |
    | `abstract Cipher` | `encrypt(Keys keys, byte[] message, ProxyReEncryption.MESSAGE_TYPE type)` Encrypts the `message` using the public key. |
    | `abstract Cipher` | `encrypt(Keys keys, byte[] message, ProxyReEncryption.MESSAGE_TYPE type, ProxyReEncryption.REENCRYPT reencrypt)` Encrypts the `message` using the public key and `reencrypt` parameter. |
    | `abstract Cipher` | `encrypt(Keys keys, Cipher cipher)` Encrypts the `Cipher` using the public key. |
    | `abstract Cipher` | `encrypt(Keys keys, Cipher cipher, ProxyReEncryption.REENCRYPT reencrypt)` Encrypts the `Cipher` using the public key and `reencrypt` parameter. |
    | `abstract Cipher` | `encrypt(Keys keys, Message message)` Encrypts the `Message` using the public key. |
    | `abstract Cipher` | `encrypt(Keys keys, Message message, ProxyReEncryption.REENCRYPT reencrypt)` Encrypts the `Message` using the public key and `reencrypt` parameter. |
    | `abstract Cipher` | `encrypt(Keys keys, java.lang.String message)` Encrypts the `message` using the public key. |
    | `abstract Cipher` | `encrypt(Keys keys, java.lang.String message, ProxyReEncryption.REENCRYPT reencrypt)` Encrypts the `message` using the public key and `reencrypt` parameter. |
    | `java.io.File` | `getCurveFile()` Returns the curve file if `storage` == `ProxyReEncryption.STORAGE.NONVOLATILE`. |
    | `it.unisa.dia.gas.jpbc.PairingParameters` | `getCurveParams()` Returns the curve parameters. |
    | `java.lang.String` | `getCurveProperties()` Returns the curve properties if `storage` == `ProxyReEncryption.STORAGE.VOLATILE`. |
    | `ProxyReEncryption.REENCRYPT` | `getDefaultReEncrypt()` Returns the default re-encryption parameter. |
    | `protected it.unisa.dia.gas.jpbc.Pairing` | `getE()` Returns `e`. |
    | `ProxyReEncryption.ENCRYPTION_MODE` | `getEncryptionMode()` Returns the `ProxyReEncryption.ENCRYPTION_MODE`. |
    | `it.unisa.dia.gas.jpbc.Element` | `getG1Gen()` Returns the generator `g1` for `G1`. |
    | `protected it.unisa.dia.gas.jpbc.ElementPowPreProcessing` | `getG1GenP()` Returns pow pre processing element `g1p` for `G1`. |
    | `it.unisa.dia.gas.jpbc.Element` | `getG2Gen()` Returns the generator `g2` for `G2`. |
    | `protected it.unisa.dia.gas.jpbc.ElementPowPreProcessing` | `getG2GenP()` Returns pow pre processing element `g2p` for `G2`. |
    | `protected it.unisa.dia.gas.jpbc.Field` | `getGT()` Returns `GT`. |
    | `protected java.lang.String` | `getInfFlag(it.unisa.dia.gas.plaf.jpbc.field.curve.CurveElement ce)` Returns `infFlag` for the `CurveElement` `ce`. |
    | `edu.ecu.hsim.ray.logger.Logger.Level` | `getLogLevel()` Returns the log level. |
    | `java.math.BigInteger` | `getOrder()` Returns the order. |
    | `it.unisa.dia.gas.jpbc.ElementPowPreProcessing` | `getZP()` Returns `Zp`. |
    | `protected it.unisa.dia.gas.jpbc.Field` | `getZr()` Returns `Zr`. |
    | `it.unisa.dia.gas.jpbc.Element` | `newEphemeralKeyX()` Returns a new, random ephemeral key `x` `Element`. |
    | `it.unisa.dia.gas.jpbc.Element` | `newEphemeralKeyX(byte[] bytes)` Returns a new ephemeral key `Element` derived from `G` with its contents set to `bytes`. |
    | `it.unisa.dia.gas.plaf.jpbc.field.curve.CurveElement` | `newG1CurveElement()` Returns a new `CurveElement` derived from `G1`. |
    | `it.unisa.dia.gas.plaf.jpbc.field.curve.CurveElement` | `newG1CurveElement(byte[] bytes)` Returns a new `CurveElement` derived from `G1` with its contents set to `bytes`. |
    | `it.unisa.dia.gas.plaf.jpbc.field.curve.CurveElement` | `newG1CurveElement(byte[] bytes, int offset)` Returns a new `CurveElement` derived from `G1` with its contents set to `bytes` beginning at `offset`. |
    | `it.unisa.dia.gas.plaf.jpbc.field.curve.CurveElement` | `newG2CurveElement()` Returns a new `CurveElement` derived from `G2`. |
    | `it.unisa.dia.gas.plaf.jpbc.field.curve.CurveElement` | `newG2CurveElement(byte[] bytes)` Returns a new `CurveElement` derived from `G2` with its contents set to `bytes`. |
    | `it.unisa.dia.gas.plaf.jpbc.field.curve.CurveElement` | `newG2CurveElement(byte[] bytes, int offset)` Returns a new `CurveElement` derived from `G2` with its contents set to `bytes` beginning at `offset`. |
    | `it.unisa.dia.gas.plaf.jpbc.field.gt.GTFiniteElement` | `newGTElement()` Returns a new `GTFiniteElement` derived from `GT`. |
    | `it.unisa.dia.gas.plaf.jpbc.field.gt.GTFiniteElement` | `newGTElement(byte[] bytes)` Returns a new `GTFiniteElement` derived from `GT` with its contents set to `bytes`. |
    | `it.unisa.dia.gas.plaf.jpbc.field.gt.GTFiniteElement` | `newGTElement(byte[] bytes, int offset)` Returns a new `GTFiniteElement` derived from `GT` with its contents set to `bytes` beginning at `offset`. |
    | `Keys` | `newKeys()` Constructs a new `ProxyReEncryption.STORAGE.VOLATILE` set of `Keys`. |
    | `Keys` | `newKeys(byte[] publicParams, byte[] secretParams)` Constructs a new `ProxyReEncryption.STORAGE.VOLATILE` set of `Keys`. |
    | `Keys` | `newKeys(java.lang.String paramsFile)` Constructs a new `ProxyReEncryption.STORAGE.NONVOLATILE` set of `Keys`. |
    | `Keys` | `newKeys(java.lang.String publicParams, java.lang.String secretParams)` Constructs a new `ProxyReEncryption.STORAGE.VOLATILE` set of `Keys`. |
    | `ReKeys` | `newReKeys(Keys delegator, Keys delegatee)` Constructs a new `ProxyReEncryption.STORAGE.VOLATILE` re-encryption key. |
    | `ReKeys` | `newReKeys(Keys delegator, Keys intermediary, Keys delegatee)` Constructs a new two-party `ProxyReEncryption.STORAGE.VOLATILE` re-encryption key. |
    | `ReKeys` | `newReKeys(Keys delegator, Keys intermediary, Keys delegatee, java.lang.String params)` Constructs a new two-party `ProxyReEncryption.STORAGE.NONVOLATILE` re-encryption key. |
    | `ReKeys` | `newReKeys(Keys delegator, Keys delegatee, java.lang.String params)` Constructs a new `ProxyReEncryption.STORAGE.NONVOLATILE` re-encryption key. |
    | `ReKeys` | `newReKeys(java.lang.String params, ProxyReEncryption.STORAGE storage)` Constructs a new `storage` re-encryption key from an existing source - `params`. |
    | `it.unisa.dia.gas.plaf.jpbc.field.z.ZrElement` | `newZrElement()` Returns a new `ZrElement` derived from `Zr`. |
    | `it.unisa.dia.gas.plaf.jpbc.field.z.ZrElement` | `newZrElement(byte[] bytes)` Returns a new `ZrElement` derived from `Zr` with its contents set to `bytes`. |
    | `it.unisa.dia.gas.plaf.jpbc.field.z.ZrElement` | `newZrElement(byte[] bytes, int offset)` Returns a new `ZrElement` derived from `Zr` with its contents set to `bytes` beginning at `offset`. |
    | `Cipher` | `readCipher(java.lang.String file)` Reads a `Cipher` from a file. |
    | `Message` | `readMessage(java.lang.String file)` Reads a `Message` from a file. |
    | `abstract Cipher` | `reencrypt(Keys rekey, Cipher cipher)` Re-encrypts a `Cipher` using the re-encryption `Keys`. |
    | `abstract Cipher` | `reencrypt(Keys key, Keys rekey, Cipher cipher)` Re-encrypts a two-party `Cipher` using the re-encryption `Keys`. |
    | `abstract void` | `scale(Cipher cipher, it.unisa.dia.gas.jpbc.Element from, it.unisa.dia.gas.jpbc.Element to, ProxyReEncryption.SCALE scaleType)` Transforms the encryption key of the `Cipher` from `from` to `to` by way of simple scaling. |
    | `abstract void` | `scale(Cipher cipher, it.unisa.dia.gas.jpbc.Element scalar, ProxyReEncryption.SCALE scaleType)` Transforms the encryption key of the `Cipher` from `from` to `to` by way of simple scaling. |
    | `abstract void` | `scale(Message message, it.unisa.dia.gas.jpbc.Element from, it.unisa.dia.gas.jpbc.Element to, ProxyReEncryption.SCALE scaleType)` Transforms the encryption key of the `Message` from `from` to `to` by way of simple scaling. |
    | `abstract void` | `scale(Message message, it.unisa.dia.gas.jpbc.Element scalar, ProxyReEncryption.SCALE scaleType)` Transforms the encryption key of the `Message` from `from` to `to` by way of simple scaling. |
    | `void` | `setDefaultReEncrypt(ProxyReEncryption.REENCRYPT reencrypt)` Sets the default re-encryption parameter. |
    | `void` | `setEncryptionMode(ProxyReEncryption.ENCRYPTION_MODE encMode)` Set the `ProxyReEncryption.ENCRYPTION_MODE`. |
    | `protected void` | `setInfFlagToZero(it.unisa.dia.gas.plaf.jpbc.field.curve.CurveElement ce)` Sets `infFlag` to zero. |
    | `void` | `setLogLevel(edu.ecu.hsim.ray.logger.Logger.Level logLevel)` Sets the log level. |
    | `abstract Cipher` | `sign(Keys keys, byte[] message, ProxyReEncryption.MESSAGE_TYPE type)` Signs the `message` using an ephemeral key `x`, returning a `Cipher` that is verified by its hash and a private key-based decryption parameter (all contained within the `Cipher`). |
    | `abstract Cipher` | `sign(Keys keys, Cipher cipher)` Signs the `Cipher` using an ephemeral key `x`, returning a `Cipher` that is verified by its hash and a private key-based decryption parameter (all contained within the `Cipher`). |
    | `abstract Cipher` | `sign(Keys sign, Keys encrypt, byte[] message, ProxyReEncryption.MESSAGE_TYPE type)` Encrypt-signs the `message` using an ephemeral key `x`, returning a `Cipher` that is decrypt-verified by its hash and a decryption parameter constructed using the signer's private key and recipients public key (all contained within the `Cipher`). |
    | `abstract Cipher` | `sign(Keys sign, Keys encrypt, Cipher cipher)` Encrypt-signs the `Cipher` using an ephemeral key `x`, returning a `Cipher` that is decrypt-verified by its hash and a decryption parameter constructed using the signer's private key and recipients public key (all contained within the `Cipher`). |
    | `abstract Cipher` | `sign(Keys sign, Keys encrypt, Message message)` Encrypt-signs the `Message` using an ephemeral key `x`, returning a `Cipher` that is decrypt-verified by its hash and a decryption parameter constructed using the signer's private key and recipients public key (all contained within the `Cipher`). |
    | `abstract Cipher` | `sign(Keys sign, Keys encrypt, java.lang.String message)` Encrypt-signs the `message` using an ephemeral key `x`, returning a `Cipher` that is decrypt-verified by its hash and a decryption parameter constructed using the signer's private key and recipients public key (all contained within the `Cipher`). |
    | `abstract Cipher` | `sign(Keys keys, Message message)` Signs the `Message` using an ephemeral key `x`, returning a `Cipher` that is verified by its hash and a private key-based decryption parameter (all contained within the `Cipher`). |
    | `abstract Cipher` | `sign(Keys keys, java.lang.String message)` Signs the `message` using an ephemeral key `x`, returning a `Cipher` that is verified by its hash and a private key-based decryption parameter (all contained within the `Cipher`). |
    | `abstract Cipher` | `toCipher(byte[] bytes)` Converts a `byte[]` to a `Cipher`. |
    | `abstract Message` | `toMessage(byte[] message, int bytePadding)` Converts a `byte[]` to a `Message` with padding. |
    | `abstract Message` | `toMessage(byte[] message, ProxyReEncryption.MESSAGE_TYPE type)` Converts a `byte[]` to a `Message`. |
    | `abstract Message` | `toMessage(java.lang.String message)` Converts a `String` to a `Message`. |
    | `abstract boolean` | `verify(Keys keys, Cipher cipher)` Verifies a signed `Cipher` using the signer's private key, a message hash, and a decryption parameter generated using the signer's private key and the message hash. |
    | `abstract boolean` | `verify(Keys keys, Cipher cipher, byte[] expected)` Verifies a signed `Cipher` using the signer's private key, a message hash, and a decryption parameter generated using the signer's private key and the message hash. |
    | `abstract boolean` | `verify(Keys keys, Cipher cipher, Message expected)` Verifies a signed `Cipher` using the signer's private key, a message hash, and a decryption parameter generated using the signer's private key and the message hash. |
    | `abstract boolean` | `verify(Keys keys, Cipher cipher, java.lang.String expected)` Verifies a signed `Cipher` using the signer's private key, a message hash, and a decryption parameter generated using the signer's private key and the message hash. |
    | `abstract boolean` | `verify(Keys verify, Keys decrypt, Cipher cipher)` Decrypt-verifies a signed `Cipher` using the signer's private key, a message hash, and a decryption parameter generated using the signer's private key and the message hash. |
    | `abstract boolean` | `verify(Keys verify, Keys decrypt, Cipher cipher, byte[] expected)` Decrypt-verifies a signed `Cipher` using the signer's private key, a message hash, and a decryption parameter generated using the signer's private key and the message hash. |
    | `abstract boolean` | `verify(Keys verify, Keys decrypt, Cipher cipher, Message expected)` Decrypt-verifies a signed `Cipher` using the signer's private key, a message hash, and a decryption parameter generated using the signer's private key and the message hash. |
    | `abstract boolean` | `verify(Keys verify, Keys decrypt, Cipher cipher, java.lang.String expected)` Decrypt-verifies a signed `Cipher` using the signer's private key, a message hash, and a decryption parameter generated using the signer's private key and the message hash. |
    | `abstract boolean` | `verify(Keys verify, Keys decrypt, Message message)` Decrypt-verifies a signed `Message` using the signer's private key, a message hash, and a decryption parameter generated using the signer's private key and the message hash. |
    | `abstract boolean` | `verify(Keys verify, Keys decrypt, Message message, byte[] expected)` Decrypt-verifies a signed `Message` using the signer's private key, a message hash, and a decryption parameter generated using the signer's private key and the message hash. |
    | `abstract boolean` | `verify(Keys verify, Keys decrypt, Message message, Message expected)` Decrypt-verifies a signed `Message` using the signer's private key, a message hash, and a decryption parameter generated using the signer's private key and the message hash. |
    | `abstract boolean` | `verify(Keys verify, Keys decrypt, Message message, java.lang.String expected)` Decrypt-verifies a signed `Message` using the signer's private key, a message hash, and a decryption parameter generated using the signer's private key and the message hash. |
    | `abstract boolean` | `verify(Keys keys, Message message)` Verifies a signed `Message` using the signer's private key, a message hash, and a decryption parameter generated using the signer's private key and the message hash. |
    | `abstract boolean` | `verify(Keys keys, Message message, byte[] expected)` Verifies a signed `Message` using the signer's private key, a message hash, and a decryption parameter generated using the signer's private key and the message hash. |
    | `abstract boolean` | `verify(Keys keys, Message message, Message expected)` Verifies a signed `Message` using the signer's private key, a message hash, and a decryption parameter generated using the signer's private key and the message hash. |
    | `abstract boolean` | `verify(Keys keys, Message message, java.lang.String expected)` Verifies a signed `Message` using the signer's private key, a message hash, and a decryption parameter generated using the signer's private key and the message hash. |
    | `abstract Message` | `verifyAndGet(Keys keys, Cipher cipher)` Verifies a signed `Cipher` using the signer's private key, a message hash, and a decryption parameter generated using the signer's private key and the message hash. |
    | `abstract Message` | `verifyAndGet(Keys verify, Keys decrypt, Cipher cipher)` Decrypt-verifies a signed `Cipher` using the signer's private key, a message hash, and a decryption parameter generated using the signer's private key and the message hash. |
    | `abstract Message` | `verifyAndGet(Keys verify, Keys decrypt, Message message)` Decrypt-verifies a signed `Message` using the signer's private key, a message hash, and a decryption parameter generated using the signer's private key and the message hash. |
    | `abstract Message` | `verifyAndGet(Keys keys, Message message)` Verifies a signed `Message` using the signer's private key, a message hash, and a decryption parameter generated using the signer's private key and the message hash. |

    - ### Methods inherited from class java.lang.Object

      `clone, equals, finalize, getClass, hashCode, notify, notifyAll, toString, wait, wait, wait`

- - ### Field Detail


    - #### storage

      ```
      protected ProxyReEncryption.STORAGE storage
      ```

      `ProxyReEncryption.STORAGE` parameter.


    - #### encModeHM

      ```
      public static final java.util.HashMap<java.lang.Integer,ProxyReEncryption.ENCRYPTION_MODE> encModeHM
      ```

      Maps `ProxyReEncryption.ENCRYPTION_MODE.code` to `ProxyReEncryption.ENCRYPTION_MODE`.


    - #### DEFAULT\_ENCRYPTION\_MODE

      ```
      protected static final ProxyReEncryption.ENCRYPTION_MODE DEFAULT_ENCRYPTION_MODE
      ```

      Default `ProxyReEncryption.ENCRYPTION_MODE`.


    - #### encMode

      ```
      protected ProxyReEncryption.ENCRYPTION_MODE encMode
      ```

      `ProxyReEncryption.ENCRYPTION_MODE` parameter.


    - #### log

      ```
      public static final edu.ecu.hsim.ray.logger.Logger log
      ```

      Global logging.


    - #### DEFAULT\_REENCRYPT

      ```
      protected ProxyReEncryption.REENCRYPT DEFAULT_REENCRYPT
      ```

      Default `ProxyReEncryption.REENCRYPT`.


    - #### e

      ```
      protected it.unisa.dia.gas.jpbc.Pairing e
      ```

      `Pairing` `e`.


    - #### G1

      ```
      protected it.unisa.dia.gas.jpbc.Field G1
      ```

      `Field` `G1`.


    - #### G2

      ```
      protected it.unisa.dia.gas.jpbc.Field G2
      ```

      `Field` `G2`.


    - #### GT

      ```
      protected it.unisa.dia.gas.jpbc.Field GT
      ```

      `Field` `GT`.


    - #### Zr

      ```
      protected it.unisa.dia.gas.jpbc.Field Zr
      ```

      `Field` `Zr`.


    - #### g1

      ```
      protected it.unisa.dia.gas.jpbc.Element g1
      ```

      `Element` `g1` generator.


    - #### g2

      ```
      protected it.unisa.dia.gas.jpbc.Element g2
      ```

      `Element` `g2` generator.


    - #### Z

      ```
      protected it.unisa.dia.gas.jpbc.Element Z
      ```

      `Element` `Z = e(g1, g2)`.


    - #### g1p

      ```
      protected it.unisa.dia.gas.jpbc.ElementPowPreProcessing g1p
      ```

      Generator 1 power pre-processing (`ElementPowPreProcessing`).


    - #### g2p

      ```
      protected it.unisa.dia.gas.jpbc.ElementPowPreProcessing g2p
      ```

      Generator 2 power pre-processing (`ElementPowPreProcessing`).


    - #### Zp

      ```
      protected it.unisa.dia.gas.jpbc.ElementPowPreProcessing Zp
      ```

      `Z` power pre-processing (`ElementPowPreProcessing`).


    - #### curveParams

      ```
      protected it.unisa.dia.gas.jpbc.PairingParameters curveParams
      ```

      Curve `PairingParameters`.


    - #### order

      ```
      protected java.math.BigInteger order
      ```

      Order.


    - #### curveFile

      ```
      protected java.io.File curveFile
      ```

      Curve `File` - if nonvolatile.


    - #### curveProperties

      ```
      protected java.lang.String curveProperties
      ```

      Curve properties - if volatile.
  - ### Constructor Detail


    - #### ProxyReEncryption

      ```
      protected ProxyReEncryption()
      ```

      Constructor.
  - ### Method Detail


    - #### newKeys

      ```
      public Keys newKeys()
      ```

      Constructs a new `ProxyReEncryption.STORAGE.VOLATILE` set of `Keys`.

      Returns:
      :   the new `Keys` object


    - #### newKeys

      ```
      public Keys newKeys(java.lang.String paramsFile)
      ```

      Constructs a new `ProxyReEncryption.STORAGE.NONVOLATILE` set of `Keys`.

      Parameters:
      :   `paramsFile` - parameters file name

      Returns:
      :   the new `Keys` object


    - #### newKeys

      ```
      public Keys newKeys(byte[] publicParams,
                          byte[] secretParams)
      ```

      Constructs a new `ProxyReEncryption.STORAGE.VOLATILE` set of `Keys`.

      Parameters:
      :   `publicParams` - public parameters in `byte[]` form
      :   `secretParams` - secret parameters in `byte[]` form

      Returns:
      :   the new `Keys` object


    - #### newKeys

      ```
      public Keys newKeys(java.lang.String publicParams,
                          java.lang.String secretParams)
      ```

      Constructs a new `ProxyReEncryption.STORAGE.VOLATILE` set of `Keys`.

      Parameters:
      :   `publicParams` - public parameters in `String` form
      :   `secretParams` - secret parameters in `String` form

      Returns:
      :   the new `Keys` object


    - #### newReKeys

      ```
      public ReKeys newReKeys(Keys delegator,
                              Keys delegatee)
      ```

      Constructs a new `ProxyReEncryption.STORAGE.VOLATILE` re-encryption key.

      Parameters:
      :   `delegator` - `Keys` delegator
      :   `delegatee` - `Keys` delegatee

      Returns:
      :   the new `ReKeys` object


    - #### newReKeys

      ```
      public ReKeys newReKeys(Keys delegator,
                              Keys intermediary,
                              Keys delegatee)
      ```

      Constructs a new two-party `ProxyReEncryption.STORAGE.VOLATILE` re-encryption key.

      Parameters:
      :   `delegator` - `Keys` delegator
      :   `intermediary` - `Keys` intermediary
      :   `delegatee` - `Keys` delegatee

      Returns:
      :   the new `ReKeys` object


    - #### newReKeys

      ```
      public ReKeys newReKeys(Keys delegator,
                              Keys delegatee,
                              java.lang.String params)
      ```

      Constructs a new `ProxyReEncryption.STORAGE.NONVOLATILE` re-encryption key.

      Parameters:
      :   `delegator` - `Keys` delegator
      :   `delegatee` - `Keys` delegatee
      :   `params` - parameters file

      Returns:
      :   the new `ReKeys` object


    - #### newReKeys

      ```
      public ReKeys newReKeys(Keys delegator,
                              Keys intermediary,
                              Keys delegatee,
                              java.lang.String params)
      ```

      Constructs a new two-party `ProxyReEncryption.STORAGE.NONVOLATILE` re-encryption key.

      Parameters:
      :   `delegator` - `Keys` delegator
      :   `intermediary` - `Keys` intermediary
      :   `delegatee` - `Keys` delegatee
      :   `params` - parameters file

      Returns:
      :   the new `ReKeys` object


    - #### newReKeys

      ```
      public ReKeys newReKeys(java.lang.String params,
                              ProxyReEncryption.STORAGE storage)
                       throws java.io.IOException
      ```

      Constructs a new `storage` re-encryption key from an existing
      source - `params`.

      Parameters:
      :   `params` - parameters
      :   `storage` - `ProxyReEncryption.STORAGE` option

      Returns:
      :   the new `ReKeys` object

      Throws:
      :   `java.io.IOException` - `IOException`


    - #### encrypt

      ```
      public abstract Cipher encrypt(Keys keys,
                                     java.lang.String message)
      ```

      Encrypts the `message` using the public key. Uses the
      `DEFAULT_REENCRYPT` parameter, which is initialized to
      `ProxyReEncryption.REENCRYPT.NO` and can be altered using
      `setDefaultReEncrypt(REENCRYPT)`. This issues new encryption
      parameters if `encMode` is `ProxyReEncryption.ENCRYPTION_MODE.DYNAMIC`.

      Parameters:
      :   `keys` - encryption `Keys`
      :   `message` - message to encrypt

      Returns:
      :   encrypted `Cipher`


    - #### encrypt

      ```
      public abstract Cipher encrypt(Keys keys,
                                     byte[] message,
                                     ProxyReEncryption.MESSAGE_TYPE type)
      ```

      Encrypts the `message` using the public key. Uses the
      `DEFAULT_REENCRYPT` parameter, which is initialized to
      `ProxyReEncryption.REENCRYPT.NO` and can be altered using
      `setDefaultReEncrypt(REENCRYPT)`. This issues new encryption
      parameters if `encMode` is `ProxyReEncryption.ENCRYPTION_MODE.DYNAMIC`.

      Parameters:
      :   `keys` - encryption `Keys`
      :   `message` - message to encrypt
      :   `type` - `ProxyReEncryption.MESSAGE_TYPE.FORMATTED` if the passed bytes are
          the result of a `Message.toBytes()` call, as this
          result is formatted; otherwise, use
          `ProxyReEncryption.MESSAGE_TYPE.UNFORMATTED`

      Returns:
      :   encrypted `Cipher`


    - #### encrypt

      ```
      public abstract Cipher encrypt(Keys keys,
                                     Message message)
      ```

      Encrypts the `Message` using the public key. Uses the
      `DEFAULT_REENCRYPT` parameter, which is initialized to
      `ProxyReEncryption.REENCRYPT.NO` and can be altered using
      `setDefaultReEncrypt(REENCRYPT)`. This issues new encryption
      parameters if `encMode` is `ProxyReEncryption.ENCRYPTION_MODE.DYNAMIC`.

      Parameters:
      :   `keys` - encryption `Keys`
      :   `message` - `Message` to encrypt

      Returns:
      :   encrypted `Cipher`


    - #### encrypt

      ```
      public abstract Cipher encrypt(Keys keys,
                                     Cipher cipher)
      ```

      Encrypts the `Cipher` using the public key. Uses the
      `DEFAULT_REENCRYPT` parameter, which is initialized to
      `ProxyReEncryption.REENCRYPT.NO` and can be altered using
      `setDefaultReEncrypt(REENCRYPT)`. This issues new encryption
      parameters if `encMode` is `ProxyReEncryption.ENCRYPTION_MODE.DYNAMIC`.

      Parameters:
      :   `keys` - encryption `Keys`
      :   `cipher` - `Cipher` to encrypt

      Returns:
      :   encrypted `Cipher`


    - #### encrypt

      ```
      public abstract Cipher encrypt(Keys keys,
                                     java.lang.String message,
                                     ProxyReEncryption.REENCRYPT reencrypt)
      ```

      Encrypts the `message` using the public key and `reencrypt`
      parameter. If `reencrypt` is `ProxyReEncryption.REENCRYPT.NO`, then first-level
      encryption is used (i.e., encryption that CANNOT be re-encrypted); else
      second-level (i.e., encryption that CAN be re-encrypted). This issues new
      encryption parameters if `encMode` is
      `ProxyReEncryption.ENCRYPTION_MODE.DYNAMIC`.

      Parameters:
      :   `keys` - encryption `Keys`
      :   `message` - message to encrypt
      :   `reencrypt` - `ProxyReEncryption.REENCRYPT` parameter

      Returns:
      :   encrypted `Cipher`


    - #### encrypt

      ```
      public abstract Cipher encrypt(Keys keys,
                                     byte[] message,
                                     ProxyReEncryption.MESSAGE_TYPE type,
                                     ProxyReEncryption.REENCRYPT reencrypt)
      ```

      Encrypts the `message` using the public key and `reencrypt`
      parameter. If `reencrypt` is `ProxyReEncryption.REENCRYPT.NO`, then first-level
      encryption is used (i.e., encryption that CANNOT be re-encrypted); else
      second-level (i.e., encryption that CAN be re-encrypted). This issues new
      encryption parameters if `encMode` is
      `ProxyReEncryption.ENCRYPTION_MODE.DYNAMIC`.

      Parameters:
      :   `keys` - encryption `Keys`
      :   `message` - message to encrypt
      :   `type` - `ProxyReEncryption.MESSAGE_TYPE.FORMATTED` if the passed bytes are
          the result of a `Message.toBytes()` call, as this
          result is formatted; otherwise, use
          `ProxyReEncryption.MESSAGE_TYPE.UNFORMATTED`
      :   `reencrypt` - `ProxyReEncryption.REENCRYPT` parameter

      Returns:
      :   encrypted `Cipher`


    - #### encrypt

      ```
      public abstract Cipher encrypt(Keys keys,
                                     Message message,
                                     ProxyReEncryption.REENCRYPT reencrypt)
      ```

      Encrypts the `Message` using the public key and `reencrypt`
      parameter. If `reencrypt` is `ProxyReEncryption.REENCRYPT.NO`, then first-level
      encryption is used (i.e., encryption that CANNOT be re-encrypted); else
      second-level (i.e., encryption that CAN be re-encrypted). This issues new
      encryption parameters if `encMode` is
      `ProxyReEncryption.ENCRYPTION_MODE.DYNAMIC`.

      Parameters:
      :   `keys` - encryption `Keys`
      :   `message` - `Message` to encrypt
      :   `reencrypt` - `ProxyReEncryption.REENCRYPT` parameter

      Returns:
      :   encrypted `Cipher`


    - #### encrypt

      ```
      public abstract Cipher encrypt(Keys keys,
                                     Cipher cipher,
                                     ProxyReEncryption.REENCRYPT reencrypt)
      ```

      Encrypts the `Cipher` using the public key and `reencrypt`
      parameter. If `reencrypt` is `ProxyReEncryption.REENCRYPT.NO`, then first-level
      encryption is used (i.e., encryption that CANNOT be re-encrypted); else
      second-level (i.e., encryption that CAN be re-encrypted). This issues new
      encryption parameters if `encMode` is
      `ProxyReEncryption.ENCRYPTION_MODE.DYNAMIC`.

      Parameters:
      :   `keys` - encryption `Keys`
      :   `cipher` - `Cipher` to encrypt
      :   `reencrypt` - `ProxyReEncryption.REENCRYPT` parameter

      Returns:
      :   encrypted `Cipher`


    - #### encrypt

      ```
      public abstract Cipher encrypt(it.unisa.dia.gas.jpbc.Element x,
                                     java.lang.String message)
      ```

      Encrypts the `message` using the ephemeral key `x`.

      Parameters:
      :   `x` - ephemeral key `Element`
      :   `message` - message to encrypt

      Returns:
      :   encrypted `Cipher`


    - #### encrypt

      ```
      public abstract Cipher encrypt(it.unisa.dia.gas.jpbc.Element x,
                                     byte[] message,
                                     ProxyReEncryption.MESSAGE_TYPE type)
      ```

      Encrypts the `message` using the ephemeral key `x`.

      Parameters:
      :   `x` - ephemeral key `Element`
      :   `message` - message to encrypt
      :   `type` - `ProxyReEncryption.MESSAGE_TYPE.FORMATTED` if the passed bytes are
          the result of a `Message.toBytes()` call, as this
          result is formatted; otherwise, use
          `ProxyReEncryption.MESSAGE_TYPE.UNFORMATTED`

      Returns:
      :   encrypted `Cipher`


    - #### encrypt

      ```
      public abstract Cipher encrypt(it.unisa.dia.gas.jpbc.Element x,
                                     Message message)
      ```

      Encrypts the `Message` using the ephemeral key `x`.

      Parameters:
      :   `x` - ephemeral key `Element`
      :   `message` - `Message` to encrypt

      Returns:
      :   encrypted `Cipher`


    - #### encrypt

      ```
      public abstract Cipher encrypt(it.unisa.dia.gas.jpbc.Element x,
                                     Cipher cipher)
      ```

      Encrypts the `Cipher` using the ephemeral key `x`.

      Parameters:
      :   `x` - ephemeral key `Element`
      :   `cipher` - `Cipher` to encrypt

      Returns:
      :   encrypted `Cipher`


    - #### decrypt

      ```
      public abstract Message decrypt(Keys keys,
                                      Cipher cipher)
      ```

      Decrypts the `Cipher` using the provided private key.

      Parameters:
      :   `keys` - decryption `Keys`
      :   `cipher` - `Cipher` to decrypt

      Returns:
      :   decrypted `Cipher` as a `Message`


    - #### decrypt

      ```
      public abstract Message decrypt(Keys keys,
                                      Message message)
      ```

      Decrypts the `Message` using the provided private key.

      Parameters:
      :   `keys` - decryption `Keys`
      :   `message` - `Message` to decrypt

      Returns:
      :   decrypted `Cipher` as a `Message`


    - #### decryptToCipher

      ```
      public abstract Cipher decryptToCipher(Keys keys,
                                             Cipher cipher)
      ```

      Decrypts the `Cipher` to a `Cipher` using the provided
      private key.

      Parameters:
      :   `keys` - decryption `Keys`
      :   `cipher` - `Cipher` to decrypt

      Returns:
      :   decrypted `Cipher` as a `Cipher`


    - #### decryptIntermediate

      ```
      public abstract Cipher decryptIntermediate(Keys key,
                                                 Cipher cipher)
      ```

      Decrypts the intermediate layer of a two-party re-encryption
      `Cipher` using the provided private key.

      Parameters:
      :   `key` - decryption `Keys`
      :   `cipher` - `Cipher` to decrypt at the middle layer

      Returns:
      :   decrypted `Cipher` as a `Cipher`


    - #### decryptDelegatee

      ```
      public abstract Message decryptDelegatee(Keys key,
                                               Cipher cipher)
      ```

      Decrypts the delegatee layer of a two-party re-encryption `Cipher`
      using the provided private key.

      Parameters:
      :   `key` - decryption `Keys`
      :   `cipher` - `Cipher` to decrypt at the final layer

      Returns:
      :   decrypted `Cipher` as a `Message`


    - #### decrypt

      ```
      public abstract Message decrypt(it.unisa.dia.gas.jpbc.Element x,
                                      Cipher cipher)
      ```

      Decrypts the `Cipher` using the provided ephemeral key `x`.

      Parameters:
      :   `x` - ephemeral key `Element`
      :   `cipher` - `Cipher` to decrypt

      Returns:
      :   decrypted `Cipher` as a `Message`


    - #### decrypt

      ```
      public abstract Message decrypt(it.unisa.dia.gas.jpbc.Element x,
                                      Message message)
      ```

      Decrypts the `Message` using the provided ephemeral key `x`.

      Parameters:
      :   `x` - ephemeral key `Element`
      :   `message` - `Message` to decrypt

      Returns:
      :   decrypted `Cipher` as a `Message`


    - #### decryptToCipher

      ```
      public abstract Cipher decryptToCipher(it.unisa.dia.gas.jpbc.Element x,
                                             Cipher cipher)
      ```

      Decrypts the `Cipher` to a `Cipher` using the provided
      ephemeral key `x`.

      Parameters:
      :   `x` - ephemeral key `Element`
      :   `cipher` - `Cipher` to decrypt

      Returns:
      :   decrypted `Cipher` as a `Cipher`


    - #### reencrypt

      ```
      public abstract Cipher reencrypt(Keys rekey,
                                       Cipher cipher)
      ```

      Re-encrypts a `Cipher` using the re-encryption `Keys`.

      Parameters:
      :   `rekey` - re-encryption `Keys`
      :   `cipher` - `Cipher` to re-encrypt

      Returns:
      :   re-encrypted `Cipher` - does not overwrite `cipher`


    - #### reencrypt

      ```
      public abstract Cipher reencrypt(Keys key,
                                       Keys rekey,
                                       Cipher cipher)
      ```

      Re-encrypts a two-party `Cipher` using the re-encryption
      `Keys`.

      Parameters:
      :   `key` - from `Keys`
      :   `rekey` - re-encryption `Keys`
      :   `cipher` - `Cipher` to re-encrypt

      Returns:
      :   re-encrypted `Cipher` - does not overwrite `cipher`


    - #### sign

      ```
      public abstract Cipher sign(Keys keys,
                                  java.lang.String message)
      ```

      Signs the `message` using an ephemeral key `x`, returning a
      `Cipher` that is verified by its hash and a private key-based
      decryption parameter (all contained within the `Cipher`).

      Parameters:
      :   `keys` - `Keys` for signing
      :   `message` - message to sign

      Returns:
      :   the signed `message` as a `Cipher`


    - #### sign

      ```
      public abstract Cipher sign(Keys keys,
                                  byte[] message,
                                  ProxyReEncryption.MESSAGE_TYPE type)
      ```

      Signs the `message` using an ephemeral key `x`, returning a
      `Cipher` that is verified by its hash and a private key-based
      decryption parameter (all contained within the `Cipher`).

      Parameters:
      :   `keys` - `Keys` for signing
      :   `message` - message to sign
      :   `type` - `ProxyReEncryption.MESSAGE_TYPE.FORMATTED` if the passed bytes are
          the result of a `Message.toBytes()` call, as this
          result is formatted; otherwise, use
          `ProxyReEncryption.MESSAGE_TYPE.UNFORMATTED`

      Returns:
      :   the signed `message` as a `Cipher`


    - #### sign

      ```
      public abstract Cipher sign(Keys keys,
                                  Message message)
      ```

      Signs the `Message` using an ephemeral key `x`, returning a
      `Cipher` that is verified by its hash and a private key-based
      decryption parameter (all contained within the `Cipher`).

      Parameters:
      :   `keys` - `Keys` for signing
      :   `message` - `Message` to sign

      Returns:
      :   the signed `Message` as a `Cipher`


    - #### sign

      ```
      public abstract Cipher sign(Keys keys,
                                  Cipher cipher)
      ```

      Signs the `Cipher` using an ephemeral key `x`, returning a
      `Cipher` that is verified by its hash and a private key-based
      decryption parameter (all contained within the `Cipher`).

      Parameters:
      :   `keys` - `Keys` for signing
      :   `cipher` - `Cipher` to sign

      Returns:
      :   the signed `Cipher` as a `Cipher`


    - #### verifyAndGet

      ```
      public abstract Message verifyAndGet(Keys keys,
                                           Message message)
      ```

      Verifies a signed `Message` using the signer's private key, a
      message hash, and a decryption parameter generated using the signer's
      private key and the message hash. This is an intermediate verification
      step, as the message contents must be the result of a verified
      `Cipher`. The contents of the message are therefore, if compounded
      signatures, a `Cipher`. This returns a verified message.

      Parameters:
      :   `keys` - `Keys` for verifying
      :   `message` - `Message` to verify

      Returns:
      :   the verified `Message` as a `Message`


    - #### verify

      ```
      public abstract boolean verify(Keys keys,
                                     Message message)
      ```

      Verifies a signed `Message` using the signer's private key, a
      message hash, and a decryption parameter generated using the signer's
      private key and the message hash. This is an intermediate verification
      step, as the message contents must be the result of a verified
      `Cipher`. The contents of the message are therefore, if compounded
      signatures, a `Cipher`. This returns `true` if verified,
      `false` otherwise.

      Parameters:
      :   `keys` - `Keys` for verifying
      :   `message` - `Message` to verify

      Returns:
      :   `true` if verified, `false` otherwise


    - #### verify

      ```
      public abstract boolean verify(Keys keys,
                                     Message message,
                                     Message expected)
      ```

      Verifies a signed `Message` using the signer's private key, a
      message hash, and a decryption parameter generated using the signer's
      private key and the message hash. This is an intermediate verification
      step, as the message contents must be the result of a verified
      `Cipher`. The contents of the message are therefore, if compounded
      signatures, a `Cipher`. This returns `true` if the contents
      of the signed message match `expected`, `false` otherwise.

      Parameters:
      :   `keys` - `Keys` for verifying
      :   `message` - `Message` to verify
      :   `expected` - the expected result

      Returns:
      :   `true` if verified, `false` otherwise


    - #### verify

      ```
      public abstract boolean verify(Keys keys,
                                     Message message,
                                     java.lang.String expected)
      ```

      Verifies a signed `Message` using the signer's private key, a
      message hash, and a decryption parameter generated using the signer's
      private key and the message hash. This is an intermediate verification
      step, as the message contents must be the result of a verified
      `Cipher`. The contents of the message are therefore, if compounded
      signatures, a `Cipher`. This returns `true` if the contents
      of the signed message match `expected`, `false` otherwise.

      Parameters:
      :   `keys` - `Keys` for verifying
      :   `message` - `Message` to verify
      :   `expected` - the expected result

      Returns:
      :   `true` if verified, `false` otherwise


    - #### verify

      ```
      public abstract boolean verify(Keys keys,
                                     Message message,
                                     byte[] expected)
      ```

      Verifies a signed `Message` using the signer's private key, a
      message hash, and a decryption parameter generated using the signer's
      private key and the message hash. This is an intermediate verification
      step, as the message contents must be the result of a verified
      `Cipher`. The contents of the message are therefore, if compounded
      signatures, a `Cipher`. This returns `true` if the contents
      of the signed message match `expected`, `false` otherwise.

      Parameters:
      :   `keys` - `Keys` for verifying
      :   `message` - `Message` to verify
      :   `expected` - the expected result

      Returns:
      :   `true` if verified, `false` otherwise


    - #### verifyAndGet

      ```
      public abstract Message verifyAndGet(Keys keys,
                                           Cipher cipher)
      ```

      Verifies a signed `Cipher` using the signer's private key, a
      message hash, and a decryption parameter generated using the signer's
      private key and the message hash. This returns a verified message.

      Parameters:
      :   `keys` - `Keys` for verifying
      :   `cipher` - `Cipher` to verify

      Returns:
      :   the verified `Cipher` as a `Message`


    - #### verify

      ```
      public abstract boolean verify(Keys keys,
                                     Cipher cipher)
      ```

      Verifies a signed `Cipher` using the signer's private key, a
      message hash, and a decryption parameter generated using the signer's
      private key and the message hash. This returns `true` if verified,
      `false` otherwise.

      Parameters:
      :   `keys` - `Keys` for verifying
      :   `cipher` - `Cipher` to verify

      Returns:
      :   `true` if verified, `false` otherwise


    - #### verify

      ```
      public abstract boolean verify(Keys keys,
                                     Cipher cipher,
                                     Message expected)
      ```

      Verifies a signed `Cipher` using the signer's private key, a
      message hash, and a decryption parameter generated using the signer's
      private key and the message hash. This returns `true` if the
      contents of the signed message match `expected`, `false`
      otherwise.

      Parameters:
      :   `keys` - `Keys` for verifying
      :   `cipher` - `Cipher` to verify
      :   `expected` - the expected result

      Returns:
      :   `true` if verified, `false` otherwise


    - #### verify

      ```
      public abstract boolean verify(Keys keys,
                                     Cipher cipher,
                                     java.lang.String expected)
      ```

      Verifies a signed `Cipher` using the signer's private key, a
      message hash, and a decryption parameter generated using the signer's
      private key and the message hash. This returns `true` if the
      contents of the signed message match `expected`, `false`
      otherwise.

      Parameters:
      :   `keys` - `Keys` for verifying
      :   `cipher` - `Cipher` to verify
      :   `expected` - the expected result

      Returns:
      :   `true` if verified, `false` otherwise


    - #### verify

      ```
      public abstract boolean verify(Keys keys,
                                     Cipher cipher,
                                     byte[] expected)
      ```

      Verifies a signed `Cipher` using the signer's private key, a
      message hash, and a decryption parameter generated using the signer's
      private key and the message hash. This returns `true` if the
      contents of the signed message match `expected`, `false`
      otherwise.

      Parameters:
      :   `keys` - `Keys` for verifying
      :   `cipher` - `Cipher` to verify
      :   `expected` - the expected result

      Returns:
      :   `true` if verified, `false` otherwise


    - #### sign

      ```
      public abstract Cipher sign(Keys sign,
                                  Keys encrypt,
                                  java.lang.String message)
      ```

      Encrypt-signs the `message` using an ephemeral key `x`,
      returning a `Cipher` that is decrypt-verified by its hash and a
      decryption parameter constructed using the signer's private key and
      recipients public key (all contained within the `Cipher`). This
      does NOT issue new encryption parameters, even if `encMode` is
      `ProxyReEncryption.ENCRYPTION_MODE.DYNAMIC`, as it is being used for encrypting an
      EXISTING message, not a new one.

      Parameters:
      :   `sign` - signer's `Keys`
      :   `encrypt` - recipient's `Keys` for encrypting
      :   `message` - message to sign

      Returns:
      :   the encrypt-signed `message` as a `Cipher`


    - #### sign

      ```
      public abstract Cipher sign(Keys sign,
                                  Keys encrypt,
                                  byte[] message,
                                  ProxyReEncryption.MESSAGE_TYPE type)
      ```

      Encrypt-signs the `message` using an ephemeral key `x`,
      returning a `Cipher` that is decrypt-verified by its hash and a
      decryption parameter constructed using the signer's private key and
      recipients public key (all contained within the `Cipher`). This
      does NOT issue new encryption parameters, even if `encMode` is
      `ProxyReEncryption.ENCRYPTION_MODE.DYNAMIC`, as it is being used for encrypting an
      EXISTING message, not a new one.

      Parameters:
      :   `sign` - signer's `Keys`
      :   `encrypt` - recipient's `Keys` for encrypting
      :   `message` - message to sign
      :   `type` - `ProxyReEncryption.MESSAGE_TYPE.FORMATTED` if the passed bytes are
          the result of a `Message.toBytes()` call, as this
          result is formatted; otherwise, use
          `ProxyReEncryption.MESSAGE_TYPE.UNFORMATTED`

      Returns:
      :   the encrypt-signed `message` as a `Cipher`


    - #### sign

      ```
      public abstract Cipher sign(Keys sign,
                                  Keys encrypt,
                                  Message message)
      ```

      Encrypt-signs the `Message` using an ephemeral key `x`,
      returning a `Cipher` that is decrypt-verified by its hash and a
      decryption parameter constructed using the signer's private key and
      recipients public key (all contained within the `Cipher`). This
      does NOT issue new encryption parameters, even if `encMode` is
      `ProxyReEncryption.ENCRYPTION_MODE.DYNAMIC`, as it is being used for encrypting an
      EXISTING message, not a new one.

      Parameters:
      :   `sign` - sign `Keys`
      :   `encrypt` - encryption `Keys`
      :   `message` - `Message` to encrypt

      Returns:
      :   the encrypt-signed `Message` as a `Cipher`


    - #### sign

      ```
      public abstract Cipher sign(Keys sign,
                                  Keys encrypt,
                                  Cipher cipher)
      ```

      Encrypt-signs the `Cipher` using an ephemeral key `x`,
      returning a `Cipher` that is decrypt-verified by its hash and a
      decryption parameter constructed using the signer's private key and
      recipients public key (all contained within the `Cipher`). This
      does NOT issue new encryption parameters, even if `encMode` is
      `ProxyReEncryption.ENCRYPTION_MODE.DYNAMIC`, as it is being used for encrypting an
      EXISTING message, not a new one.

      Parameters:
      :   `sign` - sign `Keys`
      :   `encrypt` - encryption `Keys`
      :   `cipher` - `Cipher` to encrypt

      Returns:
      :   the encrypt-signed `Cipher` as a `Cipher`


    - #### verifyAndGet

      ```
      public abstract Message verifyAndGet(Keys verify,
                                           Keys decrypt,
                                           Message message)
      ```

      Decrypt-verifies a signed `Message` using the signer's private key,
      a message hash, and a decryption parameter generated using the signer's
      private key and the message hash. This is an intermediate verification
      step, as the message contents must be the result of a decrypt-verified
      `Cipher`. The contents of the message are therefore, if compounded
      signatures, a `Cipher`. This returns a decrypt-verified message.

      Parameters:
      :   `verify` - verify `Keys`
      :   `decrypt` - decryption `Keys`
      :   `message` - `Message` to decrypt

      Returns:
      :   decrypted-verified `Message` as a `Message`


    - #### verify

      ```
      public abstract boolean verify(Keys verify,
                                     Keys decrypt,
                                     Message message)
      ```

      Decrypt-verifies a signed `Message` using the signer's private key,
      a message hash, and a decryption parameter generated using the signer's
      private key and the message hash. This is an intermediate verification
      step, as the message contents must be the result of a decrypt-verified
      `Cipher`. The contents of the message are therefore, if compounded
      signatures, a `Cipher`. This returns `true` if verified,
      `false` otherwise.

      Parameters:
      :   `verify` - verify `Keys`
      :   `decrypt` - decryption `Keys`
      :   `message` - `Message` to decrypt

      Returns:
      :   `true` if verified, `false` otherwise


    - #### verify

      ```
      public abstract boolean verify(Keys verify,
                                     Keys decrypt,
                                     Message message,
                                     Message expected)
      ```

      Decrypt-verifies a signed `Message` using the signer's private key,
      a message hash, and a decryption parameter generated using the signer's
      private key and the message hash. This is an intermediate verification
      step, as the message contents must be the result of a decrypt-verified
      `Cipher`. The contents of the message are therefore, if compounded
      signatures, a `Cipher`. This returns `true` if the contents
      of the signed message match `expected`, `false` otherwise.

      Parameters:
      :   `verify` - verify `Keys`
      :   `decrypt` - decryption `Keys`
      :   `message` - `Message` to decrypt
      :   `expected` - the expected result

      Returns:
      :   `true` if verified, `false` otherwise


    - #### verify

      ```
      public abstract boolean verify(Keys verify,
                                     Keys decrypt,
                                     Message message,
                                     java.lang.String expected)
      ```

      Decrypt-verifies a signed `Message` using the signer's private key,
      a message hash, and a decryption parameter generated using the signer's
      private key and the message hash. This is an intermediate verification
      step, as the message contents must be the result of a decrypt-verified
      `Cipher`. The contents of the message are therefore, if compounded
      signatures, a `Cipher`. This returns `true` if the contents
      of the signed message match `expected`, `false` otherwise.

      Parameters:
      :   `verify` - verify `Keys`
      :   `decrypt` - decryption `Keys`
      :   `message` - `Message` to decrypt
      :   `expected` - the expected result

      Returns:
      :   `true` if verified, `false` otherwise


    - #### verify

      ```
      public abstract boolean verify(Keys verify,
                                     Keys decrypt,
                                     Message message,
                                     byte[] expected)
      ```

      Decrypt-verifies a signed `Message` using the signer's private key,
      a message hash, and a decryption parameter generated using the signer's
      private key and the message hash. This is an intermediate verification
      step, as the message contents must be the result of a decrypt-verified
      `Cipher`. The contents of the message are therefore, if compounded
      signatures, a `Cipher`. This returns `true` if the contents
      of the signed message match `expected`, `false` otherwise.

      Parameters:
      :   `verify` - verify `Keys`
      :   `decrypt` - decryption `Keys`
      :   `message` - `Message` to decrypt
      :   `expected` - the expected result

      Returns:
      :   `true` if verified, `false` otherwise


    - #### verifyAndGet

      ```
      public abstract Message verifyAndGet(Keys verify,
                                           Keys decrypt,
                                           Cipher cipher)
      ```

      Decrypt-verifies a signed `Cipher` using the signer's private key,
      a message hash, and a decryption parameter generated using the signer's
      private key and the message hash. This returns a decrypt-verified
      message.

      Parameters:
      :   `verify` - verify `Keys`
      :   `decrypt` - decryption `Keys`
      :   `cipher` - `Cipher` to decrypt

      Returns:
      :   decrypted-verified `Cipher` as a `Message`


    - #### verify

      ```
      public abstract boolean verify(Keys verify,
                                     Keys decrypt,
                                     Cipher cipher)
      ```

      Decrypt-verifies a signed `Cipher` using the signer's private key,
      a message hash, and a decryption parameter generated using the signer's
      private key and the message hash. This returns `true` if verified,
      `false` otherwise.

      Parameters:
      :   `verify` - verify `Keys`
      :   `decrypt` - decryption `Keys`
      :   `cipher` - `Cipher` to decrypt

      Returns:
      :   `true` if verified, `false` otherwise


    - #### verify

      ```
      public abstract boolean verify(Keys verify,
                                     Keys decrypt,
                                     Cipher cipher,
                                     Message expected)
      ```

      Decrypt-verifies a signed `Cipher` using the signer's private key,
      a message hash, and a decryption parameter generated using the signer's
      private key and the message hash. This returns `true` if the
      contents of the signed message match `expected`, `false`
      otherwise.

      Parameters:
      :   `verify` - verify `Keys`
      :   `decrypt` - decryption `Keys`
      :   `cipher` - `Cipher` to decrypt
      :   `expected` - the expected result

      Returns:
      :   `true` if verified, `false` otherwise


    - #### verify

      ```
      public abstract boolean verify(Keys verify,
                                     Keys decrypt,
                                     Cipher cipher,
                                     java.lang.String expected)
      ```

      Decrypt-verifies a signed `Cipher` using the signer's private key,
      a message hash, and a decryption parameter generated using the signer's
      private key and the message hash. This returns `true` if the
      contents of the signed message match `expected`, `false`
      otherwise.

      Parameters:
      :   `verify` - verify `Keys`
      :   `decrypt` - decryption `Keys`
      :   `cipher` - `Cipher` to decrypt
      :   `expected` - the expected result

      Returns:
      :   `true` if verified, `false` otherwise


    - #### verify

      ```
      public abstract boolean verify(Keys verify,
                                     Keys decrypt,
                                     Cipher cipher,
                                     byte[] expected)
      ```

      Decrypt-verifies a signed `Cipher` using the signer's private key,
      a message hash, and a decryption parameter generated using the signer's
      private key and the message hash. This returns `true` if the
      contents of the signed message match `expected`, `false`
      otherwise.

      Parameters:
      :   `verify` - verify `Keys`
      :   `decrypt` - decryption `Keys`
      :   `cipher` - `Cipher` to decrypt
      :   `expected` - the expected result

      Returns:
      :   `true` if verified, `false` otherwise


    - #### scale

      ```
      public abstract void scale(Cipher cipher,
                                 it.unisa.dia.gas.jpbc.Element scalar,
                                 ProxyReEncryption.SCALE scaleType)
      ```

      Transforms the encryption key of the `Cipher` from `from` to
      `to` by way of simple scaling.

      Parameters:
      :   `cipher` - `Cipher` to transform via scaling
      :   `scalar` - scalar `Element`
      :   `scaleType` - `ProxyReEncryption.SCALE`


    - #### scale

      ```
      public abstract void scale(Message message,
                                 it.unisa.dia.gas.jpbc.Element scalar,
                                 ProxyReEncryption.SCALE scaleType)
      ```

      Transforms the encryption key of the `Message` from `from` to
      `to` by way of simple scaling.

      Parameters:
      :   `message` - `Message` to transform via scaling
      :   `scalar` - scalar `Element`
      :   `scaleType` - `ProxyReEncryption.SCALE`


    - #### scale

      ```
      public abstract void scale(Cipher cipher,
                                 it.unisa.dia.gas.jpbc.Element from,
                                 it.unisa.dia.gas.jpbc.Element to,
                                 ProxyReEncryption.SCALE scaleType)
      ```

      Transforms the encryption key of the `Cipher` from `from` to
      `to` by way of simple scaling.

      Parameters:
      :   `cipher` - `Cipher` to transform via scaling
      :   `from` - from `Element`
      :   `to` - to `Element`
      :   `scaleType` - `ProxyReEncryption.SCALE`


    - #### scale

      ```
      public abstract void scale(Message message,
                                 it.unisa.dia.gas.jpbc.Element from,
                                 it.unisa.dia.gas.jpbc.Element to,
                                 ProxyReEncryption.SCALE scaleType)
      ```

      Transforms the encryption key of the `Message` from `from` to
      `to` by way of simple scaling.

      Parameters:
      :   `message` - `Message` to transform via scaling
      :   `from` - from `Element`
      :   `to` - to `Element`
      :   `scaleType` - `ProxyReEncryption.SCALE`


    - #### setDefaultReEncrypt

      ```
      public void setDefaultReEncrypt(ProxyReEncryption.REENCRYPT reencrypt)
      ```

      Sets the default re-encryption parameter.

      Parameters:
      :   `reencrypt` - `ProxyReEncryption.REENCRYPT` parameter


    - #### setEncryptionMode

      ```
      public void setEncryptionMode(ProxyReEncryption.ENCRYPTION_MODE encMode)
      ```

      Set the `ProxyReEncryption.ENCRYPTION_MODE`.

      Parameters:
      :   `encMode` - `ProxyReEncryption.ENCRYPTION_MODE`


    - #### setInfFlagToZero

      ```
      protected void setInfFlagToZero(it.unisa.dia.gas.plaf.jpbc.field.curve.CurveElement ce)
      ```

      Sets `infFlag` to zero. Generating a `CurveElement` object
      results in an `infFlag` of `1`. This flag must be set to
      `0` for calculations to be performed. Reflection is the only way to
      set this value directly.

      Parameters:
      :   `ce` - `CurveElement` to set


    - #### setLogLevel

      ```
      public void setLogLevel(edu.ecu.hsim.ray.logger.Logger.Level logLevel)
      ```

      Sets the log level.

      Parameters:
      :   `logLevel` - `Logger.Level`


    - #### getCurveParams

      ```
      public it.unisa.dia.gas.jpbc.PairingParameters getCurveParams()
      ```

      Returns the curve parameters.

      Returns:
      :   the curve parameters


    - #### getDefaultReEncrypt

      ```
      public ProxyReEncryption.REENCRYPT getDefaultReEncrypt()
      ```

      Returns the default re-encryption parameter.

      Returns:
      :   the default re-encryption parameter


    - #### getE

      ```
      protected it.unisa.dia.gas.jpbc.Pairing getE()
      ```

      Returns `e`.

      Returns:
      :   `e`


    - #### getEncryptionMode

      ```
      public ProxyReEncryption.ENCRYPTION_MODE getEncryptionMode()
      ```

      Returns the `ProxyReEncryption.ENCRYPTION_MODE`.

      Returns:
      :   the `ProxyReEncryption.ENCRYPTION_MODE`


    - #### getG1Gen

      ```
      public it.unisa.dia.gas.jpbc.Element getG1Gen()
      ```

      Returns the generator `g1` for `G1`.

      Returns:
      :   the generator `g1` for `G1`


    - #### getG2Gen

      ```
      public it.unisa.dia.gas.jpbc.Element getG2Gen()
      ```

      Returns the generator `g2` for `G2`.

      Returns:
      :   the generator `g2` for `G2`


    - #### getGT

      ```
      protected it.unisa.dia.gas.jpbc.Field getGT()
      ```

      Returns `GT`.

      Returns:
      :   `GT`


    - #### getG1GenP

      ```
      protected it.unisa.dia.gas.jpbc.ElementPowPreProcessing getG1GenP()
      ```

      Returns pow pre processing element `g1p` for `G1`.

      Returns:
      :   pow pre processing element `g1p` for `G1`


    - #### getG2GenP

      ```
      protected it.unisa.dia.gas.jpbc.ElementPowPreProcessing getG2GenP()
      ```

      Returns pow pre processing element `g2p` for `G2`.

      Returns:
      :   pow pre processing element `g2p` for `G2`


    - #### getOrder

      ```
      public java.math.BigInteger getOrder()
      ```

      Returns the order.

      Returns:
      :   the order


    - #### getZr

      ```
      protected it.unisa.dia.gas.jpbc.Field getZr()
      ```

      Returns `Zr`.

      Returns:
      :   `Zr`


    - #### getInfFlag

      ```
      protected java.lang.String getInfFlag(it.unisa.dia.gas.plaf.jpbc.field.curve.CurveElement ce)
      ```

      Returns `infFlag` for the `CurveElement` `ce`.

      Parameters:
      :   `ce` - `CurveElement`

      Returns:
      :   `infFlag` for the `CurveElement` `ce`


    - #### getLogLevel

      ```
      public edu.ecu.hsim.ray.logger.Logger.Level getLogLevel()
      ```

      Returns the log level.

      Returns:
      :   the log level


    - #### getCurveFile

      ```
      public java.io.File getCurveFile()
      ```

      Returns the curve file if `storage` ==
      `ProxyReEncryption.STORAGE.NONVOLATILE`.

      Returns:
      :   the curve file if `storage` ==
          `ProxyReEncryption.STORAGE.NONVOLATILE`


    - #### getCurveProperties

      ```
      public java.lang.String getCurveProperties()
      ```

      Returns the curve properties if `storage` ==
      `ProxyReEncryption.STORAGE.VOLATILE`.

      Returns:
      :   the curve properties if `storage` ==
          `ProxyReEncryption.STORAGE.VOLATILE`


    - #### readCipher

      ```
      public Cipher readCipher(java.lang.String file)
      ```

      Reads a `Cipher` from a file.

      Parameters:
      :   `file` - file name

      Returns:
      :   a `Cipher` object


    - #### readMessage

      ```
      public Message readMessage(java.lang.String file)
      ```

      Reads a `Message` from a file.

      Parameters:
      :   `file` - file name

      Returns:
      :   a `Message` object


    - #### getZP

      ```
      public it.unisa.dia.gas.jpbc.ElementPowPreProcessing getZP()
      ```

      Returns `Zp`.

      Returns:
      :   `Zp`


    - #### newG1CurveElement

      ```
      public it.unisa.dia.gas.plaf.jpbc.field.curve.CurveElement newG1CurveElement()
      ```

      Returns a new `CurveElement` derived from `G1`.

      Returns:
      :   a new `CurveElement` derived from `G1`


    - #### newG1CurveElement

      ```
      public it.unisa.dia.gas.plaf.jpbc.field.curve.CurveElement newG1CurveElement(byte[] bytes)
      ```

      Returns a new `CurveElement` derived from `G1` with its
      contents set to `bytes`.

      Parameters:
      :   `bytes` - bytes to set

      Returns:
      :   a new `CurveElement` derived from `G1` with its
          contents set to `bytes`


    - #### newG1CurveElement

      ```
      public it.unisa.dia.gas.plaf.jpbc.field.curve.CurveElement newG1CurveElement(byte[] bytes,
                                                                                   int offset)
      ```

      Returns a new `CurveElement` derived from `G1` with its
      contents set to `bytes` beginning at `offset`. `jPBC`
      will only read the number of bytes necessary to populate the element. For
      instance, if `bytes.length` = 100, but the element is only 64 bytes
      in length, then only 64 will be processed.

      Parameters:
      :   `bytes` - bytes to set
      :   `offset` - offset into the array

      Returns:
      :   a new `CurveElement` derived from `G1` with its
          contents set to `bytes`


    - #### newG2CurveElement

      ```
      public it.unisa.dia.gas.plaf.jpbc.field.curve.CurveElement newG2CurveElement()
      ```

      Returns a new `CurveElement` derived from `G2`.

      Returns:
      :   a new `CurveElement` derived from `G2`


    - #### newG2CurveElement

      ```
      public it.unisa.dia.gas.plaf.jpbc.field.curve.CurveElement newG2CurveElement(byte[] bytes)
      ```

      Returns a new `CurveElement` derived from `G2` with its
      contents set to `bytes`.

      Parameters:
      :   `bytes` - bytes to set

      Returns:
      :   a new `CurveElement` derived from `G2` with its
          contents set to `bytes`


    - #### newG2CurveElement

      ```
      public it.unisa.dia.gas.plaf.jpbc.field.curve.CurveElement newG2CurveElement(byte[] bytes,
                                                                                   int offset)
      ```

      Returns a new `CurveElement` derived from `G2` with its
      contents set to `bytes` beginning at `offset`. `jPBC`
      will only read the number of bytes necessary to populate the element. For
      instance, if `bytes.length` = 100, but the element is only 64 bytes
      in length, then only 64 will be processed.

      Parameters:
      :   `bytes` - bytes to set
      :   `offset` - offset into the array

      Returns:
      :   a new `CurveElement` derived from `G2` with its
          contents set to `bytes`


    - #### newEphemeralKeyX

      ```
      public it.unisa.dia.gas.jpbc.Element newEphemeralKeyX()
      ```

      Returns a new, random ephemeral key `x` `Element`.

      Returns:
      :   a new, random ephemeral key `x` `Element`


    - #### newEphemeralKeyX

      ```
      public it.unisa.dia.gas.jpbc.Element newEphemeralKeyX(byte[] bytes)
      ```

      Returns a new ephemeral key `Element` derived from `G` with
      its contents set to `bytes`.

      Parameters:
      :   `bytes` - bytes to set

      Returns:
      :   a new ephemeral key `Element` derived from `G` with
          its contents set to `bytes`


    - #### newGTElement

      ```
      public it.unisa.dia.gas.plaf.jpbc.field.gt.GTFiniteElement newGTElement()
      ```

      Returns a new `GTFiniteElement` derived from `GT`.

      Returns:
      :   a new `GTFiniteElement` derived from `GT`


    - #### newGTElement

      ```
      public it.unisa.dia.gas.plaf.jpbc.field.gt.GTFiniteElement newGTElement(byte[] bytes)
      ```

      Returns a new `GTFiniteElement` derived from `GT` with its
      contents set to `bytes`.

      Parameters:
      :   `bytes` - bytes to set

      Returns:
      :   a new `GTFiniteElement` derived from `GT` with its
          contents set to `bytes`


    - #### newGTElement

      ```
      public it.unisa.dia.gas.plaf.jpbc.field.gt.GTFiniteElement newGTElement(byte[] bytes,
                                                                              int offset)
      ```

      Returns a new `GTFiniteElement` derived from `GT` with its
      contents set to `bytes` beginning at `offset`. `jPBC`
      will only read the number of bytes necessary to populate the element. For
      instance, if `bytes.length` = 100, but the element is only 64 bytes
      in length, then only 64 will be processed.

      Parameters:
      :   `bytes` - bytes to set
      :   `offset` - offset into the array

      Returns:
      :   a new `GTFiniteElement` derived from `GT` with its
          contents set to `bytes`


    - #### newZrElement

      ```
      public it.unisa.dia.gas.plaf.jpbc.field.z.ZrElement newZrElement()
      ```

      Returns a new `ZrElement` derived from `Zr`.

      Returns:
      :   a new `ZrElement` derived from `Zr`


    - #### newZrElement

      ```
      public it.unisa.dia.gas.plaf.jpbc.field.z.ZrElement newZrElement(byte[] bytes)
      ```

      Returns a new `ZrElement` derived from `Zr` with its contents
      set to `bytes`.

      Parameters:
      :   `bytes` - bytes to set

      Returns:
      :   a new `ZrElement` derived from `Zr` with its contents
          set to `bytes`


    - #### newZrElement

      ```
      public it.unisa.dia.gas.plaf.jpbc.field.z.ZrElement newZrElement(byte[] bytes,
                                                                       int offset)
      ```

      Returns a new `ZrElement` derived from `Zr` with its
      contents set to `bytes` beginning at `offset`. `jPBC`
      will only read the number of bytes necessary to populate the element. For
      instance, if `bytes.length` = 100, but the element is only 64 bytes
      in length, then only 64 will be processed.

      Parameters:
      :   `bytes` - bytes to set
      :   `offset` - offset into the array

      Returns:
      :   a new `ZrElement` derived from `Zr` with its
          contents set to `bytes`


    - #### toCipher

      ```
      public abstract Cipher toCipher(byte[] bytes)
      ```

      Converts a `byte[]` to a `Cipher`.

      Parameters:
      :   `bytes` - `byte[]` to convert

      Returns:
      :   a `Cipher` object


    - #### toMessage

      ```
      public abstract Message toMessage(java.lang.String message)
      ```

      Converts a `String` to a `Message`.

      Parameters:
      :   `message` - `String` to convert

      Returns:
      :   `String` as a `Message`


    - #### toMessage

      ```
      public abstract Message toMessage(byte[] message,
                                        ProxyReEncryption.MESSAGE_TYPE type)
      ```

      Converts a `byte[]` to a `Message`. If `type` is
      `ProxyReEncryption.MESSAGE_TYPE.FORMATTED`, the `message` will be treated as a
      formatted message (i.e., the result of a `Message.toBytes()` call)
      and the structure recovered; otherwise, it will treat the `message`
      as pure data.

      Parameters:
      :   `message` - `Message` as `byte[]` to convert
      :   `type` - `ProxyReEncryption.MESSAGE_TYPE.FORMATTED` if the passed bytes are
          the result of a `Message.toBytes()` call, as this
          result is formatted; otherwise, use
          `ProxyReEncryption.MESSAGE_TYPE.UNFORMATTED`

      Returns:
      :   `byte[]` as a `Message`


    - #### toMessage

      ```
      public abstract Message toMessage(byte[] message,
                                        int bytePadding)
      ```

      Converts a `byte[]` to a `Message` with padding. The
      `message` is always treated as `ProxyReEncryption.MESSAGE_TYPE.UNFORMATTED`.
      Use this method if the data are arbitrary bytes that are not bound to
      `ASCII`. When converted to an element, they may be outside the
      range of the `order` of the elliptic curve. As such, they will be
      subject to a `mod` operation, rending the data unrecoverable. This
      allows for padding to be added in the for of a `(byte)0` to the
      beginning of each element or element set component (e.g., `X/Y`
      coordinates). This is generally used when taking the results of a
      `Message` or `Cipher` and storing it as message bytes.

      Parameters:
      :   `message` - `Message` as `byte[]` to convert
      :   `bytePadding` - the number of leading bytes in a converted element
          (either single chunk or X/Y pairs) to pad with a
          `0`

      Returns:
      :   `byte[]` as a `Message` or `null` for error


Skip navigation links


- Package
- Class
- Use
- Tree
- Deprecated
- Index
- Help

- Prev Class
- Next Class

- Frames
- No Frames

- All Classes

- Summary:
- Nested |
- Field |
- Constr |
- Method

- Detail:
- Field |
- Constr |
- Method
