# Supplementary material for: A Blockchain Framework for Patient-Centered Health Records and Exchange (HealthChain): Evaluation and Proof-of-Concept Study
Source: J Med Internet Res. 2019 Aug 31;21(8):e13592. doi: 10.2196/13592 (PMC6743266; doi:10.2196/13592)
Supplement: Multimedia Appendix 3 [file jmir_v21i8e13592_app3.zip › ProxyReEncryption/javadoc/edu/ecu/hsim/ray/proxyreencryption/ReKeys.html]

ReKeys


JavaScript is disabled on your browser.


Skip navigation links


- Package
- Class
- Use
- Tree
- Deprecated
- Index
- Help

- Prev Class
- Next Class

- Frames
- No Frames

- All Classes

- Summary:
- Nested |
- Field |
- Constr |
- Method

- Detail:
- Field |
- Constr |
- Method


edu.ecu.hsim.ray.proxyreencryption

## Class ReKeys

- java.lang.Object
- - edu.ecu.hsim.ray.proxyreencryption.Keys
  - - edu.ecu.hsim.ray.proxyreencryption.ReKeys

- ---

    

  ```
  public class ReKeys
  extends Keys
  ```

  Re-encryption key generator, writer, and reader.

- - ### Field Summary

    - ### Fields inherited from class edu.ecu.hsim.ray.proxyreencryption.Keys

      `firstLevel, fisPub, fisSec, fosPub, fosSec, k, l, publicFile, publicKey, publicProps, publicStringProperties, secondLevel, secretFile, secretKey, secretKeyI, secretProps, secretStringProperties, sisPub, sisSec, sosPub, sosSec, storage, twoPartyScalar`
  - ### Constructor Summary

    Constructors

    | Modifier | Constructor and Description |
    | --- | --- |
    | `protected` | `ReKeys(Keys delegator, Keys delegatee)` Constructs a new `ProxyReEncryption.STORAGE.VOLATILE` re-encryption key. |
    | `protected` | `ReKeys(Keys delegator, Keys delegatee, boolean setSave)` Constructs a new `ProxyReEncryption.STORAGE.VOLATILE` re-encryption key. |
    | `protected` | `ReKeys(Keys delegator, Keys intermediary, Keys delegatee)` Constructs a new two-party `ProxyReEncryption.STORAGE.VOLATILE` re-encryption key. |
    | `protected` | `ReKeys(Keys delegator, Keys intermediary, Keys delegatee, boolean setSave)` Constructs a new two-party `ProxyReEncryption.STORAGE.VOLATILE` re-encryption key. |
    | `protected` | `ReKeys(Keys delegator, Keys intermediary, Keys delegatee, java.lang.String params)` Constructs a new two-party `ProxyReEncryption.STORAGE.NONVOLATILE` re-encryption key. |
    | `protected` | `ReKeys(Keys delegator, Keys delegatee, java.lang.String params)` Constructs a new `ProxyReEncryption.STORAGE.NONVOLATILE` re-encryption key. |
    | `protected` | `ReKeys(ProxyReEncryption proxy, java.lang.String params, ProxyReEncryption.STORAGE storage)` Constructs a new `storage` re-encryption key from an existing source - `params`. |
  - ### Method Summary

    - ### Methods inherited from class edu.ecu.hsim.ray.proxyreencryption.Keys

      `close, getDecryptFirstLevelDenominator, getDecryptSecondLevelDenominator, getFirstLevel, getK, getL, getProxy, getPublicFile, getPublicKey, getPublicProperties, getSecondLevel, getSecretFile, getSecretKey, getSecretKeyInverse, getSecretProperties, getTwoPartyScalar, newK`
    - ### Methods inherited from class java.lang.Object

      `clone, equals, finalize, getClass, hashCode, notify, notifyAll, toString, wait, wait, wait`

- - ### Constructor Detail


    - #### ReKeys

      ```
      protected ReKeys(Keys delegator,
                       Keys delegatee)
      ```

      Constructs a new `ProxyReEncryption.STORAGE.VOLATILE` re-encryption key.

      Parameters:
      :   `delegator` - `Keys` delegator
      :   `delegatee` - `Keys` delegatee


    - #### ReKeys

      ```
      protected ReKeys(Keys delegator,
                       Keys intermediary,
                       Keys delegatee)
      ```

      Constructs a new two-party `ProxyReEncryption.STORAGE.VOLATILE` re-encryption key.

      Parameters:
      :   `delegator` - `Keys` delegator
      :   `intermediary` - `Keys` intermediary
      :   `delegatee` - `Keys` delegatee


    - #### ReKeys

      ```
      protected ReKeys(Keys delegator,
                       Keys delegatee,
                       boolean setSave)
      ```

      Constructs a new `ProxyReEncryption.STORAGE.VOLATILE` re-encryption key.
      `setSave` should ONLY be set to `false` during generation
      testing.

      Parameters:
      :   `delegator` - `Keys` delegator
      :   `delegatee` - `Keys` delegatee
      :   `setSave` - `true` to set and save the values, `false`
          otherwise (e.g., temporary ReKeys during generation check)


    - #### ReKeys

      ```
      protected ReKeys(Keys delegator,
                       Keys intermediary,
                       Keys delegatee,
                       boolean setSave)
      ```

      Constructs a new two-party `ProxyReEncryption.STORAGE.VOLATILE` re-encryption key.
      `setSave` should ONLY be set to `false` during generation
      testing.

      Parameters:
      :   `delegator` - `Keys` delegator
      :   `intermediary` - `Keys` intermediary
      :   `delegatee` - `Keys` delegatee
      :   `setSave` - `true` to set and save the values, `false`
          otherwise (e.g., temporary ReKeys during generation check)


    - #### ReKeys

      ```
      protected ReKeys(Keys delegator,
                       Keys delegatee,
                       java.lang.String params)
      ```

      Constructs a new `ProxyReEncryption.STORAGE.NONVOLATILE` re-encryption key.

      Parameters:
      :   `delegator` - `Keys` delegator
      :   `delegatee` - `Keys` delegatee
      :   `params` - parameters file


    - #### ReKeys

      ```
      protected ReKeys(Keys delegator,
                       Keys intermediary,
                       Keys delegatee,
                       java.lang.String params)
      ```

      Constructs a new two-party `ProxyReEncryption.STORAGE.NONVOLATILE` re-encryption key.

      Parameters:
      :   `delegator` - `Keys` delegator
      :   `intermediary` - `Keys` intermediary
      :   `delegatee` - `Keys` delegatee
      :   `params` - parameters file


    - #### ReKeys

      ```
      protected ReKeys(ProxyReEncryption proxy,
                       java.lang.String params,
                       ProxyReEncryption.STORAGE storage)
                throws java.io.IOException
      ```

      Constructs a new `storage` re-encryption key from an existing
      source - `params`.

      Parameters:
      :   `proxy` - `ProxyReEncryption`
      :   `params` - parameters
      :   `storage` - `ProxyReEncryption.STORAGE` option

      Throws:
      :   `java.io.IOException` - `IOException`


Skip navigation links


- Package
- Class
- Use
- Tree
- Deprecated
- Index
- Help

- Prev Class
- Next Class

- Frames
- No Frames

- All Classes

- Summary:
- Nested |
- Field |
- Constr |
- Method

- Detail:
- Field |
- Constr |
- Method
