# Supplementary material for: A Blockchain Framework for Patient-Centered Health Records and Exchange (HealthChain): Evaluation and Proof-of-Concept Study
Source: J Med Internet Res. 2019 Aug 31;21(8):e13592. doi: 10.2196/13592 (PMC6743266; doi:10.2196/13592)
Supplement: Multimedia Appendix 3 [file jmir_v21i8e13592_app3.zip › ProxyReEncryption/javadoc/edu/ecu/hsim/ray/proxyreencryption/TypeAPairingIO.html]

TypeAPairingIO


JavaScript is disabled on your browser.


Skip navigation links


- Package
- Class
- Use
- Tree
- Deprecated
- Index
- Help

- Prev Class
- Next Class

- Frames
- No Frames

- All Classes

- Summary:
- Nested |
- Field |
- Constr |
- Method

- Detail:
- Field |
- Constr |
- Method


edu.ecu.hsim.ray.proxyreencryption

## Class TypeAPairingIO

- java.lang.Object
- - it.unisa.dia.gas.plaf.jpbc.pairing.AbstractPairing
  - - it.unisa.dia.gas.plaf.jpbc.pairing.a.TypeAPairing
    - - edu.ecu.hsim.ray.proxyreencryption.TypeAPairingIO

- All Implemented Interfaces:
  :   it.unisa.dia.gas.jpbc.Pairing

  ---

    

  ```
  public class TypeAPairingIO
  extends it.unisa.dia.gas.plaf.jpbc.pairing.a.TypeAPairing
  ```

  Extension to `TypeAPairing` allowing for reading in parameters.

- - ### Field Summary

    - ### Fields inherited from class it.unisa.dia.gas.plaf.jpbc.pairing.a.TypeAPairing

      `Eq, exp1, exp2, Fq, Fq2, genNoCofac, h, MILLER_AFFINE_METHOD, MILLER_PROJECTTIVE_METHOD, NAF_MILLER_PROJECTTIVE_METHOD, phikOnr, q, r, sign1`
    - ### Fields inherited from class it.unisa.dia.gas.plaf.jpbc.pairing.AbstractPairing

      `G1, G2, GT, pairingMap, random, Zr`
  - ### Constructor Summary

    Constructors

    Constructor and Description

    `TypeAPairingIO(it.unisa.dia.gas.jpbc.PairingParameters params)`

    Constructor.
  - ### Method Summary

    All Methods Instance Methods Concrete Methods

    | Modifier and Type | Method and Description |
    | --- | --- |
    | `protected it.unisa.dia.gas.jpbc.Field` | `getEq()` Returns `Eq`. |
    | `protected it.unisa.dia.gas.jpbc.Field` | `getFq()` Returns `Fq`. |
    | `java.math.BigInteger` | `getOrder()` Returns the order. |
    | `protected it.unisa.dia.gas.jpbc.Field<? extends it.unisa.dia.gas.jpbc.Point>` | `initEq()` Initialize `Eq`. |
    | `protected it.unisa.dia.gas.jpbc.Field` | `initFp(java.math.BigInteger order)` Initialize `Fp`. |
    | `protected void` | `initParams(it.unisa.dia.gas.jpbc.PairingParameters curveParams)` Initialize curve parameters. |

    - ### Methods inherited from class it.unisa.dia.gas.plaf.jpbc.pairing.a.TypeAPairing

      `initFi, initFields, initGT, initMap`
    - ### Methods inherited from class it.unisa.dia.gas.plaf.jpbc.pairing.AbstractPairing

      `getDegree, getFieldAt, getFieldIndex, getG1, getG2, getGT, getPairingMap, getPairingPreProcessingFromBytes, getPairingPreProcessingFromBytes, getPairingPreProcessingFromElement, getPairingPreProcessingLengthInBytes, getZr, isAlmostCoddh, isProductPairingSupported, isSymmetric, pairing, pairing, setPairingMap`
    - ### Methods inherited from class java.lang.Object

      `clone, equals, finalize, getClass, hashCode, notify, notifyAll, toString, wait, wait, wait`

- - ### Constructor Detail


    - #### TypeAPairingIO

      ```
      public TypeAPairingIO(it.unisa.dia.gas.jpbc.PairingParameters params)
      ```

      Constructor.

      Parameters:
      :   `params` - curve parameters
  - ### Method Detail


    - #### initParams

      ```
      protected void initParams(it.unisa.dia.gas.jpbc.PairingParameters curveParams)
      ```

      Initialize curve parameters.

      Overrides:
      :   `initParams` in class `it.unisa.dia.gas.plaf.jpbc.pairing.a.TypeAPairing`

      Parameters:
      :   `curveParams` - `PairingParameters`


    - #### initFp

      ```
      protected it.unisa.dia.gas.jpbc.Field initFp(java.math.BigInteger order)
      ```

      Initialize `Fp`.

      Overrides:
      :   `initFp` in class `it.unisa.dia.gas.plaf.jpbc.pairing.a.TypeAPairing`

      Parameters:
      :   `order` - order

      Returns:
      :   `Fp` as a `ZrField`


    - #### initEq

      ```
      protected it.unisa.dia.gas.jpbc.Field<? extends it.unisa.dia.gas.jpbc.Point> initEq()
      ```

      Initialize `Eq`.

      Overrides:
      :   `initEq` in class `it.unisa.dia.gas.plaf.jpbc.pairing.a.TypeAPairing`

      Returns:
      :   `Eq` as `CurveField`


    - #### getEq

      ```
      protected it.unisa.dia.gas.jpbc.Field getEq()
      ```

      Returns `Eq`.

      Returns:
      :   `Eq`


    - #### getFq

      ```
      protected it.unisa.dia.gas.jpbc.Field getFq()
      ```

      Returns `Fq`.

      Returns:
      :   `Fq`


    - #### getOrder

      ```
      public java.math.BigInteger getOrder()
      ```

      Returns the order.

      Returns:
      :   the order


Skip navigation links


- Package
- Class
- Use
- Tree
- Deprecated
- Index
- Help

- Prev Class
- Next Class

- Frames
- No Frames

- All Classes

- Summary:
- Nested |
- Field |
- Constr |
- Method

- Detail:
- Field |
- Constr |
- Method
