# Supplementary material for: A Blockchain Framework for Patient-Centered Health Records and Exchange (HealthChain): Evaluation and Proof-of-Concept Study
Source: J Med Internet Res. 2019 Aug 31;21(8):e13592. doi: 10.2196/13592 (PMC6743266; doi:10.2196/13592)
Supplement: Multimedia Appendix 3 [file jmir_v21i8e13592_app3.zip › ProxyReEncryption/javadoc/index-files/index-1.html]

A-Index


JavaScript is disabled on your browser.


Skip navigation links


- Package
- Class
- Use
- Tree
- Deprecated
- Index
- Help

- Prev Letter
- Next Letter

- Frames
- No Frames

- All Classes

A C D E F G I K L M N O P R S T V W Z 


## A

add(Element, int) - Method in class edu.ecu.hsim.ray.proxyreencryption.Cipher
:   Add an `Element`.

add(Element, int) - Method in class edu.ecu.hsim.ray.proxyreencryption.Message
:   Add an `Element`.

AFGH - Class in edu.ecu.hsim.ray.proxyreencryption
:   Background

AFGH() - Constructor for class edu.ecu.hsim.ray.proxyreencryption.AFGH
:   Constructs a new `STORAGE#VOLATILE` `AFGH` with elliptic
    curve key length of size `AFGH.DEFAULT_LENGTH` and encryption mode of
    `ProxyReEncryption.DEFAULT_ENCRYPTION_MODE`.

AFGH(ProxyReEncryption.ENCRYPTION\_MODE) - Constructor for class edu.ecu.hsim.ray.proxyreencryption.AFGH
:   Constructs a new `STORAGE#VOLATILE` `AFGH` with elliptic
    curve key length of size `AFGH.DEFAULT_LENGTH` and k mode of
    `encMode`.

AFGH(AFGH.LENGTH) - Constructor for class edu.ecu.hsim.ray.proxyreencryption.AFGH
:   Constructs a new `STORAGE#VOLATILE` `AFGH` with elliptic
    curve key length of size `length` and encryption mode of
    `ProxyReEncryption.DEFAULT_ENCRYPTION_MODE`.

AFGH(AFGH.LENGTH, ProxyReEncryption.ENCRYPTION\_MODE) - Constructor for class edu.ecu.hsim.ray.proxyreencryption.AFGH
:   Constructs a new `STORAGE#VOLATILE` `AFGH` with elliptic
    curve key length of size `length` and encryption mode of
    `encMode`.

AFGH(byte[], ProxyReEncryption.STORAGE) - Constructor for class edu.ecu.hsim.ray.proxyreencryption.AFGH
:   Constructs a new `storage` `AFGH`.

AFGH(String, ProxyReEncryption.STORAGE) - Constructor for class edu.ecu.hsim.ray.proxyreencryption.AFGH
:   Constructs a new `storage` `AFGH`.

AFGH(byte[], ProxyReEncryption.STORAGE, ProxyReEncryption.ENCRYPTION\_MODE) - Constructor for class edu.ecu.hsim.ray.proxyreencryption.AFGH
:   Constructs a new `storage` `AFGH`.

AFGH(String, ProxyReEncryption.STORAGE, ProxyReEncryption.ENCRYPTION\_MODE) - Constructor for class edu.ecu.hsim.ray.proxyreencryption.AFGH
:   Constructs a new `storage` `AFGH`.

AFGH(AFGH.LENGTH, byte[]) - Constructor for class edu.ecu.hsim.ray.proxyreencryption.AFGH
:   Constructs a new `STORAGE#NONVOLATILE` `AFGH`.

AFGH(AFGH.LENGTH, String) - Constructor for class edu.ecu.hsim.ray.proxyreencryption.AFGH
:   Constructs a new `STORAGE#NONVOLATILE` `AFGH`.

AFGH(AFGH.LENGTH, byte[], ProxyReEncryption.ENCRYPTION\_MODE) - Constructor for class edu.ecu.hsim.ray.proxyreencryption.AFGH
:   Constructs a new `STORAGE#NONVOLATILE` `AFGH`.

AFGH(AFGH.LENGTH, String, ProxyReEncryption.ENCRYPTION\_MODE) - Constructor for class edu.ecu.hsim.ray.proxyreencryption.AFGH
:   Constructs a new `STORAGE#NONVOLATILE` `AFGH`.

AFGH.LENGTH - Enum in edu.ecu.hsim.ray.proxyreencryption
:   The employed curve is a Type A pairing Montgomery curve with embedding
    degree of \(k=2\).

A C D E F G I K L M N O P R S T V W Z

Skip navigation links


- Package
- Class
- Use
- Tree
- Deprecated
- Index
- Help

- Prev Letter
- Next Letter

- Frames
- No Frames

- All Classes
