# Supplementary material for: A Blockchain Framework for Patient-Centered Health Records and Exchange (HealthChain): Evaluation and Proof-of-Concept Study
Source: J Med Internet Res. 2019 Aug 31;21(8):e13592. doi: 10.2196/13592 (PMC6743266; doi:10.2196/13592)
Supplement: Multimedia Appendix 3 [file jmir_v21i8e13592_app3.zip › ProxyReEncryption/javadoc/index-files/index-11.html]

N-Index


JavaScript is disabled on your browser.


Skip navigation links


- Package
- Class
- Use
- Tree
- Deprecated
- Index
- Help

- Prev Letter
- Next Letter

- Frames
- No Frames

- All Classes

A C D E F G I K L M N O P R S T V W Z 


## N

newEphemeralKeyX() - Method in class edu.ecu.hsim.ray.proxyreencryption.ProxyReEncryption
:   Returns a new, random ephemeral key `x` `Element`.

newEphemeralKeyX(byte[]) - Method in class edu.ecu.hsim.ray.proxyreencryption.ProxyReEncryption
:   Returns a new ephemeral key `Element` derived from `G` with
    its contents set to `bytes`.

newG1CurveElement() - Method in class edu.ecu.hsim.ray.proxyreencryption.ProxyReEncryption
:   Returns a new `CurveElement` derived from `G1`.

newG1CurveElement(byte[]) - Method in class edu.ecu.hsim.ray.proxyreencryption.ProxyReEncryption
:   Returns a new `CurveElement` derived from `G1` with its
    contents set to `bytes`.

newG1CurveElement(byte[], int) - Method in class edu.ecu.hsim.ray.proxyreencryption.ProxyReEncryption
:   Returns a new `CurveElement` derived from `G1` with its
    contents set to `bytes` beginning at `offset`.

newG2CurveElement() - Method in class edu.ecu.hsim.ray.proxyreencryption.ProxyReEncryption
:   Returns a new `CurveElement` derived from `G2`.

newG2CurveElement(byte[]) - Method in class edu.ecu.hsim.ray.proxyreencryption.ProxyReEncryption
:   Returns a new `CurveElement` derived from `G2` with its
    contents set to `bytes`.

newG2CurveElement(byte[], int) - Method in class edu.ecu.hsim.ray.proxyreencryption.ProxyReEncryption
:   Returns a new `CurveElement` derived from `G2` with its
    contents set to `bytes` beginning at `offset`.

newGTElement() - Method in class edu.ecu.hsim.ray.proxyreencryption.ProxyReEncryption
:   Returns a new `GTFiniteElement` derived from `GT`.

newGTElement(byte[]) - Method in class edu.ecu.hsim.ray.proxyreencryption.ProxyReEncryption
:   Returns a new `GTFiniteElement` derived from `GT` with its
    contents set to `bytes`.

newGTElement(byte[], int) - Method in class edu.ecu.hsim.ray.proxyreencryption.ProxyReEncryption
:   Returns a new `GTFiniteElement` derived from `GT` with its
    contents set to `bytes` beginning at `offset`.

newK() - Method in class edu.ecu.hsim.ray.proxyreencryption.Keys
:   Generates, saves, and returns a new `Keys.k`.

newKeys() - Method in class edu.ecu.hsim.ray.proxyreencryption.ProxyReEncryption
:   Constructs a new `ProxyReEncryption.STORAGE.VOLATILE` set of `Keys`.

newKeys(String) - Method in class edu.ecu.hsim.ray.proxyreencryption.ProxyReEncryption
:   Constructs a new `ProxyReEncryption.STORAGE.NONVOLATILE` set of `Keys`.

newKeys(byte[], byte[]) - Method in class edu.ecu.hsim.ray.proxyreencryption.ProxyReEncryption
:   Constructs a new `ProxyReEncryption.STORAGE.VOLATILE` set of `Keys`.

newKeys(String, String) - Method in class edu.ecu.hsim.ray.proxyreencryption.ProxyReEncryption
:   Constructs a new `ProxyReEncryption.STORAGE.VOLATILE` set of `Keys`.

newReKeys(Keys, Keys) - Method in class edu.ecu.hsim.ray.proxyreencryption.ProxyReEncryption
:   Constructs a new `ProxyReEncryption.STORAGE.VOLATILE` re-encryption key.

newReKeys(Keys, Keys, Keys) - Method in class edu.ecu.hsim.ray.proxyreencryption.ProxyReEncryption
:   Constructs a new two-party `ProxyReEncryption.STORAGE.VOLATILE` re-encryption key.

newReKeys(Keys, Keys, String) - Method in class edu.ecu.hsim.ray.proxyreencryption.ProxyReEncryption
:   Constructs a new `ProxyReEncryption.STORAGE.NONVOLATILE` re-encryption key.

newReKeys(Keys, Keys, Keys, String) - Method in class edu.ecu.hsim.ray.proxyreencryption.ProxyReEncryption
:   Constructs a new two-party `ProxyReEncryption.STORAGE.NONVOLATILE` re-encryption key.

newReKeys(String, ProxyReEncryption.STORAGE) - Method in class edu.ecu.hsim.ray.proxyreencryption.ProxyReEncryption
:   Constructs a new `storage` re-encryption key from an existing
    source - `params`.

newZrElement() - Method in class edu.ecu.hsim.ray.proxyreencryption.ProxyReEncryption
:   Returns a new `ZrElement` derived from `Zr`.

newZrElement(byte[]) - Method in class edu.ecu.hsim.ray.proxyreencryption.ProxyReEncryption
:   Returns a new `ZrElement` derived from `Zr` with its contents
    set to `bytes`.

newZrElement(byte[], int) - Method in class edu.ecu.hsim.ray.proxyreencryption.ProxyReEncryption
:   Returns a new `ZrElement` derived from `Zr` with its
    contents set to `bytes` beginning at `offset`.

A C D E F G I K L M N O P R S T V W Z

Skip navigation links


- Package
- Class
- Use
- Tree
- Deprecated
- Index
- Help

- Prev Letter
- Next Letter

- Frames
- No Frames

- All Classes
