# Supplementary material for: A Blockchain Framework for Patient-Centered Health Records and Exchange (HealthChain): Evaluation and Proof-of-Concept Study
Source: J Med Internet Res. 2019 Aug 31;21(8):e13592. doi: 10.2196/13592 (PMC6743266; doi:10.2196/13592)
Supplement: Multimedia Appendix 3 [file jmir_v21i8e13592_app3.zip › ProxyReEncryption/javadoc/index-files/index-13.html]

P-Index


JavaScript is disabled on your browser.


Skip navigation links


- Package
- Class
- Use
- Tree
- Deprecated
- Index
- Help

- Prev Letter
- Next Letter

- Frames
- No Frames

- All Classes

A C D E F G I K L M N O P R S T V W Z 


## P

PK\_EXT - Static variable in class edu.ecu.hsim.ray.proxyreencryption.Constants
:   Public properties file extensions.

PK\_PREFIX - Static variable in class edu.ecu.hsim.ray.proxyreencryption.Constants
:   Public key key.

proxy - Variable in class edu.ecu.hsim.ray.proxyreencryption.Cipher
:   `ProxyReEncryption` object.

ProxyReEncryption - Class in edu.ecu.hsim.ray.proxyreencryption
:   Proxy re-encryption

ProxyReEncryption() - Constructor for class edu.ecu.hsim.ray.proxyreencryption.ProxyReEncryption
:   Constructor.

ProxyReEncryption.ENCRYPTION\_MODE - Enum in edu.ecu.hsim.ray.proxyreencryption
:   Encryption mode options:
    Encryption mode refers to how the encryption keys will be treated.

ProxyReEncryption.MESSAGE\_TYPE - Enum in edu.ecu.hsim.ray.proxyreencryption
:   Message `byte[]` type.

ProxyReEncryption.REENCRYPT - Enum in edu.ecu.hsim.ray.proxyreencryption
:   Re-encryption type.

ProxyReEncryption.SCALE - Enum in edu.ecu.hsim.ray.proxyreencryption
:   Scale input type.

ProxyReEncryption.STORAGE - Enum in edu.ecu.hsim.ray.proxyreencryption
:   Storage volatility options:
    Storage volatility refers how the generated keys will be stored and read.

publicFile - Variable in class edu.ecu.hsim.ray.proxyreencryption.Keys
:   Public properties `File`.

publicKey - Variable in class edu.ecu.hsim.ray.proxyreencryption.Keys
:   Public key `Element`.

publicProps - Variable in class edu.ecu.hsim.ray.proxyreencryption.Keys
:   Public `Properties` file.

publicStringProperties - Variable in class edu.ecu.hsim.ray.proxyreencryption.Keys
:   Public properties as `String`.

A C D E F G I K L M N O P R S T V W Z

Skip navigation links


- Package
- Class
- Use
- Tree
- Deprecated
- Index
- Help

- Prev Letter
- Next Letter

- Frames
- No Frames

- All Classes
