# Supplementary material for: A Blockchain Framework for Patient-Centered Health Records and Exchange (HealthChain): Evaluation and Proof-of-Concept Study
Source: J Med Internet Res. 2019 Aug 31;21(8):e13592. doi: 10.2196/13592 (PMC6743266; doi:10.2196/13592)
Supplement: Multimedia Appendix 3 [file jmir_v21i8e13592_app3.zip › ProxyReEncryption/javadoc/index-files/index-14.html]

R-Index


JavaScript is disabled on your browser.


Skip navigation links


- Package
- Class
- Use
- Tree
- Deprecated
- Index
- Help

- Prev Letter
- Next Letter

- Frames
- No Frames

- All Classes

A C D E F G I K L M N O P R S T V W Z 


## R

readCipher(String) - Method in class edu.ecu.hsim.ray.proxyreencryption.ProxyReEncryption
:   Reads a `Cipher` from a file.

readMessage(String) - Method in class edu.ecu.hsim.ray.proxyreencryption.ProxyReEncryption
:   Reads a `Message` from a file.

reencrypt(Keys, Cipher) - Method in class edu.ecu.hsim.ray.proxyreencryption.AFGH
:   Re-encrypts a `Cipher` using the re-encryption `Keys`.

reencrypt(Keys, Keys, Cipher) - Method in class edu.ecu.hsim.ray.proxyreencryption.AFGH
:   Re-encrypts a two-party `Cipher` using the re-encryption
    `Keys`.

reencrypt(Keys, Cipher) - Method in class edu.ecu.hsim.ray.proxyreencryption.ProxyReEncryption
:   Re-encrypts a `Cipher` using the re-encryption `Keys`.

reencrypt(Keys, Keys, Cipher) - Method in class edu.ecu.hsim.ray.proxyreencryption.ProxyReEncryption
:   Re-encrypts a two-party `Cipher` using the re-encryption
    `Keys`.

reencryptionPairing - Variable in class edu.ecu.hsim.ray.proxyreencryption.Cipher
:   Re-encryption pairing - re-encryption/two-party/sign/encrypt-sign

reencryptionScalar - Variable in class edu.ecu.hsim.ray.proxyreencryption.Cipher
:   Re-encryption scalar - two-party/sign/encrypt-sign

ReKeys - Class in edu.ecu.hsim.ray.proxyreencryption
:   Re-encryption key generator, writer, and reader.

ReKeys(Keys, Keys) - Constructor for class edu.ecu.hsim.ray.proxyreencryption.ReKeys
:   Constructs a new `ProxyReEncryption.STORAGE.VOLATILE` re-encryption key.

ReKeys(Keys, Keys, Keys) - Constructor for class edu.ecu.hsim.ray.proxyreencryption.ReKeys
:   Constructs a new two-party `ProxyReEncryption.STORAGE.VOLATILE` re-encryption key.

ReKeys(Keys, Keys, boolean) - Constructor for class edu.ecu.hsim.ray.proxyreencryption.ReKeys
:   Constructs a new `ProxyReEncryption.STORAGE.VOLATILE` re-encryption key.

ReKeys(Keys, Keys, Keys, boolean) - Constructor for class edu.ecu.hsim.ray.proxyreencryption.ReKeys
:   Constructs a new two-party `ProxyReEncryption.STORAGE.VOLATILE` re-encryption key.

ReKeys(Keys, Keys, String) - Constructor for class edu.ecu.hsim.ray.proxyreencryption.ReKeys
:   Constructs a new `ProxyReEncryption.STORAGE.NONVOLATILE` re-encryption key.

ReKeys(Keys, Keys, Keys, String) - Constructor for class edu.ecu.hsim.ray.proxyreencryption.ReKeys
:   Constructs a new two-party `ProxyReEncryption.STORAGE.NONVOLATILE` re-encryption key.

ReKeys(ProxyReEncryption, String, ProxyReEncryption.STORAGE) - Constructor for class edu.ecu.hsim.ray.proxyreencryption.ReKeys
:   Constructs a new `storage` re-encryption key from an existing
    source - `params`.

RK\_ENCP - Static variable in class edu.ecu.hsim.ray.proxyreencryption.Constants
:   Re-encryption key encryption parameter key.

RK\_EXT - Static variable in class edu.ecu.hsim.ray.proxyreencryption.Constants
:   Re-encryption key file extension.

RK\_RKEY - Static variable in class edu.ecu.hsim.ray.proxyreencryption.Constants
:   Re-encryption key key.

RK\_TPS - Static variable in class edu.ecu.hsim.ray.proxyreencryption.Constants
:   Two-party scalar.

A C D E F G I K L M N O P R S T V W Z

Skip navigation links


- Package
- Class
- Use
- Tree
- Deprecated
- Index
- Help

- Prev Letter
- Next Letter

- Frames
- No Frames

- All Classes
