# Supplementary material for: A Blockchain Framework for Patient-Centered Health Records and Exchange (HealthChain): Evaluation and Proof-of-Concept Study
Source: J Med Internet Res. 2019 Aug 31;21(8):e13592. doi: 10.2196/13592 (PMC6743266; doi:10.2196/13592)
Supplement: Multimedia Appendix 3 [file jmir_v21i8e13592_app3.zip › ProxyReEncryption/javadoc/index-files/index-15.html]

S-Index


JavaScript is disabled on your browser.


Skip navigation links


- Package
- Class
- Use
- Tree
- Deprecated
- Index
- Help

- Prev Letter
- Next Letter

- Frames
- No Frames

- All Classes

A C D E F G I K L M N O P R S T V W Z 


## S

scale(Cipher, Element, ProxyReEncryption.SCALE) - Method in class edu.ecu.hsim.ray.proxyreencryption.AFGH


scale(Message, Element, ProxyReEncryption.SCALE) - Method in class edu.ecu.hsim.ray.proxyreencryption.AFGH


scale(Cipher, Element, Element, ProxyReEncryption.SCALE) - Method in class edu.ecu.hsim.ray.proxyreencryption.AFGH


scale(Message, Element, Element, ProxyReEncryption.SCALE) - Method in class edu.ecu.hsim.ray.proxyreencryption.AFGH


scale(Cipher, Element, ProxyReEncryption.SCALE) - Method in class edu.ecu.hsim.ray.proxyreencryption.ProxyReEncryption
:   Transforms the encryption key of the `Cipher` from `from` to
    `to` by way of simple scaling.

scale(Message, Element, ProxyReEncryption.SCALE) - Method in class edu.ecu.hsim.ray.proxyreencryption.ProxyReEncryption
:   Transforms the encryption key of the `Message` from `from` to
    `to` by way of simple scaling.

scale(Cipher, Element, Element, ProxyReEncryption.SCALE) - Method in class edu.ecu.hsim.ray.proxyreencryption.ProxyReEncryption
:   Transforms the encryption key of the `Cipher` from `from` to
    `to` by way of simple scaling.

scale(Message, Element, Element, ProxyReEncryption.SCALE) - Method in class edu.ecu.hsim.ray.proxyreencryption.ProxyReEncryption
:   Transforms the encryption key of the `Message` from `from` to
    `to` by way of simple scaling.

secondLevel - Variable in class edu.ecu.hsim.ray.proxyreencryption.Keys
:   Second-level `Element`.

secretFile - Variable in class edu.ecu.hsim.ray.proxyreencryption.Keys
:   Secret properties `File`.

secretKey - Variable in class edu.ecu.hsim.ray.proxyreencryption.Keys
:   Secrete key `Element`.

secretKeyI - Variable in class edu.ecu.hsim.ray.proxyreencryption.Keys
:   Secret key inverse `Element`.

secretProps - Variable in class edu.ecu.hsim.ray.proxyreencryption.Keys
:   Secret `Properties` file.

secretStringProperties - Variable in class edu.ecu.hsim.ray.proxyreencryption.Keys
:   Secret properties as `String`.

setDecryptionParameter(Element) - Method in class edu.ecu.hsim.ray.proxyreencryption.Cipher
:   Sets the X/Y values for the decryption parameter pairing element.

setDefaultReEncrypt(ProxyReEncryption.REENCRYPT) - Method in class edu.ecu.hsim.ray.proxyreencryption.ProxyReEncryption
:   Sets the default re-encryption parameter.

setEncryptionMode(ProxyReEncryption.ENCRYPTION\_MODE) - Method in class edu.ecu.hsim.ray.proxyreencryption.ProxyReEncryption
:   Set the `ProxyReEncryption.ENCRYPTION_MODE`.

setInfFlagToZero(CurveElement) - Method in class edu.ecu.hsim.ray.proxyreencryption.ProxyReEncryption
:   Sets `infFlag` to zero.

setLogLevel(Logger.Level) - Method in class edu.ecu.hsim.ray.proxyreencryption.ProxyReEncryption
:   Sets the log level.

setReencryptionPairing(byte[]) - Method in class edu.ecu.hsim.ray.proxyreencryption.Cipher
:   Sets the X/Y values for the re-encryption pairing element.

setReencryptionPairing(byte[], Cipher.PAIRING\_TYPE) - Method in class edu.ecu.hsim.ray.proxyreencryption.Cipher
:   Sets the X/Y values for the re-encryption pairing element.

setReencryptionPairing(Element) - Method in class edu.ecu.hsim.ray.proxyreencryption.Cipher
:   Sets the X/Y values for the re-encryption pairing element.

setReencryptionScalar(byte[]) - Method in class edu.ecu.hsim.ray.proxyreencryption.Cipher
:   Sets the value of the intermediate re-encryption scalar element.

setReencryptionScalar(byte[], Cipher.PAIRING\_TYPE) - Method in class edu.ecu.hsim.ray.proxyreencryption.Cipher
:   Sets the value of the intermediate re-encryption scalar element.

setReencryptionScalar(Element) - Method in class edu.ecu.hsim.ray.proxyreencryption.Cipher
:   Sets the value of the intermediate re-encryption scalar element.

setSignedHash(Element) - Method in class edu.ecu.hsim.ray.proxyreencryption.Cipher
:   Sets the hash value of a signed/encrypt-signed message.

setToTwoParty() - Method in class edu.ecu.hsim.ray.proxyreencryption.Cipher
:   Sets the `Cipher.LEVEL` to `Cipher.LEVEL.TWOPARTY`.

sign(Keys, String) - Method in class edu.ecu.hsim.ray.proxyreencryption.AFGH


sign(Keys, byte[], ProxyReEncryption.MESSAGE\_TYPE) - Method in class edu.ecu.hsim.ray.proxyreencryption.AFGH


sign(Keys, Message) - Method in class edu.ecu.hsim.ray.proxyreencryption.AFGH


sign(Keys, Cipher) - Method in class edu.ecu.hsim.ray.proxyreencryption.AFGH


sign(Keys, Keys, String) - Method in class edu.ecu.hsim.ray.proxyreencryption.AFGH


sign(Keys, Keys, byte[], ProxyReEncryption.MESSAGE\_TYPE) - Method in class edu.ecu.hsim.ray.proxyreencryption.AFGH


sign(Keys, Keys, Message) - Method in class edu.ecu.hsim.ray.proxyreencryption.AFGH


sign(Keys, Keys, Cipher) - Method in class edu.ecu.hsim.ray.proxyreencryption.AFGH


sign(Keys, String) - Method in class edu.ecu.hsim.ray.proxyreencryption.ProxyReEncryption
:   Signs the `message` using an ephemeral key `x`, returning a
    `Cipher` that is verified by its hash and a private key-based
    decryption parameter (all contained within the `Cipher`).

sign(Keys, byte[], ProxyReEncryption.MESSAGE\_TYPE) - Method in class edu.ecu.hsim.ray.proxyreencryption.ProxyReEncryption
:   Signs the `message` using an ephemeral key `x`, returning a
    `Cipher` that is verified by its hash and a private key-based
    decryption parameter (all contained within the `Cipher`).

sign(Keys, Message) - Method in class edu.ecu.hsim.ray.proxyreencryption.ProxyReEncryption
:   Signs the `Message` using an ephemeral key `x`, returning a
    `Cipher` that is verified by its hash and a private key-based
    decryption parameter (all contained within the `Cipher`).

sign(Keys, Cipher) - Method in class edu.ecu.hsim.ray.proxyreencryption.ProxyReEncryption
:   Signs the `Cipher` using an ephemeral key `x`, returning a
    `Cipher` that is verified by its hash and a private key-based
    decryption parameter (all contained within the `Cipher`).

sign(Keys, Keys, String) - Method in class edu.ecu.hsim.ray.proxyreencryption.ProxyReEncryption
:   Encrypt-signs the `message` using an ephemeral key `x`,
    returning a `Cipher` that is decrypt-verified by its hash and a
    decryption parameter constructed using the signer's private key and
    recipients public key (all contained within the `Cipher`).

sign(Keys, Keys, byte[], ProxyReEncryption.MESSAGE\_TYPE) - Method in class edu.ecu.hsim.ray.proxyreencryption.ProxyReEncryption
:   Encrypt-signs the `message` using an ephemeral key `x`,
    returning a `Cipher` that is decrypt-verified by its hash and a
    decryption parameter constructed using the signer's private key and
    recipients public key (all contained within the `Cipher`).

sign(Keys, Keys, Message) - Method in class edu.ecu.hsim.ray.proxyreencryption.ProxyReEncryption
:   Encrypt-signs the `Message` using an ephemeral key `x`,
    returning a `Cipher` that is decrypt-verified by its hash and a
    decryption parameter constructed using the signer's private key and
    recipients public key (all contained within the `Cipher`).

sign(Keys, Keys, Cipher) - Method in class edu.ecu.hsim.ray.proxyreencryption.ProxyReEncryption
:   Encrypt-signs the `Cipher` using an ephemeral key `x`,
    returning a `Cipher` that is decrypt-verified by its hash and a
    decryption parameter constructed using the signer's private key and
    recipients public key (all contained within the `Cipher`).

signedHash - Variable in class edu.ecu.hsim.ray.proxyreencryption.Cipher
:   Signed hash - two-party/sign/encrypt-sign

sisPub - Variable in class edu.ecu.hsim.ray.proxyreencryption.Keys
:   Public properties `StringReader`.

sisSec - Variable in class edu.ecu.hsim.ray.proxyreencryption.Keys
:   Secret properties `StringReader`.

SK\_EXT - Static variable in class edu.ecu.hsim.ray.proxyreencryption.Constants
:   Secret properties file extension.

sosPub - Variable in class edu.ecu.hsim.ray.proxyreencryption.Keys
:   Public properties `StringWriter`.

sosSec - Variable in class edu.ecu.hsim.ray.proxyreencryption.Keys
:   Secret properties `StringWriter`.

storage - Variable in class edu.ecu.hsim.ray.proxyreencryption.Keys
:   `ProxyReEncryption.STORAGE` parameter.

storage - Variable in class edu.ecu.hsim.ray.proxyreencryption.ProxyReEncryption
:   `ProxyReEncryption.STORAGE` parameter.

A C D E F G I K L M N O P R S T V W Z

Skip navigation links


- Package
- Class
- Use
- Tree
- Deprecated
- Index
- Help

- Prev Letter
- Next Letter

- Frames
- No Frames

- All Classes
