# Supplementary material for: A Blockchain Framework for Patient-Centered Health Records and Exchange (HealthChain): Evaluation and Proof-of-Concept Study
Source: J Med Internet Res. 2019 Aug 31;21(8):e13592. doi: 10.2196/13592 (PMC6743266; doi:10.2196/13592)
Supplement: Multimedia Appendix 3 [file jmir_v21i8e13592_app3.zip › ProxyReEncryption/javadoc/index-files/index-16.html]

T-Index


JavaScript is disabled on your browser.


Skip navigation links


- Package
- Class
- Use
- Tree
- Deprecated
- Index
- Help

- Prev Letter
- Next Letter

- Frames
- No Frames

- All Classes

A C D E F G I K L M N O P R S T V W Z 


## T

toBytes() - Method in class edu.ecu.hsim.ray.proxyreencryption.Cipher
:   Converts a `Cipher` to a `byte[]`.

toBytes() - Method in class edu.ecu.hsim.ray.proxyreencryption.Message
:   Converts a `Message` to a `byte[]`.

toBytesDataOnly() - Method in class edu.ecu.hsim.ray.proxyreencryption.Message
:   Converts a `Message` to a `byte[]` of the data only (no
    metadata).

toBytesDataOnly(int) - Method in class edu.ecu.hsim.ray.proxyreencryption.Message
:   Converts a `Message` to a `byte[]` of the data only (no
    metadata), extracting any added padding via
    `ProxyReEncryption.toMessage(byte[], int)`.

toBytesDataOnly(ByteArrayOutputStream) - Method in class edu.ecu.hsim.ray.proxyreencryption.Message
:   Converts a `Message` to a `byte[]` of the data only (no
    metadata), stored in the `baos` `ByteArrayOutputStream`
    parameter.

toCipher(byte[]) - Method in class edu.ecu.hsim.ray.proxyreencryption.AFGH


toCipher(byte[]) - Method in class edu.ecu.hsim.ray.proxyreencryption.ProxyReEncryption
:   Converts a `byte[]` to a `Cipher`.

toHashBytes() - Method in class edu.ecu.hsim.ray.proxyreencryption.Cipher
:   Converts a `Cipher` to a `byte[]` suitable for hashing.

toHashBytes() - Method in class edu.ecu.hsim.ray.proxyreencryption.Message
:   Converts a `Message` to a `byte[]` suitable for hashing.

toMessage(String) - Method in class edu.ecu.hsim.ray.proxyreencryption.AFGH


toMessage(byte[], ProxyReEncryption.MESSAGE\_TYPE) - Method in class edu.ecu.hsim.ray.proxyreencryption.AFGH


toMessage(byte[], int) - Method in class edu.ecu.hsim.ray.proxyreencryption.AFGH


toMessage(String) - Method in class edu.ecu.hsim.ray.proxyreencryption.ProxyReEncryption
:   Converts a `String` to a `Message`.

toMessage(byte[], ProxyReEncryption.MESSAGE\_TYPE) - Method in class edu.ecu.hsim.ray.proxyreencryption.ProxyReEncryption
:   Converts a `byte[]` to a `Message`.

toMessage(byte[], int) - Method in class edu.ecu.hsim.ray.proxyreencryption.ProxyReEncryption
:   Converts a `byte[]` to a `Message` with padding.

toString() - Method in class edu.ecu.hsim.ray.proxyreencryption.Cipher


toString() - Method in class edu.ecu.hsim.ray.proxyreencryption.Message


twoPartyScalar - Variable in class edu.ecu.hsim.ray.proxyreencryption.Keys
:   Two-party re-encryption scalar `Element`.

TypeAPairingIO - Class in edu.ecu.hsim.ray.proxyreencryption
:   Extension to `TypeAPairing` allowing for reading in parameters.

TypeAPairingIO(PairingParameters) - Constructor for class edu.ecu.hsim.ray.proxyreencryption.TypeAPairingIO
:   Constructor.

A C D E F G I K L M N O P R S T V W Z

Skip navigation links


- Package
- Class
- Use
- Tree
- Deprecated
- Index
- Help

- Prev Letter
- Next Letter

- Frames
- No Frames

- All Classes
