# Supplementary material for: A Blockchain Framework for Patient-Centered Health Records and Exchange (HealthChain): Evaluation and Proof-of-Concept Study
Source: J Med Internet Res. 2019 Aug 31;21(8):e13592. doi: 10.2196/13592 (PMC6743266; doi:10.2196/13592)
Supplement: Multimedia Appendix 3 [file jmir_v21i8e13592_app3.zip › ProxyReEncryption/javadoc/index-files/index-17.html]

V-Index


JavaScript is disabled on your browser.


Skip navigation links


- Package
- Class
- Use
- Tree
- Deprecated
- Index
- Help

- Prev Letter
- Next Letter

- Frames
- No Frames

- All Classes

A C D E F G I K L M N O P R S T V W Z 


## V

valueOf(String) - Static method in enum edu.ecu.hsim.ray.proxyreencryption.AFGH.LENGTH
:   Returns the enum constant of this type with the specified name.

valueOf(String) - Static method in enum edu.ecu.hsim.ray.proxyreencryption.Cipher.LEVEL
:   Returns the enum constant of this type with the specified name.

valueOf(String) - Static method in enum edu.ecu.hsim.ray.proxyreencryption.Cipher.PAIRING\_TYPE
:   Returns the enum constant of this type with the specified name.

valueOf(String) - Static method in enum edu.ecu.hsim.ray.proxyreencryption.ProxyReEncryption.ENCRYPTION\_MODE
:   Returns the enum constant of this type with the specified name.

valueOf(String) - Static method in enum edu.ecu.hsim.ray.proxyreencryption.ProxyReEncryption.MESSAGE\_TYPE
:   Returns the enum constant of this type with the specified name.

valueOf(String) - Static method in enum edu.ecu.hsim.ray.proxyreencryption.ProxyReEncryption.REENCRYPT
:   Returns the enum constant of this type with the specified name.

valueOf(String) - Static method in enum edu.ecu.hsim.ray.proxyreencryption.ProxyReEncryption.SCALE
:   Returns the enum constant of this type with the specified name.

valueOf(String) - Static method in enum edu.ecu.hsim.ray.proxyreencryption.ProxyReEncryption.STORAGE
:   Returns the enum constant of this type with the specified name.

values() - Static method in enum edu.ecu.hsim.ray.proxyreencryption.AFGH.LENGTH
:   Returns an array containing the constants of this enum type, in
    the order they are declared.

values() - Static method in enum edu.ecu.hsim.ray.proxyreencryption.Cipher.LEVEL
:   Returns an array containing the constants of this enum type, in
    the order they are declared.

values() - Static method in enum edu.ecu.hsim.ray.proxyreencryption.Cipher.PAIRING\_TYPE
:   Returns an array containing the constants of this enum type, in
    the order they are declared.

values() - Static method in enum edu.ecu.hsim.ray.proxyreencryption.ProxyReEncryption.ENCRYPTION\_MODE
:   Returns an array containing the constants of this enum type, in
    the order they are declared.

values() - Static method in enum edu.ecu.hsim.ray.proxyreencryption.ProxyReEncryption.MESSAGE\_TYPE
:   Returns an array containing the constants of this enum type, in
    the order they are declared.

values() - Static method in enum edu.ecu.hsim.ray.proxyreencryption.ProxyReEncryption.REENCRYPT
:   Returns an array containing the constants of this enum type, in
    the order they are declared.

values() - Static method in enum edu.ecu.hsim.ray.proxyreencryption.ProxyReEncryption.SCALE
:   Returns an array containing the constants of this enum type, in
    the order they are declared.

values() - Static method in enum edu.ecu.hsim.ray.proxyreencryption.ProxyReEncryption.STORAGE
:   Returns an array containing the constants of this enum type, in
    the order they are declared.

verify(Keys, Message) - Method in class edu.ecu.hsim.ray.proxyreencryption.AFGH


verify(Keys, Message, Message) - Method in class edu.ecu.hsim.ray.proxyreencryption.AFGH


verify(Keys, Message, String) - Method in class edu.ecu.hsim.ray.proxyreencryption.AFGH


verify(Keys, Message, byte[]) - Method in class edu.ecu.hsim.ray.proxyreencryption.AFGH


verify(Keys, Cipher) - Method in class edu.ecu.hsim.ray.proxyreencryption.AFGH


verify(Keys, Cipher, Message) - Method in class edu.ecu.hsim.ray.proxyreencryption.AFGH


verify(Keys, Cipher, String) - Method in class edu.ecu.hsim.ray.proxyreencryption.AFGH


verify(Keys, Cipher, byte[]) - Method in class edu.ecu.hsim.ray.proxyreencryption.AFGH


verify(Keys, Keys, Message) - Method in class edu.ecu.hsim.ray.proxyreencryption.AFGH


verify(Keys, Keys, Message, Message) - Method in class edu.ecu.hsim.ray.proxyreencryption.AFGH


verify(Keys, Keys, Message, String) - Method in class edu.ecu.hsim.ray.proxyreencryption.AFGH


verify(Keys, Keys, Message, byte[]) - Method in class edu.ecu.hsim.ray.proxyreencryption.AFGH


verify(Keys, Keys, Cipher) - Method in class edu.ecu.hsim.ray.proxyreencryption.AFGH


verify(Keys, Keys, Cipher, Message) - Method in class edu.ecu.hsim.ray.proxyreencryption.AFGH


verify(Keys, Keys, Cipher, String) - Method in class edu.ecu.hsim.ray.proxyreencryption.AFGH


verify(Keys, Keys, Cipher, byte[]) - Method in class edu.ecu.hsim.ray.proxyreencryption.AFGH


verify(Keys, Message) - Method in class edu.ecu.hsim.ray.proxyreencryption.ProxyReEncryption
:   Verifies a signed `Message` using the signer's private key, a
    message hash, and a decryption parameter generated using the signer's
    private key and the message hash.

verify(Keys, Message, Message) - Method in class edu.ecu.hsim.ray.proxyreencryption.ProxyReEncryption
:   Verifies a signed `Message` using the signer's private key, a
    message hash, and a decryption parameter generated using the signer's
    private key and the message hash.

verify(Keys, Message, String) - Method in class edu.ecu.hsim.ray.proxyreencryption.ProxyReEncryption
:   Verifies a signed `Message` using the signer's private key, a
    message hash, and a decryption parameter generated using the signer's
    private key and the message hash.

verify(Keys, Message, byte[]) - Method in class edu.ecu.hsim.ray.proxyreencryption.ProxyReEncryption
:   Verifies a signed `Message` using the signer's private key, a
    message hash, and a decryption parameter generated using the signer's
    private key and the message hash.

verify(Keys, Cipher) - Method in class edu.ecu.hsim.ray.proxyreencryption.ProxyReEncryption
:   Verifies a signed `Cipher` using the signer's private key, a
    message hash, and a decryption parameter generated using the signer's
    private key and the message hash.

verify(Keys, Cipher, Message) - Method in class edu.ecu.hsim.ray.proxyreencryption.ProxyReEncryption
:   Verifies a signed `Cipher` using the signer's private key, a
    message hash, and a decryption parameter generated using the signer's
    private key and the message hash.

verify(Keys, Cipher, String) - Method in class edu.ecu.hsim.ray.proxyreencryption.ProxyReEncryption
:   Verifies a signed `Cipher` using the signer's private key, a
    message hash, and a decryption parameter generated using the signer's
    private key and the message hash.

verify(Keys, Cipher, byte[]) - Method in class edu.ecu.hsim.ray.proxyreencryption.ProxyReEncryption
:   Verifies a signed `Cipher` using the signer's private key, a
    message hash, and a decryption parameter generated using the signer's
    private key and the message hash.

verify(Keys, Keys, Message) - Method in class edu.ecu.hsim.ray.proxyreencryption.ProxyReEncryption
:   Decrypt-verifies a signed `Message` using the signer's private key,
    a message hash, and a decryption parameter generated using the signer's
    private key and the message hash.

verify(Keys, Keys, Message, Message) - Method in class edu.ecu.hsim.ray.proxyreencryption.ProxyReEncryption
:   Decrypt-verifies a signed `Message` using the signer's private key,
    a message hash, and a decryption parameter generated using the signer's
    private key and the message hash.

verify(Keys, Keys, Message, String) - Method in class edu.ecu.hsim.ray.proxyreencryption.ProxyReEncryption
:   Decrypt-verifies a signed `Message` using the signer's private key,
    a message hash, and a decryption parameter generated using the signer's
    private key and the message hash.

verify(Keys, Keys, Message, byte[]) - Method in class edu.ecu.hsim.ray.proxyreencryption.ProxyReEncryption
:   Decrypt-verifies a signed `Message` using the signer's private key,
    a message hash, and a decryption parameter generated using the signer's
    private key and the message hash.

verify(Keys, Keys, Cipher) - Method in class edu.ecu.hsim.ray.proxyreencryption.ProxyReEncryption
:   Decrypt-verifies a signed `Cipher` using the signer's private key,
    a message hash, and a decryption parameter generated using the signer's
    private key and the message hash.

verify(Keys, Keys, Cipher, Message) - Method in class edu.ecu.hsim.ray.proxyreencryption.ProxyReEncryption
:   Decrypt-verifies a signed `Cipher` using the signer's private key,
    a message hash, and a decryption parameter generated using the signer's
    private key and the message hash.

verify(Keys, Keys, Cipher, String) - Method in class edu.ecu.hsim.ray.proxyreencryption.ProxyReEncryption
:   Decrypt-verifies a signed `Cipher` using the signer's private key,
    a message hash, and a decryption parameter generated using the signer's
    private key and the message hash.

verify(Keys, Keys, Cipher, byte[]) - Method in class edu.ecu.hsim.ray.proxyreencryption.ProxyReEncryption
:   Decrypt-verifies a signed `Cipher` using the signer's private key,
    a message hash, and a decryption parameter generated using the signer's
    private key and the message hash.

verifyAndGet(Keys, Message) - Method in class edu.ecu.hsim.ray.proxyreencryption.AFGH


verifyAndGet(Keys, Cipher) - Method in class edu.ecu.hsim.ray.proxyreencryption.AFGH


verifyAndGet(Keys, Keys, Message) - Method in class edu.ecu.hsim.ray.proxyreencryption.AFGH


verifyAndGet(Keys, Keys, Cipher) - Method in class edu.ecu.hsim.ray.proxyreencryption.AFGH


verifyAndGet(Keys, Message) - Method in class edu.ecu.hsim.ray.proxyreencryption.ProxyReEncryption
:   Verifies a signed `Message` using the signer's private key, a
    message hash, and a decryption parameter generated using the signer's
    private key and the message hash.

verifyAndGet(Keys, Cipher) - Method in class edu.ecu.hsim.ray.proxyreencryption.ProxyReEncryption
:   Verifies a signed `Cipher` using the signer's private key, a
    message hash, and a decryption parameter generated using the signer's
    private key and the message hash.

verifyAndGet(Keys, Keys, Message) - Method in class edu.ecu.hsim.ray.proxyreencryption.ProxyReEncryption
:   Decrypt-verifies a signed `Message` using the signer's private key,
    a message hash, and a decryption parameter generated using the signer's
    private key and the message hash.

verifyAndGet(Keys, Keys, Cipher) - Method in class edu.ecu.hsim.ray.proxyreencryption.ProxyReEncryption
:   Decrypt-verifies a signed `Cipher` using the signer's private key,
    a message hash, and a decryption parameter generated using the signer's
    private key and the message hash.

A C D E F G I K L M N O P R S T V W Z

Skip navigation links


- Package
- Class
- Use
- Tree
- Deprecated
- Index
- Help

- Prev Letter
- Next Letter

- Frames
- No Frames

- All Classes
