# Supplementary material for: A Blockchain Framework for Patient-Centered Health Records and Exchange (HealthChain): Evaluation and Proof-of-Concept Study
Source: J Med Internet Res. 2019 Aug 31;21(8):e13592. doi: 10.2196/13592 (PMC6743266; doi:10.2196/13592)
Supplement: Multimedia Appendix 3 [file jmir_v21i8e13592_app3.zip › ProxyReEncryption/javadoc/index-files/index-19.html]

Z-Index


JavaScript is disabled on your browser.


Skip navigation links


- Package
- Class
- Use
- Tree
- Deprecated
- Index
- Help

- Prev Letter
- Next Letter

- Frames
- No Frames

- All Classes

A C D E F G I K L M N O P R S T V W Z 


## Z

Z - Variable in class edu.ecu.hsim.ray.proxyreencryption.ProxyReEncryption
:   `Element` `Z = e(g1, g2)`.

Zp - Variable in class edu.ecu.hsim.ray.proxyreencryption.ProxyReEncryption
:   `Z` power pre-processing (`ElementPowPreProcessing`).

ZP\_PREFIX - Static variable in class edu.ecu.hsim.ray.proxyreencryption.Constants
:   Zp key.

Zr - Variable in class edu.ecu.hsim.ray.proxyreencryption.ProxyReEncryption
:   `Field` `Zr`.

ZR\_FQ\_PREFIX - Static variable in class edu.ecu.hsim.ray.proxyreencryption.Constants
:   Zr key.

A C D E F G I K L M N O P R S T V W Z

Skip navigation links


- Package
- Class
- Use
- Tree
- Deprecated
- Index
- Help

- Prev Letter
- Next Letter

- Frames
- No Frames

- All Classes
