# Supplementary material for: A Blockchain Framework for Patient-Centered Health Records and Exchange (HealthChain): Evaluation and Proof-of-Concept Study
Source: J Med Internet Res. 2019 Aug 31;21(8):e13592. doi: 10.2196/13592 (PMC6743266; doi:10.2196/13592)
Supplement: Multimedia Appendix 3 [file jmir_v21i8e13592_app3.zip › ProxyReEncryption/javadoc/index-files/index-2.html]

C-Index


JavaScript is disabled on your browser.


Skip navigation links


- Package
- Class
- Use
- Tree
- Deprecated
- Index
- Help

- Prev Letter
- Next Letter

- Frames
- No Frames

- All Classes

A C D E F G I K L M N O P R S T V W Z 


## C

canReEncrypt() - Method in class edu.ecu.hsim.ray.proxyreencryption.Cipher
:   Returns `true` if can re-encrypt, `false` otherwise.

Cipher - Class in edu.ecu.hsim.ray.proxyreencryption
:   Stores enciphered data as a list of elements and lengths.

Cipher(ProxyReEncryption, Cipher.LEVEL) - Constructor for class edu.ecu.hsim.ray.proxyreencryption.Cipher
:   Constructor.

Cipher(ProxyReEncryption, Cipher.LEVEL, Element) - Constructor for class edu.ecu.hsim.ray.proxyreencryption.Cipher
:   Re-encryption constructor.

Cipher(ProxyReEncryption, Cipher.LEVEL, Cipher, Element) - Constructor for class edu.ecu.hsim.ray.proxyreencryption.Cipher
:   Re-encryption constructor.

Cipher(ProxyReEncryption, Cipher.LEVEL, Element, Element) - Constructor for class edu.ecu.hsim.ray.proxyreencryption.Cipher
:   Re-encryption constructor for sign/encrypt-sign constructor.

Cipher(ProxyReEncryption, Cipher.LEVEL, Cipher, Element, Element) - Constructor for class edu.ecu.hsim.ray.proxyreencryption.Cipher
:   Re-encryption constructor for two-party decryption This sets the
    intermediate's scalar and delegatee's re-encryption, which is used during
    the decryption process by the recipient.

Cipher.LEVEL - Enum in edu.ecu.hsim.ray.proxyreencryption
:   Flags each `Cipher` with the appropriate level.

Cipher.PAIRING\_TYPE - Enum in edu.ecu.hsim.ray.proxyreencryption
:   When loading a pairing `Element`, one can specify if it is a
    formatted or unformatted byte array.

CIPHER\_EXT - Static variable in class edu.ecu.hsim.ray.proxyreencryption.Constants
:   Cipher file extension.

close() - Method in class edu.ecu.hsim.ray.proxyreencryption.Keys
:   Closes the file input and output streams.

code - Variable in enum edu.ecu.hsim.ray.proxyreencryption.ProxyReEncryption.ENCRYPTION\_MODE
:   Code.

Constants - Class in edu.ecu.hsim.ray.proxyreencryption
:   Constants.

Constants() - Constructor for class edu.ecu.hsim.ray.proxyreencryption.Constants


CURVE\_PARAMS\_EXT - Static variable in class edu.ecu.hsim.ray.proxyreencryption.Constants
:   Curve parameters file extension.

curveFile - Variable in class edu.ecu.hsim.ray.proxyreencryption.ProxyReEncryption
:   Curve `File` - if nonvolatile.

curveParams - Variable in class edu.ecu.hsim.ray.proxyreencryption.ProxyReEncryption
:   Curve `PairingParameters`.

curveProperties - Variable in class edu.ecu.hsim.ray.proxyreencryption.ProxyReEncryption
:   Curve properties - if volatile.

A C D E F G I K L M N O P R S T V W Z

Skip navigation links


- Package
- Class
- Use
- Tree
- Deprecated
- Index
- Help

- Prev Letter
- Next Letter

- Frames
- No Frames

- All Classes
