# Supplementary material for: A Blockchain Framework for Patient-Centered Health Records and Exchange (HealthChain): Evaluation and Proof-of-Concept Study
Source: J Med Internet Res. 2019 Aug 31;21(8):e13592. doi: 10.2196/13592 (PMC6743266; doi:10.2196/13592)
Supplement: Multimedia Appendix 3 [file jmir_v21i8e13592_app3.zip › ProxyReEncryption/javadoc/index-files/index-3.html]

D-Index


JavaScript is disabled on your browser.


Skip navigation links


- Package
- Class
- Use
- Tree
- Deprecated
- Index
- Help

- Prev Letter
- Next Letter

- Frames
- No Frames

- All Classes

A C D E F G I K L M N O P R S T V W Z 


## D

decrypt(Keys, Cipher) - Method in class edu.ecu.hsim.ray.proxyreencryption.AFGH


decrypt(Keys, Message) - Method in class edu.ecu.hsim.ray.proxyreencryption.AFGH


decrypt(Element, Cipher) - Method in class edu.ecu.hsim.ray.proxyreencryption.AFGH


decrypt(Element, Message) - Method in class edu.ecu.hsim.ray.proxyreencryption.AFGH


decrypt(Keys, Cipher) - Method in class edu.ecu.hsim.ray.proxyreencryption.ProxyReEncryption
:   Decrypts the `Cipher` using the provided private key.

decrypt(Keys, Message) - Method in class edu.ecu.hsim.ray.proxyreencryption.ProxyReEncryption
:   Decrypts the `Message` using the provided private key.

decrypt(Element, Cipher) - Method in class edu.ecu.hsim.ray.proxyreencryption.ProxyReEncryption
:   Decrypts the `Cipher` using the provided ephemeral key `x`.

decrypt(Element, Message) - Method in class edu.ecu.hsim.ray.proxyreencryption.ProxyReEncryption
:   Decrypts the `Message` using the provided ephemeral key `x`.

decryptDelegatee(Keys, Cipher) - Method in class edu.ecu.hsim.ray.proxyreencryption.AFGH


decryptDelegatee(Keys, Cipher) - Method in class edu.ecu.hsim.ray.proxyreencryption.ProxyReEncryption
:   Decrypts the delegatee layer of a two-party re-encryption `Cipher`
    using the provided private key.

decryptIntermediate(Keys, Cipher) - Method in class edu.ecu.hsim.ray.proxyreencryption.AFGH


decryptIntermediate(Keys, Cipher) - Method in class edu.ecu.hsim.ray.proxyreencryption.ProxyReEncryption
:   Decrypts the intermediate layer of a two-party re-encryption
    `Cipher` using the provided private key.

decryptionParameter - Variable in class edu.ecu.hsim.ray.proxyreencryption.Cipher
:   Decryption parameter - re-encryption/two-party/sign/encrypt-sign

decryptToCipher(Keys, Cipher) - Method in class edu.ecu.hsim.ray.proxyreencryption.AFGH


decryptToCipher(Element, Cipher) - Method in class edu.ecu.hsim.ray.proxyreencryption.AFGH


decryptToCipher(Keys, Cipher) - Method in class edu.ecu.hsim.ray.proxyreencryption.ProxyReEncryption
:   Decrypts the `Cipher` to a `Cipher` using the provided
    private key.

decryptToCipher(Element, Cipher) - Method in class edu.ecu.hsim.ray.proxyreencryption.ProxyReEncryption
:   Decrypts the `Cipher` to a `Cipher` using the provided
    ephemeral key `x`.

decryptVerifyImpl(Keys, Keys, Message) - Method in class edu.ecu.hsim.ray.proxyreencryption.AFGH
:   Decrypt-verifies the `cipher` using the private key of the
    recipient (`decrypt`) and public key of the signer
    (`verify`), returning the decrypt-verified cipher as a
    `Message`.

DEFAULT\_ENCRYPTION\_MODE - Static variable in class edu.ecu.hsim.ray.proxyreencryption.ProxyReEncryption
:   Default `ProxyReEncryption.ENCRYPTION_MODE`.

DEFAULT\_REENCRYPT - Variable in class edu.ecu.hsim.ray.proxyreencryption.ProxyReEncryption
:   Default `ProxyReEncryption.REENCRYPT`.

DOI - Static variable in class edu.ecu.hsim.ray.proxyreencryption.Constants
:   Sample text - the Declaration of Independence.

A C D E F G I K L M N O P R S T V W Z

Skip navigation links


- Package
- Class
- Use
- Tree
- Deprecated
- Index
- Help

- Prev Letter
- Next Letter

- Frames
- No Frames

- All Classes
