# Supplementary material for: A Blockchain Framework for Patient-Centered Health Records and Exchange (HealthChain): Evaluation and Proof-of-Concept Study
Source: J Med Internet Res. 2019 Aug 31;21(8):e13592. doi: 10.2196/13592 (PMC6743266; doi:10.2196/13592)
Supplement: Multimedia Appendix 3 [file jmir_v21i8e13592_app3.zip › ProxyReEncryption/javadoc/index-files/index-4.html]

E-Index


JavaScript is disabled on your browser.


Skip navigation links


- Package
- Class
- Use
- Tree
- Deprecated
- Index
- Help

- Prev Letter
- Next Letter

- Frames
- No Frames

- All Classes

A C D E F G I K L M N O P R S T V W Z 


## E

e - Variable in class edu.ecu.hsim.ray.proxyreencryption.ProxyReEncryption
:   `Pairing` `e`.

edu.ecu.hsim.ray.proxyreencryption - package edu.ecu.hsim.ray.proxyreencryption


elements - Variable in class edu.ecu.hsim.ray.proxyreencryption.Cipher
:   Cipher `Elements`.

ENC\_MODE\_PREFIX - Static variable in class edu.ecu.hsim.ray.proxyreencryption.Constants
:   Encryption mode key.

encMode - Variable in class edu.ecu.hsim.ray.proxyreencryption.ProxyReEncryption
:   `ProxyReEncryption.ENCRYPTION_MODE` parameter.

encModeHM - Static variable in class edu.ecu.hsim.ray.proxyreencryption.ProxyReEncryption
:   Maps `ProxyReEncryption.ENCRYPTION_MODE.code` to `ProxyReEncryption.ENCRYPTION_MODE`.

ENCODING - Static variable in class edu.ecu.hsim.ray.proxyreencryption.Constants
:   `Charset` encoding.

encrypt(Keys, String) - Method in class edu.ecu.hsim.ray.proxyreencryption.AFGH


encrypt(Keys, byte[], ProxyReEncryption.MESSAGE\_TYPE) - Method in class edu.ecu.hsim.ray.proxyreencryption.AFGH


encrypt(Keys, Message) - Method in class edu.ecu.hsim.ray.proxyreencryption.AFGH


encrypt(Keys, Cipher) - Method in class edu.ecu.hsim.ray.proxyreencryption.AFGH


encrypt(Keys, String, ProxyReEncryption.REENCRYPT) - Method in class edu.ecu.hsim.ray.proxyreencryption.AFGH


encrypt(Keys, byte[], ProxyReEncryption.MESSAGE\_TYPE, ProxyReEncryption.REENCRYPT) - Method in class edu.ecu.hsim.ray.proxyreencryption.AFGH


encrypt(Keys, Message, ProxyReEncryption.REENCRYPT) - Method in class edu.ecu.hsim.ray.proxyreencryption.AFGH


encrypt(Keys, Cipher, ProxyReEncryption.REENCRYPT) - Method in class edu.ecu.hsim.ray.proxyreencryption.AFGH


encrypt(Element, String) - Method in class edu.ecu.hsim.ray.proxyreencryption.AFGH


encrypt(Element, byte[], ProxyReEncryption.MESSAGE\_TYPE) - Method in class edu.ecu.hsim.ray.proxyreencryption.AFGH


encrypt(Element, Message) - Method in class edu.ecu.hsim.ray.proxyreencryption.AFGH


encrypt(Element, Cipher) - Method in class edu.ecu.hsim.ray.proxyreencryption.AFGH


encrypt(Keys, String) - Method in class edu.ecu.hsim.ray.proxyreencryption.ProxyReEncryption
:   Encrypts the `message` using the public key.

encrypt(Keys, byte[], ProxyReEncryption.MESSAGE\_TYPE) - Method in class edu.ecu.hsim.ray.proxyreencryption.ProxyReEncryption
:   Encrypts the `message` using the public key.

encrypt(Keys, Message) - Method in class edu.ecu.hsim.ray.proxyreencryption.ProxyReEncryption
:   Encrypts the `Message` using the public key.

encrypt(Keys, Cipher) - Method in class edu.ecu.hsim.ray.proxyreencryption.ProxyReEncryption
:   Encrypts the `Cipher` using the public key.

encrypt(Keys, String, ProxyReEncryption.REENCRYPT) - Method in class edu.ecu.hsim.ray.proxyreencryption.ProxyReEncryption
:   Encrypts the `message` using the public key and `reencrypt`
    parameter.

encrypt(Keys, byte[], ProxyReEncryption.MESSAGE\_TYPE, ProxyReEncryption.REENCRYPT) - Method in class edu.ecu.hsim.ray.proxyreencryption.ProxyReEncryption
:   Encrypts the `message` using the public key and `reencrypt`
    parameter.

encrypt(Keys, Message, ProxyReEncryption.REENCRYPT) - Method in class edu.ecu.hsim.ray.proxyreencryption.ProxyReEncryption
:   Encrypts the `Message` using the public key and `reencrypt`
    parameter.

encrypt(Keys, Cipher, ProxyReEncryption.REENCRYPT) - Method in class edu.ecu.hsim.ray.proxyreencryption.ProxyReEncryption
:   Encrypts the `Cipher` using the public key and `reencrypt`
    parameter.

encrypt(Element, String) - Method in class edu.ecu.hsim.ray.proxyreencryption.ProxyReEncryption
:   Encrypts the `message` using the ephemeral key `x`.

encrypt(Element, byte[], ProxyReEncryption.MESSAGE\_TYPE) - Method in class edu.ecu.hsim.ray.proxyreencryption.ProxyReEncryption
:   Encrypts the `message` using the ephemeral key `x`.

encrypt(Element, Message) - Method in class edu.ecu.hsim.ray.proxyreencryption.ProxyReEncryption
:   Encrypts the `Message` using the ephemeral key `x`.

encrypt(Element, Cipher) - Method in class edu.ecu.hsim.ray.proxyreencryption.ProxyReEncryption
:   Encrypts the `Cipher` using the ephemeral key `x`.

EQ\_PREFIX\_I - Static variable in class edu.ecu.hsim.ray.proxyreencryption.Constants
:   Eq data key.

EQ\_PREFIX\_N - Static variable in class edu.ecu.hsim.ray.proxyreencryption.Constants
:   Eq Base64 size key.

A C D E F G I K L M N O P R S T V W Z

Skip navigation links


- Package
- Class
- Use
- Tree
- Deprecated
- Index
- Help

- Prev Letter
- Next Letter

- Frames
- No Frames

- All Classes
