# Supplementary material for: A Blockchain Framework for Patient-Centered Health Records and Exchange (HealthChain): Evaluation and Proof-of-Concept Study
Source: J Med Internet Res. 2019 Aug 31;21(8):e13592. doi: 10.2196/13592 (PMC6743266; doi:10.2196/13592)
Supplement: Multimedia Appendix 3 [file jmir_v21i8e13592_app3.zip › ProxyReEncryption/javadoc/index-files/index-5.html]

F-Index


JavaScript is disabled on your browser.


Skip navigation links


- Package
- Class
- Use
- Tree
- Deprecated
- Index
- Help

- Prev Letter
- Next Letter

- Frames
- No Frames

- All Classes

A C D E F G I K L M N O P R S T V W Z 


## F

firstLevel - Variable in class edu.ecu.hsim.ray.proxyreencryption.Keys
:   First-level `Element`.

fisPub - Variable in class edu.ecu.hsim.ray.proxyreencryption.Keys
:   Public properties `FileInputStream`.

fisSec - Variable in class edu.ecu.hsim.ray.proxyreencryption.Keys
:   Secret properties `FileInputStream`.

fosPub - Variable in class edu.ecu.hsim.ray.proxyreencryption.Keys
:   Public properties `FileOutputStream`.

fosSec - Variable in class edu.ecu.hsim.ray.proxyreencryption.Keys
:   Secret properties `FileOutputStream`.

A C D E F G I K L M N O P R S T V W Z

Skip navigation links


- Package
- Class
- Use
- Tree
- Deprecated
- Index
- Help

- Prev Letter
- Next Letter

- Frames
- No Frames

- All Classes
