# Supplementary material for: A Blockchain Framework for Patient-Centered Health Records and Exchange (HealthChain): Evaluation and Proof-of-Concept Study
Source: J Med Internet Res. 2019 Aug 31;21(8):e13592. doi: 10.2196/13592 (PMC6743266; doi:10.2196/13592)
Supplement: Multimedia Appendix 3 [file jmir_v21i8e13592_app3.zip › ProxyReEncryption/javadoc/index-files/index-6.html]

G-Index


JavaScript is disabled on your browser.


Skip navigation links


- Package
- Class
- Use
- Tree
- Deprecated
- Index
- Help

- Prev Letter
- Next Letter

- Frames
- No Frames

- All Classes

A C D E F G I K L M N O P R S T V W Z 


## G

G1 - Variable in class edu.ecu.hsim.ray.proxyreencryption.ProxyReEncryption
:   `Field` `G1`.

g1 - Variable in class edu.ecu.hsim.ray.proxyreencryption.ProxyReEncryption
:   `Element` `g1` generator.

g1p - Variable in class edu.ecu.hsim.ray.proxyreencryption.ProxyReEncryption
:   Generator 1 power pre-processing (`ElementPowPreProcessing`).

G1P\_PREFIX - Static variable in class edu.ecu.hsim.ray.proxyreencryption.Constants
:   G1 preprocessing key

G1X\_PREFIX - Static variable in class edu.ecu.hsim.ray.proxyreencryption.Constants
:   G1.x key.

G1Y\_PREFIX - Static variable in class edu.ecu.hsim.ray.proxyreencryption.Constants
:   G1.y key.

G2 - Variable in class edu.ecu.hsim.ray.proxyreencryption.ProxyReEncryption
:   `Field` `G2`.

g2 - Variable in class edu.ecu.hsim.ray.proxyreencryption.ProxyReEncryption
:   `Element` `g2` generator.

g2p - Variable in class edu.ecu.hsim.ray.proxyreencryption.ProxyReEncryption
:   Generator 2 power pre-processing (`ElementPowPreProcessing`).

G2P\_PREFIX - Static variable in class edu.ecu.hsim.ray.proxyreencryption.Constants
:   G2 preprocessing key.

G2X\_PREFIX - Static variable in class edu.ecu.hsim.ray.proxyreencryption.Constants
:   G2.x key.

G2Y\_PREFIX - Static variable in class edu.ecu.hsim.ray.proxyreencryption.Constants
:   G2.y key.

GEN\_PREFIX\_I - Static variable in class edu.ecu.hsim.ray.proxyreencryption.Constants
:   Generator data key.

GEN\_PREFIX\_N - Static variable in class edu.ecu.hsim.ray.proxyreencryption.Constants
:   Generator Base64 size key.

getByteLength() - Method in class edu.ecu.hsim.ray.proxyreencryption.Message
:   Returns the byte length.

getCurveFile() - Method in class edu.ecu.hsim.ray.proxyreencryption.ProxyReEncryption
:   Returns the curve file if `ProxyReEncryption.storage` ==
    `ProxyReEncryption.STORAGE.NONVOLATILE`.

getCurveParams() - Method in class edu.ecu.hsim.ray.proxyreencryption.ProxyReEncryption
:   Returns the curve parameters.

getCurveProperties() - Method in class edu.ecu.hsim.ray.proxyreencryption.ProxyReEncryption
:   Returns the curve properties if `ProxyReEncryption.storage` ==
    `ProxyReEncryption.STORAGE.VOLATILE`.

getDecryptFirstLevelDenominator() - Method in class edu.ecu.hsim.ray.proxyreencryption.Keys
:   Returns the first-level decryption denominator as an immutable
    `Element`.

getDecryptionParameter() - Method in class edu.ecu.hsim.ray.proxyreencryption.Cipher
:   Returns the decryption parameter element (for decrypt-verify).

getDecryptSecondLevelDenominator() - Method in class edu.ecu.hsim.ray.proxyreencryption.Keys
:   Returns the second-level decryption denominator as an immutable
    `Element`.

getDefaultReEncrypt() - Method in class edu.ecu.hsim.ray.proxyreencryption.ProxyReEncryption
:   Returns the default re-encryption parameter.

getE() - Method in class edu.ecu.hsim.ray.proxyreencryption.ProxyReEncryption
:   Returns `e`.

getElements() - Method in class edu.ecu.hsim.ray.proxyreencryption.Cipher
:   Returns the list of elements.

getElements() - Method in class edu.ecu.hsim.ray.proxyreencryption.Message
:   Returns the list of elements.

getEncryptionMode() - Method in class edu.ecu.hsim.ray.proxyreencryption.ProxyReEncryption
:   Returns the `ProxyReEncryption.ENCRYPTION_MODE`.

getEq() - Method in class edu.ecu.hsim.ray.proxyreencryption.TypeAPairingIO
:   Returns `Eq`.

getFirstLevel() - Method in class edu.ecu.hsim.ray.proxyreencryption.Keys
:   Returns `Keys.firstLevel`.

getFq() - Method in class edu.ecu.hsim.ray.proxyreencryption.TypeAPairingIO
:   Returns `Fq`.

getG1Gen() - Method in class edu.ecu.hsim.ray.proxyreencryption.ProxyReEncryption
:   Returns the generator `g1` for `G1`.

getG1GenP() - Method in class edu.ecu.hsim.ray.proxyreencryption.ProxyReEncryption
:   Returns pow pre processing element `g1p` for `G1`.

getG2Gen() - Method in class edu.ecu.hsim.ray.proxyreencryption.ProxyReEncryption
:   Returns the generator `g2` for `G2`.

getG2GenP() - Method in class edu.ecu.hsim.ray.proxyreencryption.ProxyReEncryption
:   Returns pow pre processing element `g2p` for `G2`.

getGT() - Method in class edu.ecu.hsim.ray.proxyreencryption.ProxyReEncryption
:   Returns `GT`.

getInfFlag(CurveElement) - Method in class edu.ecu.hsim.ray.proxyreencryption.ProxyReEncryption
:   Returns `infFlag` for the `CurveElement` `ce`.

getK() - Method in class edu.ecu.hsim.ray.proxyreencryption.Keys
:   Returns a static `Keys.k`.

getL() - Method in class edu.ecu.hsim.ray.proxyreencryption.Keys
:   Returns `Keys.l`.

getLength() - Method in class edu.ecu.hsim.ray.proxyreencryption.Message
:   Returns the total length of all elements.

getLengths() - Method in class edu.ecu.hsim.ray.proxyreencryption.Cipher
:   Returns the list of lengths.

getLengths() - Method in class edu.ecu.hsim.ray.proxyreencryption.Message
:   Returns the list of lengths.

getLevel() - Method in class edu.ecu.hsim.ray.proxyreencryption.Cipher
:   Returns the `Cipher.LEVEL`.

getLevel() - Method in enum edu.ecu.hsim.ray.proxyreencryption.ProxyReEncryption.REENCRYPT
:   Returns the `Cipher.LEVEL`.

getLogLevel() - Method in class edu.ecu.hsim.ray.proxyreencryption.ProxyReEncryption
:   Returns the log level.

getOrder() - Method in class edu.ecu.hsim.ray.proxyreencryption.ProxyReEncryption
:   Returns the order.

getOrder() - Method in class edu.ecu.hsim.ray.proxyreencryption.TypeAPairingIO
:   Returns the order.

getProxy() - Method in class edu.ecu.hsim.ray.proxyreencryption.Keys
:   Returns the `ProxyReEncryption` object associated with this
    `Keys` object.

getPublicFile() - Method in class edu.ecu.hsim.ray.proxyreencryption.Keys
:   Returns the public file if `Keys.storage` ==
    `ProxyReEncryption.STORAGE.NONVOLATILE`.

getPublicKey() - Method in class edu.ecu.hsim.ray.proxyreencryption.Keys
:   Returns the public key.

getPublicProperties() - Method in class edu.ecu.hsim.ray.proxyreencryption.Keys
:   Returns public properties as a `String`.

getReencryptionPairing() - Method in class edu.ecu.hsim.ray.proxyreencryption.Cipher
:   Returns the re-encryption pairing element.

getReencryptionScalar() - Method in class edu.ecu.hsim.ray.proxyreencryption.Cipher
:   Returns the intermediate re-encryption scalar element.

getSecondLevel() - Method in class edu.ecu.hsim.ray.proxyreencryption.Keys
:   Returns `Keys.secondLevel`.

getSecretFile() - Method in class edu.ecu.hsim.ray.proxyreencryption.Keys
:   Returns the secret file if `Keys.storage` ==
    `ProxyReEncryption.STORAGE.NONVOLATILE`.

getSecretKey() - Method in class edu.ecu.hsim.ray.proxyreencryption.Keys
:   Returns the secret key.

getSecretKeyInverse() - Method in class edu.ecu.hsim.ray.proxyreencryption.Keys
:   Returns the inverse of the secret key.

getSecretProperties() - Method in class edu.ecu.hsim.ray.proxyreencryption.Keys
:   Returns secret properties as a `String`.

getSignedHash() - Method in class edu.ecu.hsim.ray.proxyreencryption.Cipher
:   Returns the hash for signed/encrypt-signed messages.

getTwoPartyScalar() - Method in class edu.ecu.hsim.ray.proxyreencryption.Keys
:   Returns `Keys.twoPartyScalar`.

getZP() - Method in class edu.ecu.hsim.ray.proxyreencryption.ProxyReEncryption
:   Returns `Zp`.

getZr() - Method in class edu.ecu.hsim.ray.proxyreencryption.ProxyReEncryption
:   Returns `Zr`.

GT - Variable in class edu.ecu.hsim.ray.proxyreencryption.ProxyReEncryption
:   `Field` `GT`.

A C D E F G I K L M N O P R S T V W Z

Skip navigation links


- Package
- Class
- Use
- Tree
- Deprecated
- Index
- Help

- Prev Letter
- Next Letter

- Frames
- No Frames

- All Classes
