# Supplementary material for: A Blockchain Framework for Patient-Centered Health Records and Exchange (HealthChain): Evaluation and Proof-of-Concept Study
Source: J Med Internet Res. 2019 Aug 31;21(8):e13592. doi: 10.2196/13592 (PMC6743266; doi:10.2196/13592)
Supplement: Multimedia Appendix 3 [file jmir_v21i8e13592_app3.zip › ProxyReEncryption/javadoc/index-files/index-8.html]

K-Index


JavaScript is disabled on your browser.


Skip navigation links


- Package
- Class
- Use
- Tree
- Deprecated
- Index
- Help

- Prev Letter
- Next Letter

- Frames
- No Frames

- All Classes

A C D E F G I K L M N O P R S T V W Z 


## K

k - Variable in class edu.ecu.hsim.ray.proxyreencryption.Keys
:   Secret parameter `k` `Element`.

KEY\_K - Static variable in class edu.ecu.hsim.ray.proxyreencryption.Constants
:   K key.

KEY\_L - Static variable in class edu.ecu.hsim.ray.proxyreencryption.Constants
:   L key.

KEY\_V - Static variable in class edu.ecu.hsim.ray.proxyreencryption.Constants
:   Secret key `BigInteger` key.

KEY\_Z - Static variable in class edu.ecu.hsim.ray.proxyreencryption.Constants
:   Secret key Z field order key.

Keys - Class in edu.ecu.hsim.ray.proxyreencryption
:   Generates/reads public and secret (private) keys.

Keys() - Constructor for class edu.ecu.hsim.ray.proxyreencryption.Keys
:   Empty constructor used by `ReKeys` as it follows a different
    process during its construction.

Keys(ProxyReEncryption, boolean) - Constructor for class edu.ecu.hsim.ray.proxyreencryption.Keys
:   Constructor for curve parameter generation testing.

Keys(ProxyReEncryption) - Constructor for class edu.ecu.hsim.ray.proxyreencryption.Keys
:   Constructs a new `ProxyReEncryption.STORAGE.VOLATILE` set of `Keys`.

Keys(ProxyReEncryption, String) - Constructor for class edu.ecu.hsim.ray.proxyreencryption.Keys
:   Constructs a new `ProxyReEncryption.STORAGE.NONVOLATILE` set of `Keys`.

Keys(ProxyReEncryption, byte[], byte[]) - Constructor for class edu.ecu.hsim.ray.proxyreencryption.Keys
:   Constructs a new `ProxyReEncryption.STORAGE.VOLATILE` set of `Keys`.

Keys(ProxyReEncryption, String, String) - Constructor for class edu.ecu.hsim.ray.proxyreencryption.Keys
:   Constructs a new `ProxyReEncryption.STORAGE.VOLATILE` set of `Keys`.

A C D E F G I K L M N O P R S T V W Z

Skip navigation links


- Package
- Class
- Use
- Tree
- Deprecated
- Index
- Help

- Prev Letter
- Next Letter

- Frames
- No Frames

- All Classes
