# Supplementary material for: A Blockchain Framework for Patient-Centered Health Records and Exchange (HealthChain): Evaluation and Proof-of-Concept Study
Source: J Med Internet Res. 2019 Aug 31;21(8):e13592. doi: 10.2196/13592 (PMC6743266; doi:10.2196/13592)
Supplement: Multimedia Appendix 3 [file jmir_v21i8e13592_app3.zip › ProxyReEncryption/javadoc/index.html]

Generated Documentation (Untitled)


<noscript>
<div>JavaScript is disabled on your browser.</div>
</noscript>
<h2>Frame Alert</h2>
<p>This document is designed to be viewed using the frames feature. If you see this message, you are using a non-frame-capable web client. Link to <a href="edu/ecu/hsim/ray/proxyreencryption/package-summary.html">Non-frame version</a>.</p>
