# Supplementary material for: A Blockchain Framework for Patient-Centered Health Records and Exchange (HealthChain): Evaluation and Proof-of-Concept Study
Source: J Med Internet Res. 2019 Aug 31;21(8):e13592. doi: 10.2196/13592 (PMC6743266; doi:10.2196/13592)
Supplement: Multimedia Appendix 3 [file jmir_v21i8e13592_app3.zip › ProxyReEncryption/javadoc/overview-tree.html]

Class Hierarchy


JavaScript is disabled on your browser.


Skip navigation links


- Package
- Class
- Use
- Tree
- Deprecated
- Index
- Help

- Prev
- Next

- Frames
- No Frames

- All Classes

# Hierarchy For All Packages

Package Hierarchies:

- edu.ecu.hsim.ray.proxyreencryption

## Class Hierarchy

- java.lang.Object
  - it.unisa.dia.gas.plaf.jpbc.pairing.AbstractPairing (implements it.unisa.dia.gas.jpbc.Pairing)
    - it.unisa.dia.gas.plaf.jpbc.pairing.a.TypeAPairing
      - edu.ecu.hsim.ray.proxyreencryption.TypeAPairingIO
  - edu.ecu.hsim.ray.proxyreencryption.Cipher
  - edu.ecu.hsim.ray.proxyreencryption.Constants
  - edu.ecu.hsim.ray.proxyreencryption.Keys
    - edu.ecu.hsim.ray.proxyreencryption.ReKeys
  - edu.ecu.hsim.ray.proxyreencryption.Message
  - edu.ecu.hsim.ray.proxyreencryption.ProxyReEncryption
    - edu.ecu.hsim.ray.proxyreencryption.AFGH

## Enum Hierarchy

- java.lang.Object
  - java.lang.Enum<E> (implements java.lang.Comparable<T>, java.io.Serializable)
    - edu.ecu.hsim.ray.proxyreencryption.AFGH.LENGTH
    - edu.ecu.hsim.ray.proxyreencryption.Cipher.PAIRING\_TYPE
    - edu.ecu.hsim.ray.proxyreencryption.Cipher.LEVEL
    - edu.ecu.hsim.ray.proxyreencryption.ProxyReEncryption.STORAGE
    - edu.ecu.hsim.ray.proxyreencryption.ProxyReEncryption.ENCRYPTION\_MODE
    - edu.ecu.hsim.ray.proxyreencryption.ProxyReEncryption.MESSAGE\_TYPE
    - edu.ecu.hsim.ray.proxyreencryption.ProxyReEncryption.REENCRYPT
    - edu.ecu.hsim.ray.proxyreencryption.ProxyReEncryption.SCALE

Skip navigation links


- Package
- Class
- Use
- Tree
- Deprecated
- Index
- Help

- Prev
- Next

- Frames
- No Frames

- All Classes
